# Supplementary material for: Inhibiting host-protein deposition on urinary catheters reduces associated urinary tract infections
Source: eLife. 2022 Mar 29;11:e75798. doi: 10.7554/eLife.75798 (PMC8986317; doi:10.7554/eLife.75798)
Supplement: Figure 6—source data 1. — Individual data from all figures involving small datasets displayed in individual tabs of this source file. This includes Figures 1B and 2A-F, Figure 3B, Figure 4, Figure 1—figure supplement 1 and Figure 2—figure supplement 1. [file elife-75798-fig6-data1.zip › Flores_Data/AF1_limma-graphs_AFCat1.pdf]

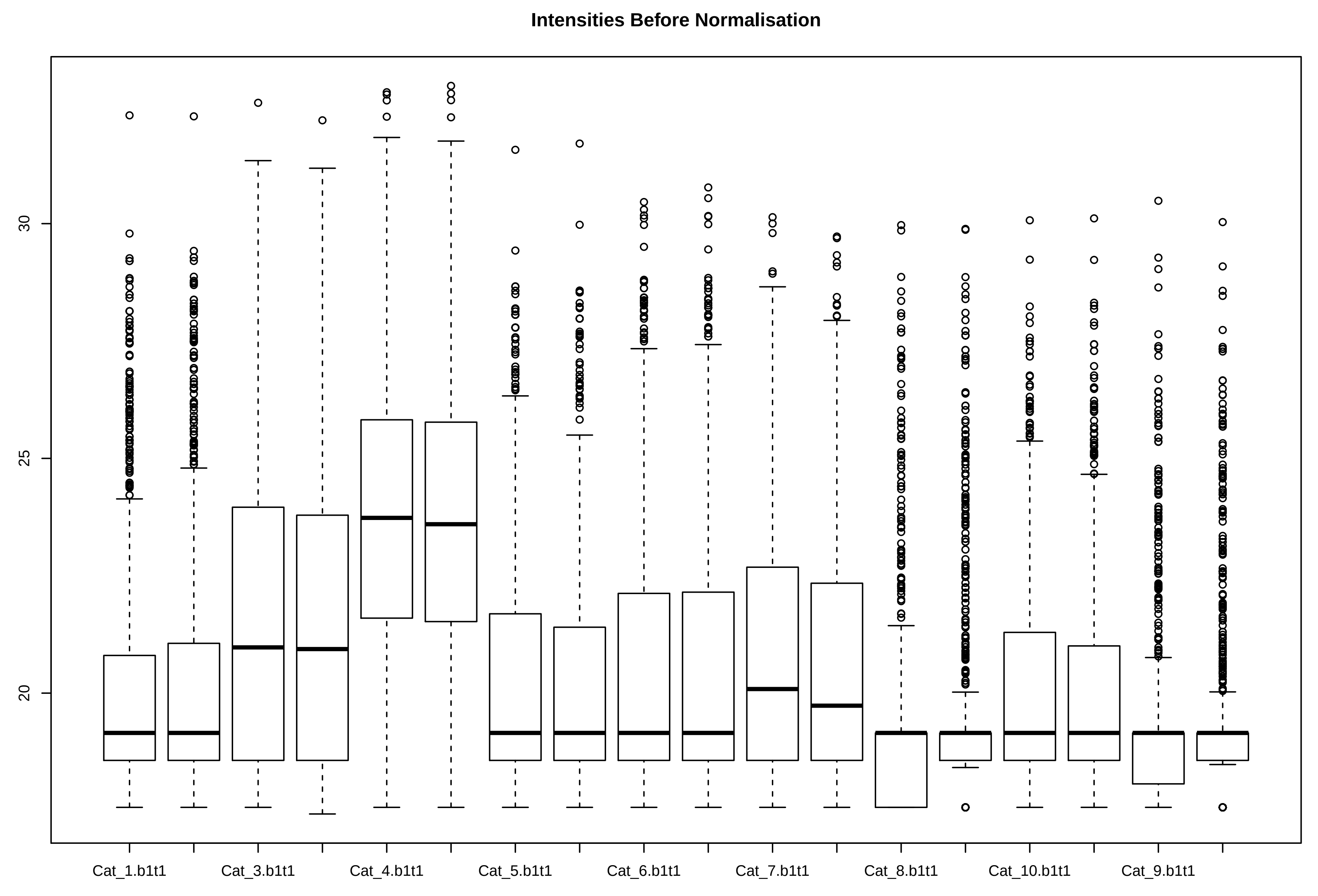
Intensities Before Normalisation
30
25
20
Cat_1.b1t1
Cat_3.b1t1
Cat_4.b1t1
Cat_5.b1t1
Cat_6.b1t1
Cat_7.b1t1
Cat_8.b1t1
Cat_10.b1t1
Cat_9.b1t1

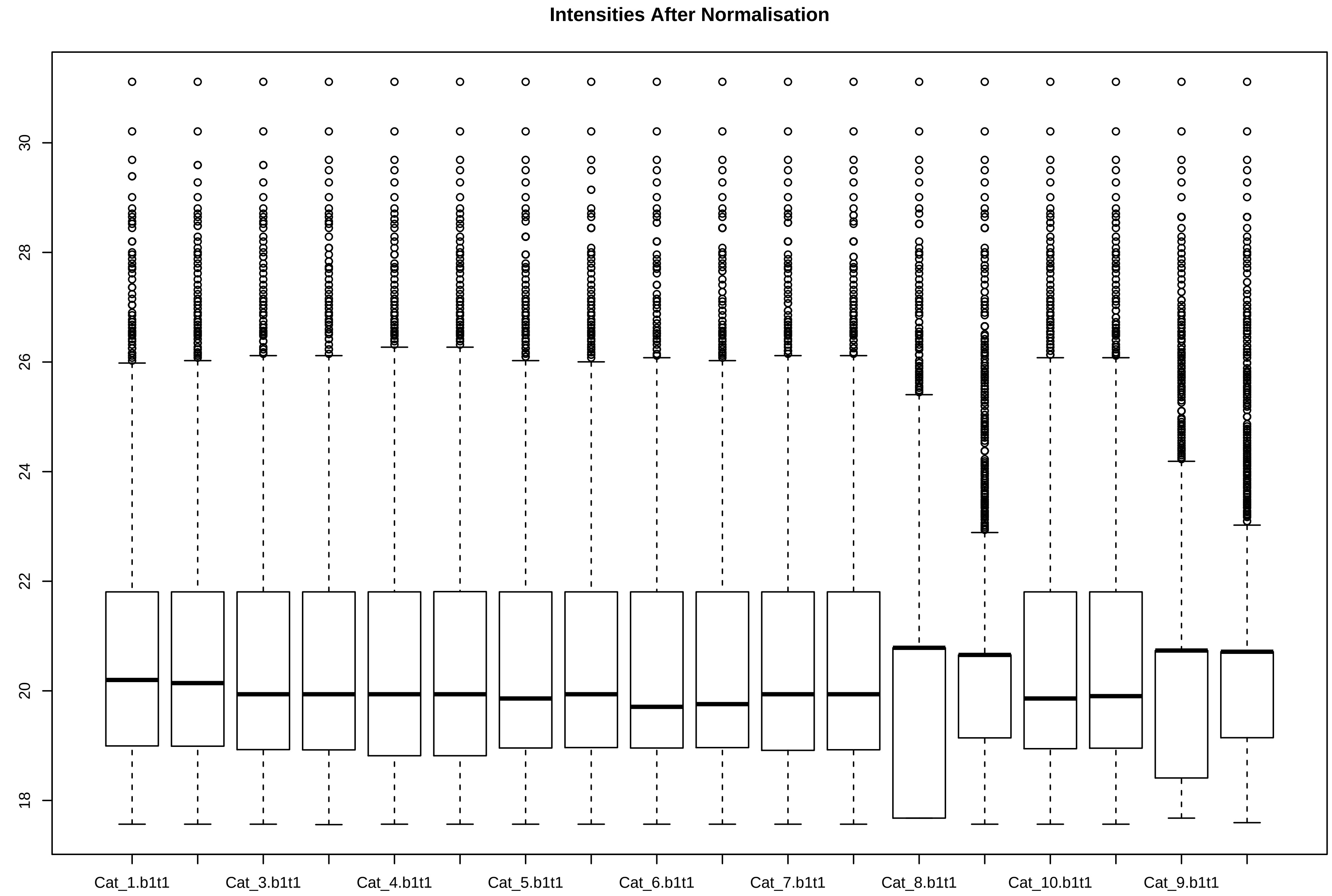
Intensities After Normalisation
18
20
22
24
26
28
30
Cat_1.b1t1
Cat_3.b1t1
Cat_4.b1t1
Cat_5.b1t1
Cat_6.b1t1
Cat_7.b1t1
Cat_8.b1t1
Cat_10.b1t1
Cat_9.b1t1

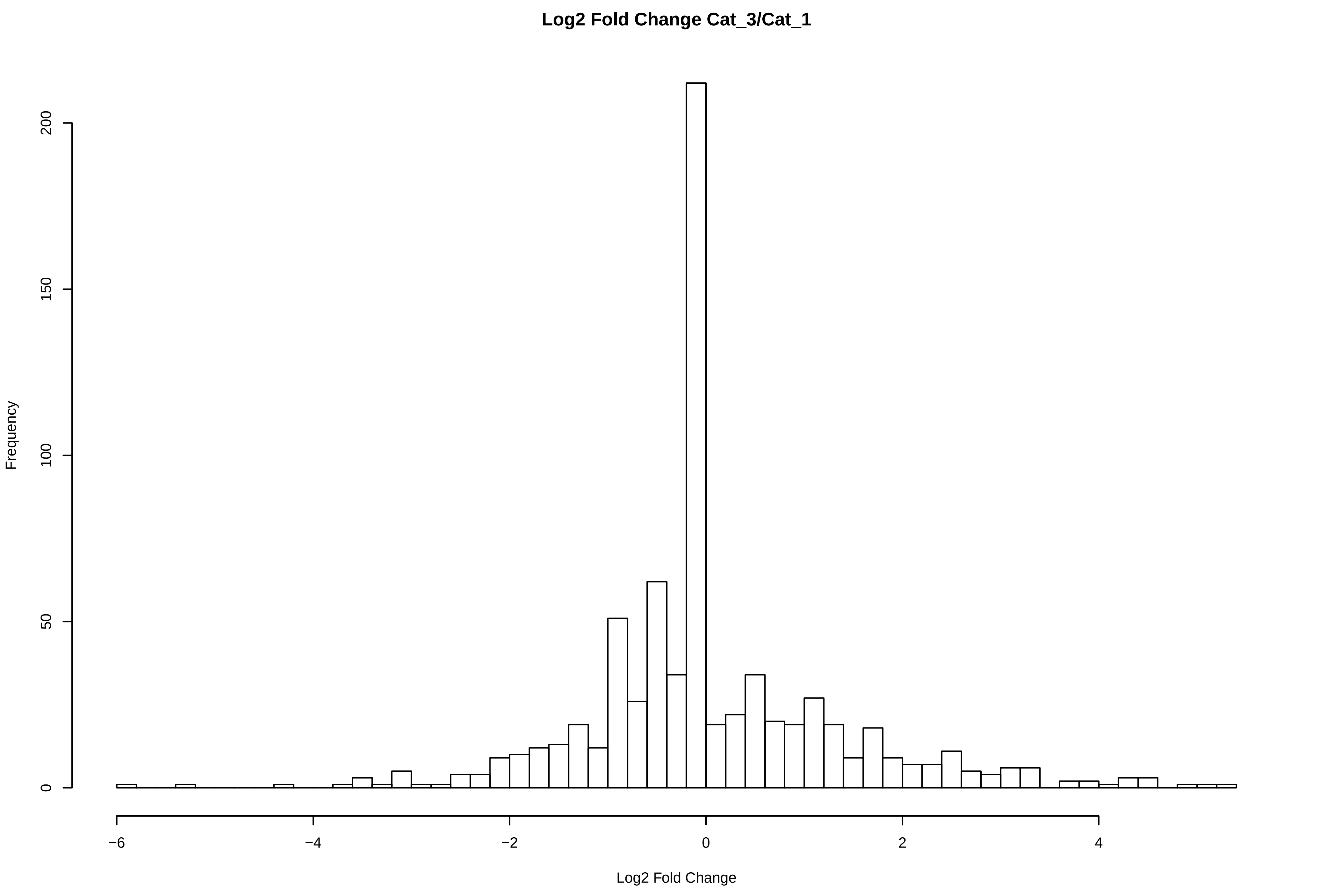
Log2 Fold Change Cat_3/Cat_1
Frequency
0
50
100
150
200
−6
−4
−2
0
2
4
Log2 Fold Change

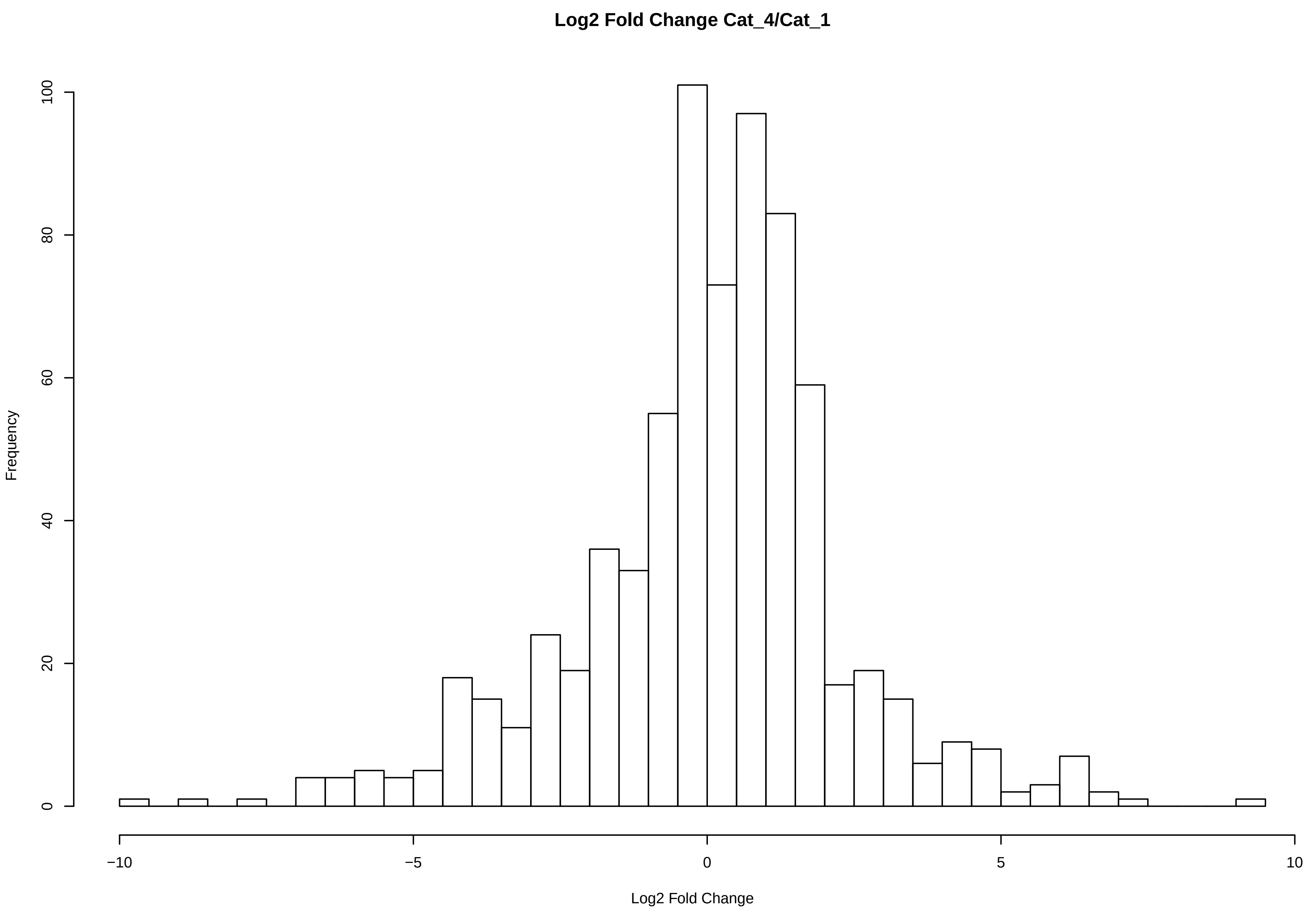
Log2 Fold Change Cat_4/Cat_1
Frequency
0
20
40
60
80
100
−10
−5
0
5
10
Log2 Fold Change

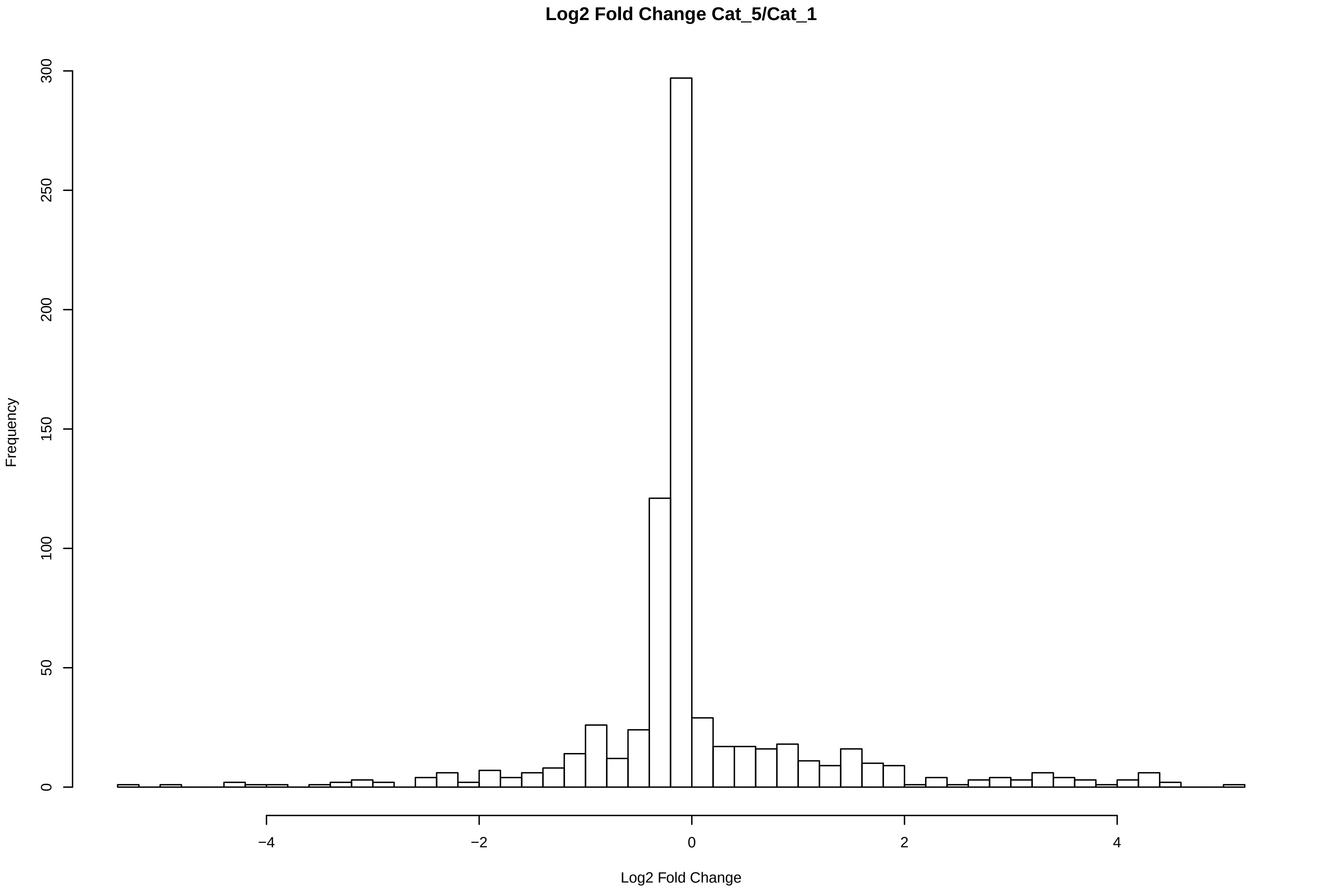

Log2 Fold Change Cat_5/Cat_1
Frequency
0
50
100
150
200
250
300
−4
−2
0
2
4
Log2 Fold Change

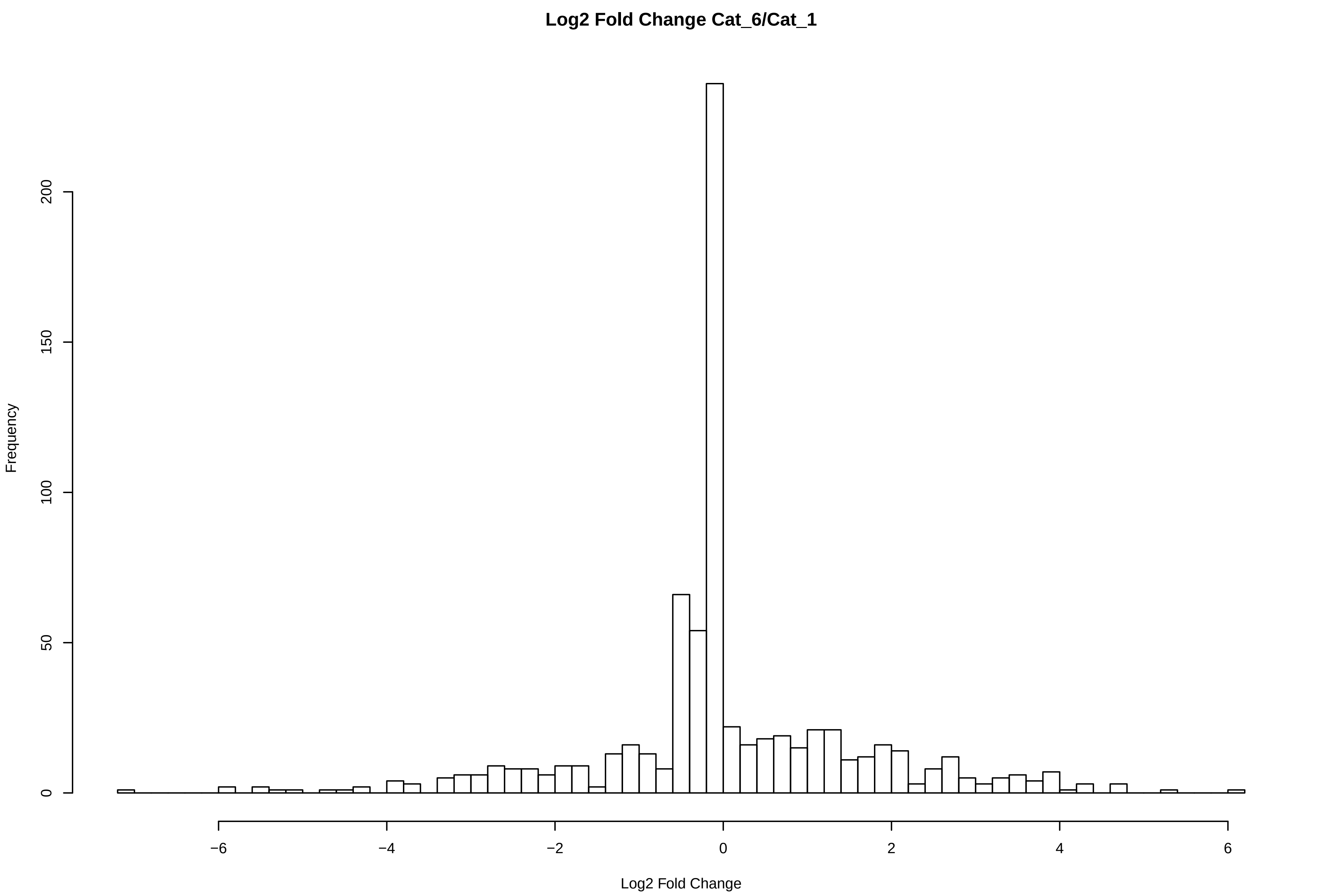

Log2 Fold Change Cat_6/Cat_1
Frequency
0
50
100
150
200
−6
−4
−2
0
2
4
6
Log2 Fold Change

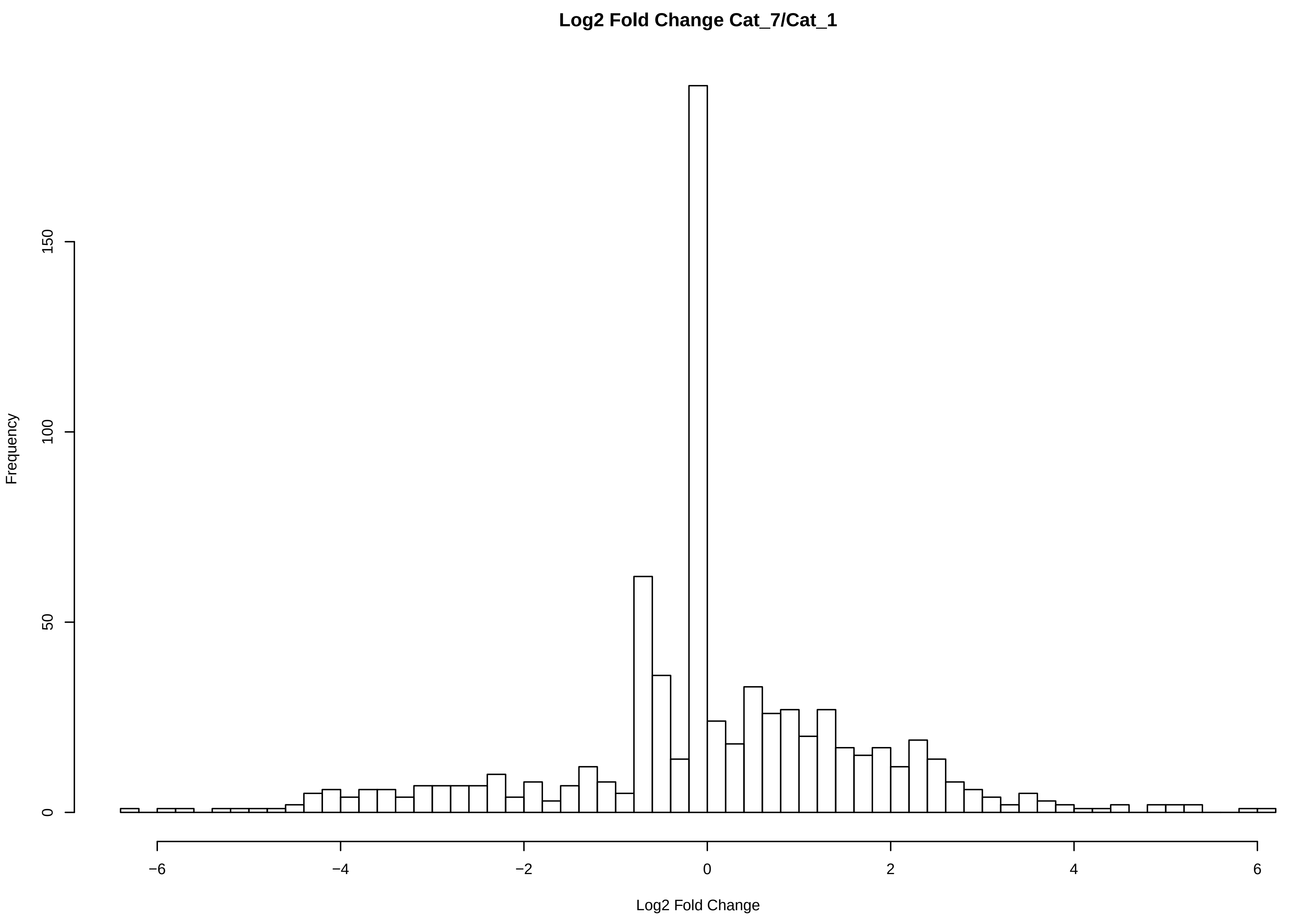
Log2 Fold Change Cat_7/Cat_1
Frequency
0
50
100
150
−6
−4
−2
0
2
4
6
Log2 Fold Change

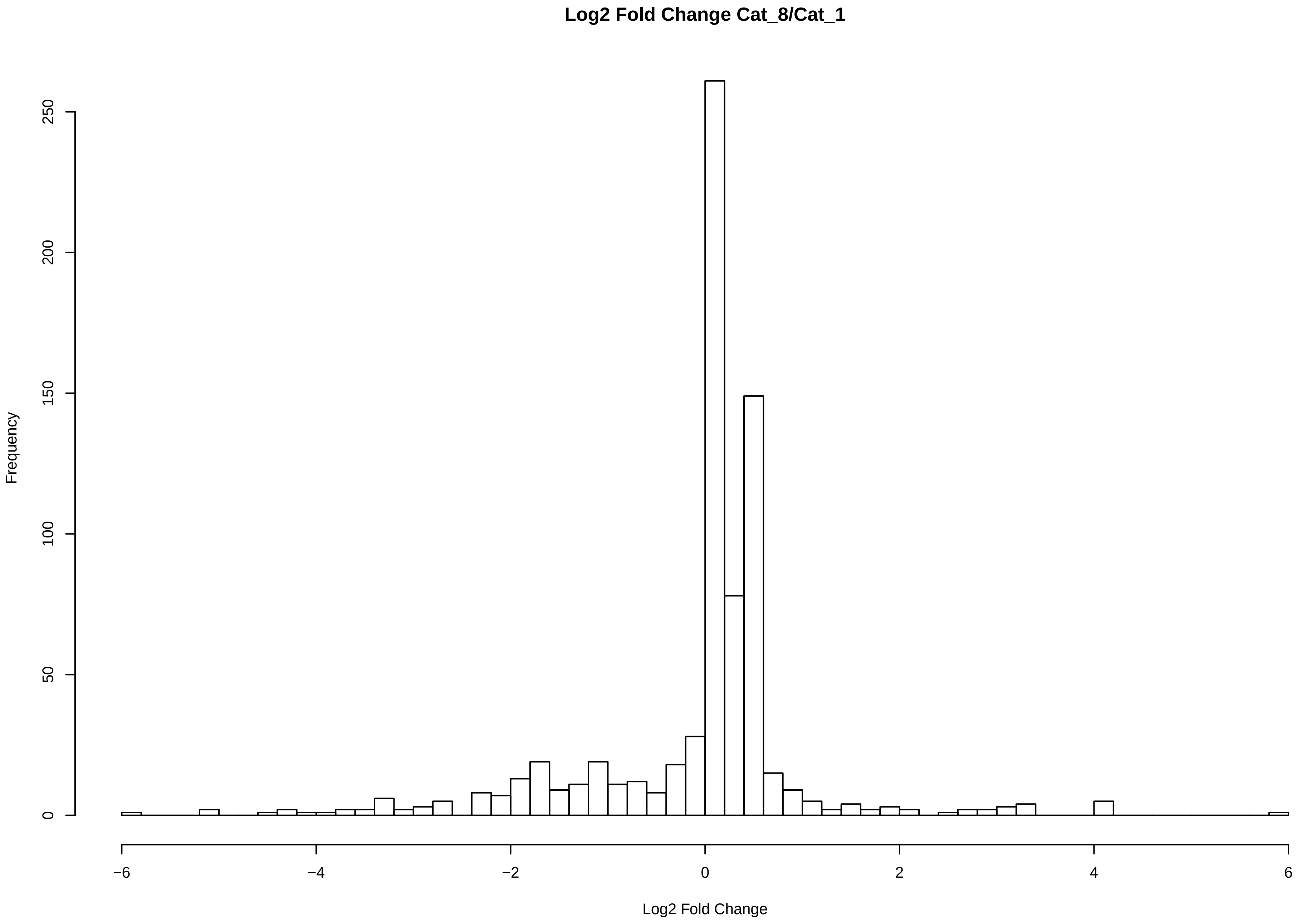
Log2 Fold Change Cat_8/Cat_1
Frequency
0
50
100
150
200
250
−6
−4
−2
0
2
4
6
Log2 Fold Change

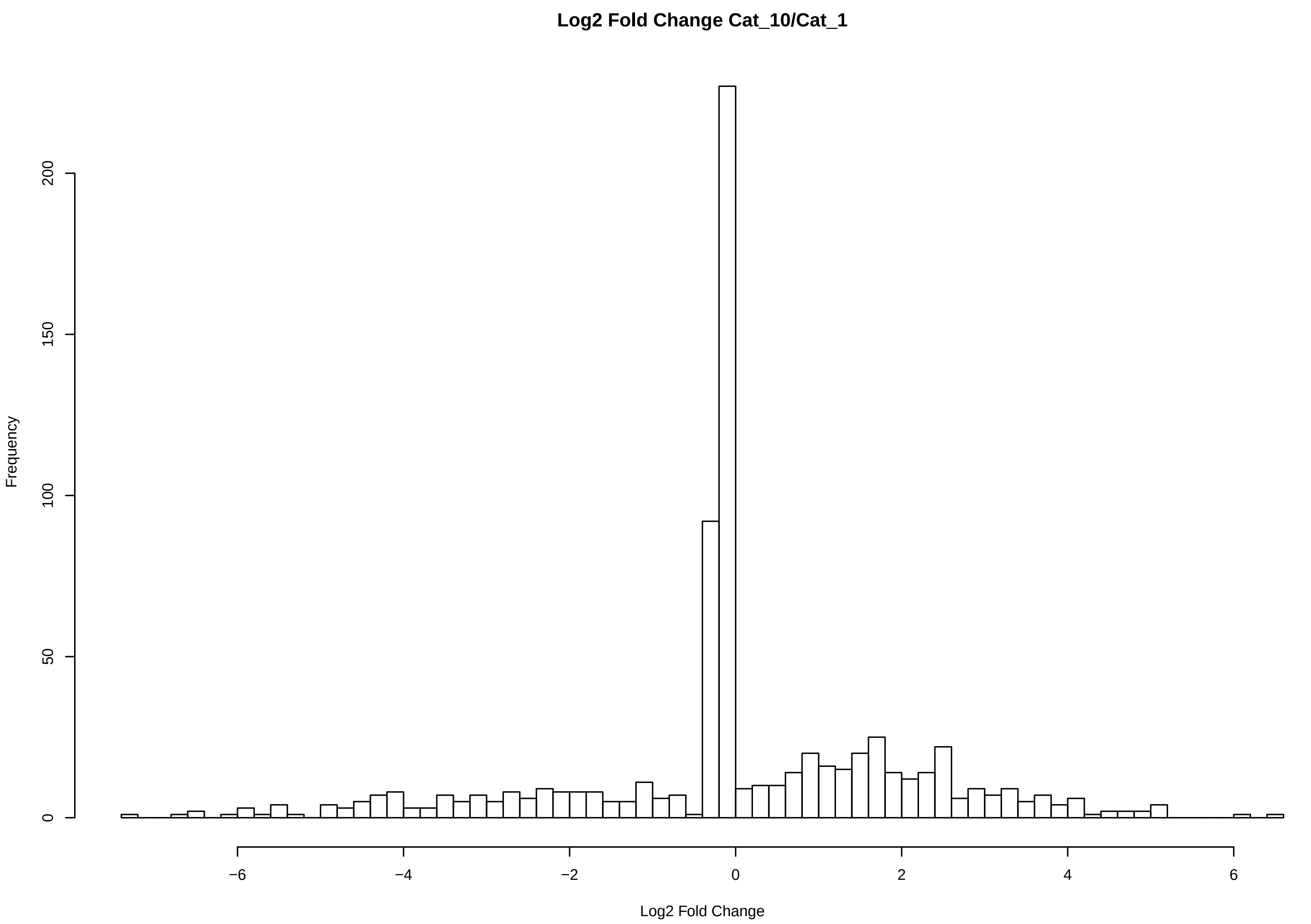
Log2 Fold Change Cat_10/Cat_1
Frequency
0
50
100
150
200
−6
−4
−2
0
2
4
6
Log2 Fold Change

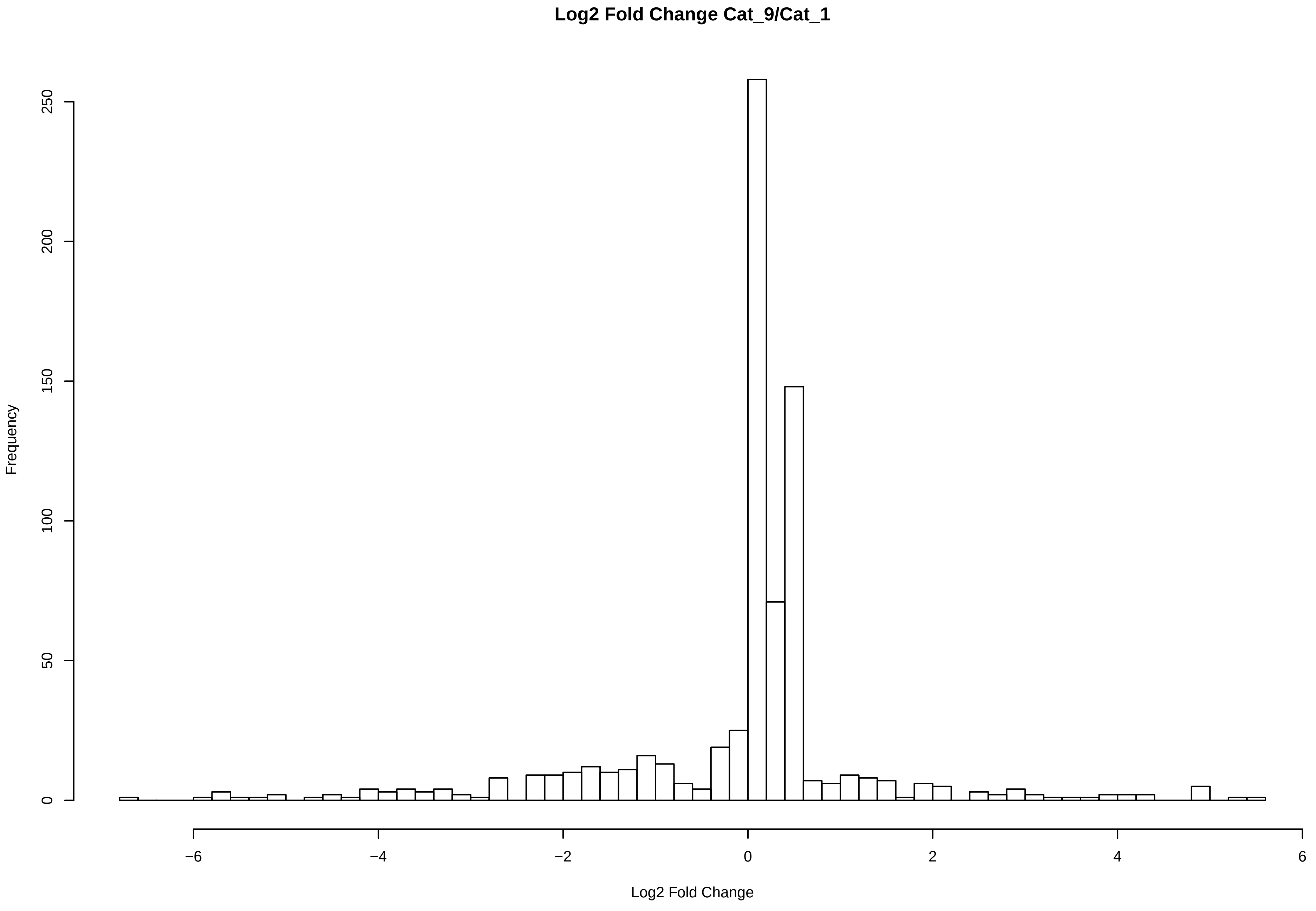
Log2 Fold Change Cat_9/Cat_1
Frequency
0
50
100
150
200
250
−6
−4
−2
0
2
4
6
Log2 Fold Change

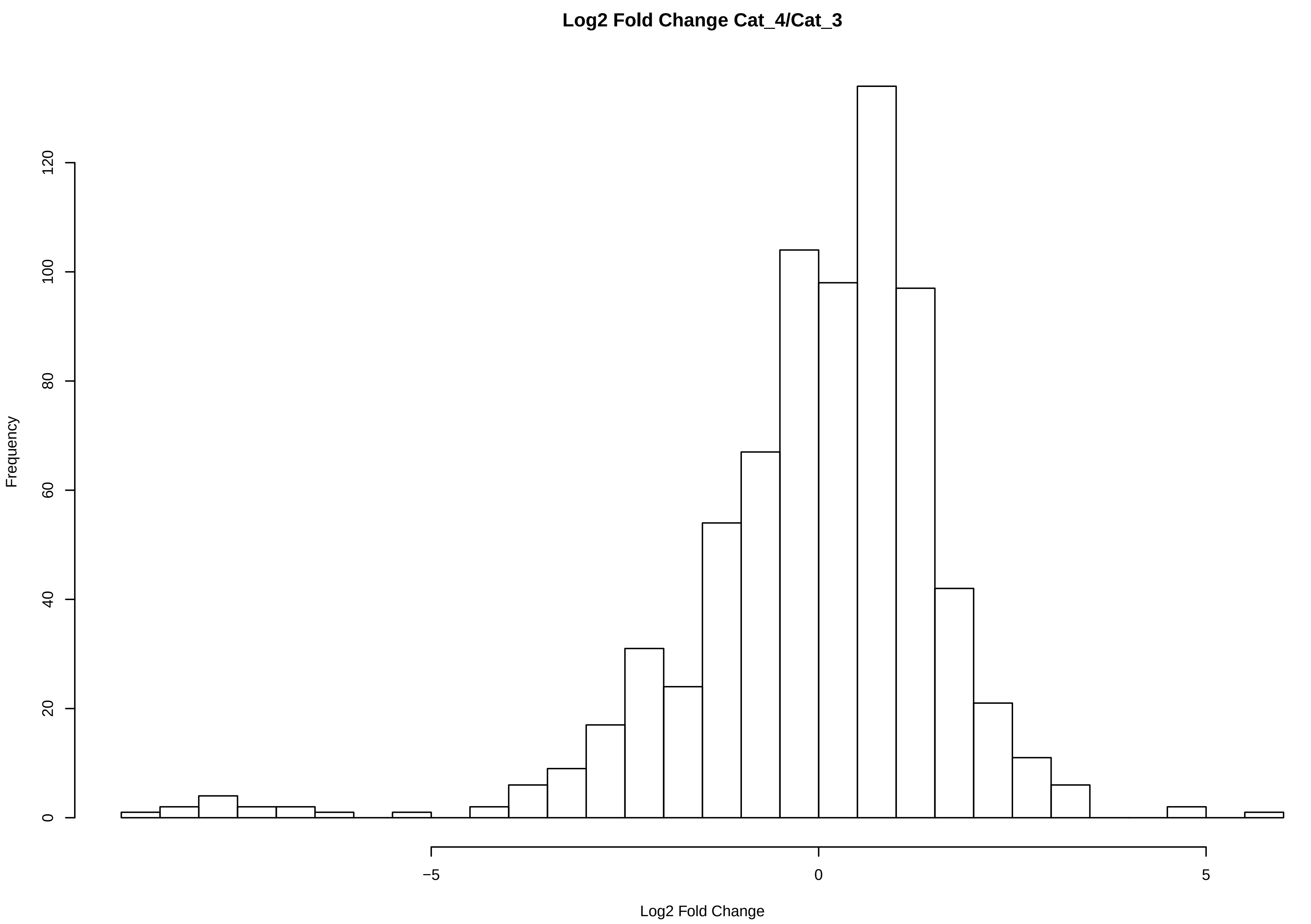

Log2 Fold Change Cat_4/Cat_3
Frequency
0
20
40
60
80
100
120
−5
0
5
Log2 Fold Change

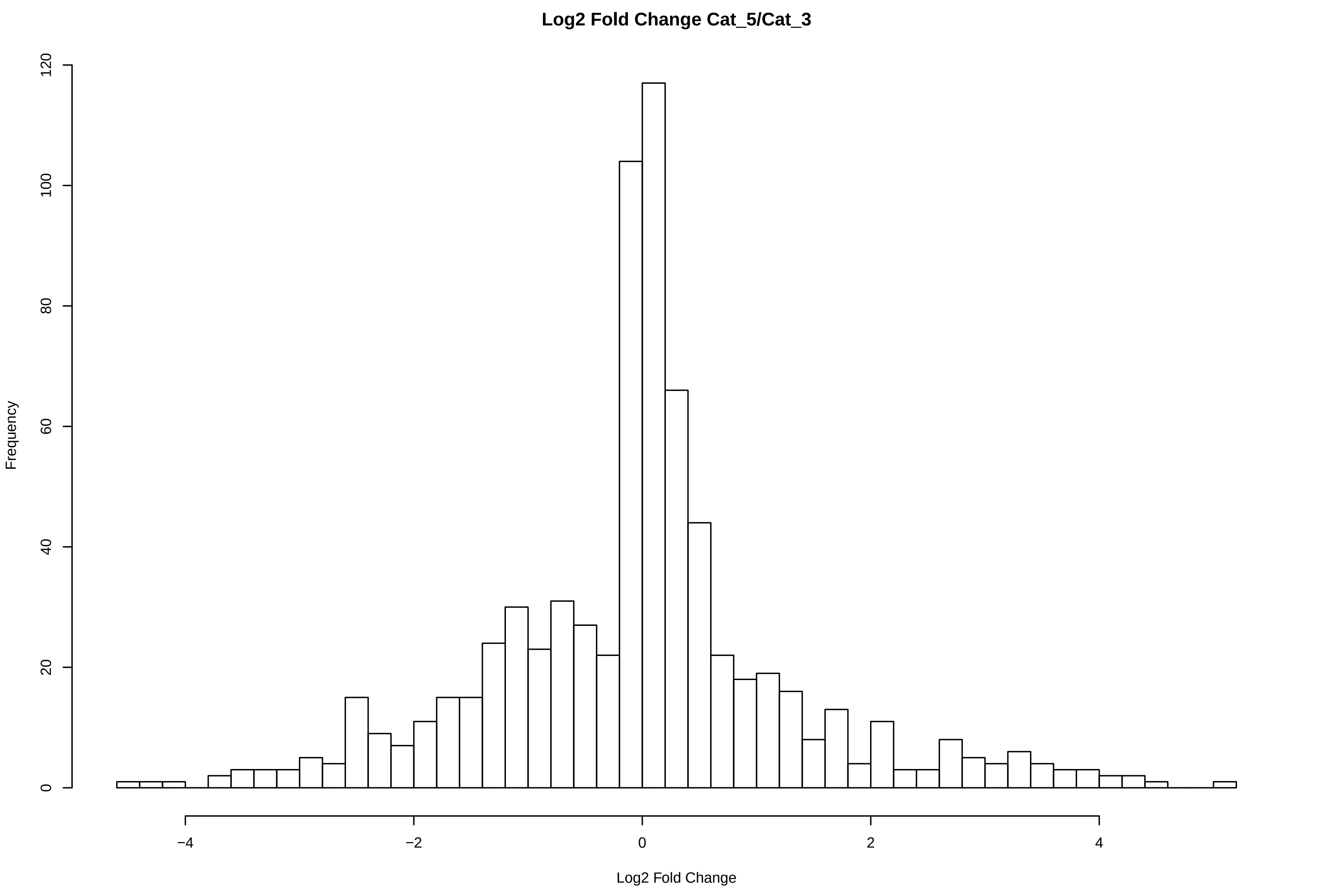
Log2 Fold Change Cat_5/Cat_3
Frequency
0
20
40
60
80
100
120
−4
−2
0
2
4
Log2 Fold Change

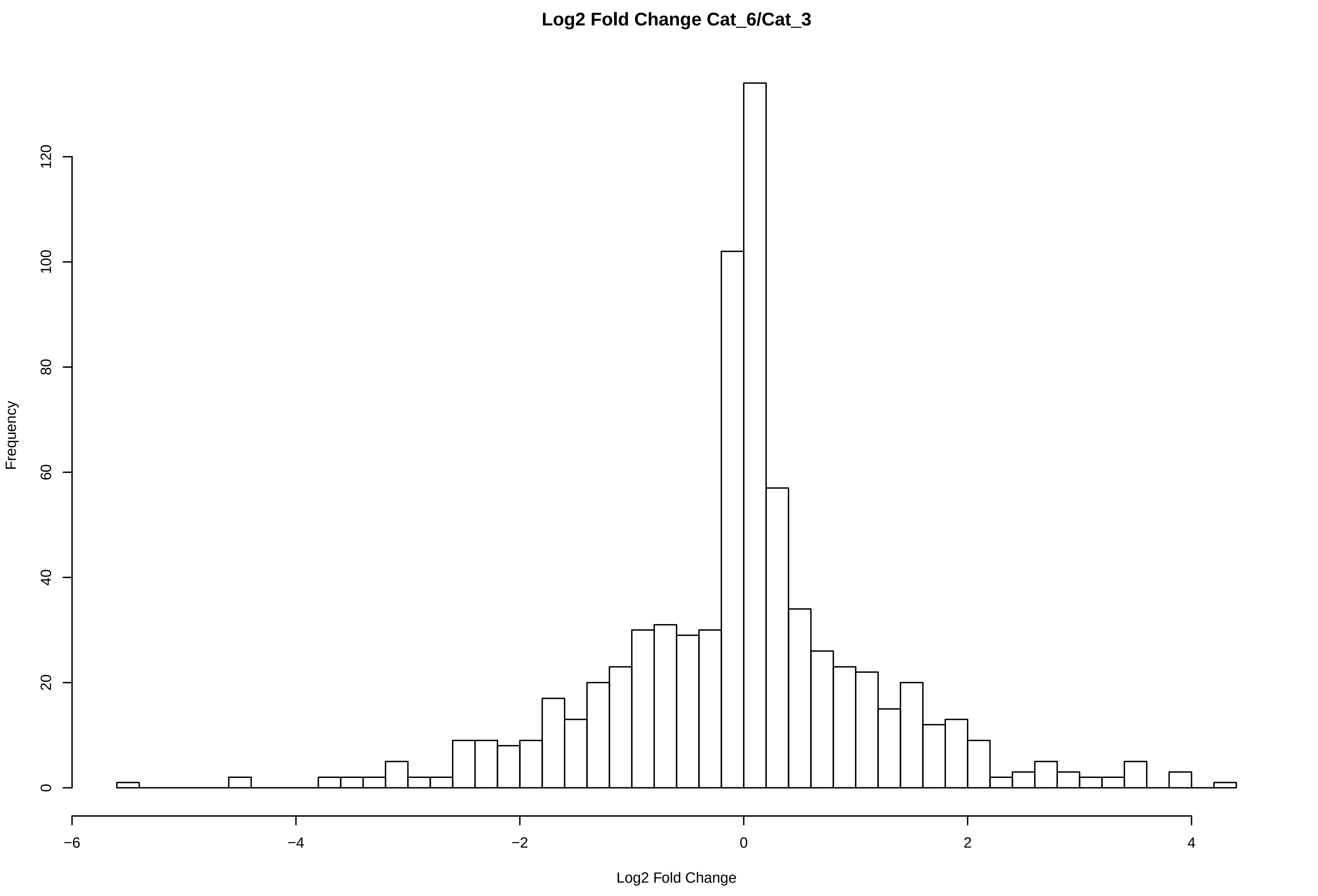

Log2 Fold Change Cat_6/Cat_3
Frequency
0
20
40
60
80
100
120
−6
−4
−2
0
2
4
Log2 Fold Change

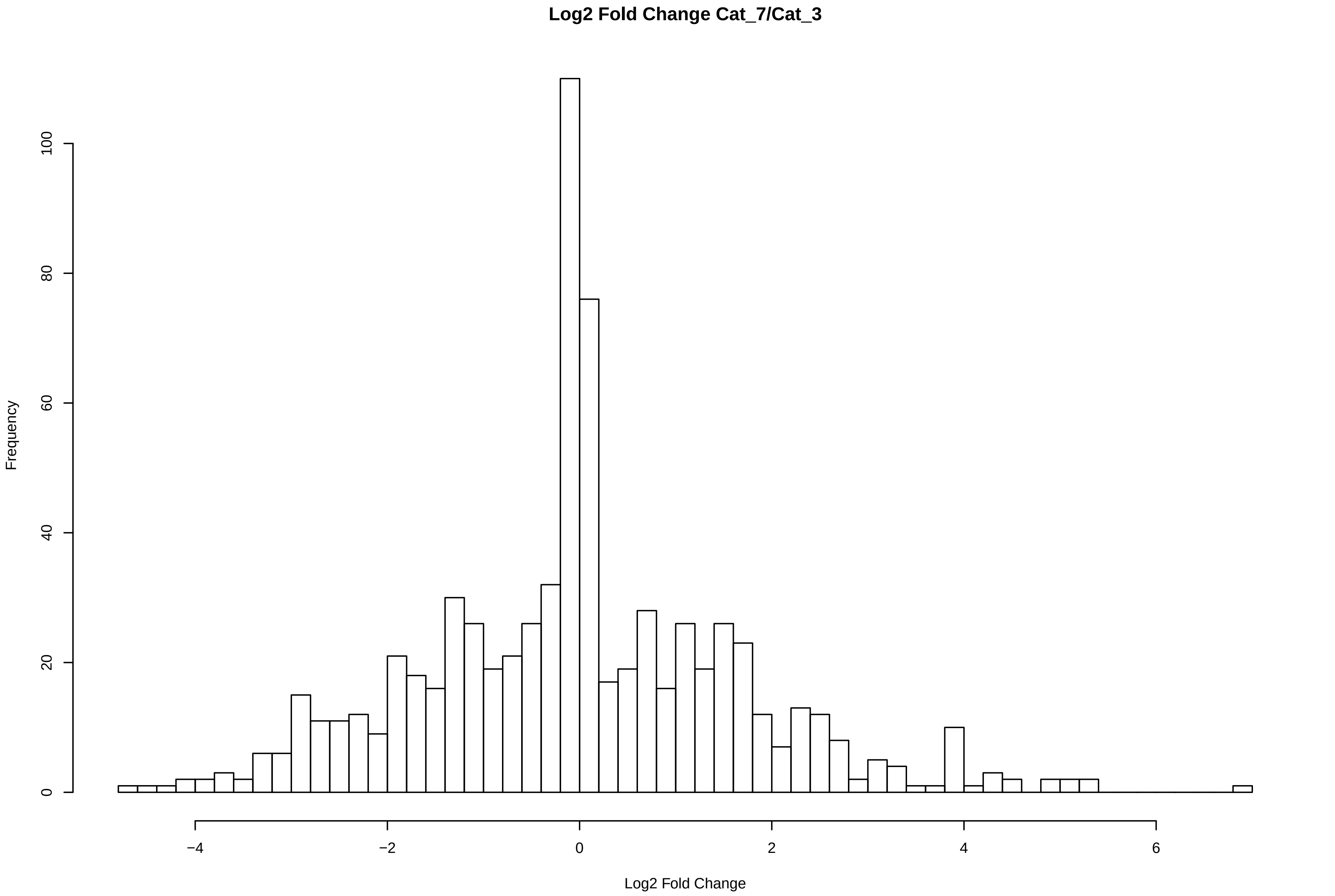

Log2 Fold Change Cat_7/Cat_3
Frequency
0
20
40
60
80
100
−4
−2
0
2
4
6
Log2 Fold Change

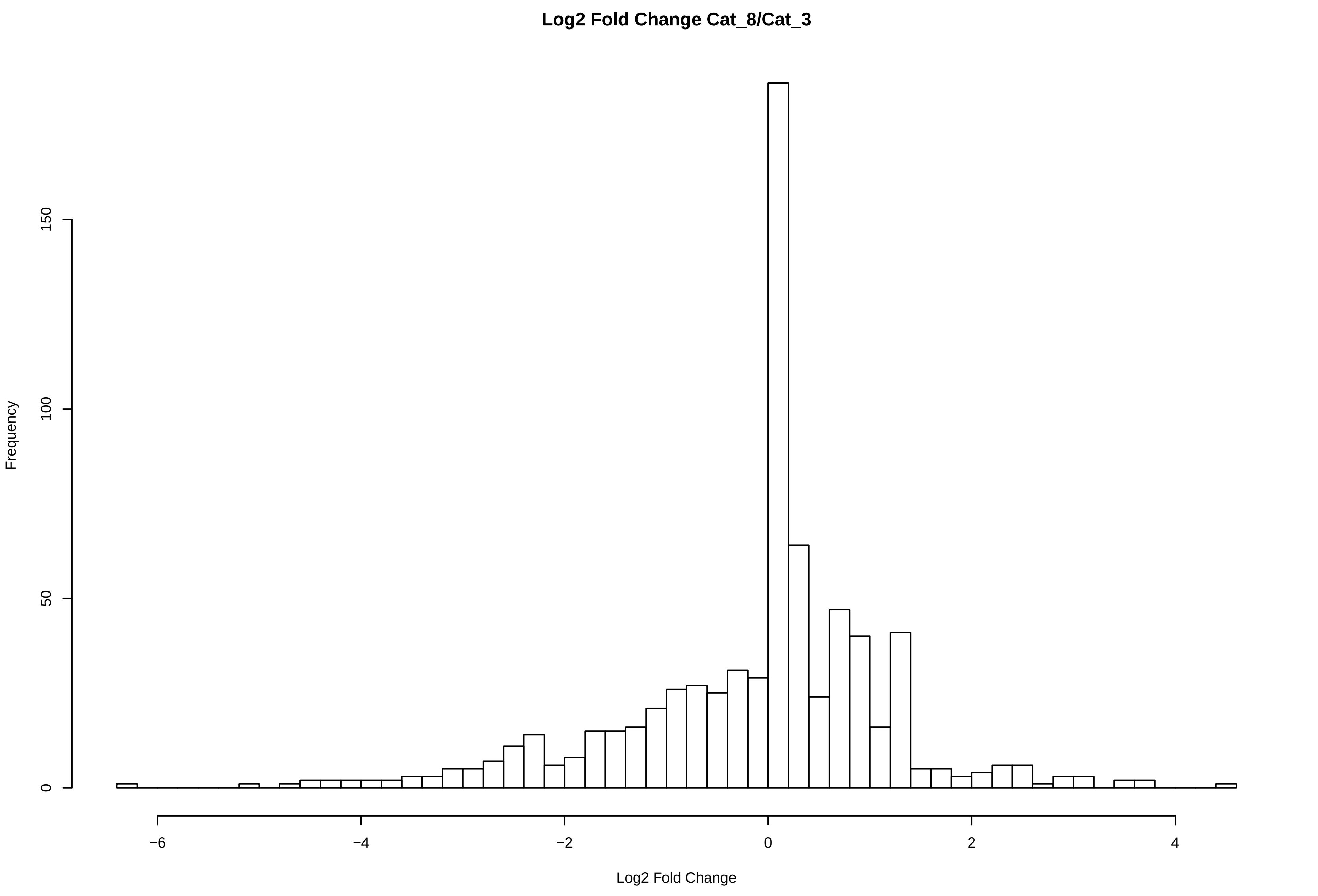

Log2 Fold Change Cat_8/Cat_3
Frequency
0
50
100
150
−6
−4
−2
0
2
4
Log2 Fold Change

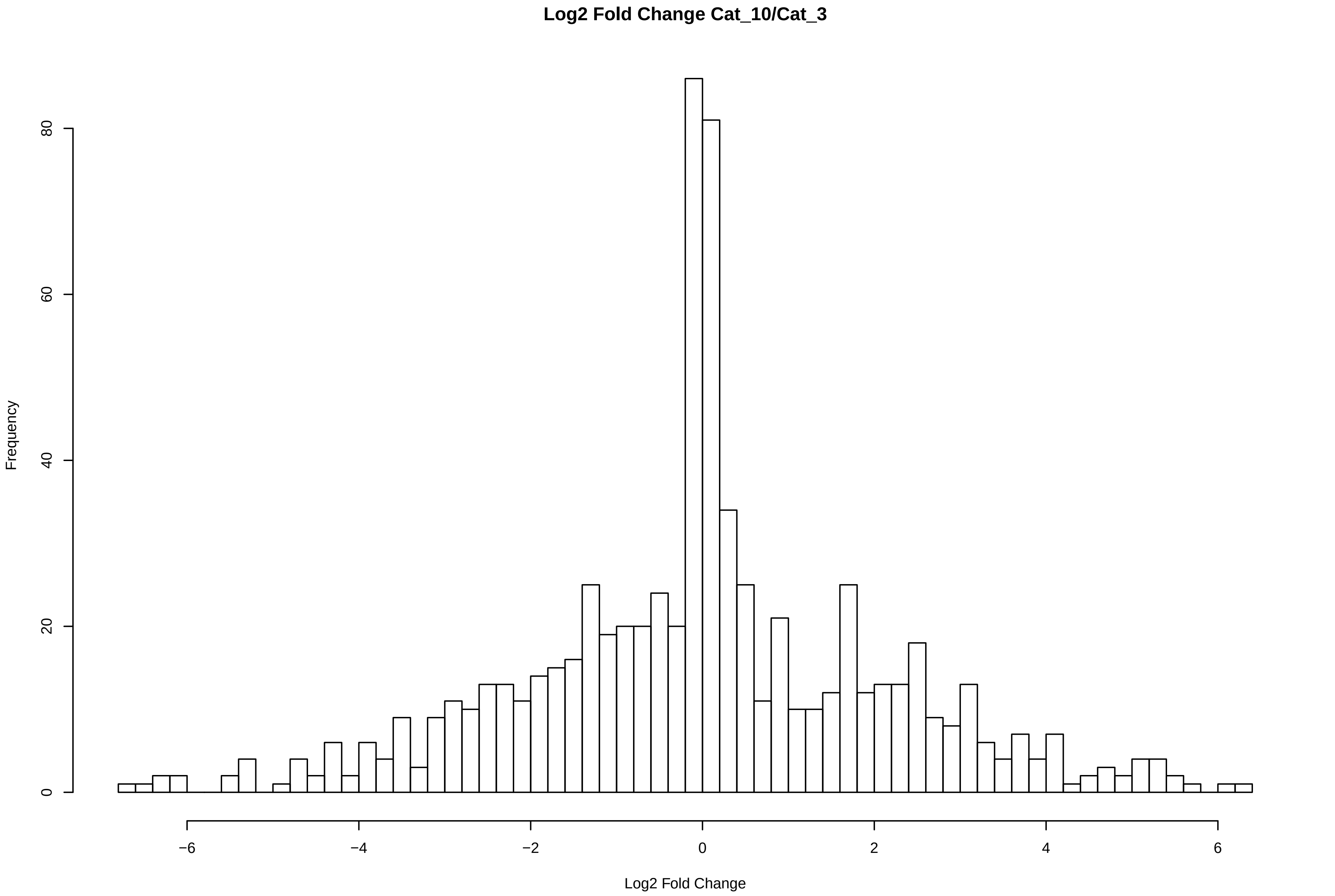
Log2 Fold Change Cat_10/Cat_3
Frequency
0
20
40
60
80
−6
−4
−2
0
2
4
6
Log2 Fold Change

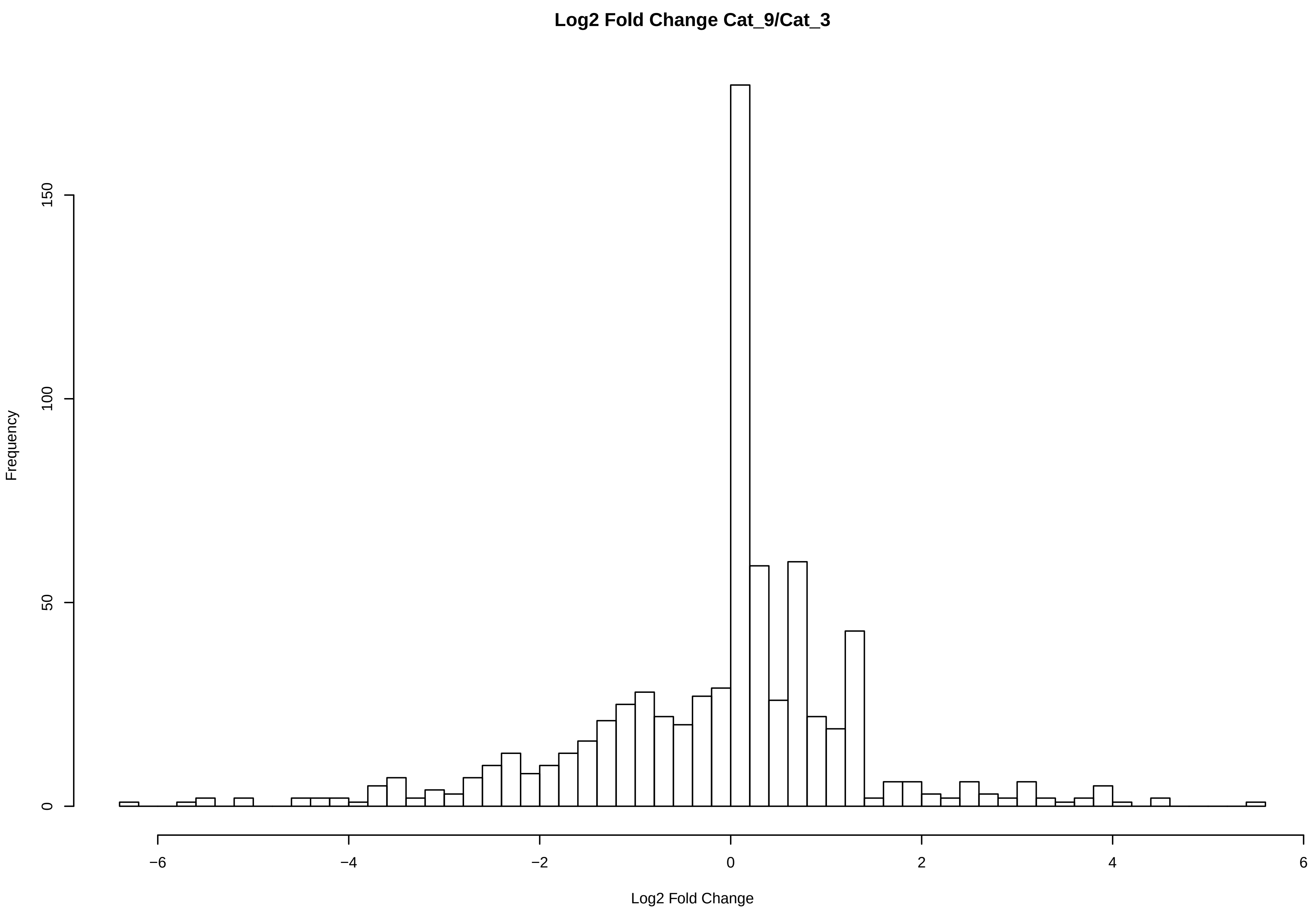

Log2 Fold Change Cat_9/Cat_3
Frequency
0
50
100
150
−6
−4
−2
0
2
4
6
Log2 Fold Change

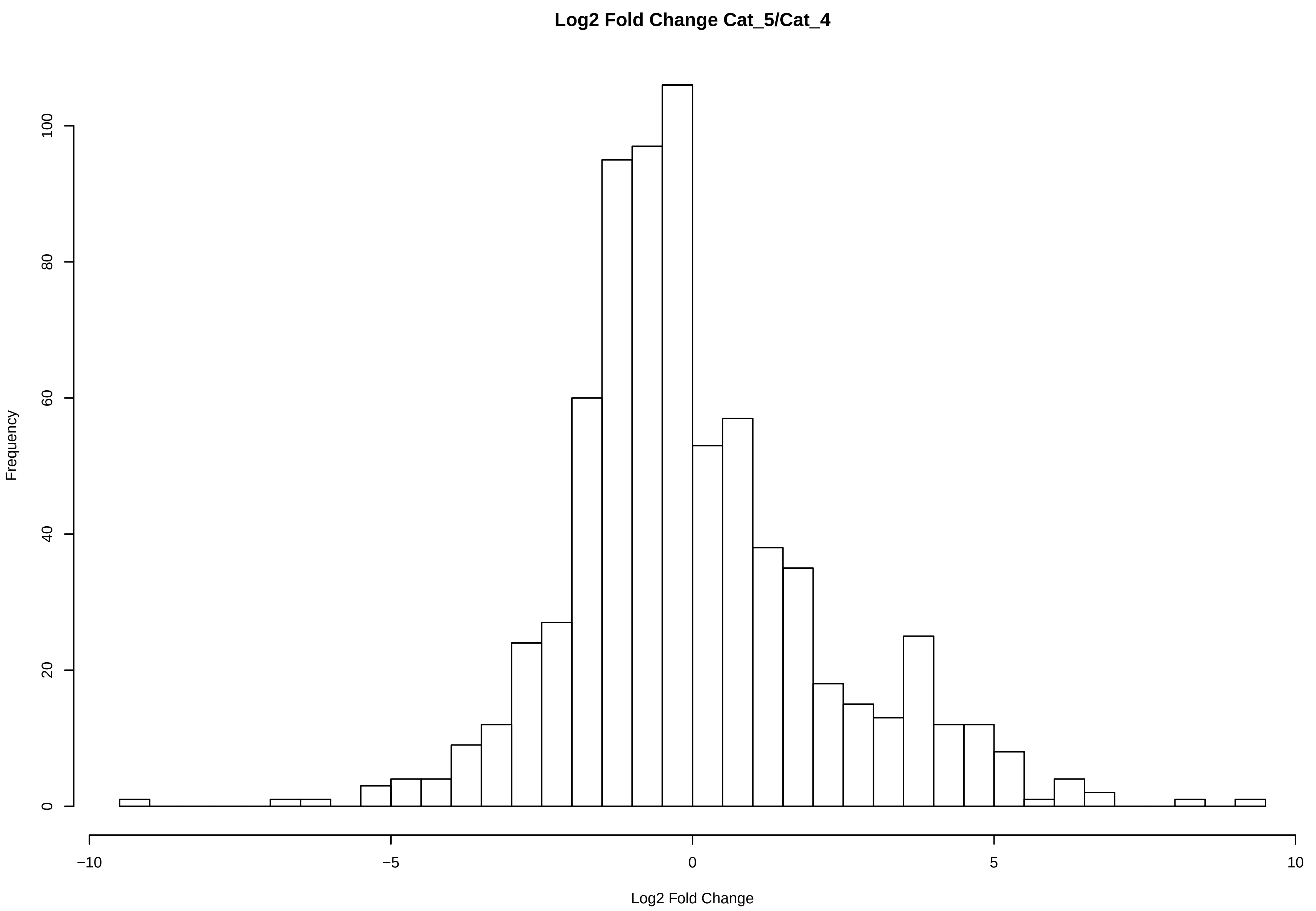

Log2 Fold Change Cat_5/Cat_4
Frequency
0
20
40
60
80
100
−10
−5
0
5
10
Log2 Fold Change

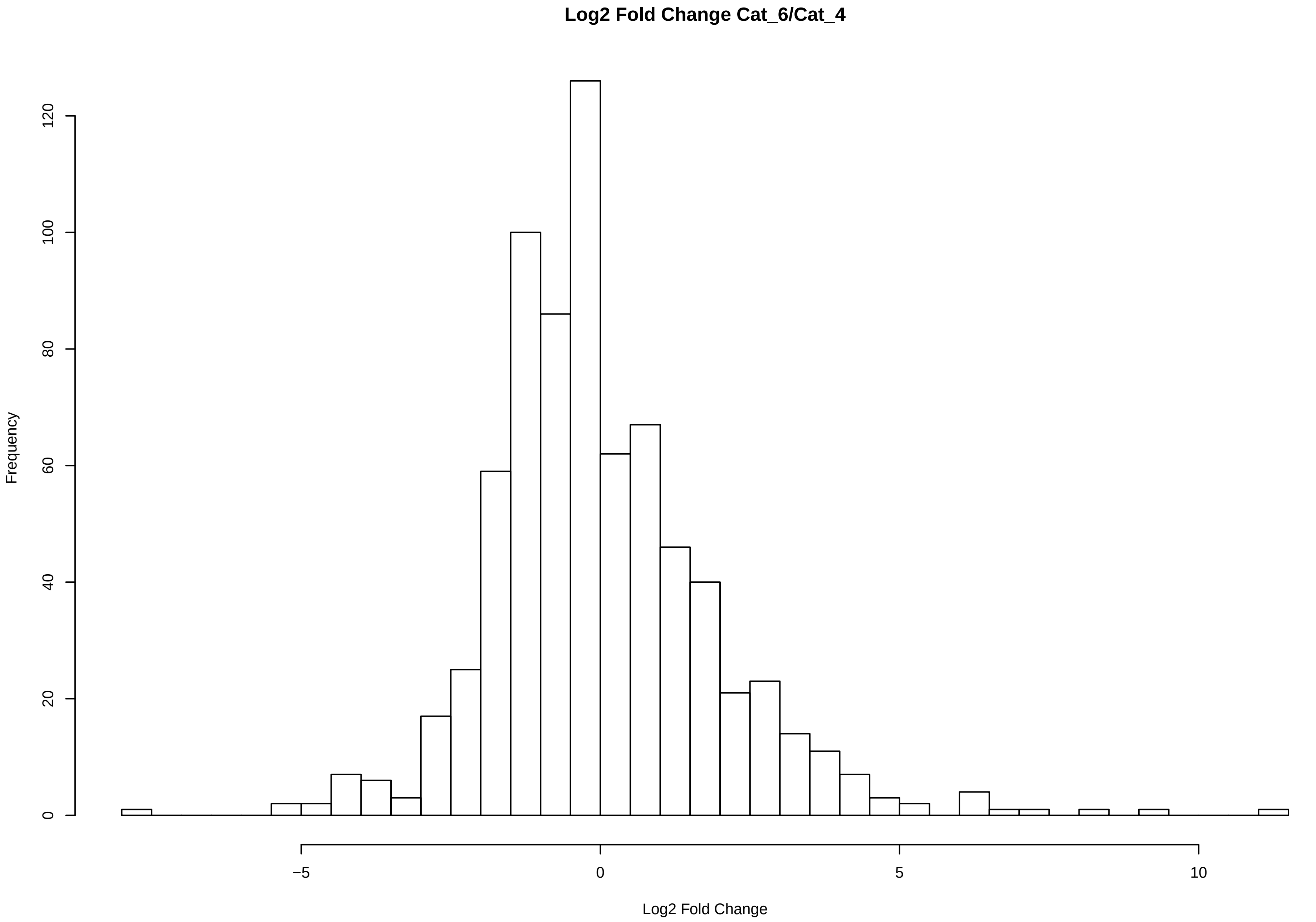
Log2 Fold Change Cat_6/Cat_4
Frequency
0
20
40
60
80
100
120
−5
0
5
10
Log2 Fold Change

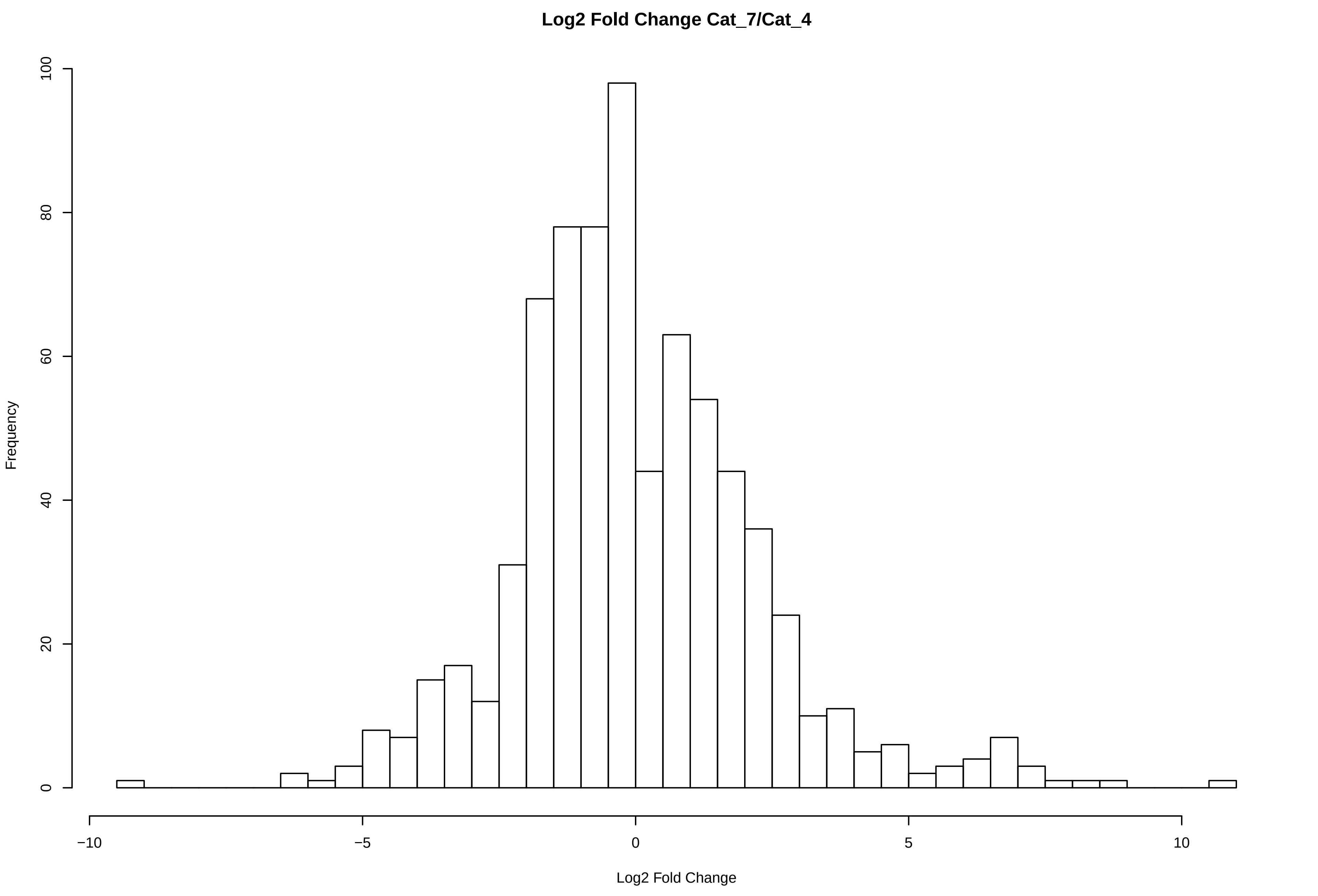
Log2 Fold Change Cat_7/Cat_4
Frequency
0
20
40
60
80
100
−10
−5
0
5
10
Log2 Fold Change

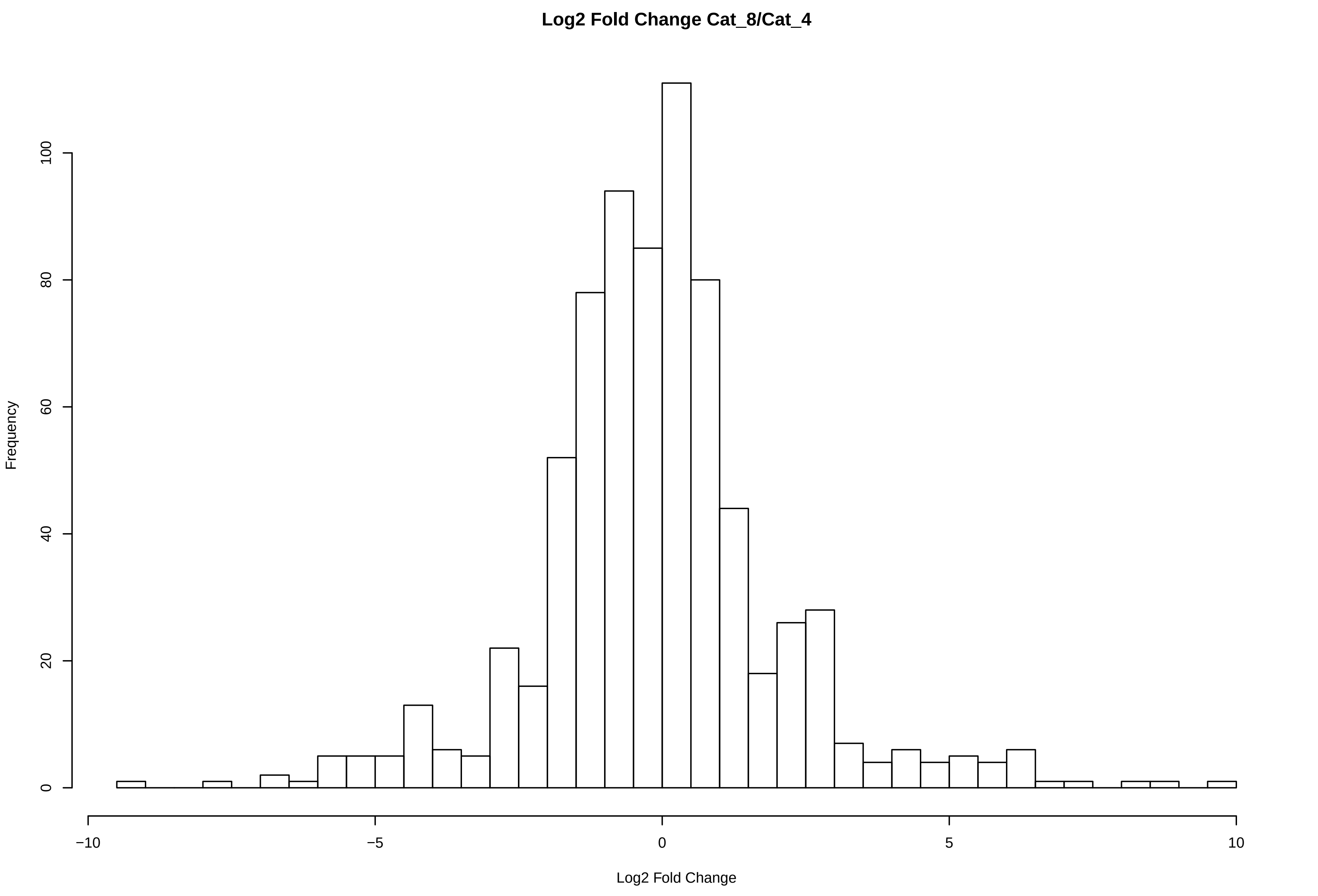
Log2 Fold Change Cat_8/Cat_4
Frequency
0
20
40
60
80
100
−10
−5
0
5
10
Log2 Fold Change

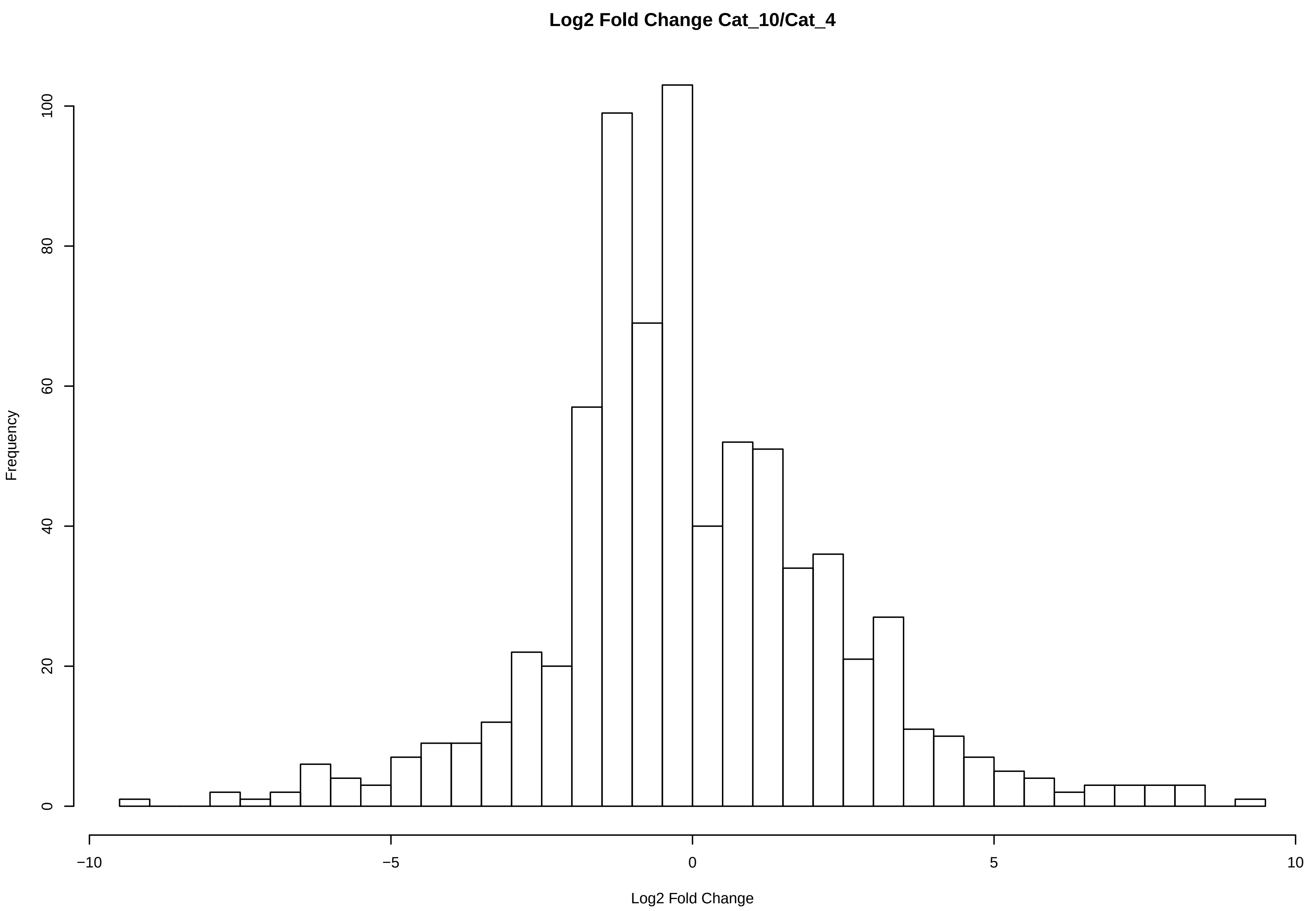

Log2 Fold Change Cat_10/Cat_4
Frequency
0
20
40
60
80
100
−10
−5
0
5
10
Log2 Fold Change

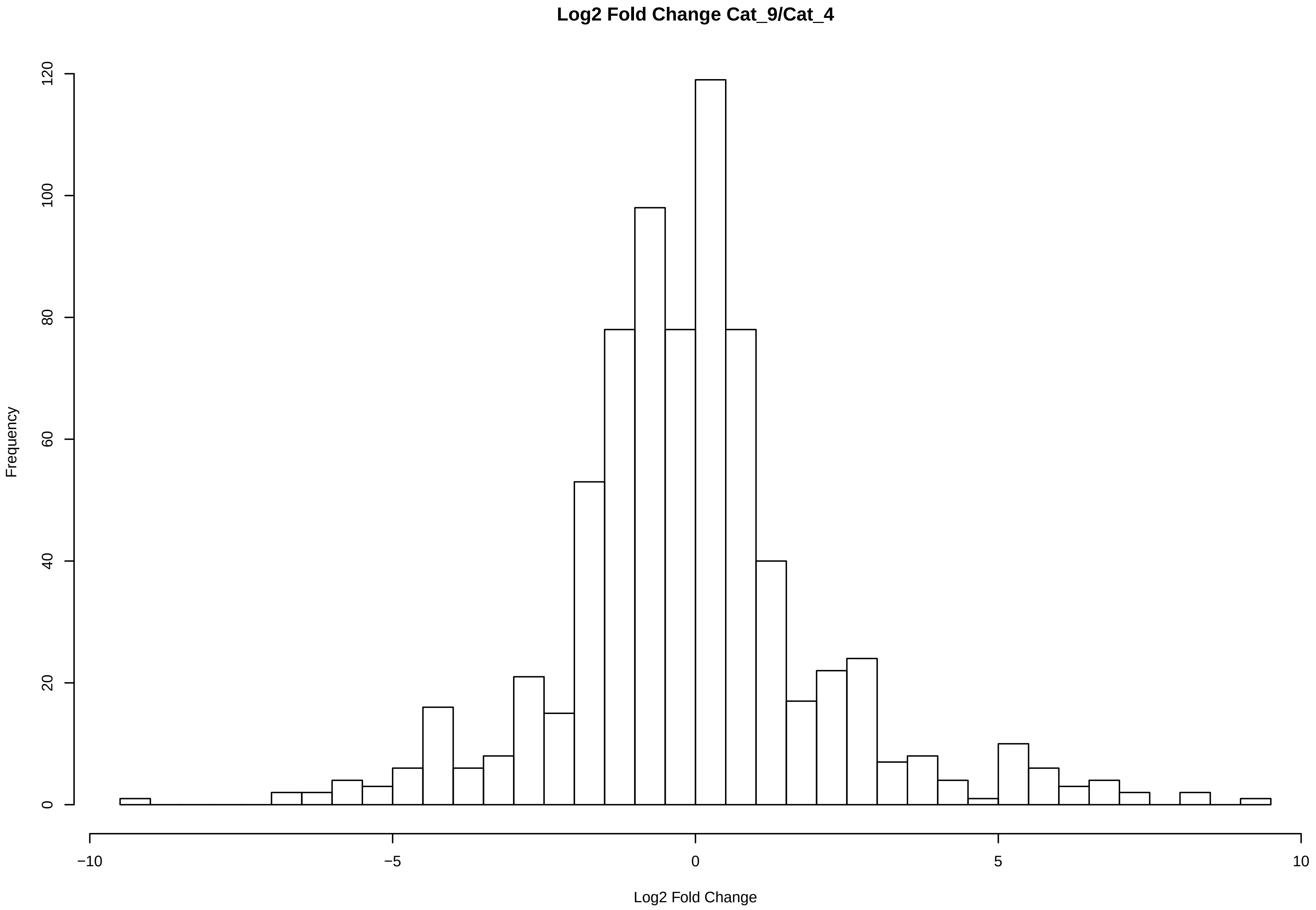
Log2 Fold Change Cat_9/Cat_4
Frequency
0
20
40
60
80
100
120
−10
−5
0
5
10
Log2 Fold Change

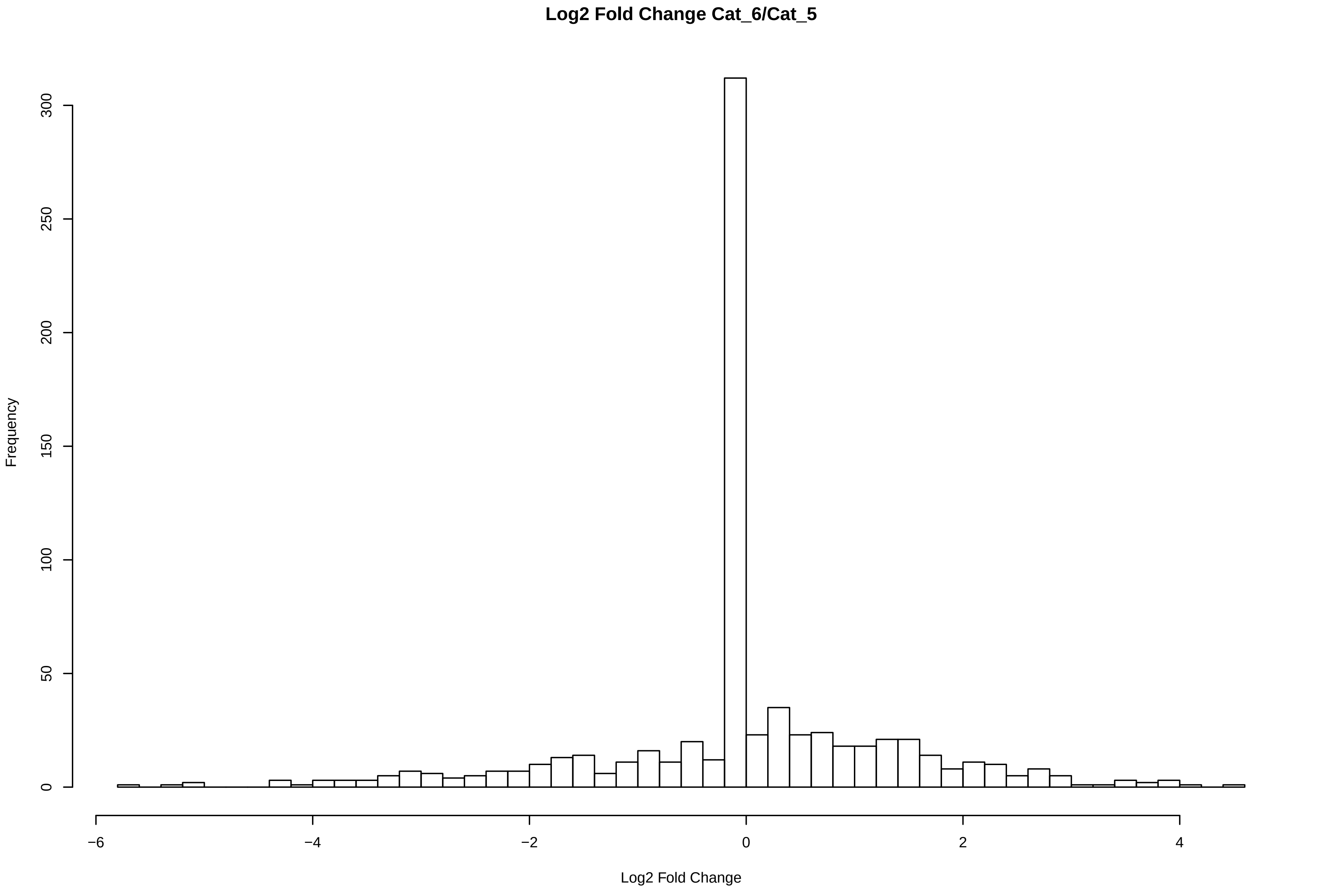
Log2 Fold Change Cat_6/Cat_5
Frequency
0
50
100
150
200
250
300
−6
−4
−2
0
2
4
Log2 Fold Change

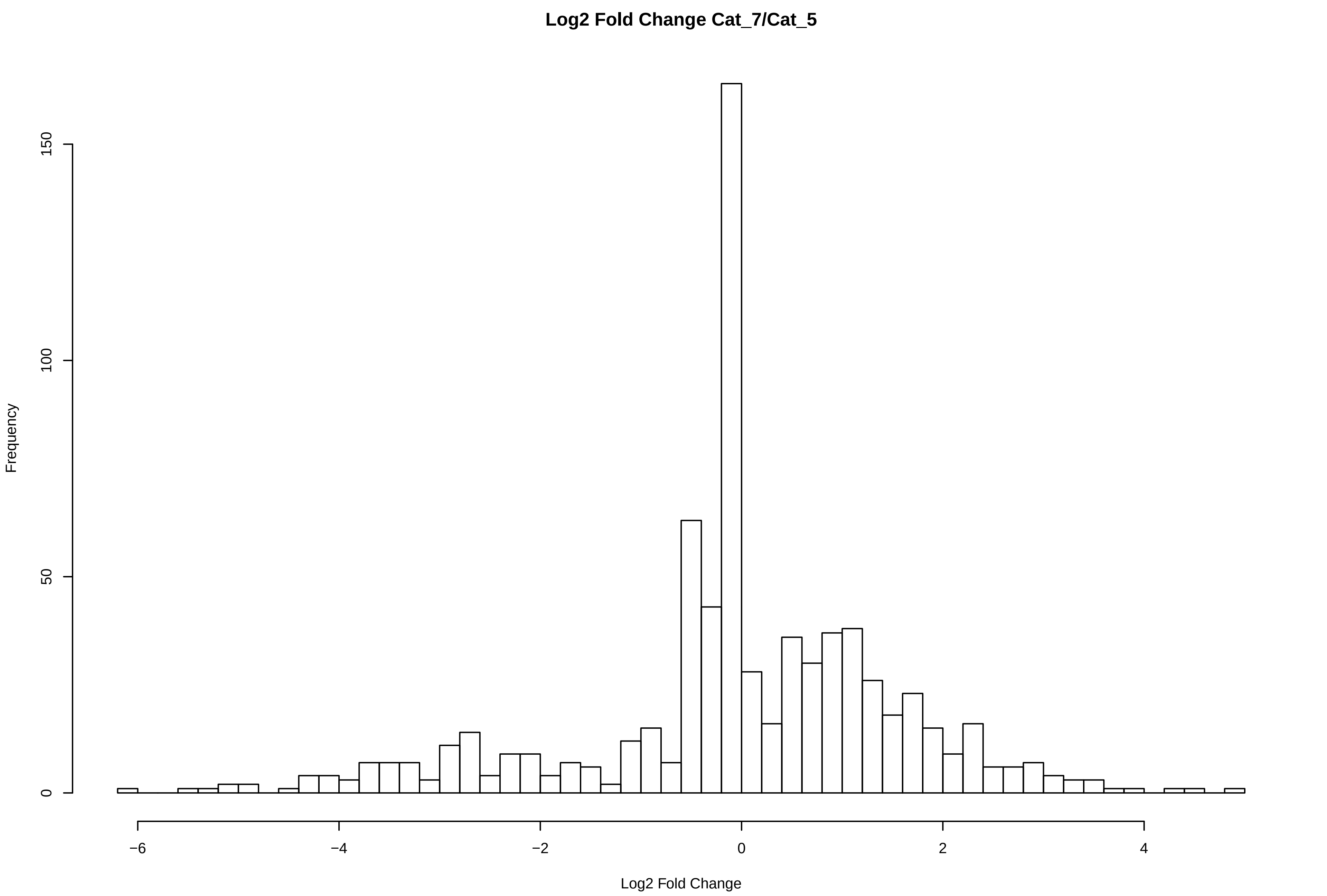

Log2 Fold Change Cat_7/Cat_5
Frequency
0
50
100
150
−6
−4
−2
0
2
4
Log2 Fold Change

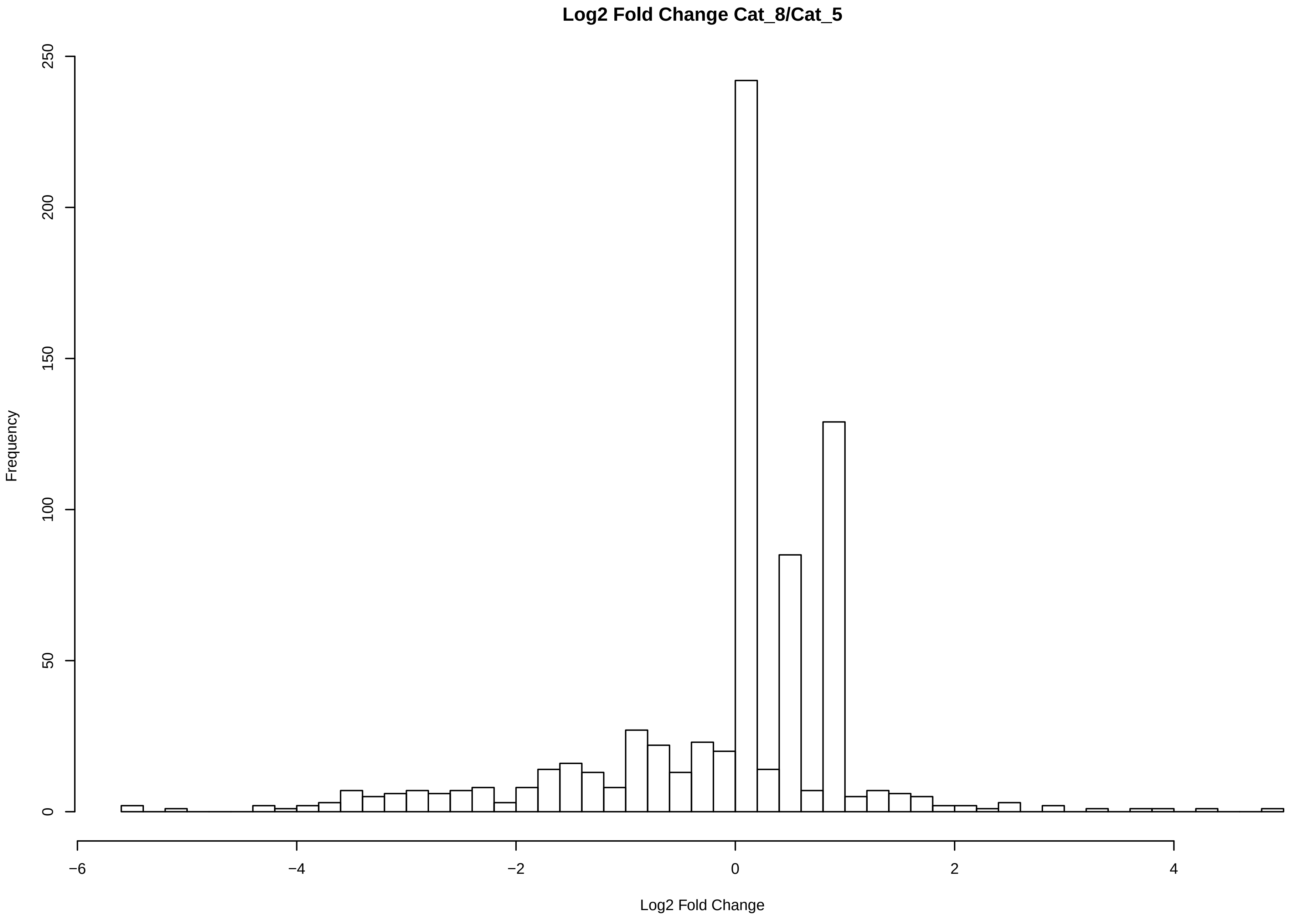

Log2 Fold Change Cat_8/Cat_5
Frequency
0
50
100
150
200
250
−6
−4
−2
0
2
4
Log2 Fold Change

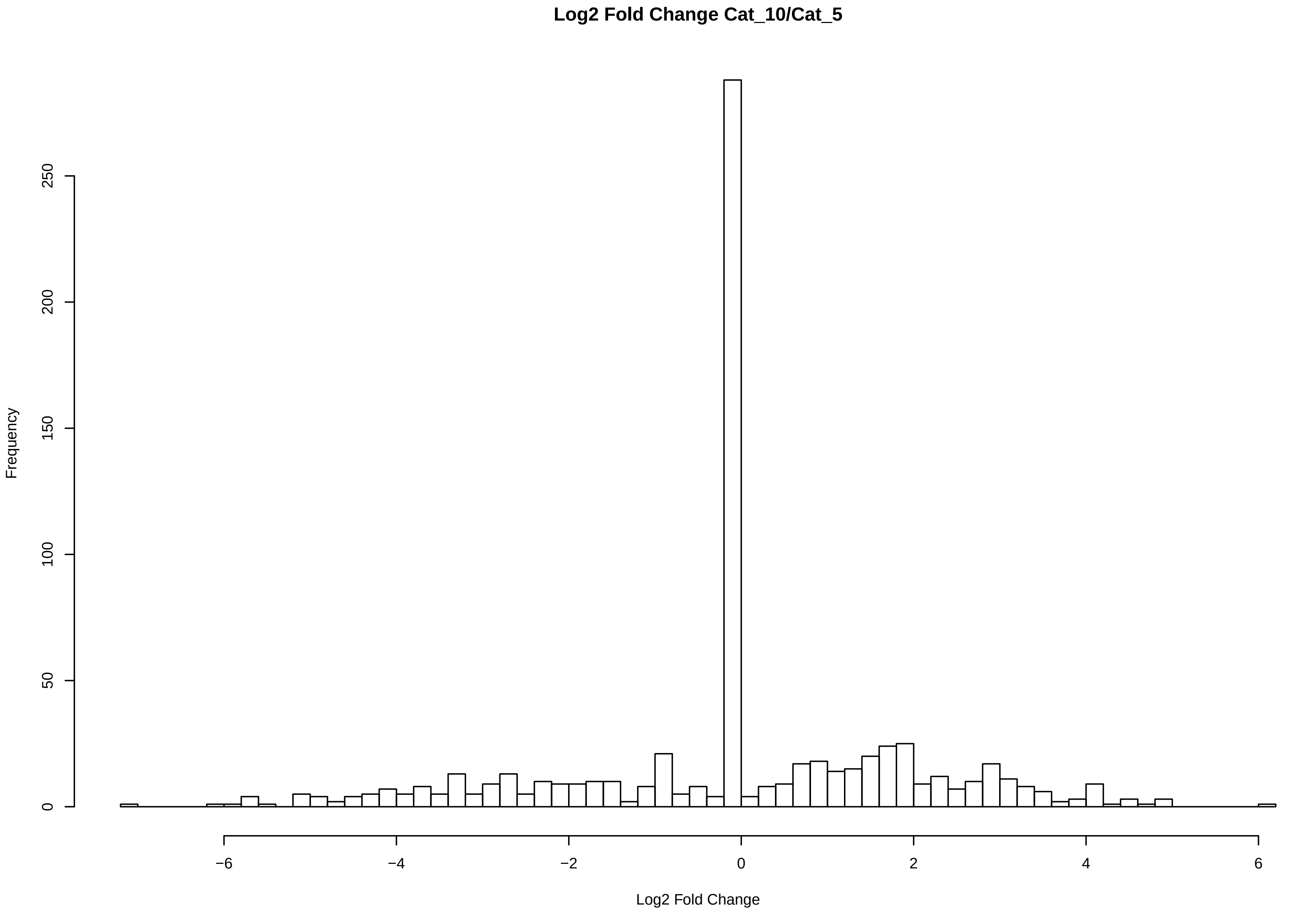
Log2 Fold Change Cat_10/Cat_5
Frequency
0
50
100
150
200
250
−6
−4
−2
0
2
4
6
Log2 Fold Change

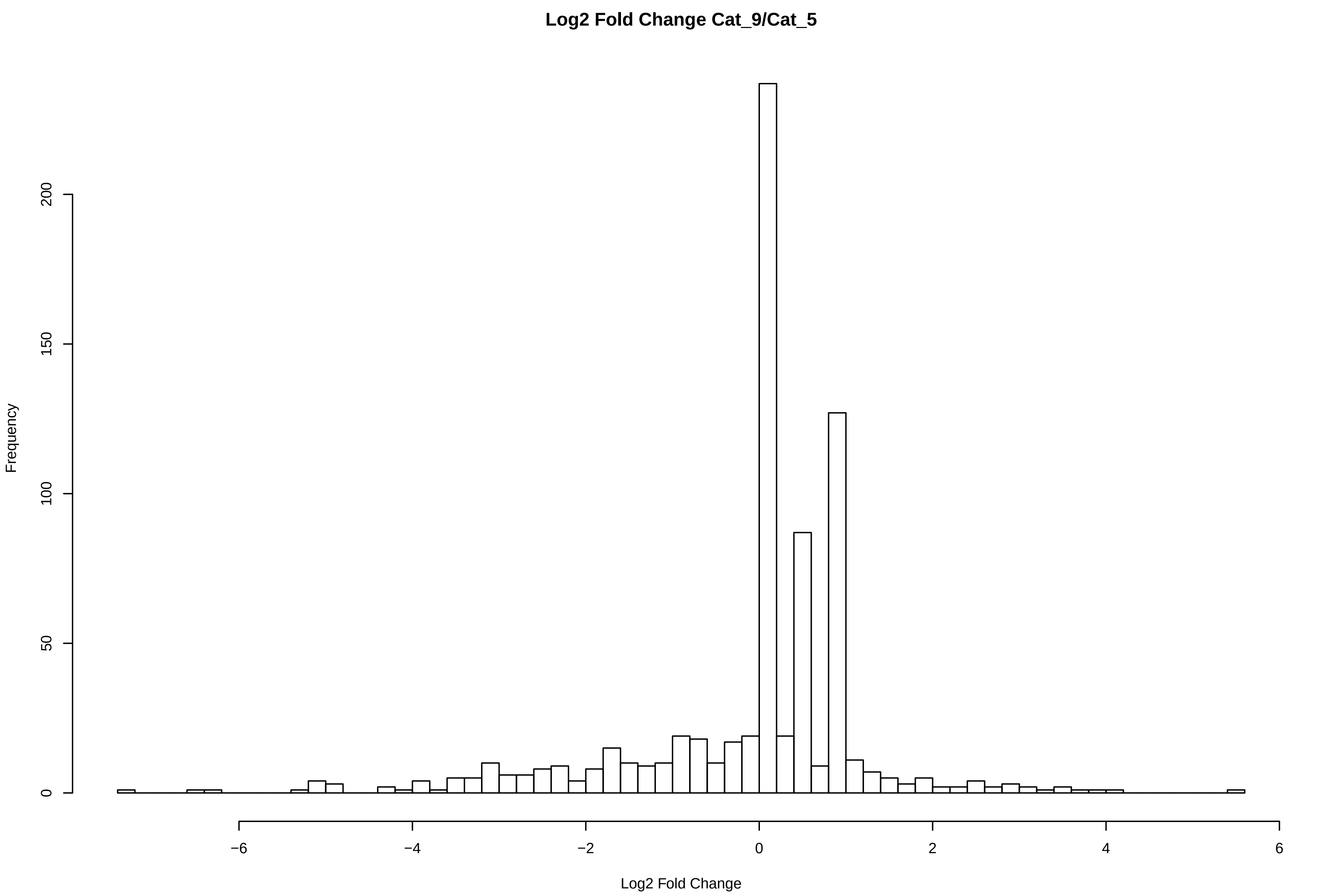

Log2 Fold Change Cat_9/Cat_5
Frequency
0
50
100
150
200
−6
−4
−2
0
2
4
6
Log2 Fold Change

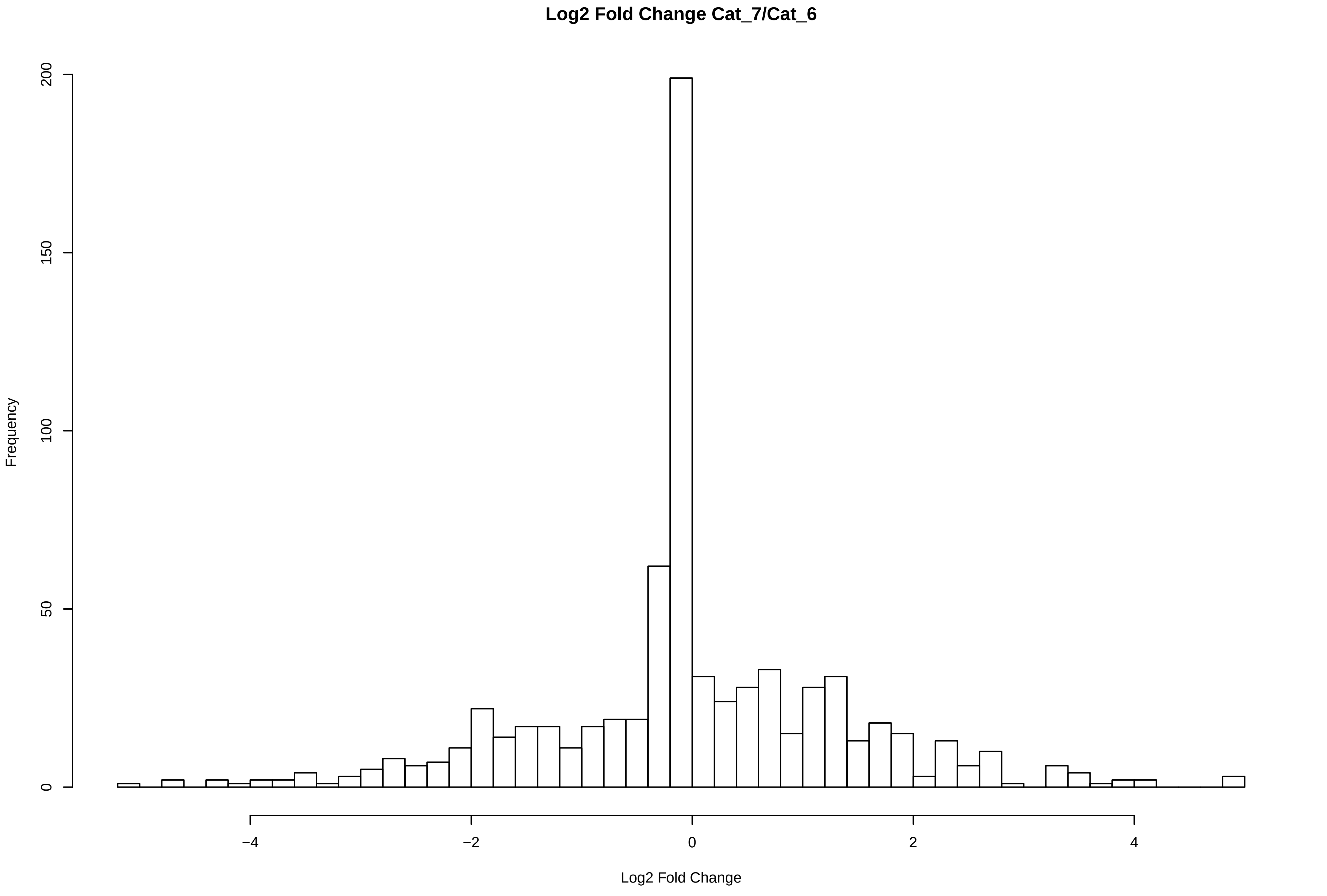

Log2 Fold Change Cat_7/Cat_6
Frequency
0
50
100
150
200
−4
−2
0
2
4
Log2 Fold Change

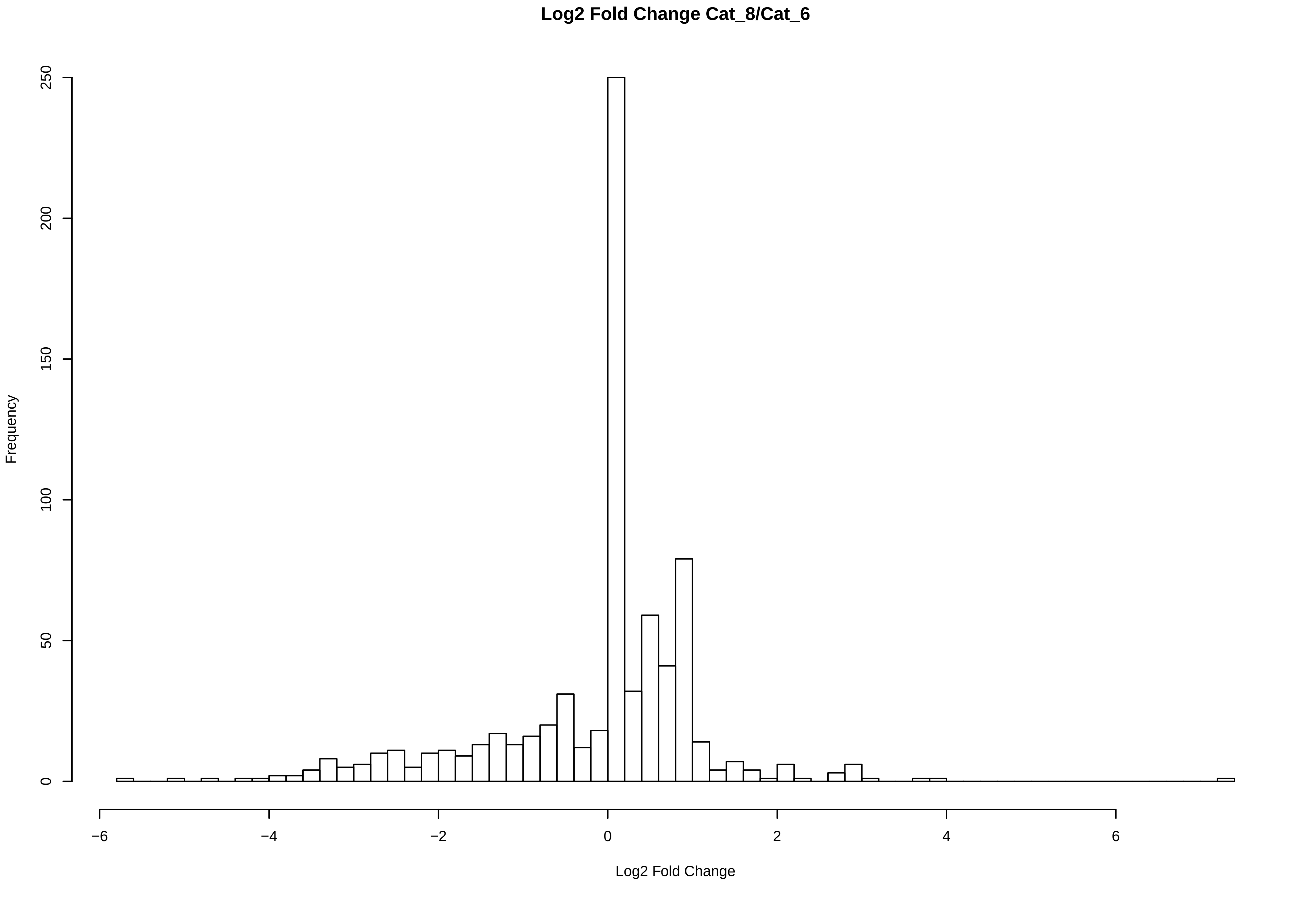
Log2 Fold Change Cat_8/Cat_6
Frequency
0
50
100
150
200
250
−6
−4
−2
0
2
4
6
Log2 Fold Change

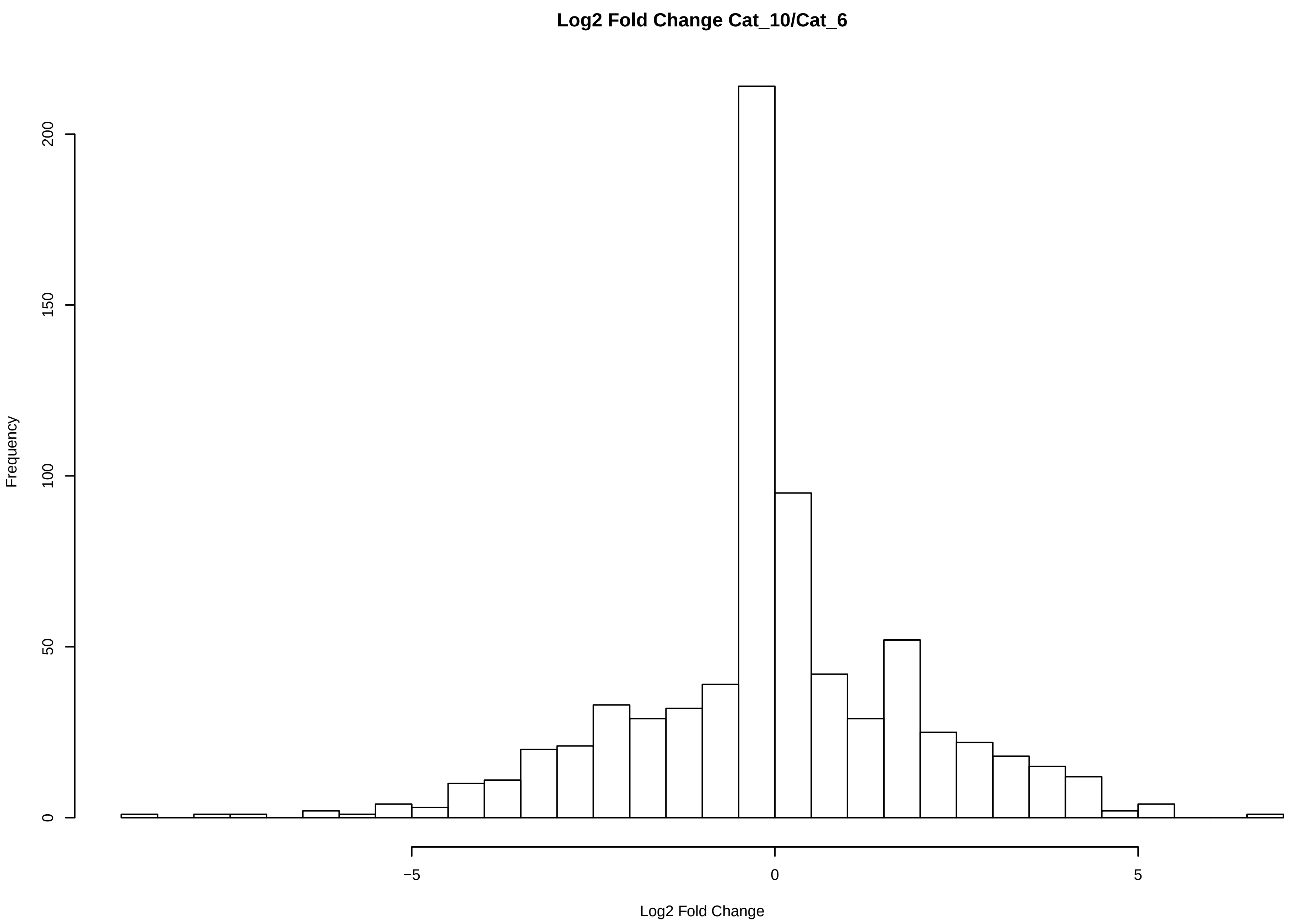

Log2 Fold Change Cat_10/Cat_6
Frequency
0
50
100
150
200
−5
0
5
Log2 Fold Change

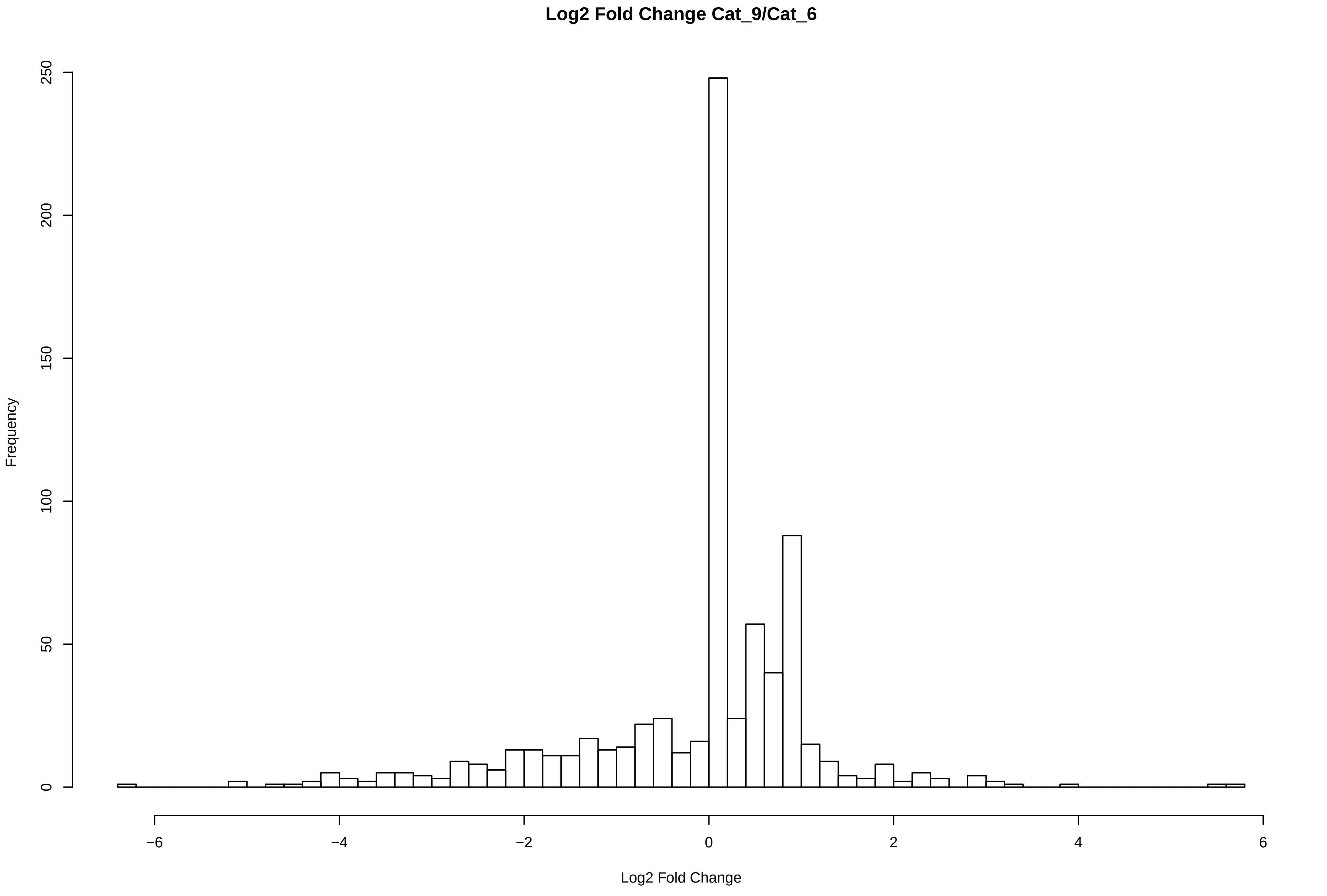

Log2 Fold Change Cat_9/Cat_6
Frequency
Log2 Fold Change
0
50
100
150
200
250
−6
−4
−2
0
2
4
6

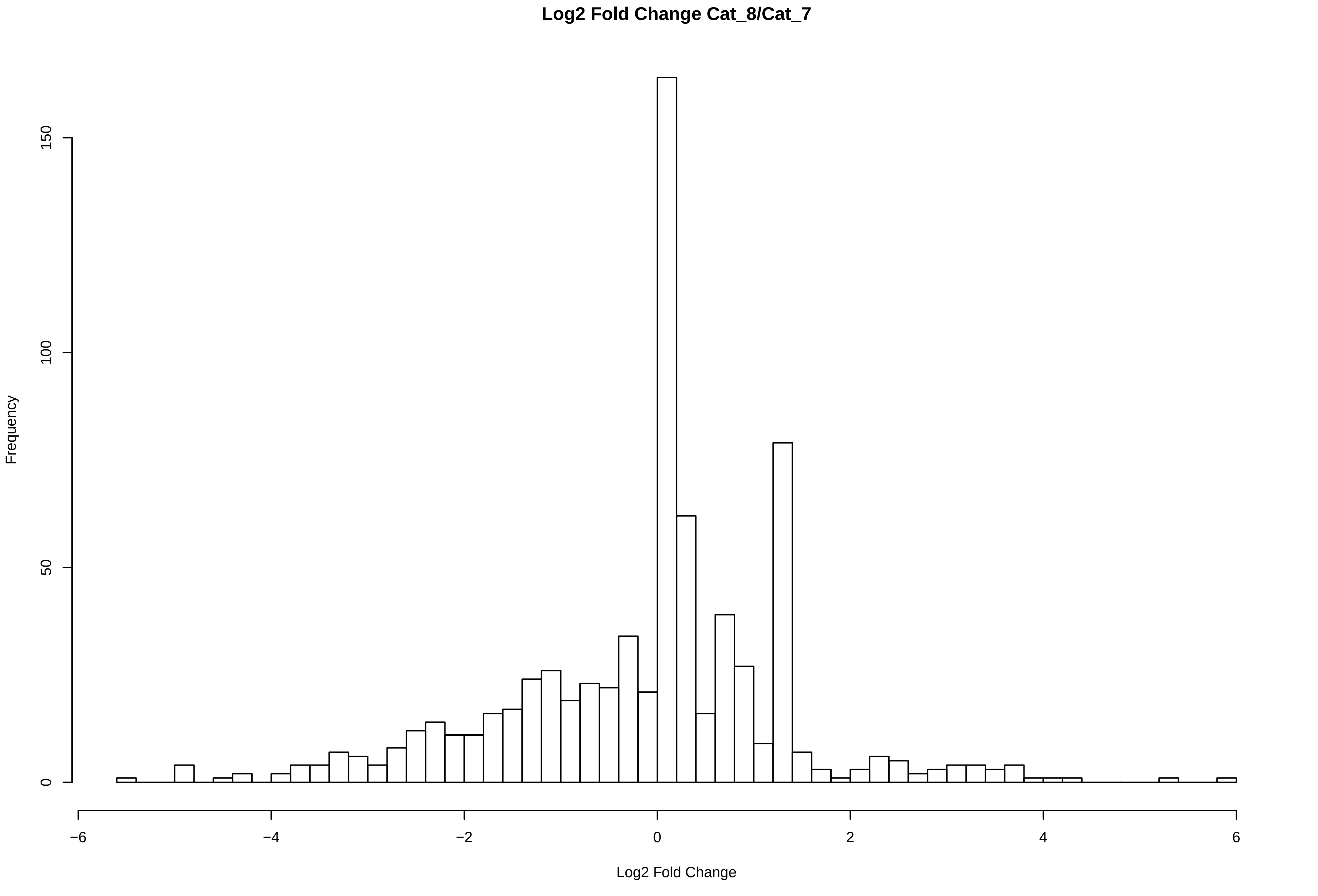
Log2 Fold Change Cat_8/Cat_7
Frequency
0
50
100
150
−6
−4
−2
0
2
4
6
Log2 Fold Change

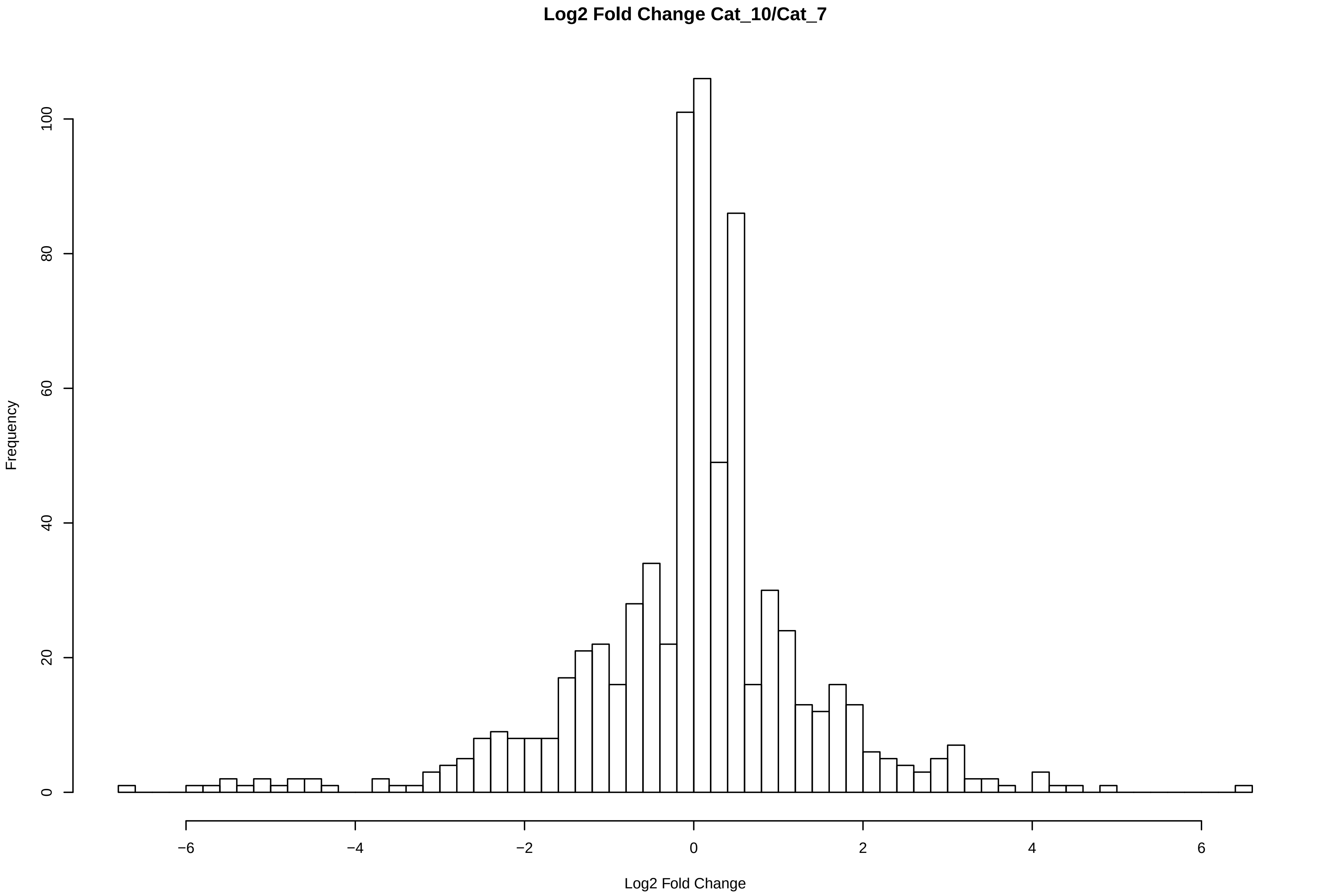

Log2 Fold Change Cat_10/Cat_7
Frequency
0
20
40
60
80
100
−6
−4
−2
0
2
4
6
Log2 Fold Change

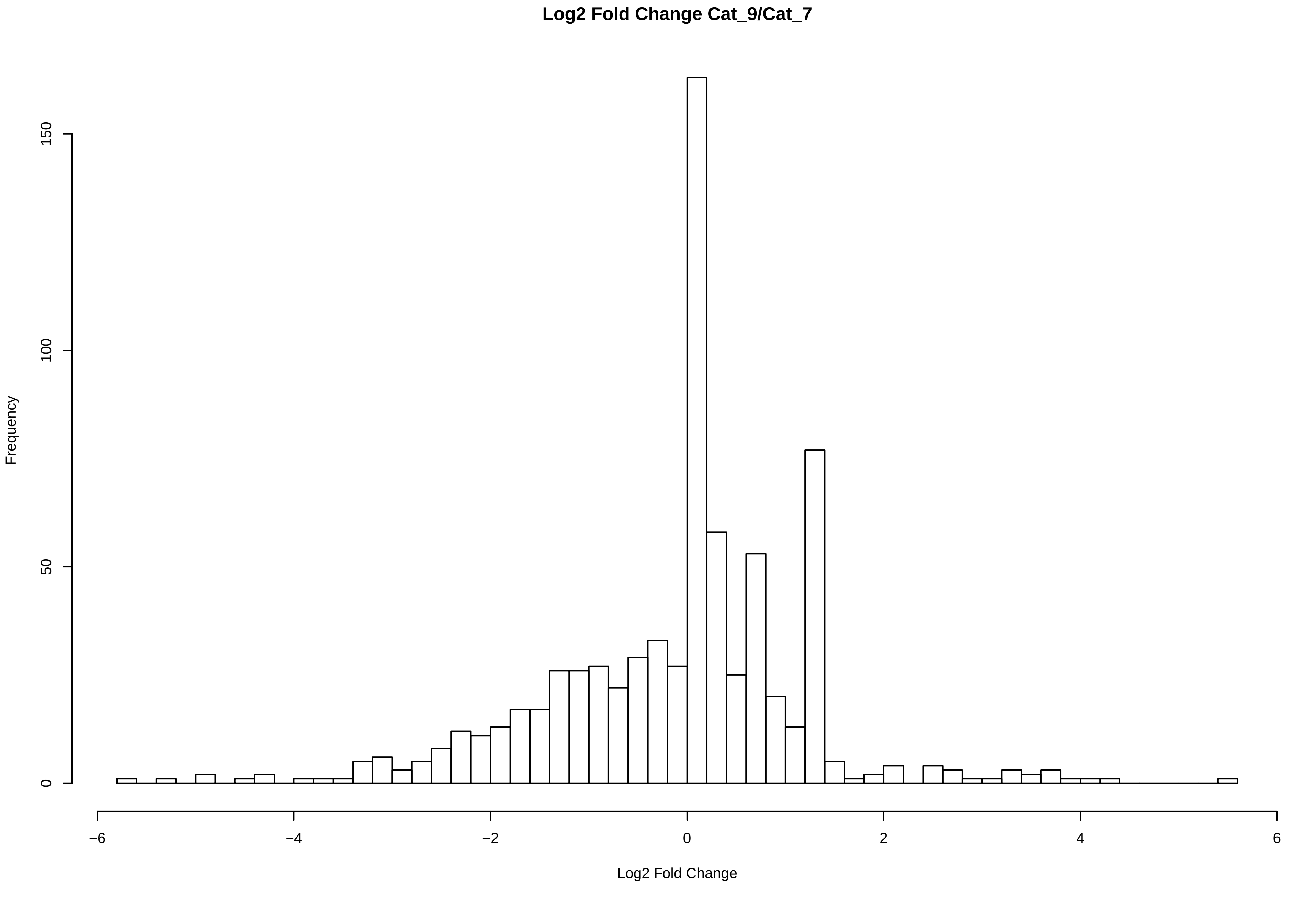
Log2 Fold Change Cat_9/Cat_7
Frequency
150
100
50
0
−6
−4
−2
0
2
4
6
Log2 Fold Change

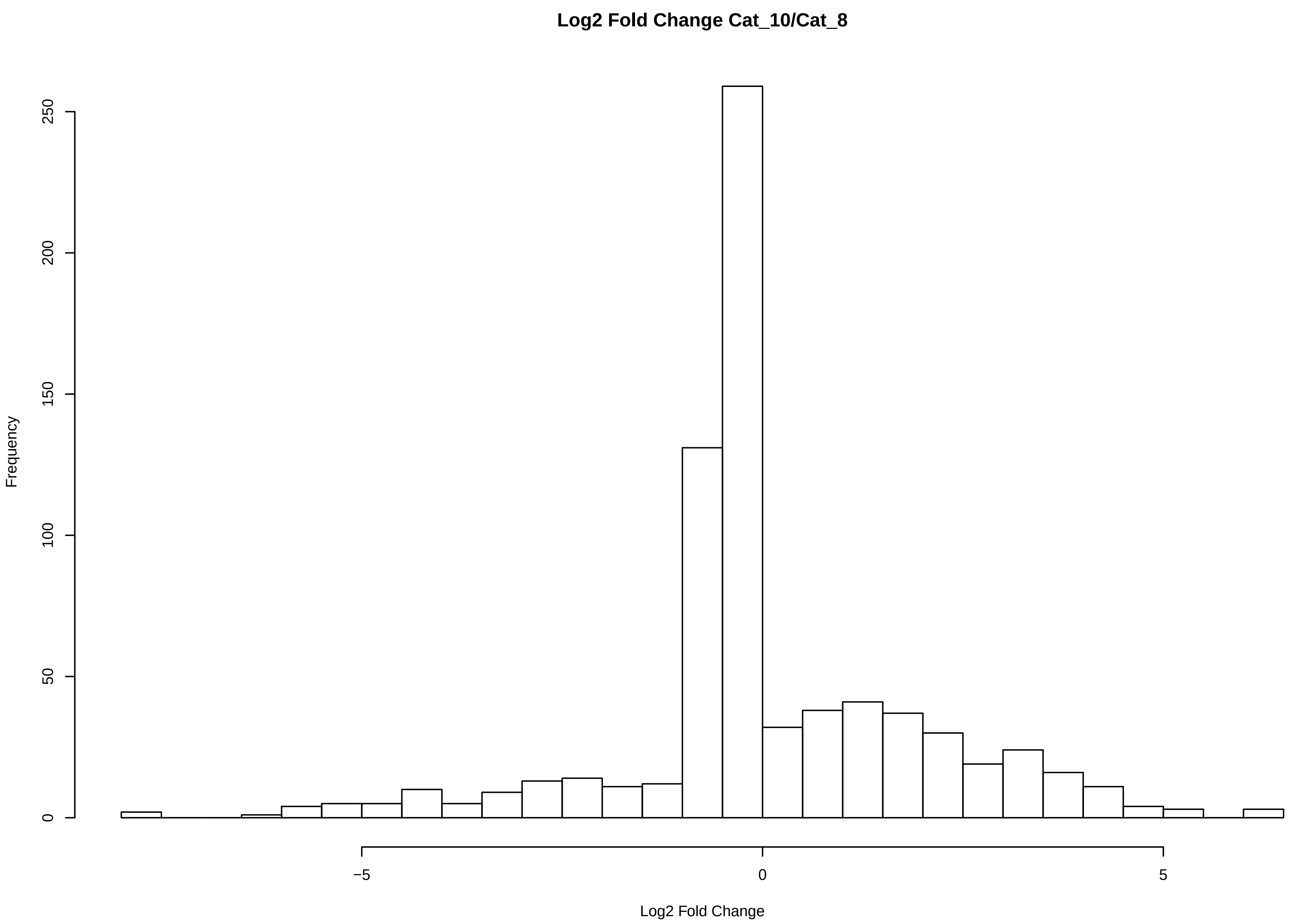

Log2 Fold Change Cat_10/Cat_8
Frequency
0
50
100
150
200
250
−5
0
5
Log2 Fold Change

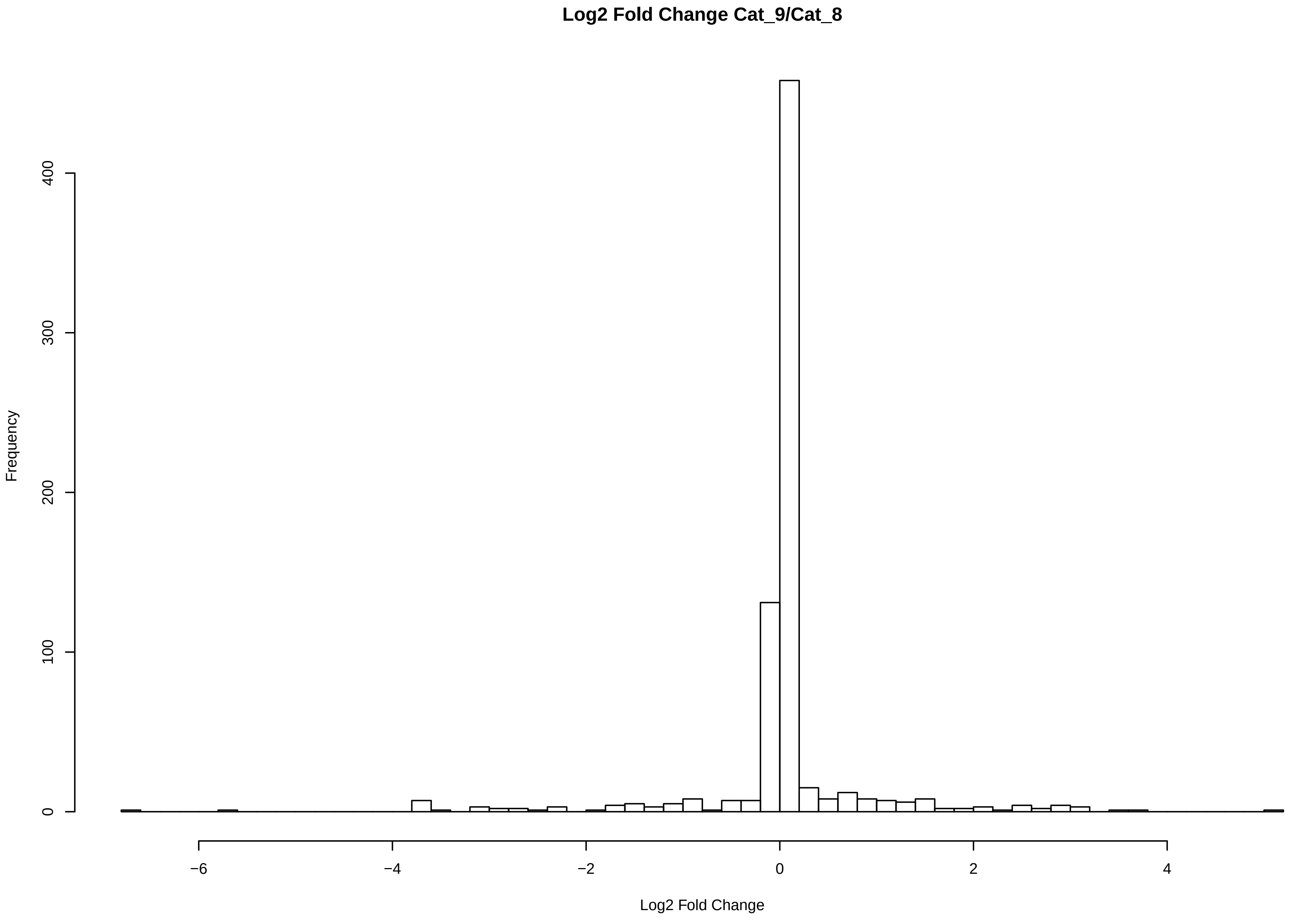

Log2 Fold Change Cat_9/Cat_8
Frequency
0
100
200
300
400
−6
−4
−2
0
2
4
Log2 Fold Change

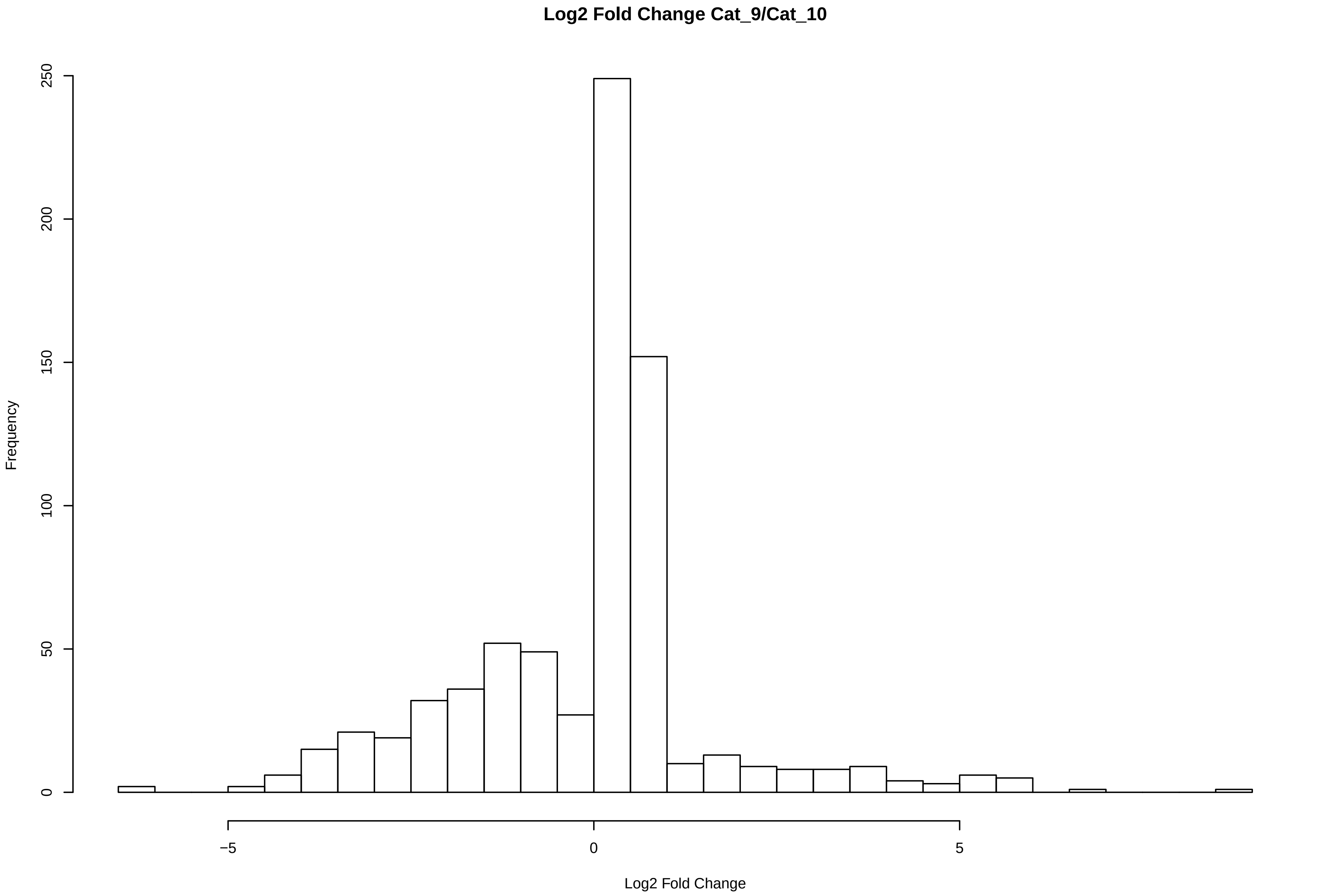

Log2 Fold Change Cat_9/Cat_10
Frequency
0
50
100
150
200
250
−5
0
5
Log2 Fold Change
