# Supplementary figures and images for: Inhibiting host-protein deposition on urinary catheters reduces associated urinary tract infections (part 2 of 2)
Source: eLife. 2022 Mar 29;11:e75798. doi: 10.7554/eLife.75798 (PMC8986317; doi:10.7554/eLife.75798)

**P-value vs Fold change**

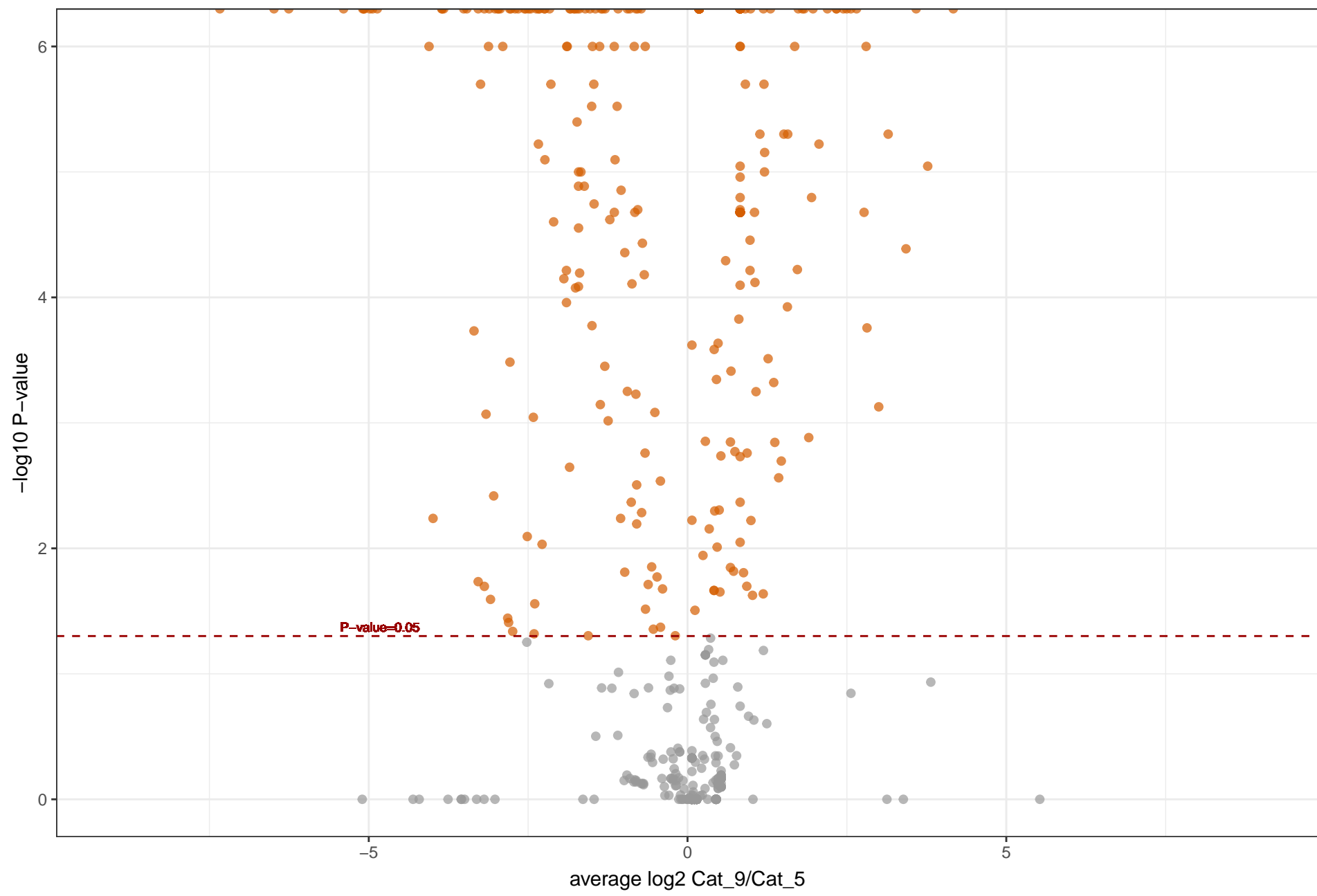

Supplement: Figure 6—source data 1. — Individual data from all figures involving small datasets displayed in individual tabs of this source file. This includes Figures 1B and 2A-F, Figure 3B, Figure 4, Figure 1—figure supplement 1 and Figure 2—figure supplement 1. [file elife-75798-fig6-data1.zip › Flores_Data/AF1_Cat_9.Cat_5-volcano_AFCat1.pdf]

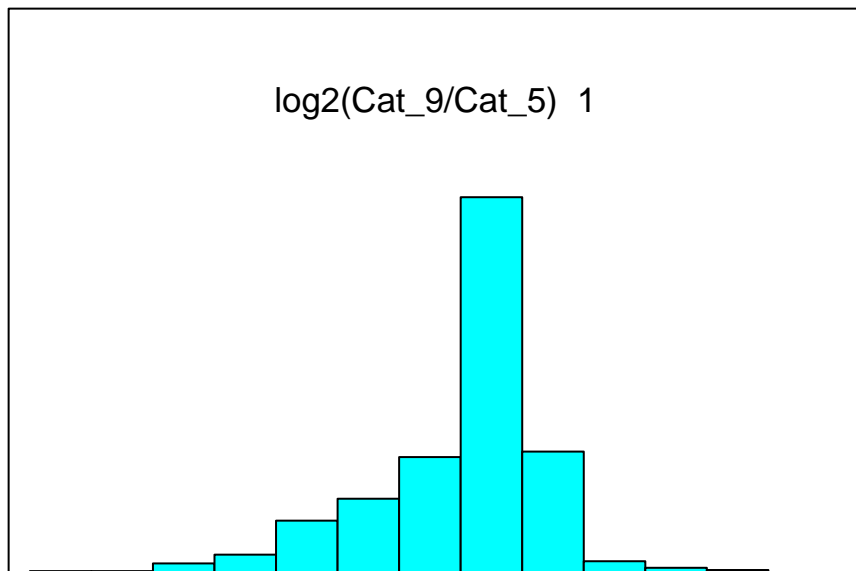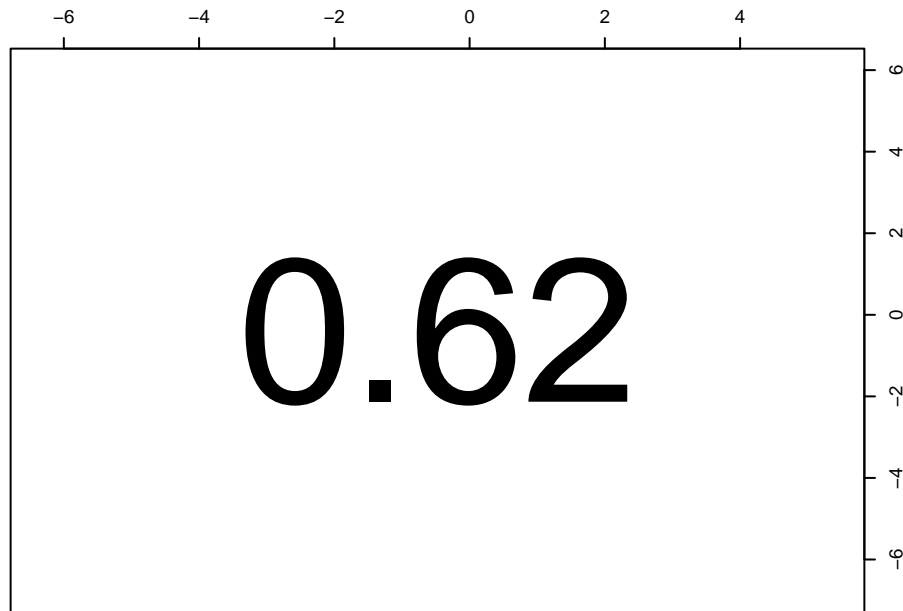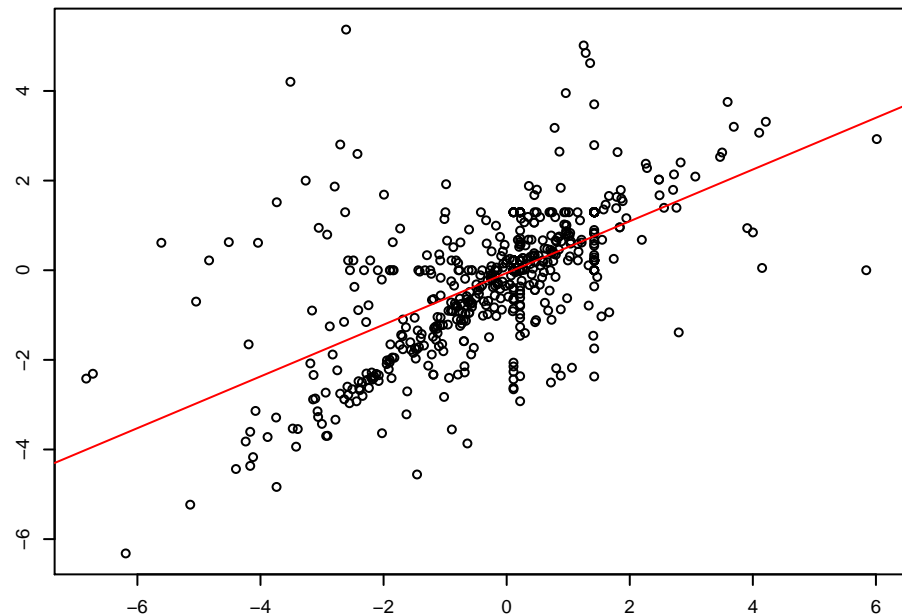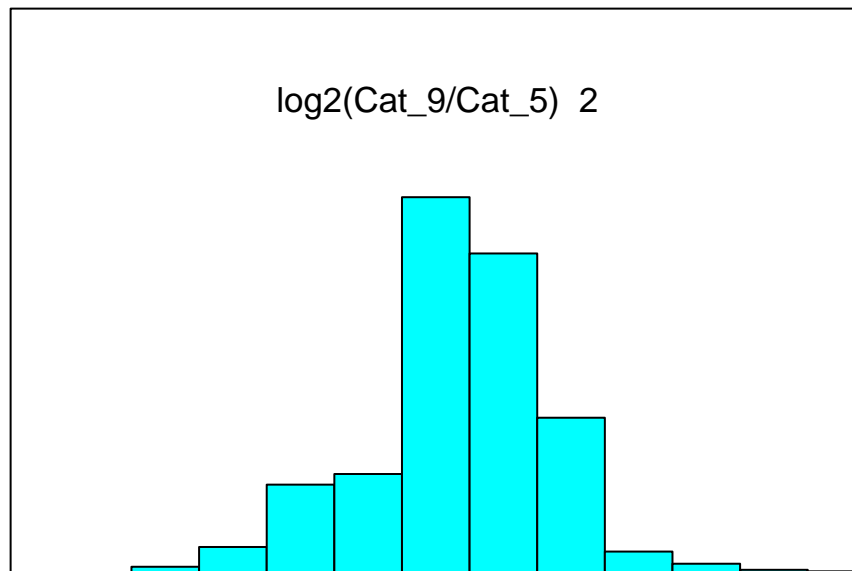

Supplement: Figure 6—source data 1. — Individual data from all figures involving small datasets displayed in individual tabs of this source file. This includes Figures 1B and 2A-F, Figure 3B, Figure 4, Figure 1—figure supplement 1 and Figure 2—figure supplement 1. [file elife-75798-fig6-data1.zip › Flores_Data/AF1_Cat_9.Cat_5-reproducibility_AFCat1.pdf]

Value-ordered fold change

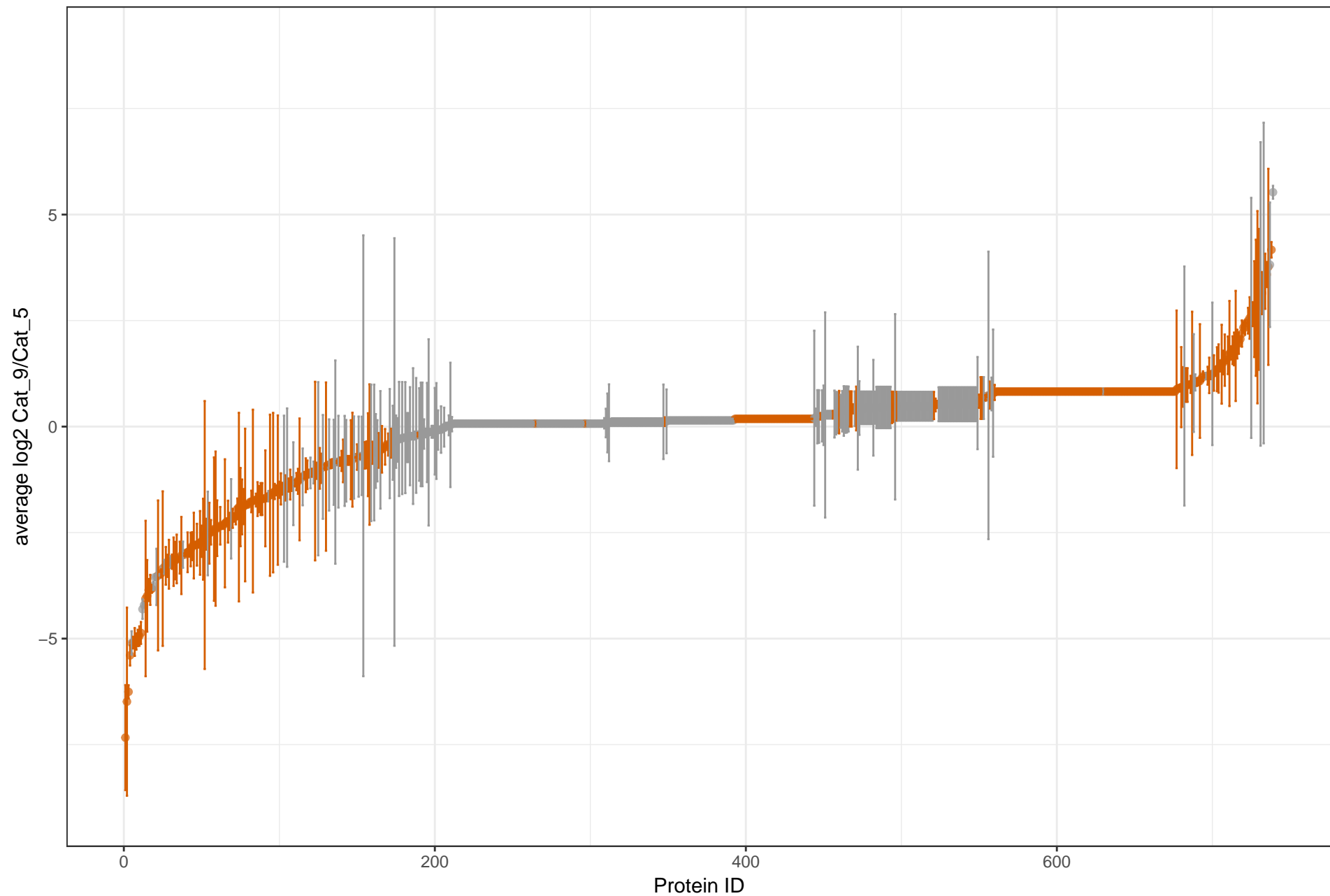

Supplement: Figure 6—source data 1. — Individual data from all figures involving small datasets displayed in individual tabs of this source file. This includes Figures 1B and 2A-F, Figure 3B, Figure 4, Figure 1—figure supplement 1 and Figure 2—figure supplement 1. [file elife-75798-fig6-data1.zip › Flores_Data/AF1_Cat_9.Cat_5-value-ordered-log-ratio_AFCat1.pdf]

MA plot

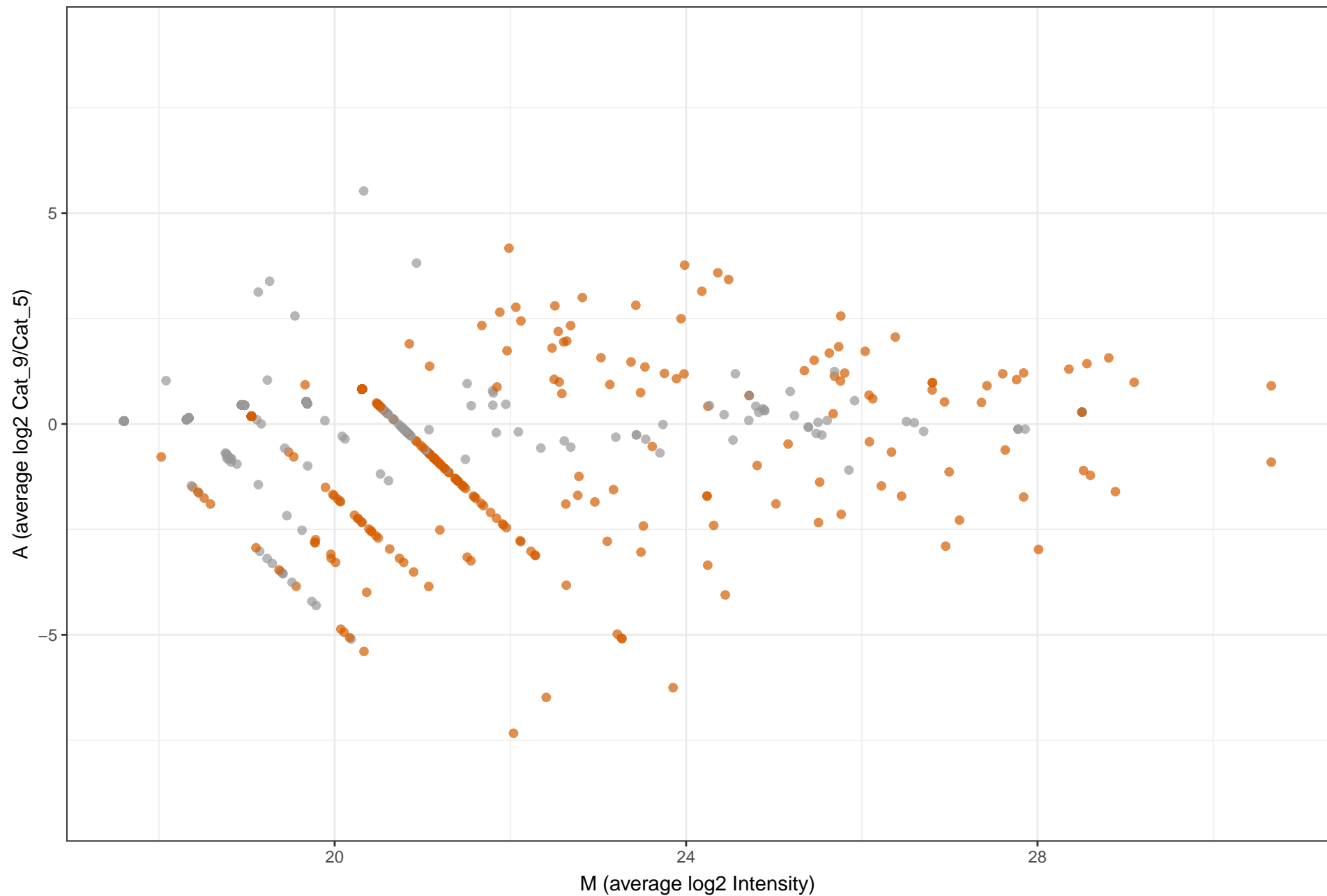

Supplement: Figure 6—source data 1. — Individual data from all figures involving small datasets displayed in individual tabs of this source file. This includes Figures 1B and 2A-F, Figure 3B, Figure 4, Figure 1—figure supplement 1 and Figure 2—figure supplement 1. [file elife-75798-fig6-data1.zip › Flores_Data/AF1_Cat_9.Cat_5-MA_AFCat1.pdf]

P-value vs Fold change

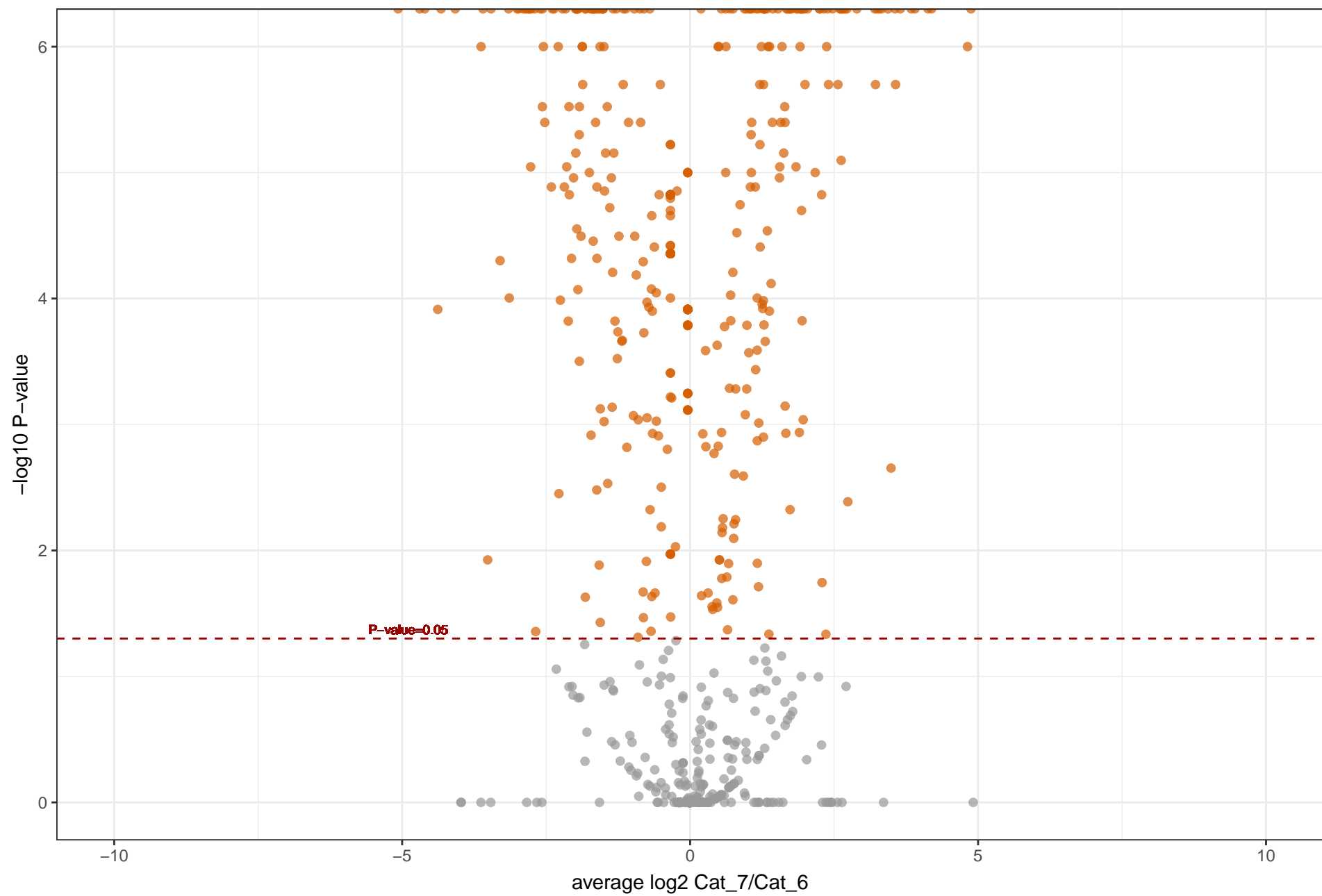

Supplement: Figure 6—source data 1. — Individual data from all figures involving small datasets displayed in individual tabs of this source file. This includes Figures 1B and 2A-F, Figure 3B, Figure 4, Figure 1—figure supplement 1 and Figure 2—figure supplement 1. [file elife-75798-fig6-data1.zip › Flores_Data/AF1_Cat_7.Cat_6-volcano_AFCat1.pdf]

MA plot

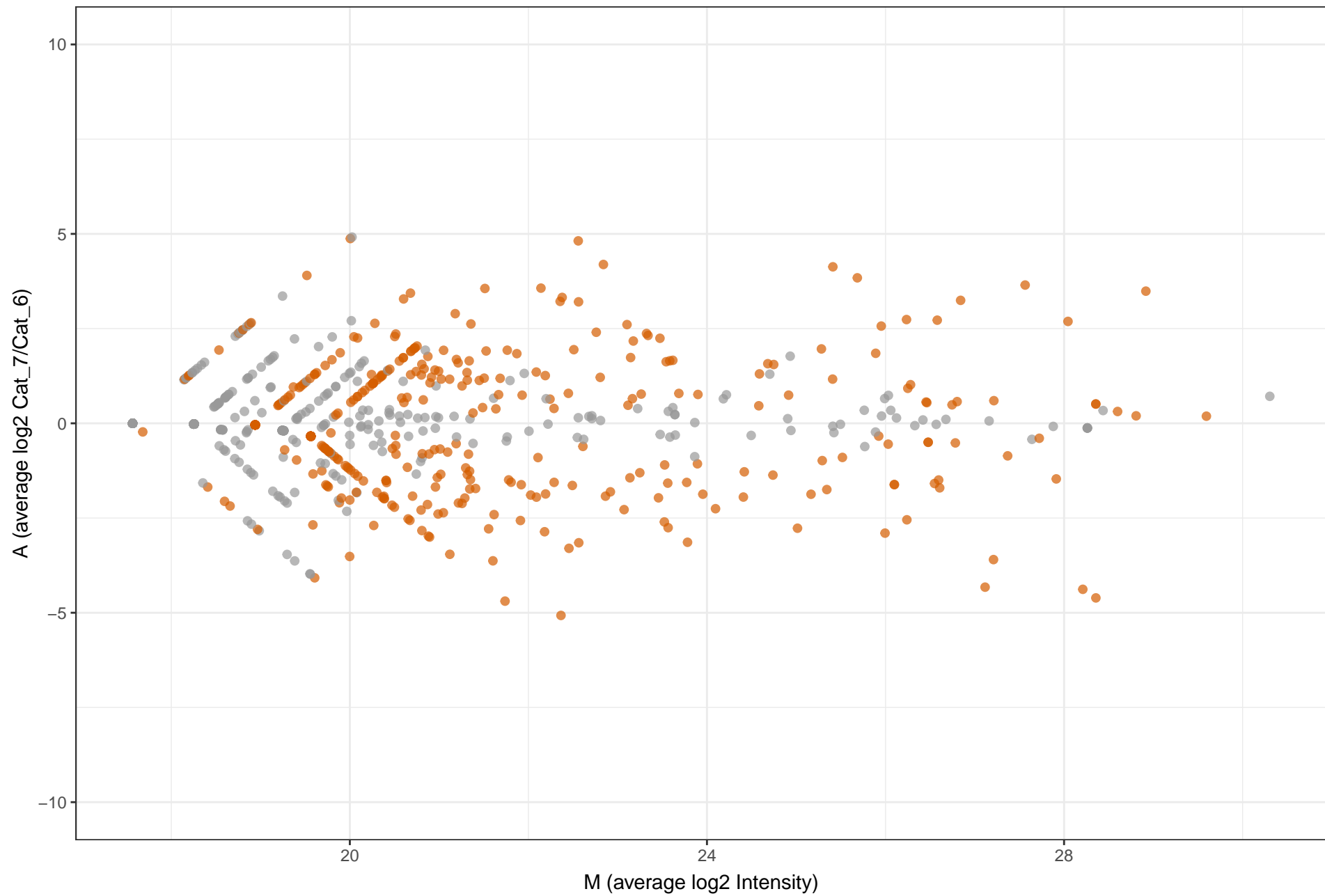

Supplement: Figure 6—source data 1. — Individual data from all figures involving small datasets displayed in individual tabs of this source file. This includes Figures 1B and 2A-F, Figure 3B, Figure 4, Figure 1—figure supplement 1 and Figure 2—figure supplement 1. [file elife-75798-fig6-data1.zip › Flores_Data/AF1_Cat_7.Cat_6-MA_AFCat1.pdf]

Value-ordered fold change

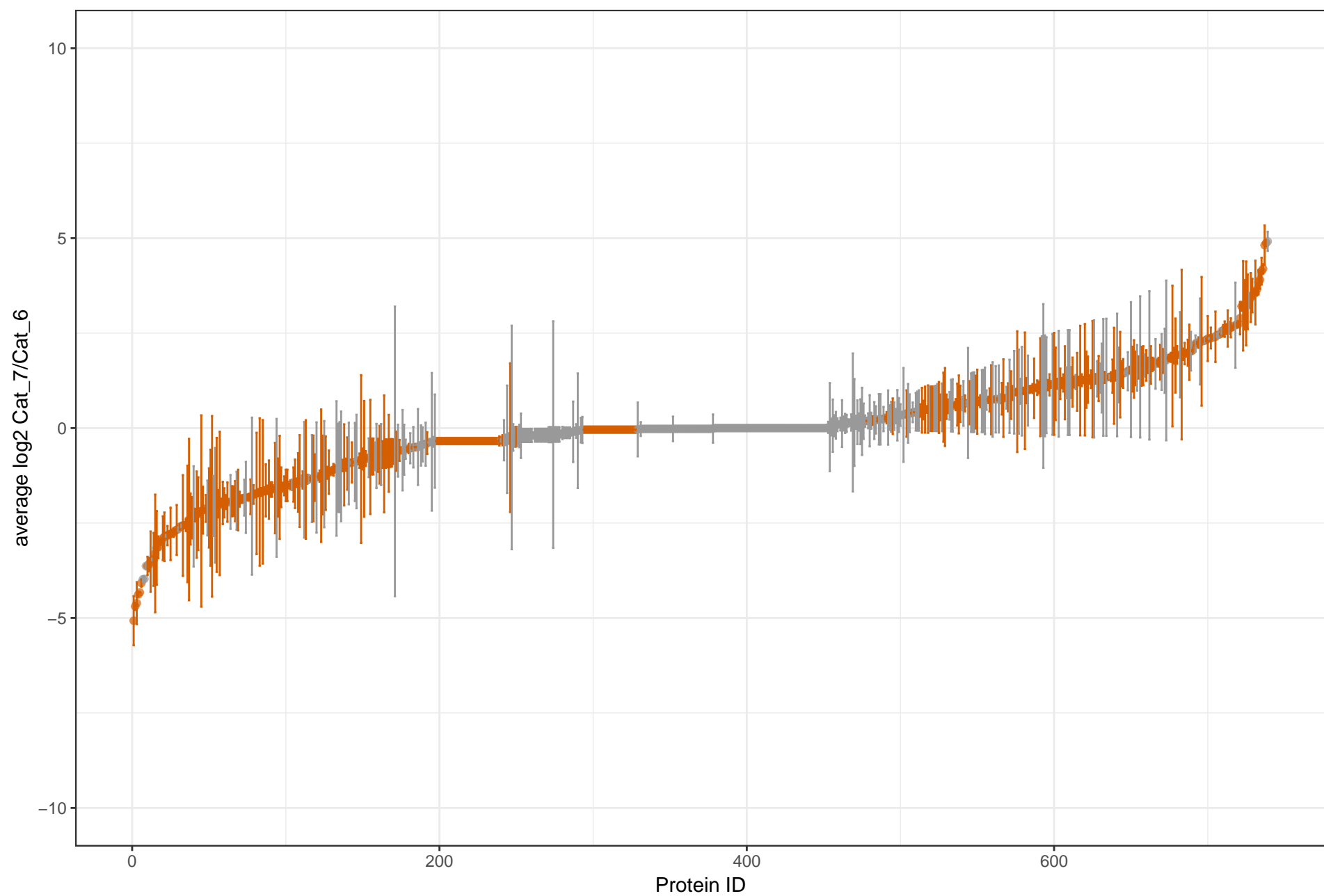

Supplement: Figure 6—source data 1. — Individual data from all figures involving small datasets displayed in individual tabs of this source file. This includes Figures 1B and 2A-F, Figure 3B, Figure 4, Figure 1—figure supplement 1 and Figure 2—figure supplement 1. [file elife-75798-fig6-data1.zip › Flores_Data/AF1_Cat_7.Cat_6-value-ordered-log-ratio_AFCat1.pdf]

P-value vs Fold change

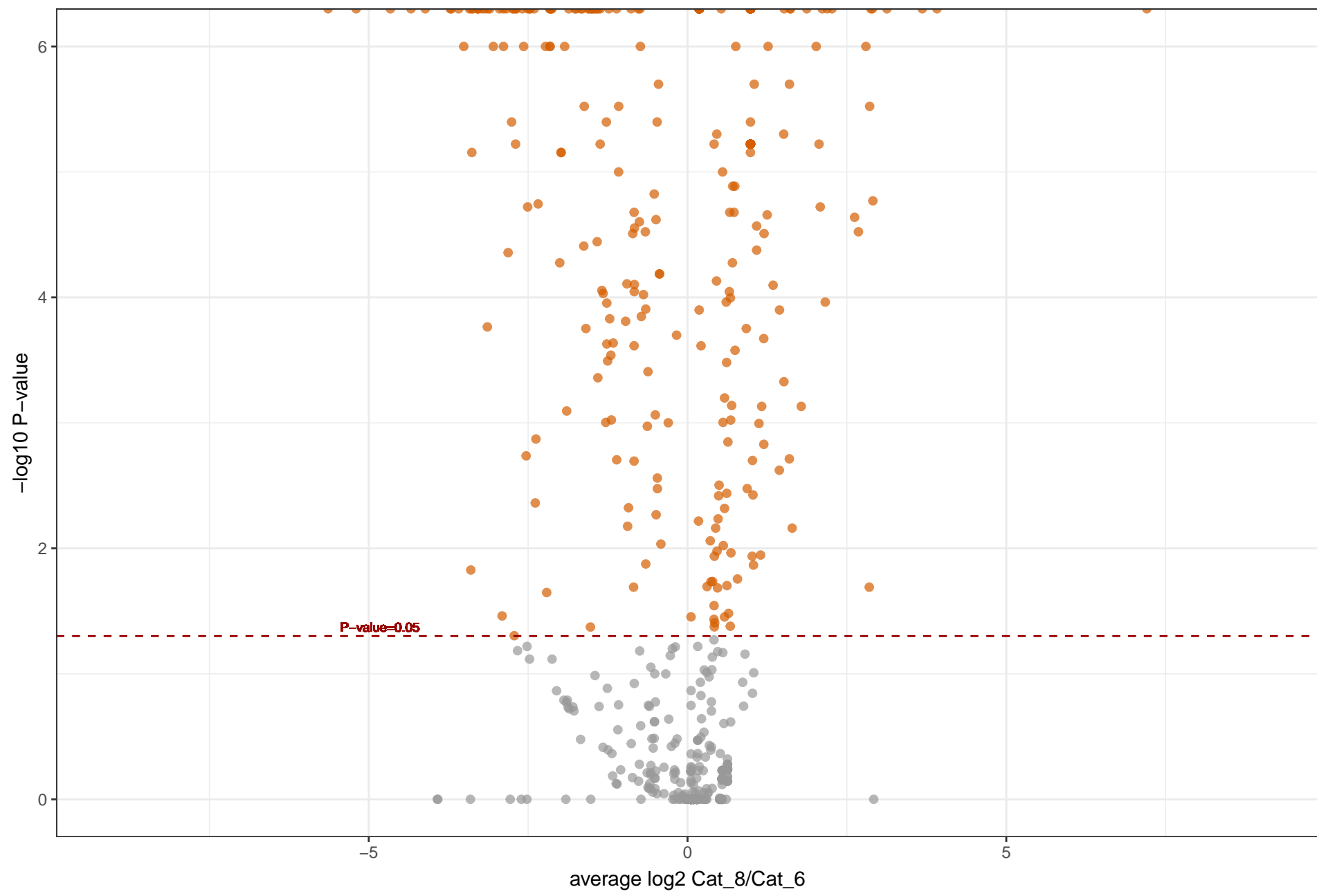

Supplement: Figure 6—source data 1. — Individual data from all figures involving small datasets displayed in individual tabs of this source file. This includes Figures 1B and 2A-F, Figure 3B, Figure 4, Figure 1—figure supplement 1 and Figure 2—figure supplement 1. [file elife-75798-fig6-data1.zip › Flores_Data/AF1_Cat_8.Cat_6-volcano_AFCat1.pdf]

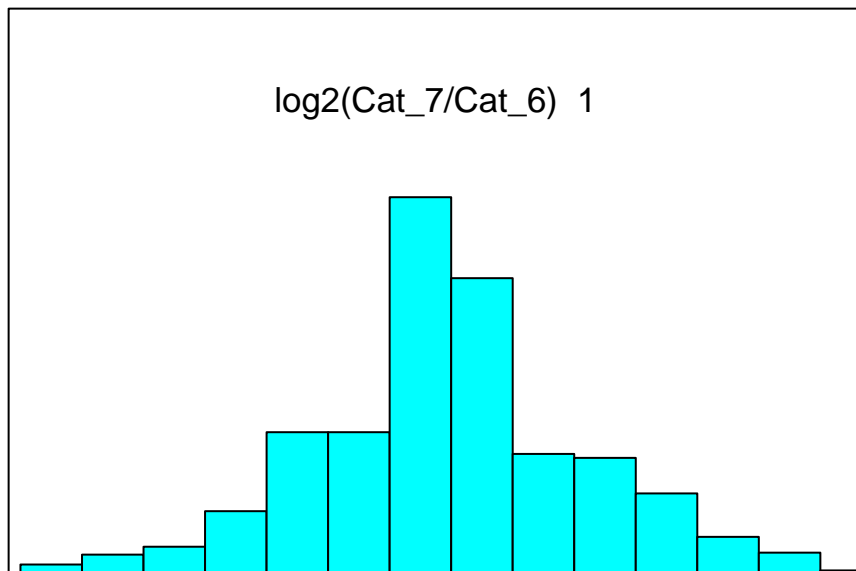

-6 -4 -2 0 2 4 6

6  
4  
2  
0  
-2  
-4  
-6

0.82

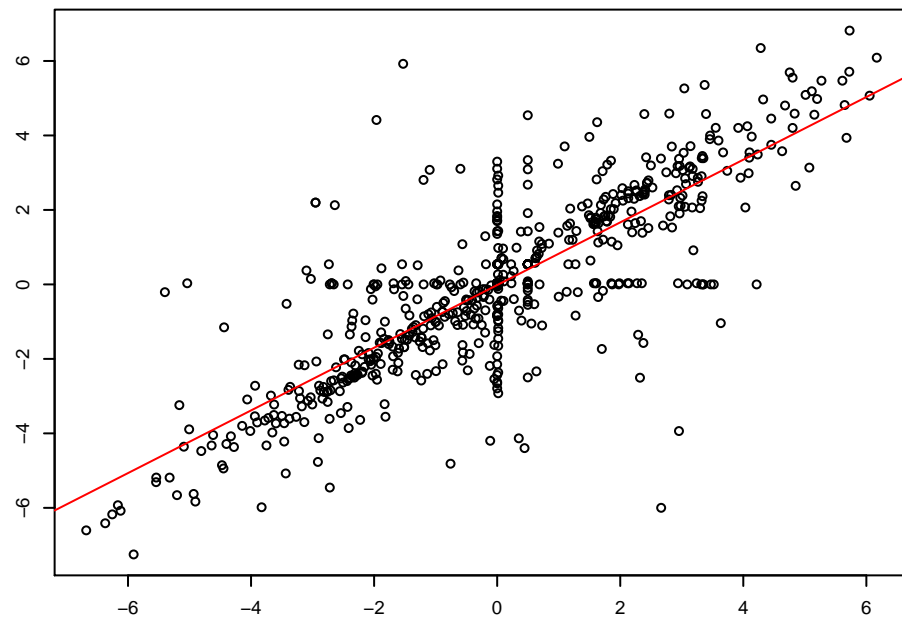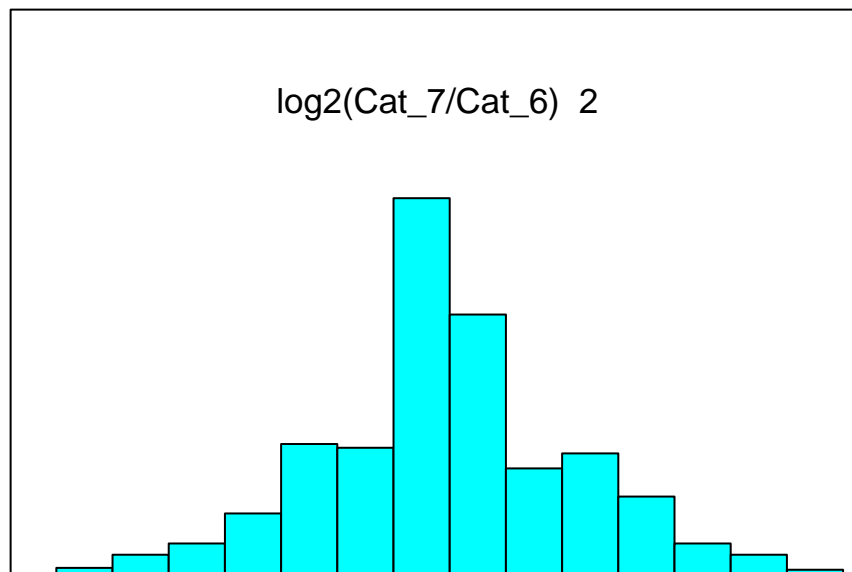

Supplement: Figure 6—source data 1. — Individual data from all figures involving small datasets displayed in individual tabs of this source file. This includes Figures 1B and 2A-F, Figure 3B, Figure 4, Figure 1—figure supplement 1 and Figure 2—figure supplement 1. [file elife-75798-fig6-data1.zip › Flores_Data/AF1_Cat_7.Cat_6-reproducibility_AFCat1.pdf]

MA plot

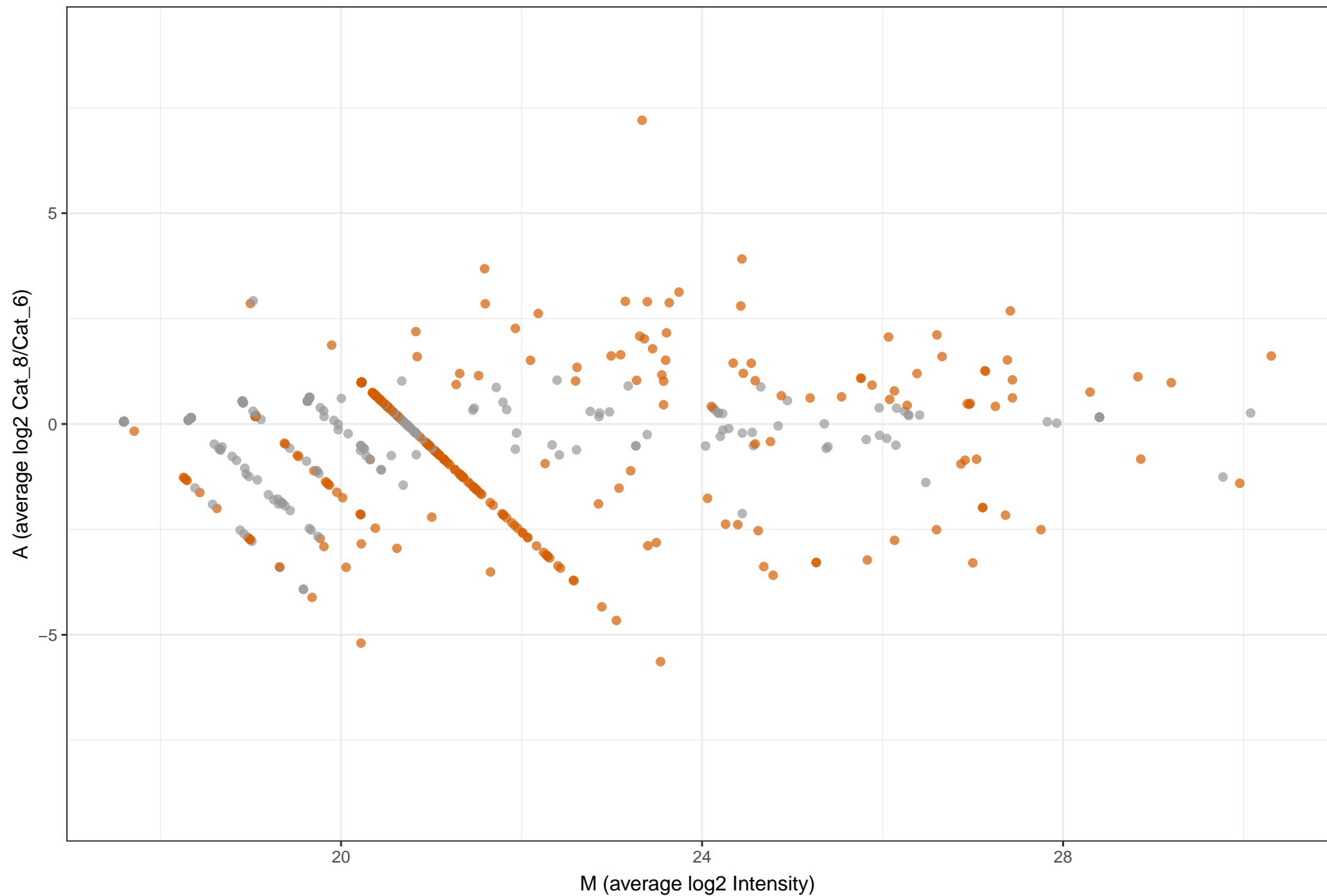

Supplement: Figure 6—source data 1. — Individual data from all figures involving small datasets displayed in individual tabs of this source file. This includes Figures 1B and 2A-F, Figure 3B, Figure 4, Figure 1—figure supplement 1 and Figure 2—figure supplement 1. [file elife-75798-fig6-data1.zip › Flores_Data/AF1_Cat_8.Cat_6-MA_AFCat1.pdf]

Value-ordered fold change

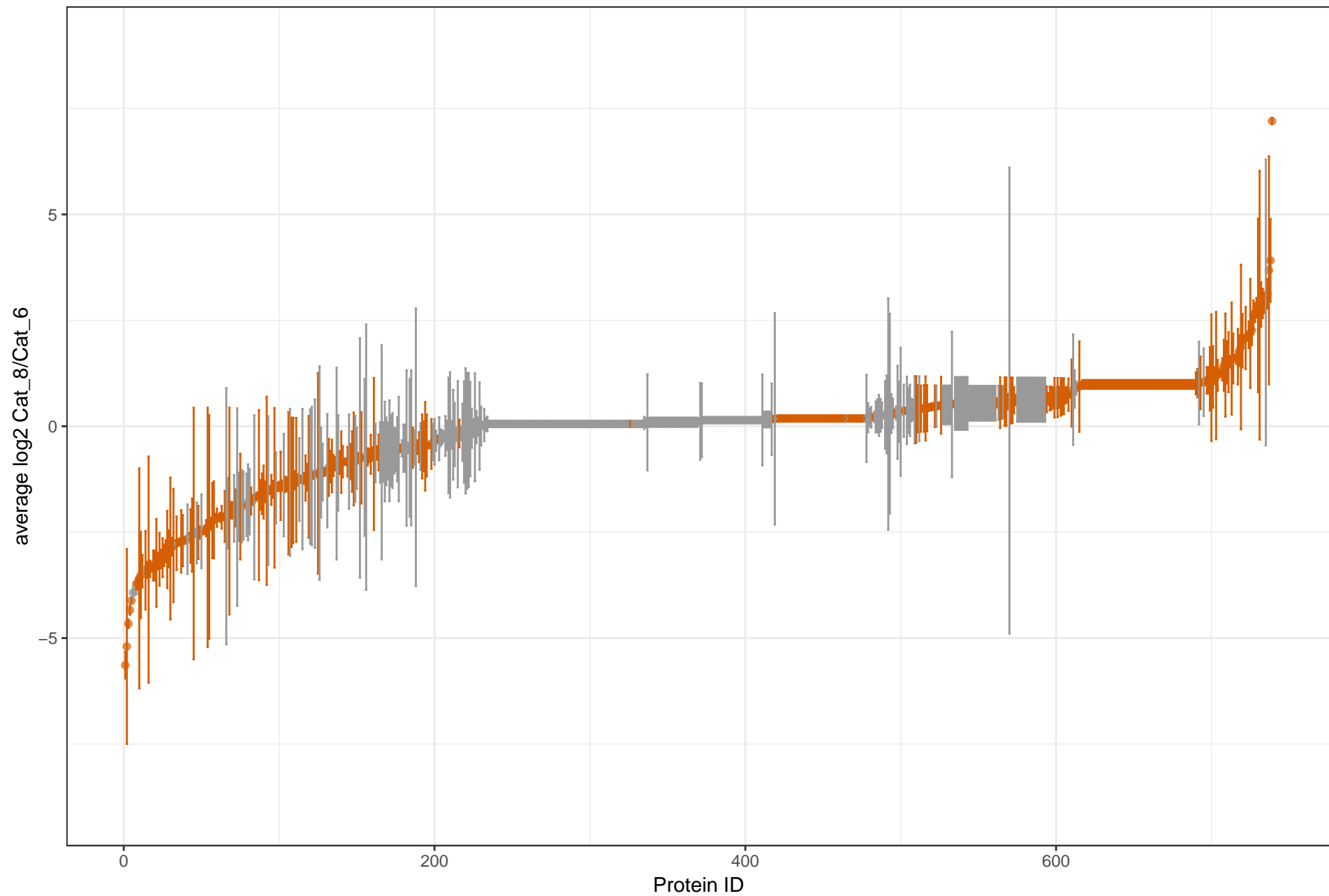

Supplement: Figure 6—source data 1. — Individual data from all figures involving small datasets displayed in individual tabs of this source file. This includes Figures 1B and 2A-F, Figure 3B, Figure 4, Figure 1—figure supplement 1 and Figure 2—figure supplement 1. [file elife-75798-fig6-data1.zip › Flores_Data/AF1_Cat_8.Cat_6-value-ordered-log-ratio_AFCat1.pdf]

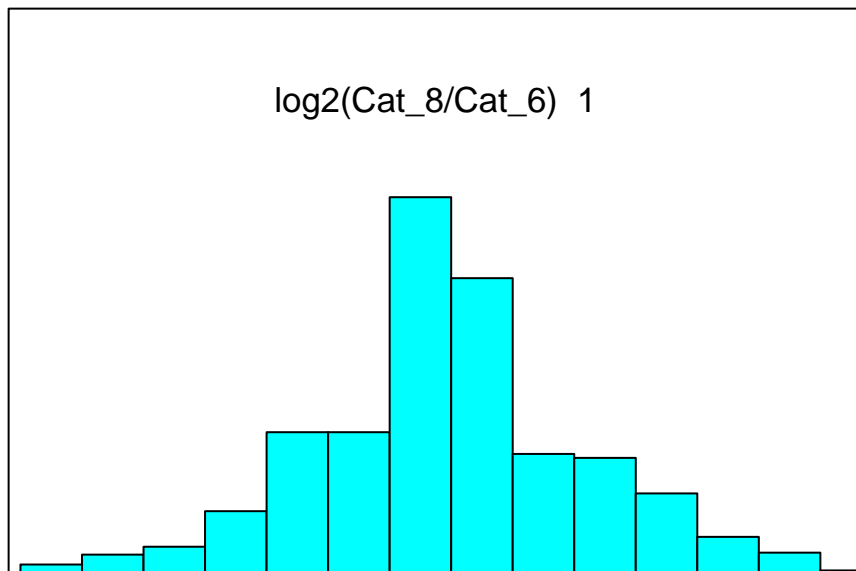

-6 -4 -2 0 2 4 6

6  
4  
2  
0  
-2  
-4  
-6

0.82

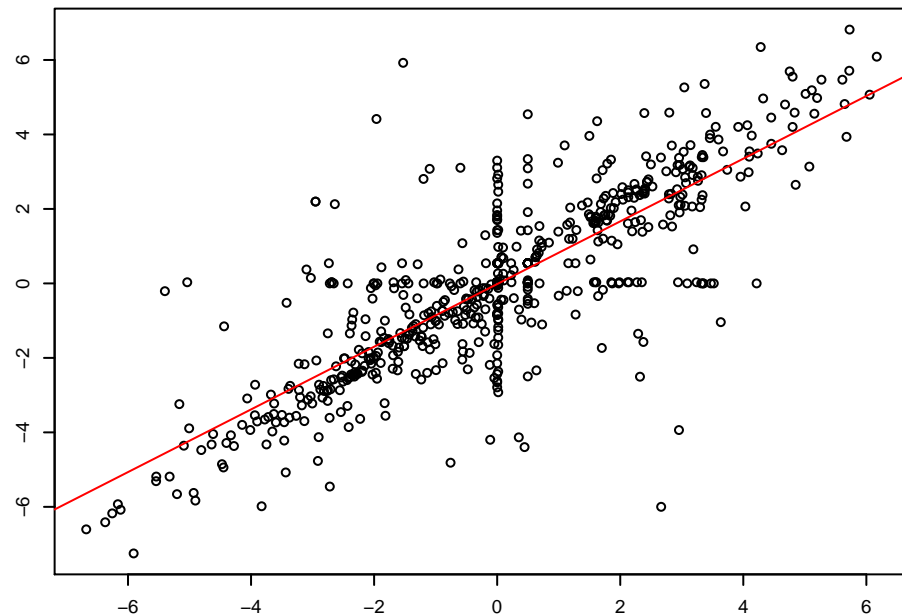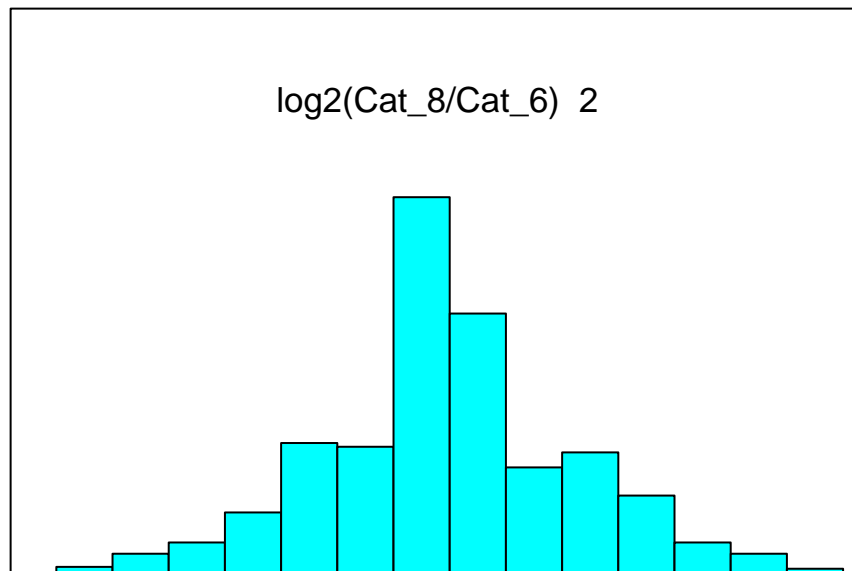

Supplement: Figure 6—source data 1. — Individual data from all figures involving small datasets displayed in individual tabs of this source file. This includes Figures 1B and 2A-F, Figure 3B, Figure 4, Figure 1—figure supplement 1 and Figure 2—figure supplement 1. [file elife-75798-fig6-data1.zip › Flores_Data/AF1_Cat_8.Cat_6-reproducibility_AFCat1.pdf]

P-value vs Fold change

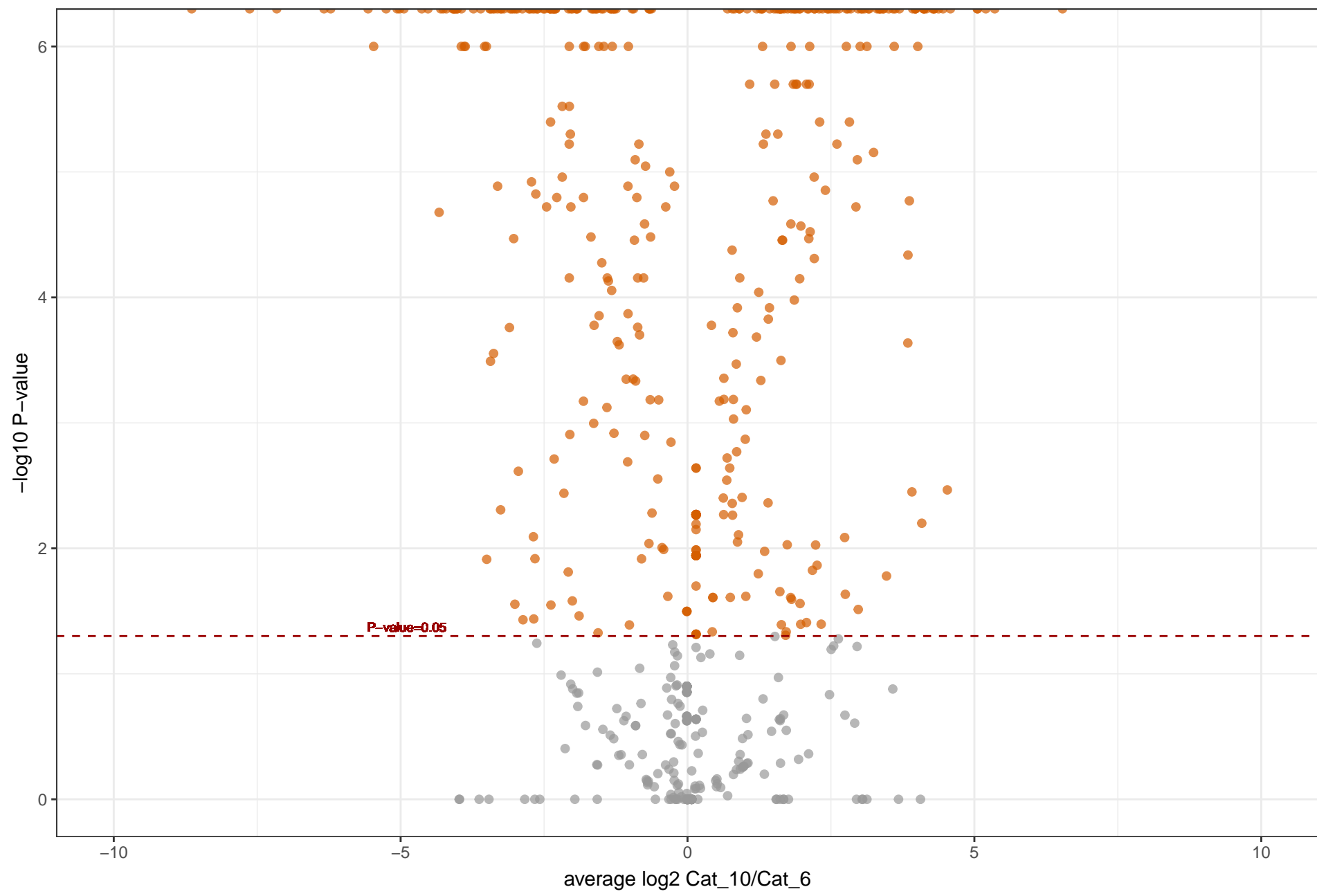

Supplement: Figure 6—source data 1. — Individual data from all figures involving small datasets displayed in individual tabs of this source file. This includes Figures 1B and 2A-F, Figure 3B, Figure 4, Figure 1—figure supplement 1 and Figure 2—figure supplement 1. [file elife-75798-fig6-data1.zip › Flores_Data/AF1_Cat_10.Cat_6-volcano_AFCat1.pdf]

Value-ordered fold change

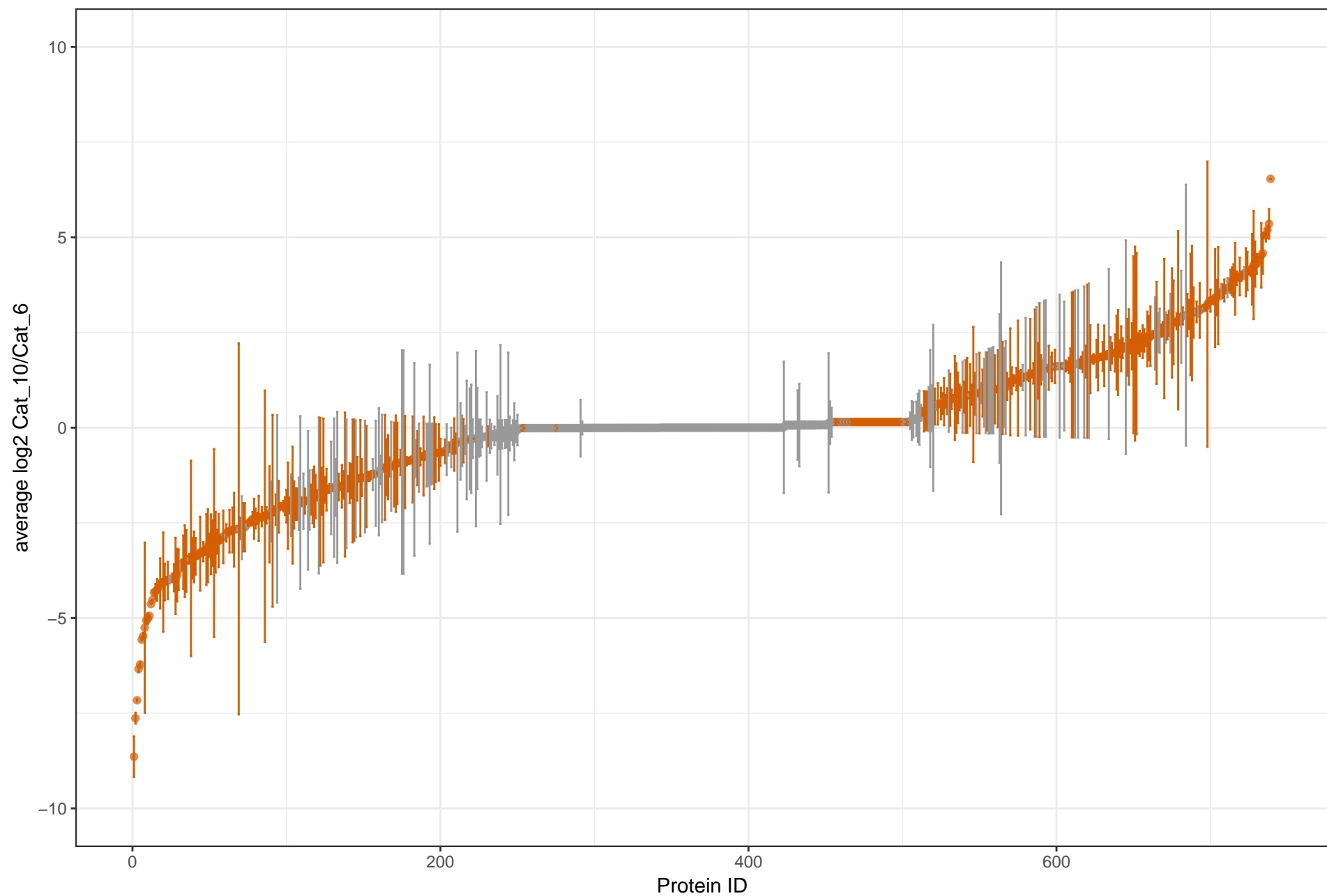

Supplement: Figure 6—source data 1. — Individual data from all figures involving small datasets displayed in individual tabs of this source file. This includes Figures 1B and 2A-F, Figure 3B, Figure 4, Figure 1—figure supplement 1 and Figure 2—figure supplement 1. [file elife-75798-fig6-data1.zip › Flores_Data/AF1_Cat_10.Cat_6-value-ordered-log-ratio_AFCat1.pdf]

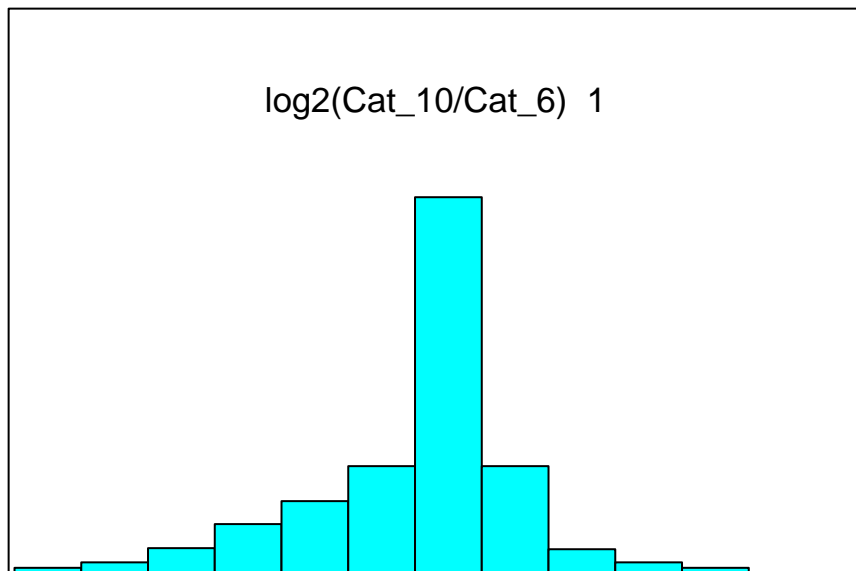

-6 -4 -2 0 2 4 6

6

4

2

0

-2

-4

-6

0.72

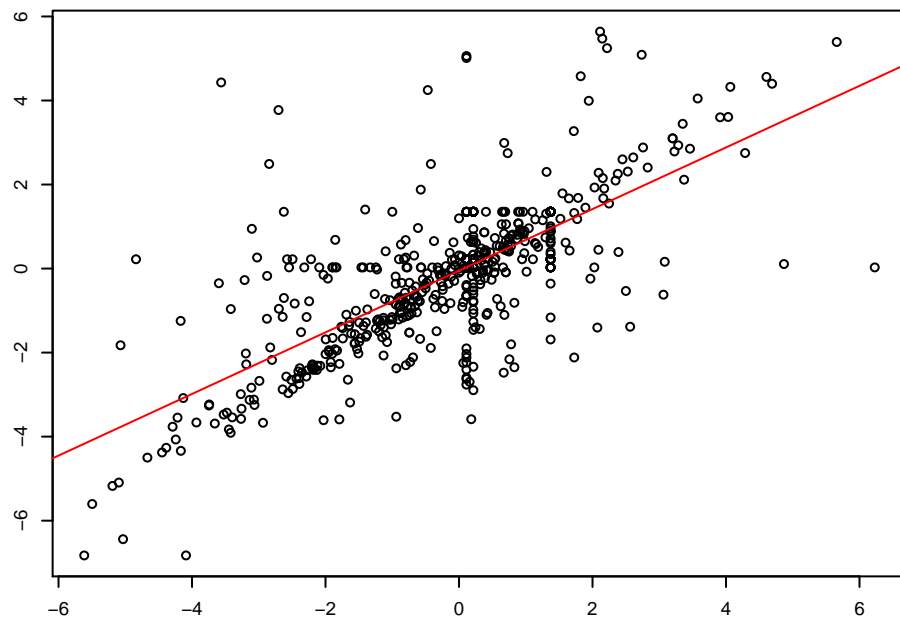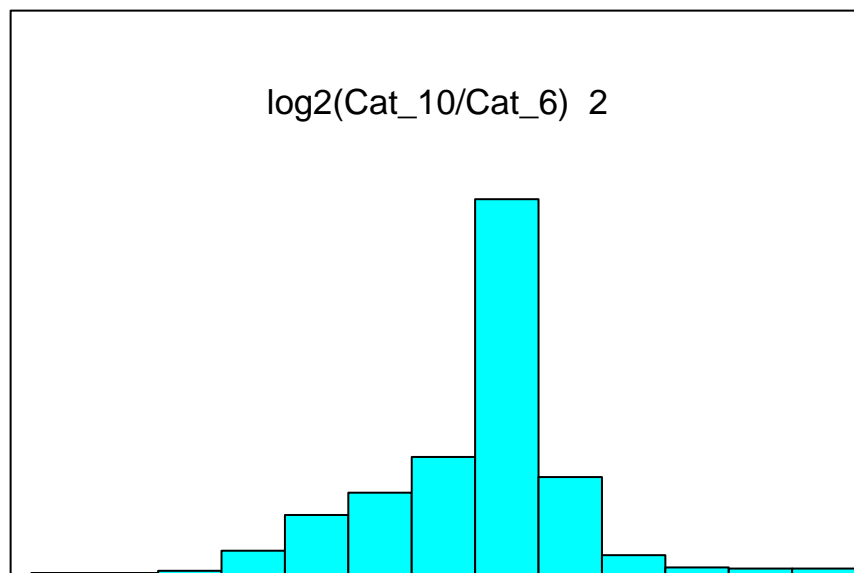

Supplement: Figure 6—source data 1. — Individual data from all figures involving small datasets displayed in individual tabs of this source file. This includes Figures 1B and 2A-F, Figure 3B, Figure 4, Figure 1—figure supplement 1 and Figure 2—figure supplement 1. [file elife-75798-fig6-data1.zip › Flores_Data/AF1_Cat_10.Cat_6-reproducibility_AFCat1.pdf]

P-value vs Fold change

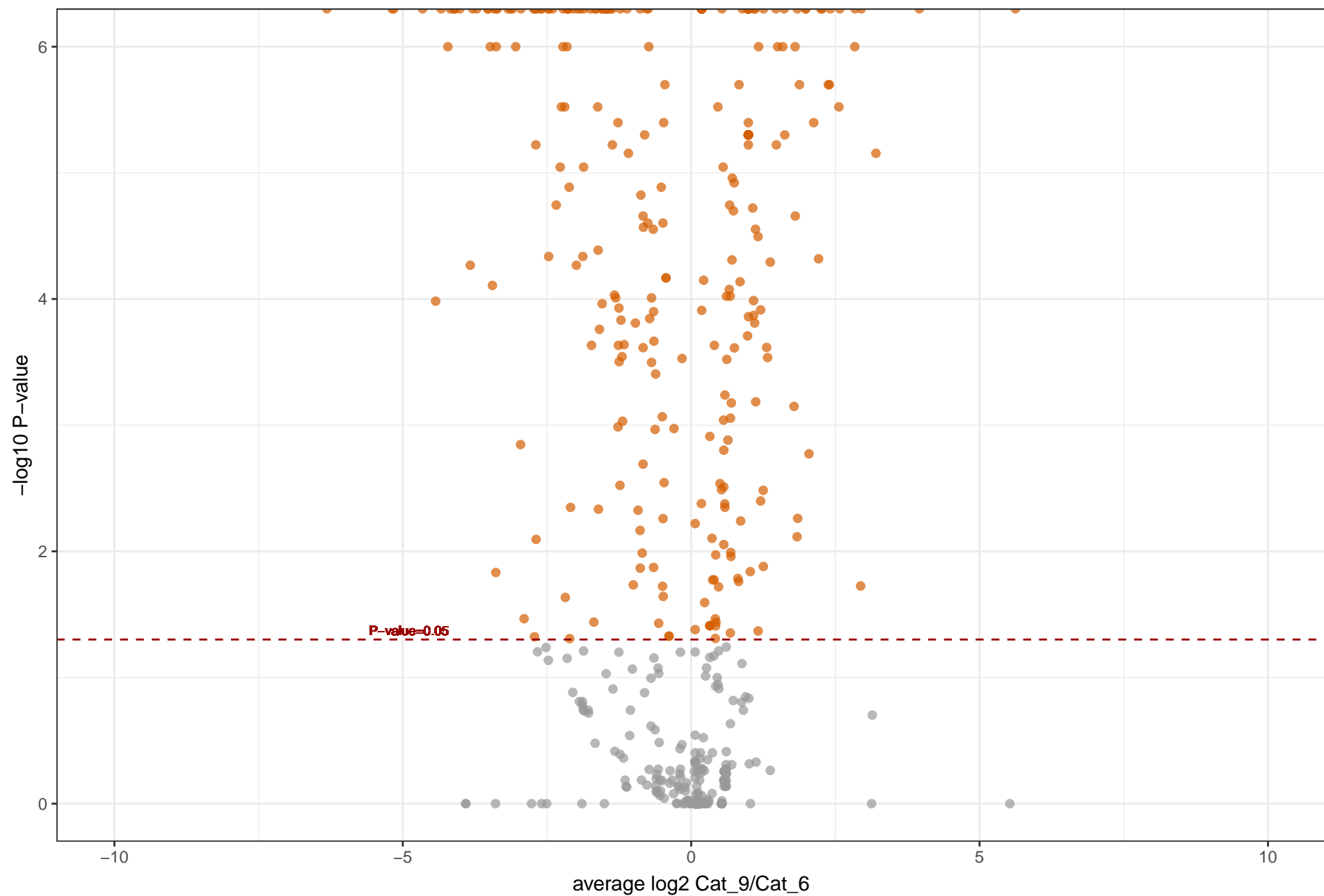

Supplement: Figure 6—source data 1. — Individual data from all figures involving small datasets displayed in individual tabs of this source file. This includes Figures 1B and 2A-F, Figure 3B, Figure 4, Figure 1—figure supplement 1 and Figure 2—figure supplement 1. [file elife-75798-fig6-data1.zip › Flores_Data/AF1_Cat_9.Cat_6-volcano_AFCat1.pdf]

MA plot

A (average log2 Cat\_10/Cat\_6)

10  
5  
0  
-5  
-10

M (average log2 Intensity)

20

24

28

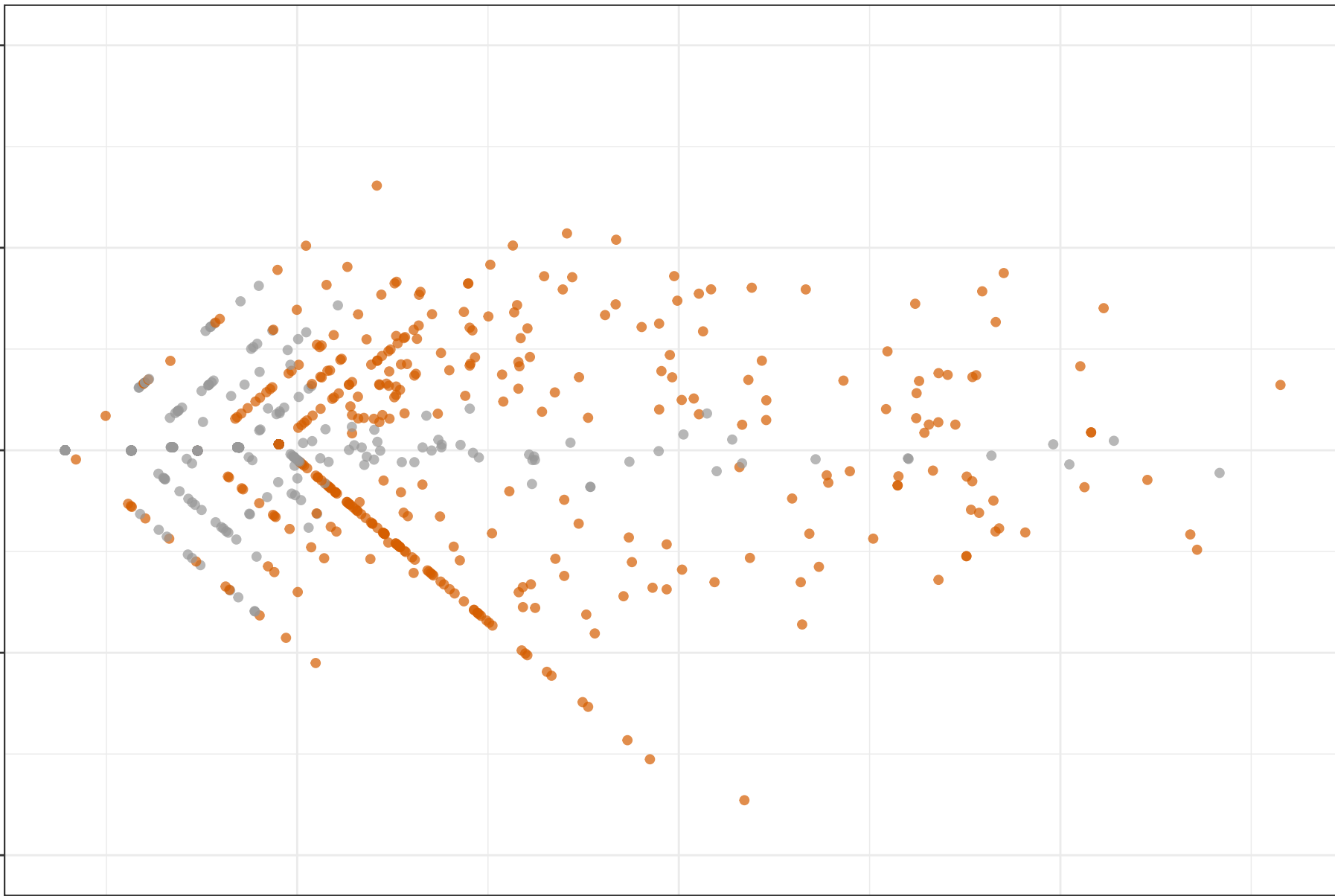

Supplement: Figure 6—source data 1. — Individual data from all figures involving small datasets displayed in individual tabs of this source file. This includes Figures 1B and 2A-F, Figure 3B, Figure 4, Figure 1—figure supplement 1 and Figure 2—figure supplement 1. [file elife-75798-fig6-data1.zip › Flores_Data/AF1_Cat_10.Cat_6-MA_AFCat1.pdf]

Value-ordered fold change

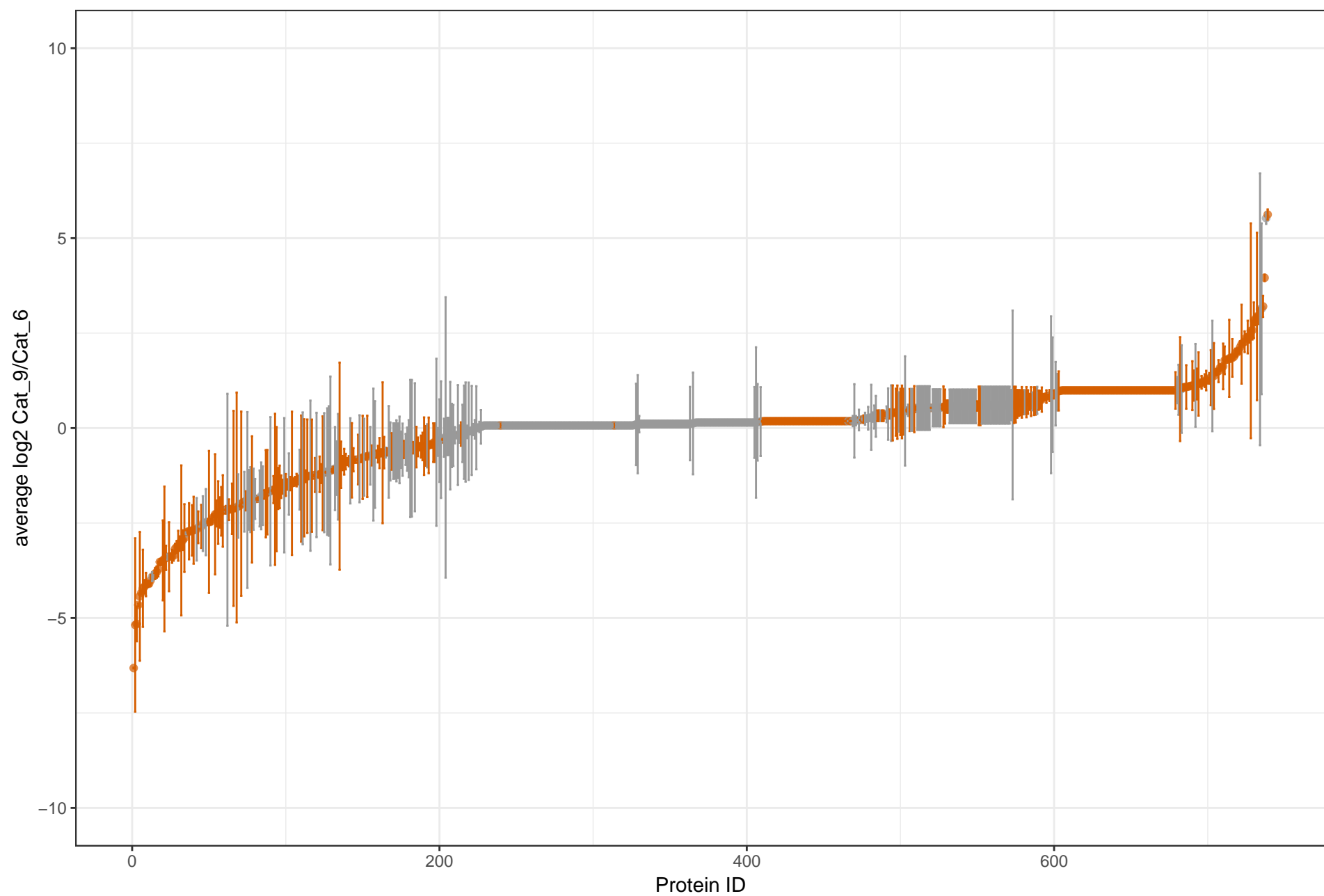

Supplement: Figure 6—source data 1. — Individual data from all figures involving small datasets displayed in individual tabs of this source file. This includes Figures 1B and 2A-F, Figure 3B, Figure 4, Figure 1—figure supplement 1 and Figure 2—figure supplement 1. [file elife-75798-fig6-data1.zip › Flores_Data/AF1_Cat_9.Cat_6-value-ordered-log-ratio_AFCat1.pdf]

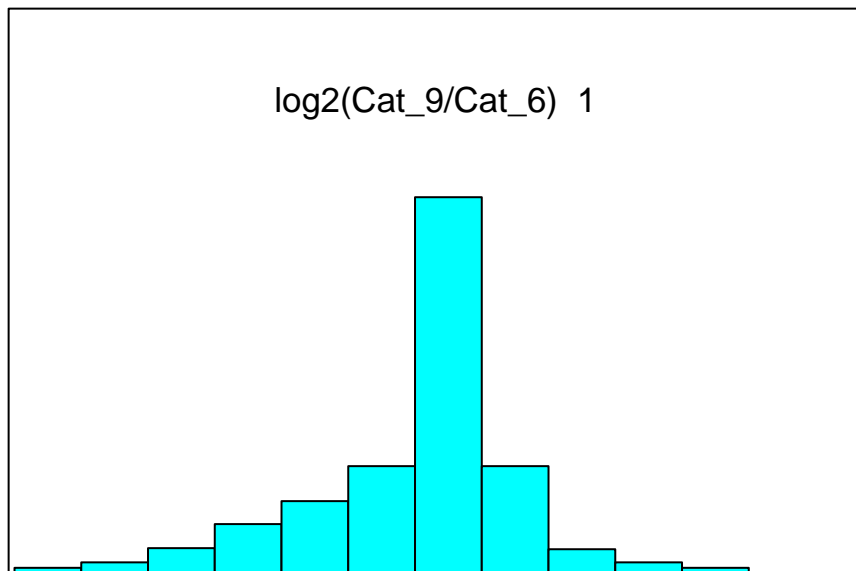

0.72

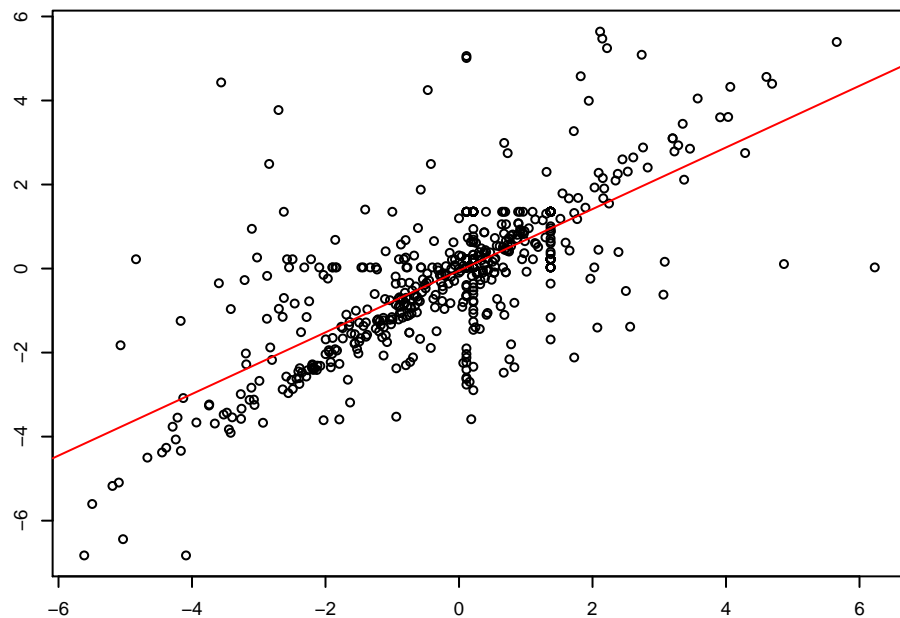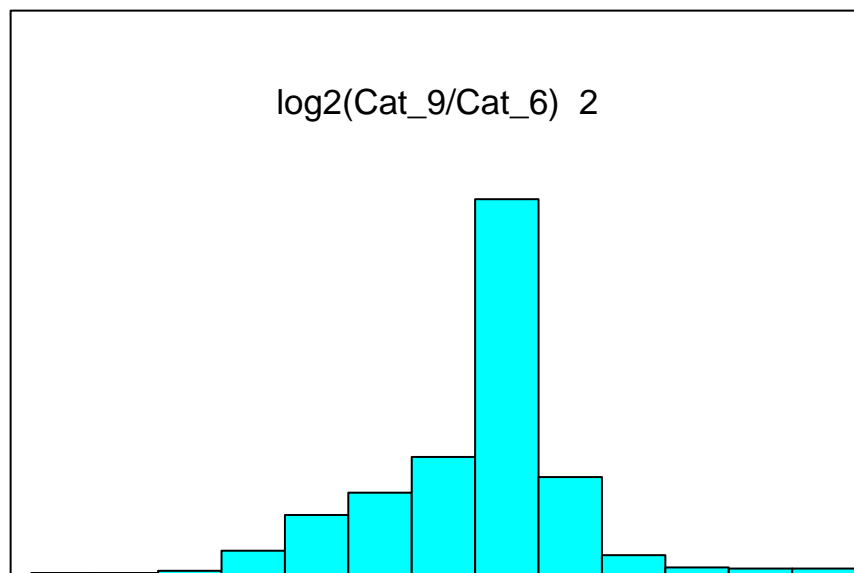

Supplement: Figure 6—source data 1. — Individual data from all figures involving small datasets displayed in individual tabs of this source file. This includes Figures 1B and 2A-F, Figure 3B, Figure 4, Figure 1—figure supplement 1 and Figure 2—figure supplement 1. [file elife-75798-fig6-data1.zip › Flores_Data/AF1_Cat_9.Cat_6-reproducibility_AFCat1.pdf]

MA plot

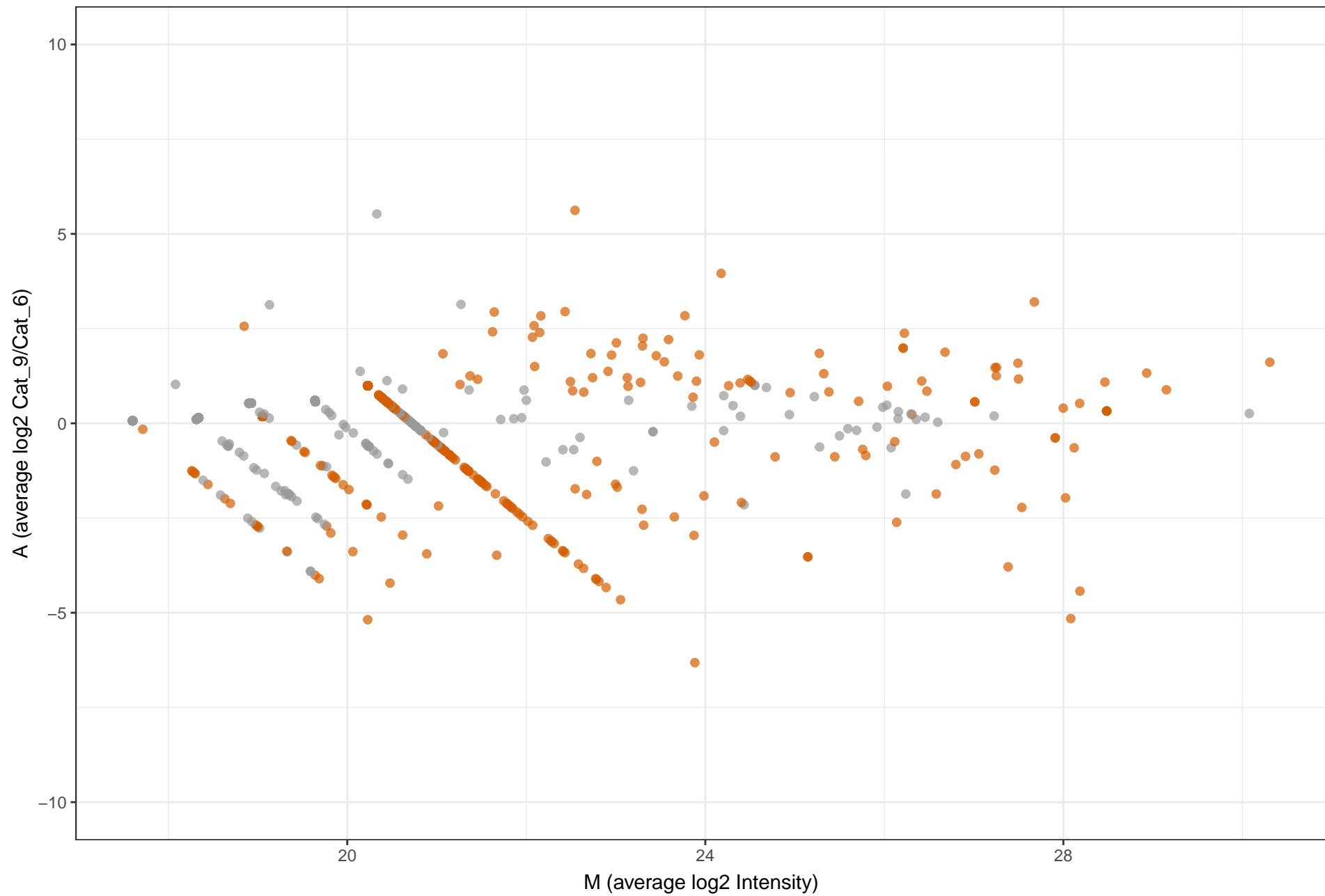

Supplement: Figure 6—source data 1. — Individual data from all figures involving small datasets displayed in individual tabs of this source file. This includes Figures 1B and 2A-F, Figure 3B, Figure 4, Figure 1—figure supplement 1 and Figure 2—figure supplement 1. [file elife-75798-fig6-data1.zip › Flores_Data/AF1_Cat_9.Cat_6-MA_AFCat1.pdf]

P-value vs Fold change

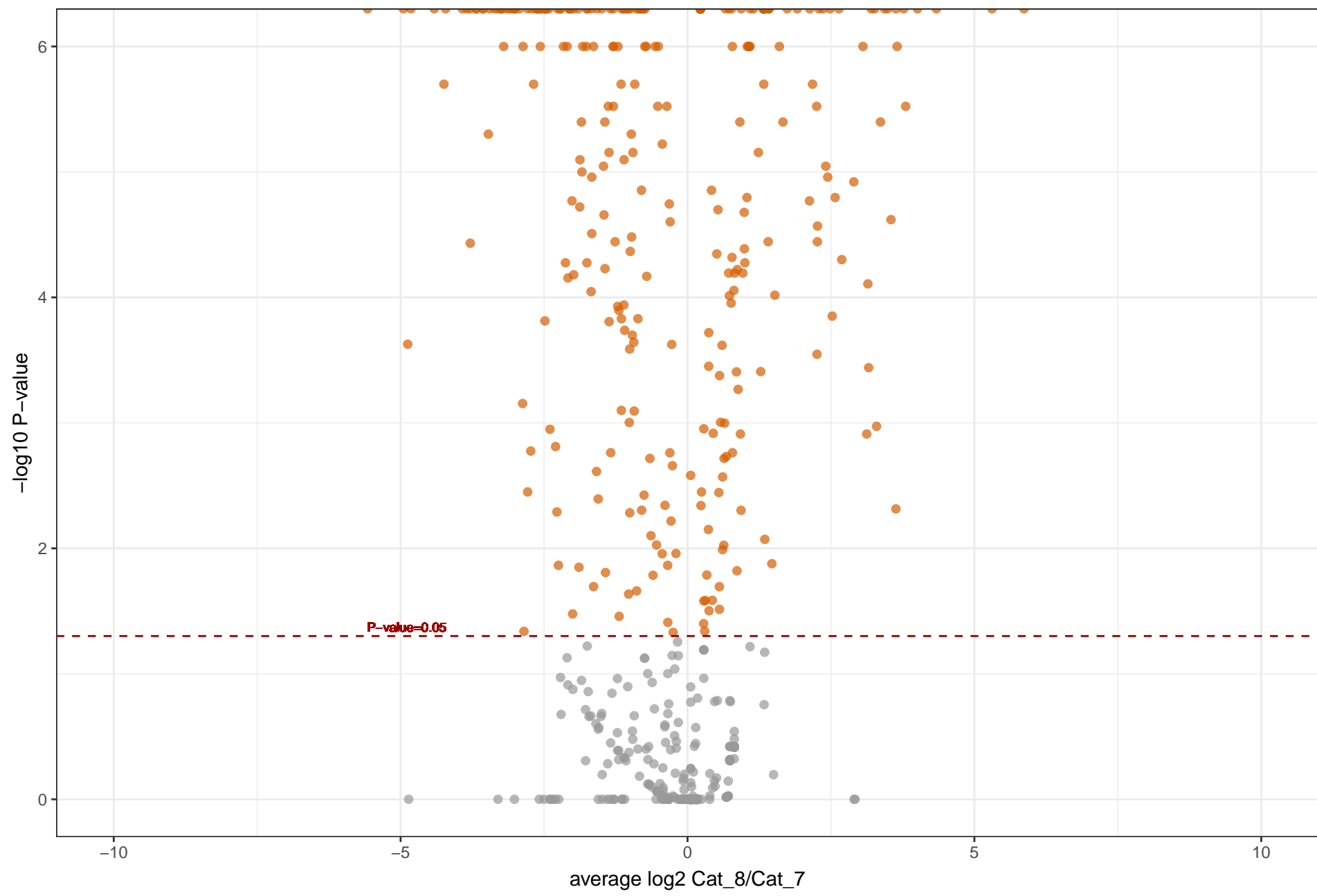

Supplement: Figure 6—source data 1. — Individual data from all figures involving small datasets displayed in individual tabs of this source file. This includes Figures 1B and 2A-F, Figure 3B, Figure 4, Figure 1—figure supplement 1 and Figure 2—figure supplement 1. [file elife-75798-fig6-data1.zip › Flores_Data/AF1_Cat_8.Cat_7-volcano_AFCat1.pdf]

Value-ordered fold change

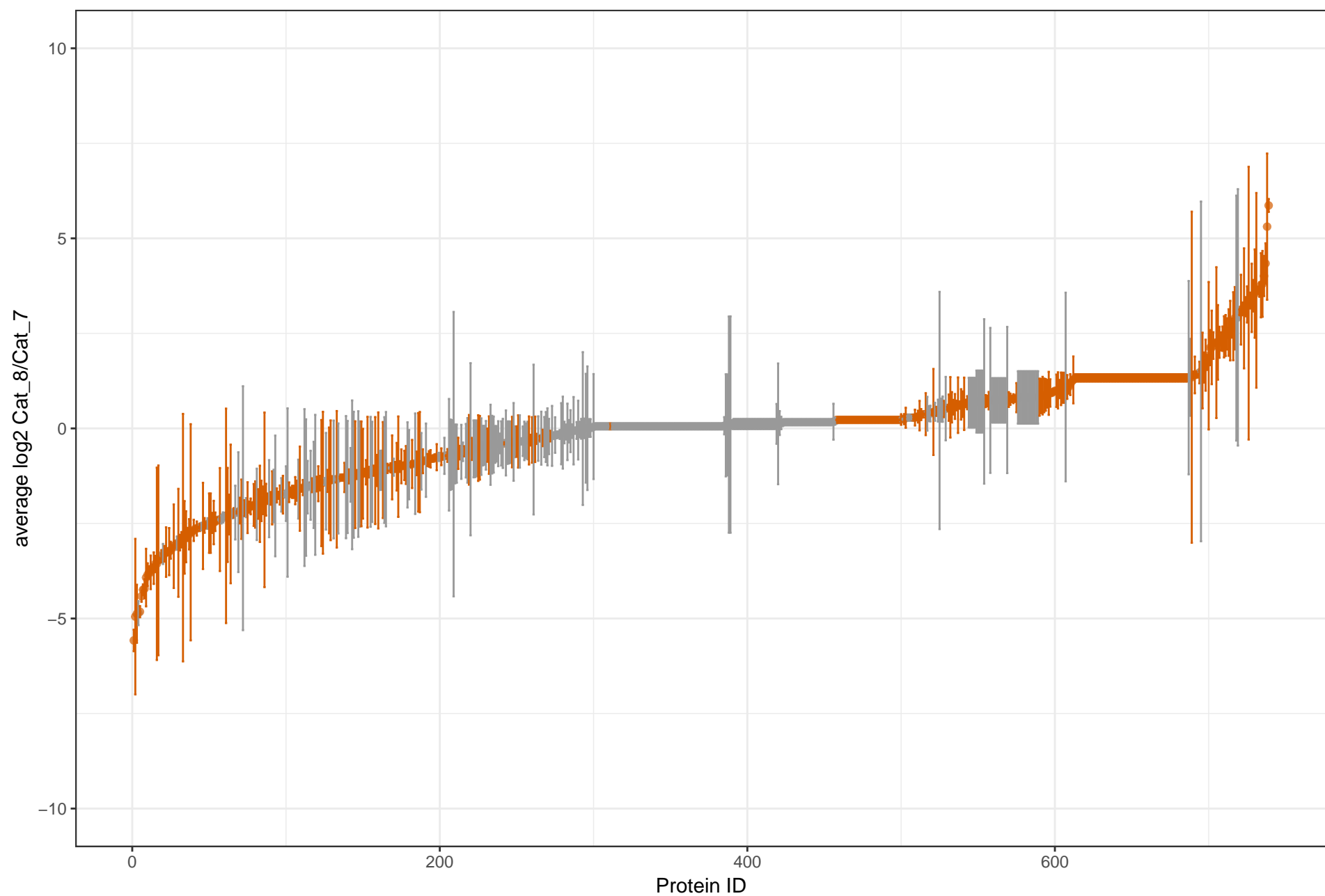

Supplement: Figure 6—source data 1. — Individual data from all figures involving small datasets displayed in individual tabs of this source file. This includes Figures 1B and 2A-F, Figure 3B, Figure 4, Figure 1—figure supplement 1 and Figure 2—figure supplement 1. [file elife-75798-fig6-data1.zip › Flores_Data/AF1_Cat_8.Cat_7-value-ordered-log-ratio_AFCat1.pdf]

MA plot

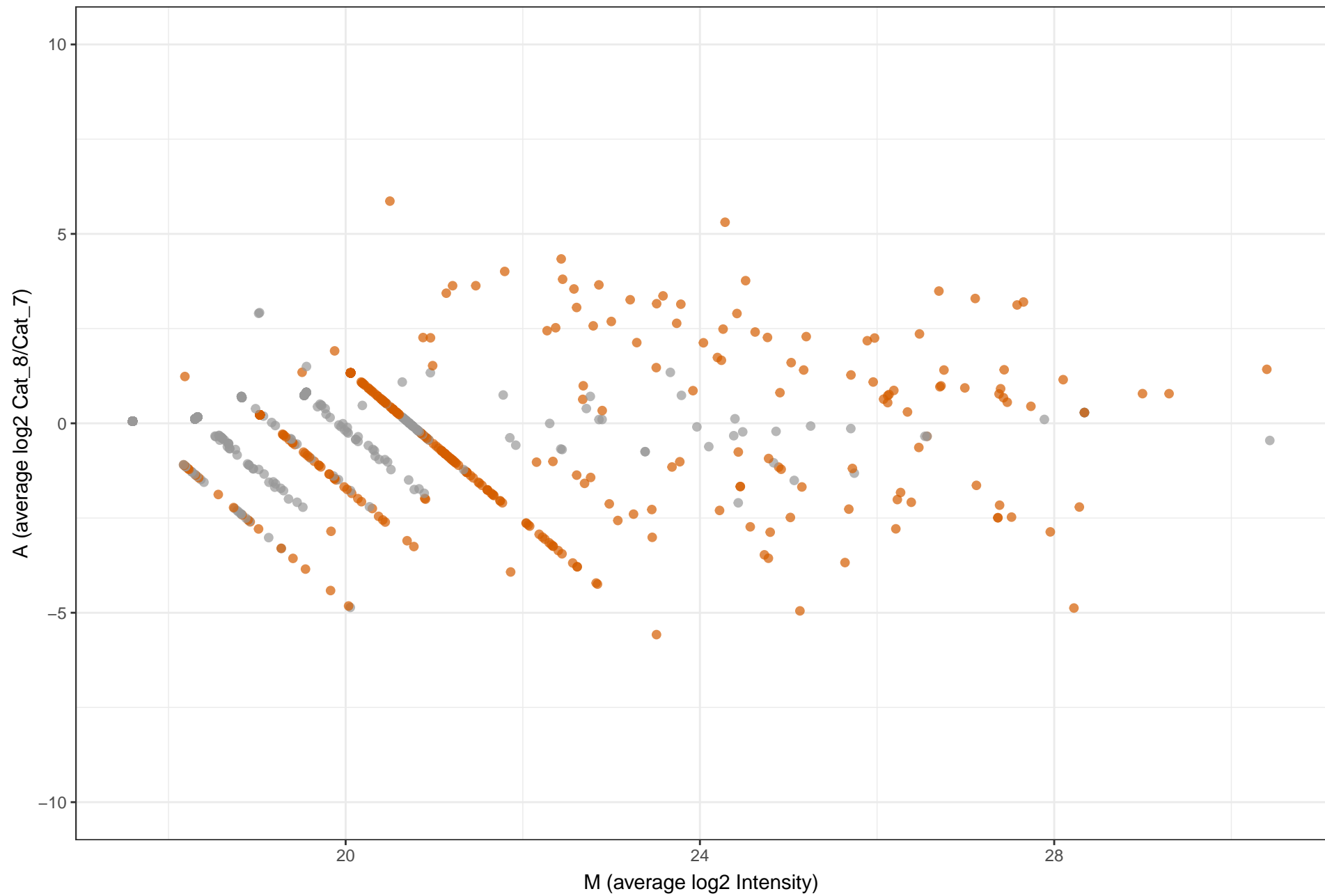

Supplement: Figure 6—source data 1. — Individual data from all figures involving small datasets displayed in individual tabs of this source file. This includes Figures 1B and 2A-F, Figure 3B, Figure 4, Figure 1—figure supplement 1 and Figure 2—figure supplement 1. [file elife-75798-fig6-data1.zip › Flores_Data/AF1_Cat_8.Cat_7-MA_AFCat1.pdf]

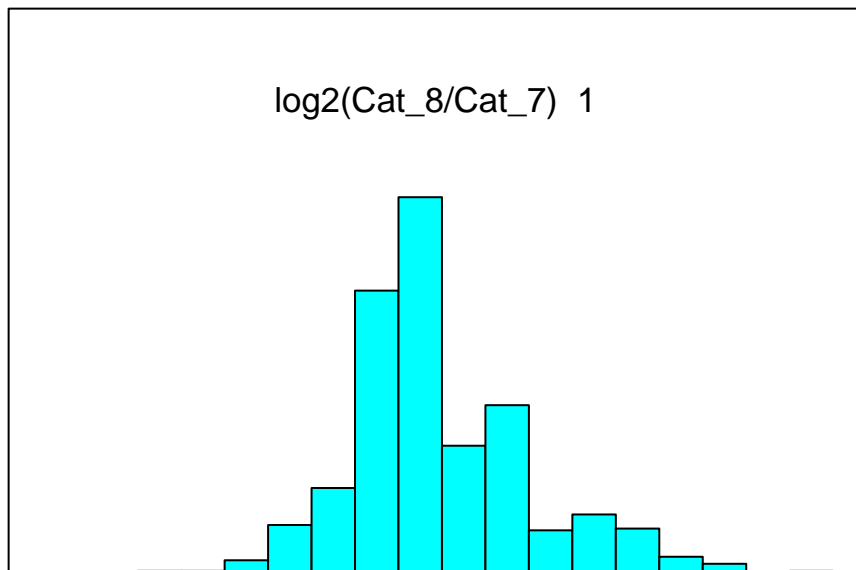

-5 0 5

5  
0  
-5

0.85

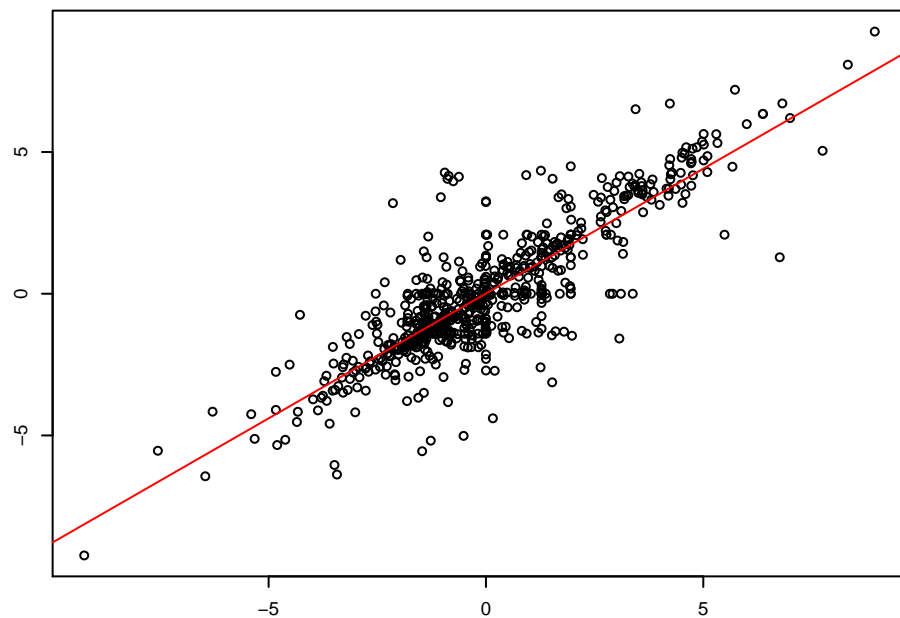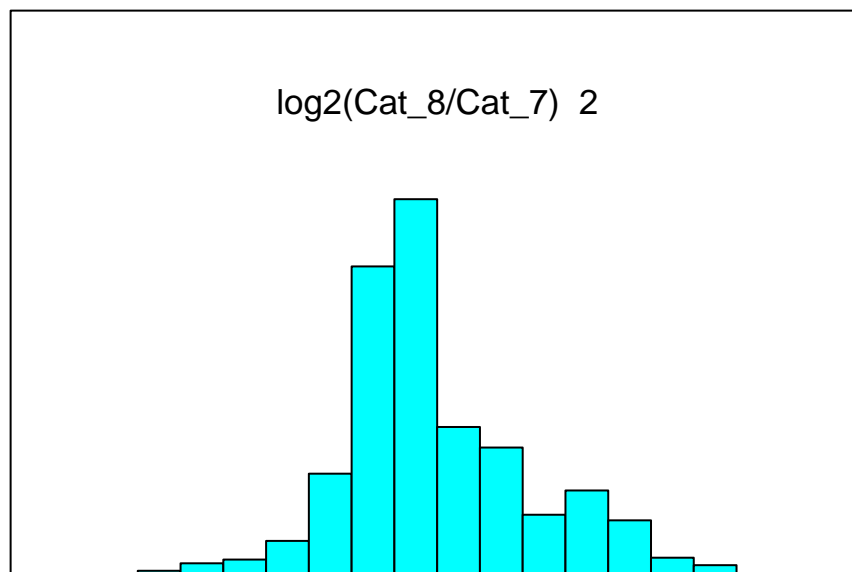

Supplement: Figure 6—source data 1. — Individual data from all figures involving small datasets displayed in individual tabs of this source file. This includes Figures 1B and 2A-F, Figure 3B, Figure 4, Figure 1—figure supplement 1 and Figure 2—figure supplement 1. [file elife-75798-fig6-data1.zip › Flores_Data/AF1_Cat_8.Cat_7-reproducibility_AFCat1.pdf]

P-value vs Fold change

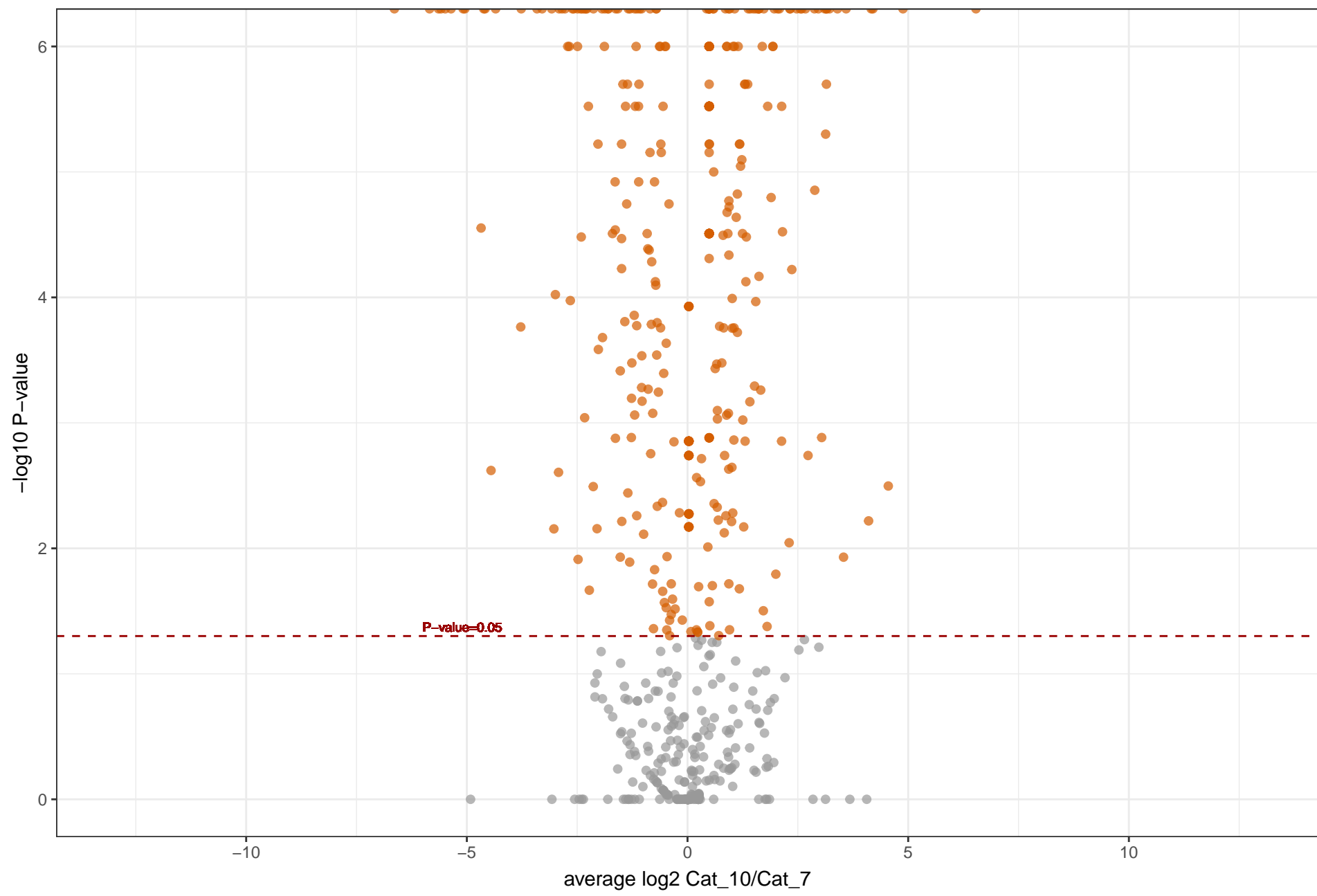

Supplement: Figure 6—source data 1. — Individual data from all figures involving small datasets displayed in individual tabs of this source file. This includes Figures 1B and 2A-F, Figure 3B, Figure 4, Figure 1—figure supplement 1 and Figure 2—figure supplement 1. [file elife-75798-fig6-data1.zip › Flores_Data/AF1_Cat_10.Cat_7-volcano_AFCat1.pdf]

Value-ordered fold change

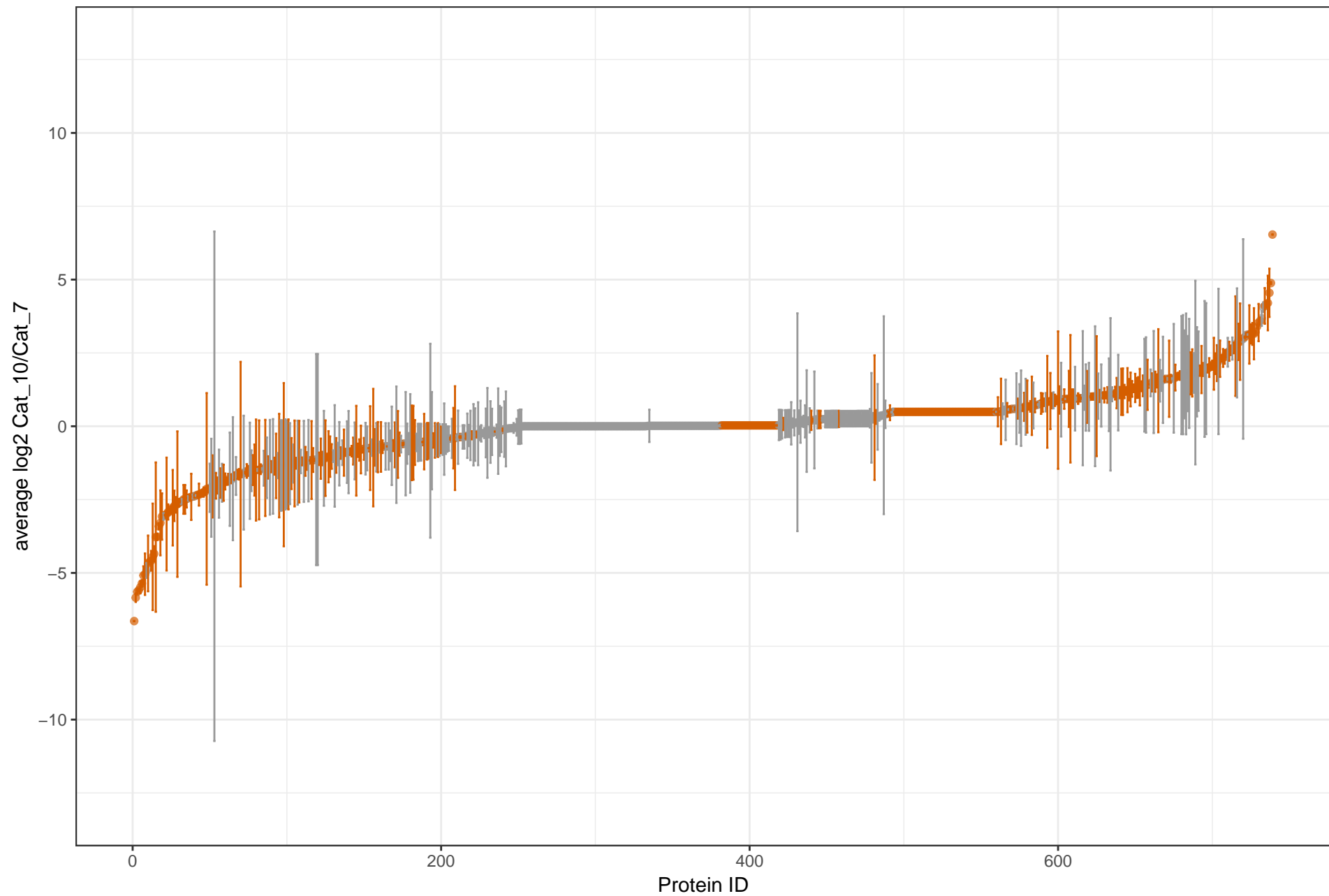

Supplement: Figure 6—source data 1. — Individual data from all figures involving small datasets displayed in individual tabs of this source file. This includes Figures 1B and 2A-F, Figure 3B, Figure 4, Figure 1—figure supplement 1 and Figure 2—figure supplement 1. [file elife-75798-fig6-data1.zip › Flores_Data/AF1_Cat_10.Cat_7-value-ordered-log-ratio_AFCat1.pdf]

MA plot

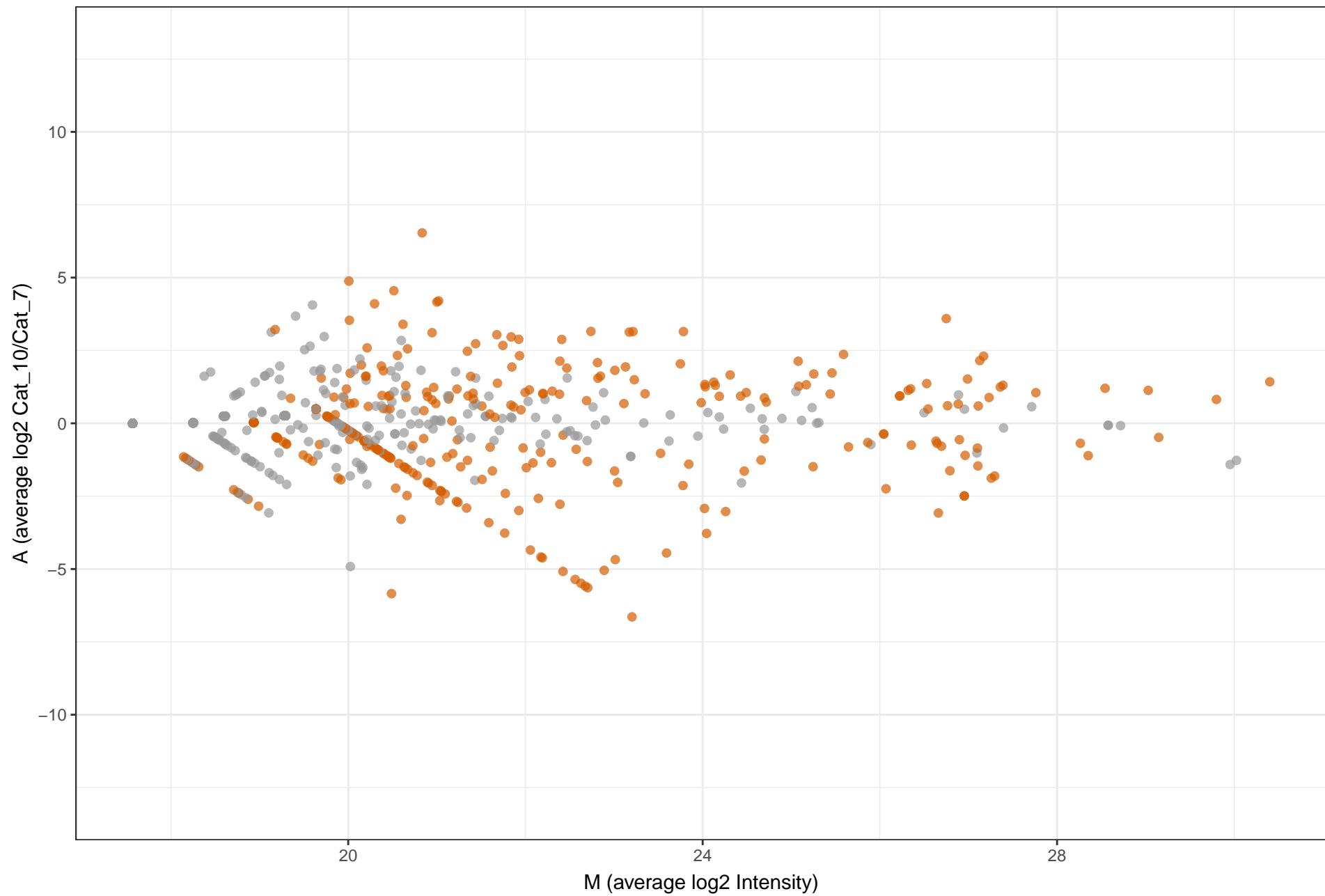

Supplement: Figure 6—source data 1. — Individual data from all figures involving small datasets displayed in individual tabs of this source file. This includes Figures 1B and 2A-F, Figure 3B, Figure 4, Figure 1—figure supplement 1 and Figure 2—figure supplement 1. [file elife-75798-fig6-data1.zip › Flores_Data/AF1_Cat_10.Cat_7-MA_AFCat1.pdf]

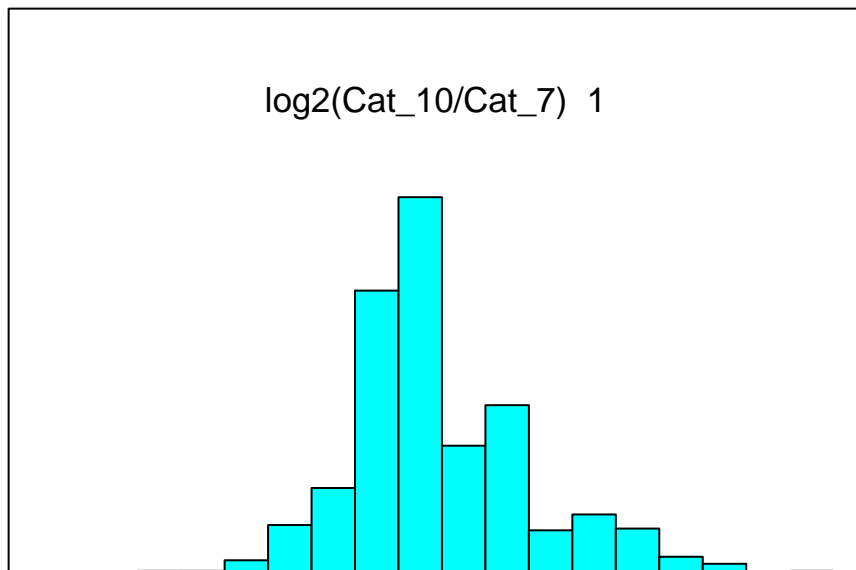

-5 0 5

5  
0  
-5

0.85

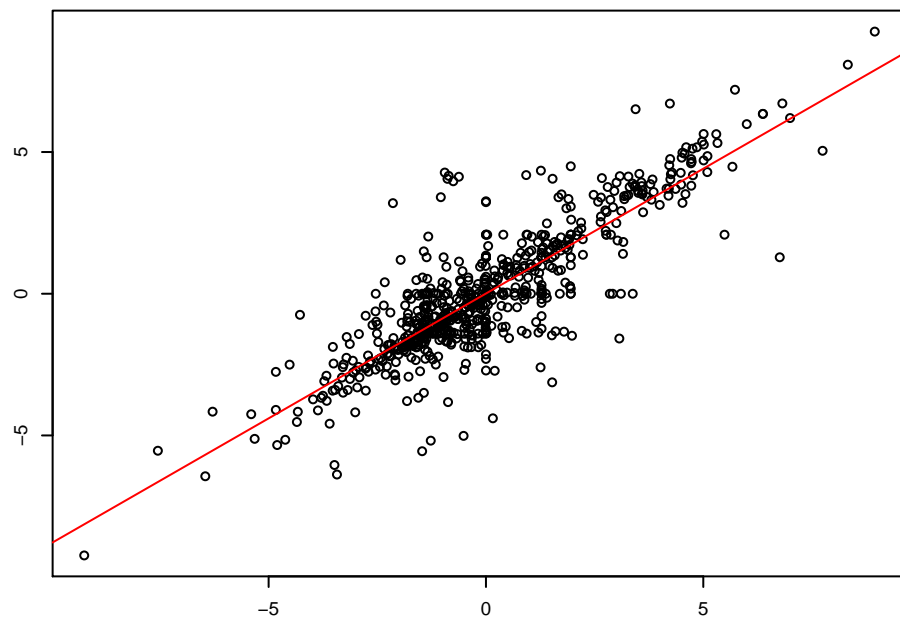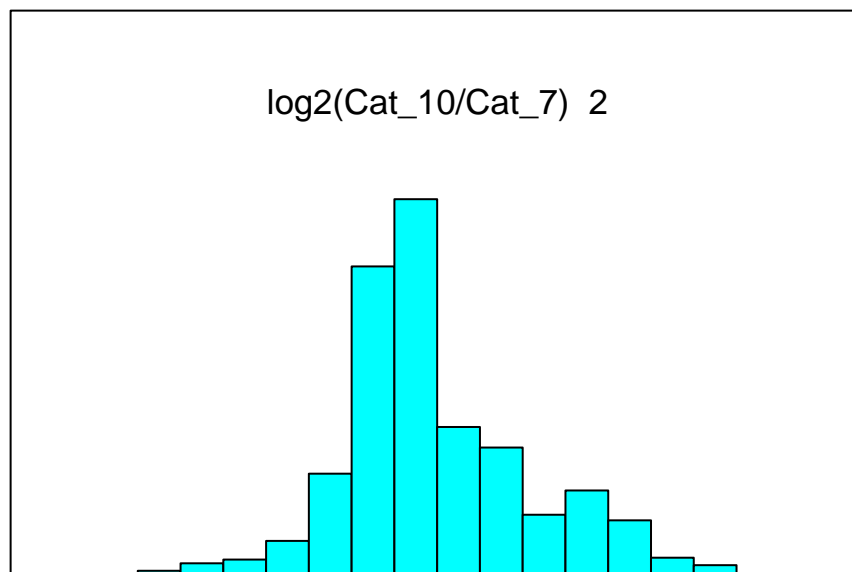

Supplement: Figure 6—source data 1. — Individual data from all figures involving small datasets displayed in individual tabs of this source file. This includes Figures 1B and 2A-F, Figure 3B, Figure 4, Figure 1—figure supplement 1 and Figure 2—figure supplement 1. [file elife-75798-fig6-data1.zip › Flores_Data/AF1_Cat_10.Cat_7-reproducibility_AFCat1.pdf]

P-value vs Fold change

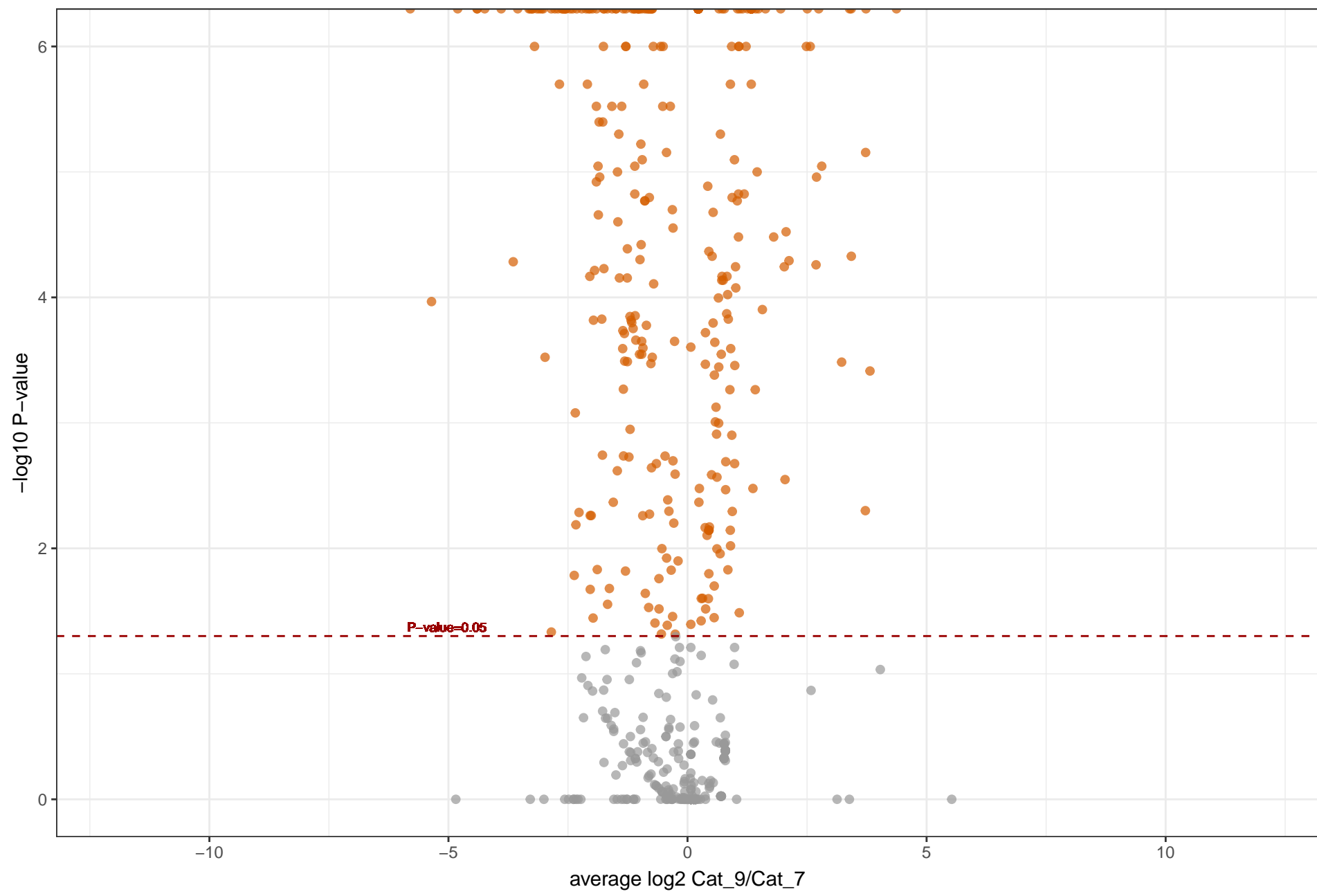

Supplement: Figure 6—source data 1. — Individual data from all figures involving small datasets displayed in individual tabs of this source file. This includes Figures 1B and 2A-F, Figure 3B, Figure 4, Figure 1—figure supplement 1 and Figure 2—figure supplement 1. [file elife-75798-fig6-data1.zip › Flores_Data/AF1_Cat_9.Cat_7-volcano_AFCat1.pdf]

Value-ordered fold change

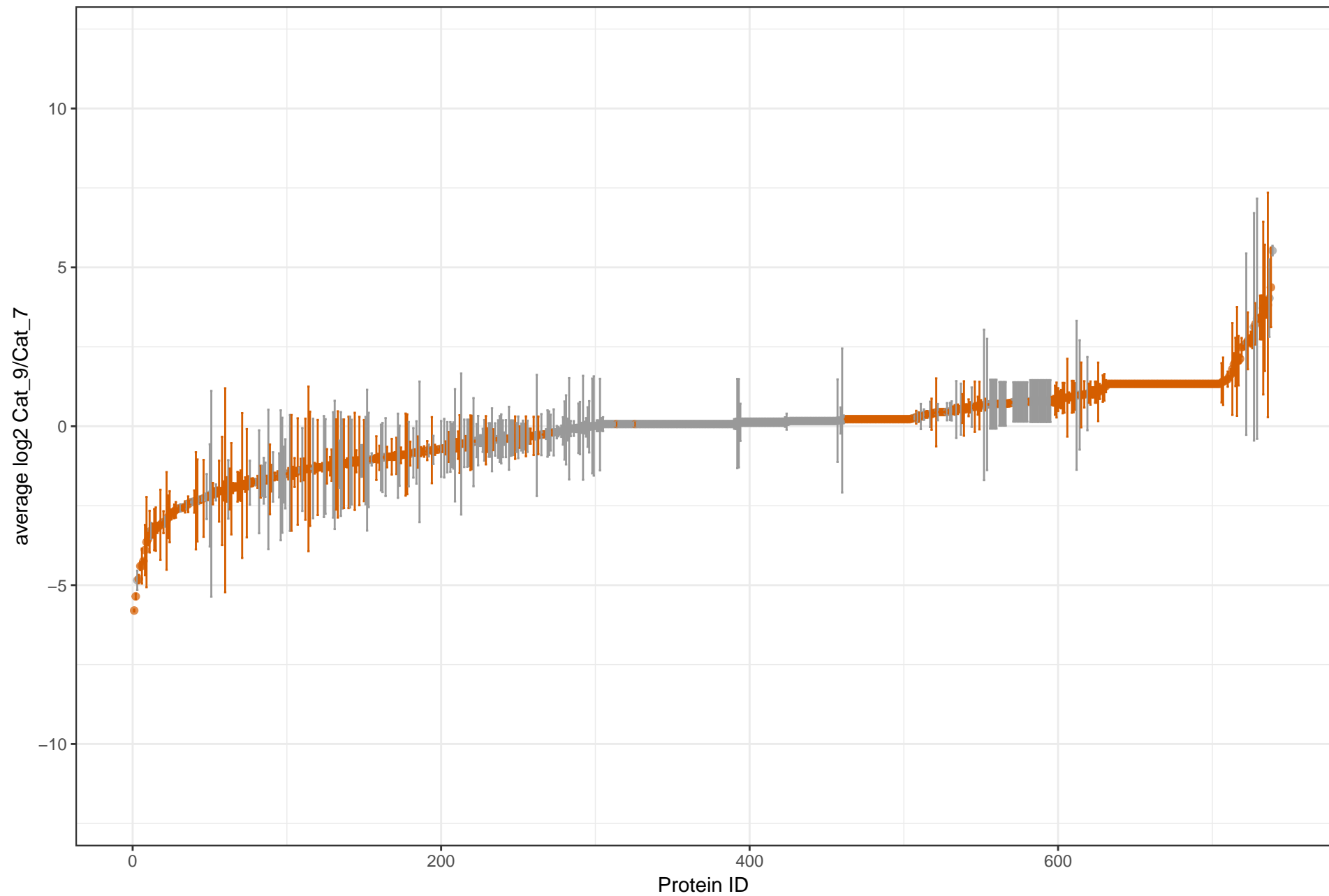

Supplement: Figure 6—source data 1. — Individual data from all figures involving small datasets displayed in individual tabs of this source file. This includes Figures 1B and 2A-F, Figure 3B, Figure 4, Figure 1—figure supplement 1 and Figure 2—figure supplement 1. [file elife-75798-fig6-data1.zip › Flores_Data/AF1_Cat_9.Cat_7-value-ordered-log-ratio_AFCat1.pdf]

MA plot

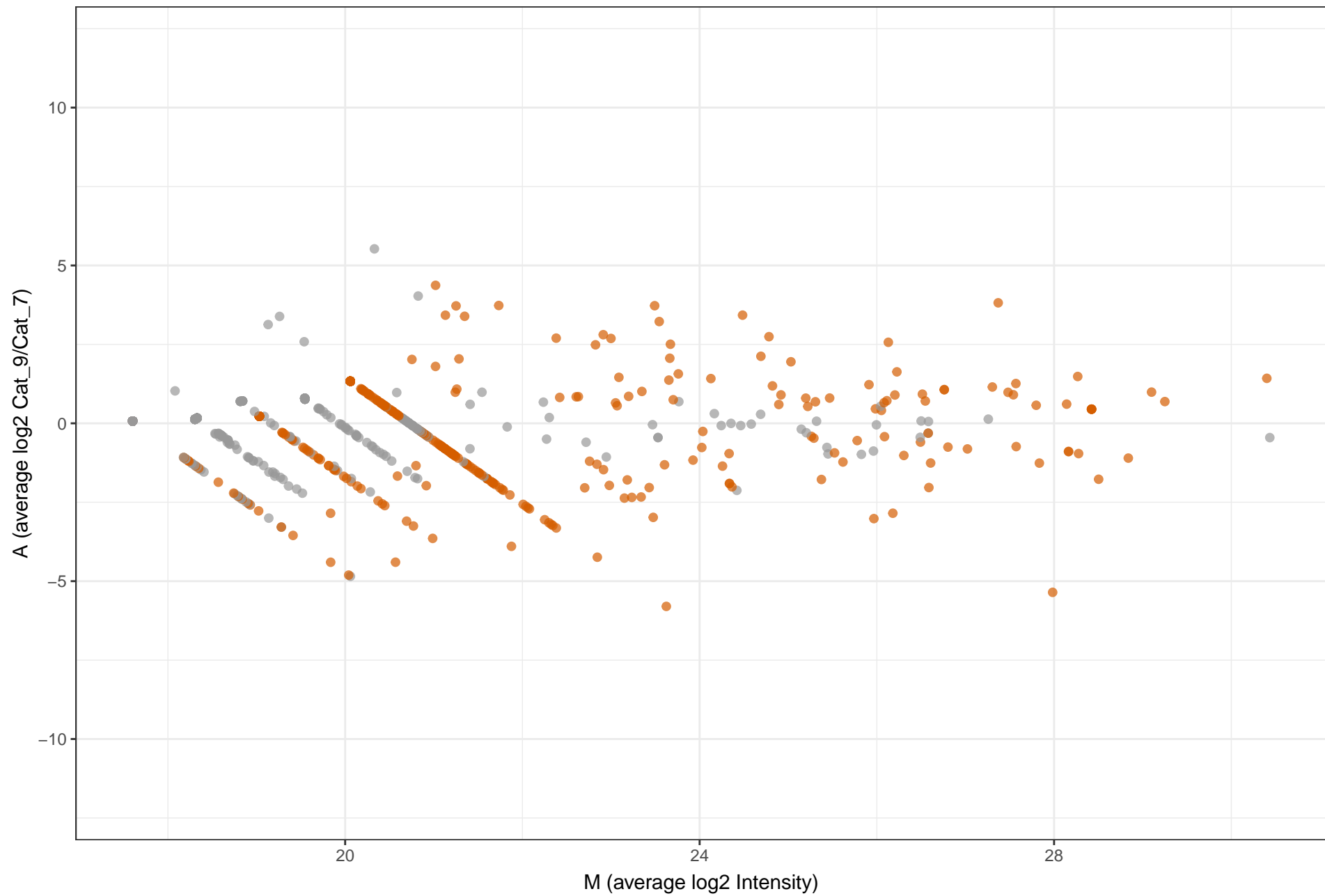

Supplement: Figure 6—source data 1. — Individual data from all figures involving small datasets displayed in individual tabs of this source file. This includes Figures 1B and 2A-F, Figure 3B, Figure 4, Figure 1—figure supplement 1 and Figure 2—figure supplement 1. [file elife-75798-fig6-data1.zip › Flores_Data/AF1_Cat_9.Cat_7-MA_AFCat1.pdf]

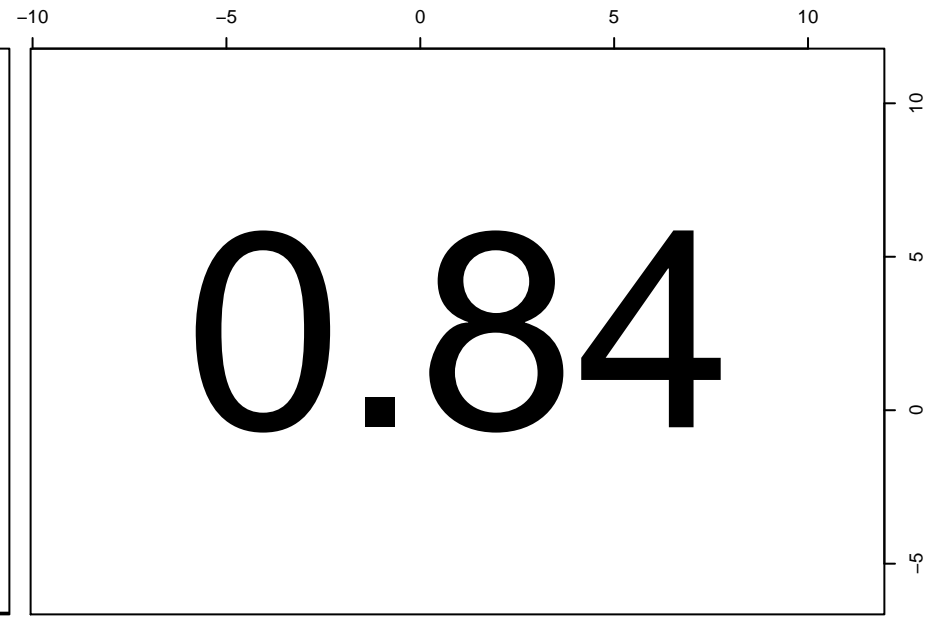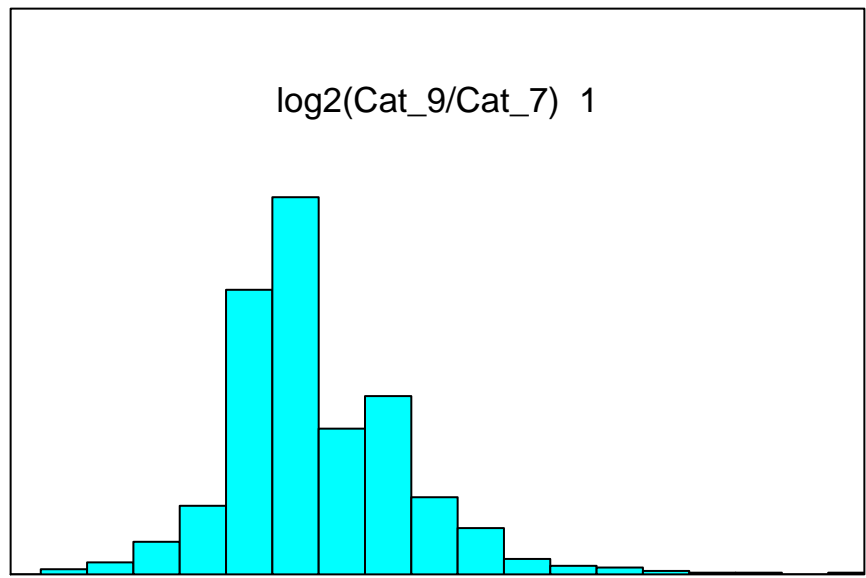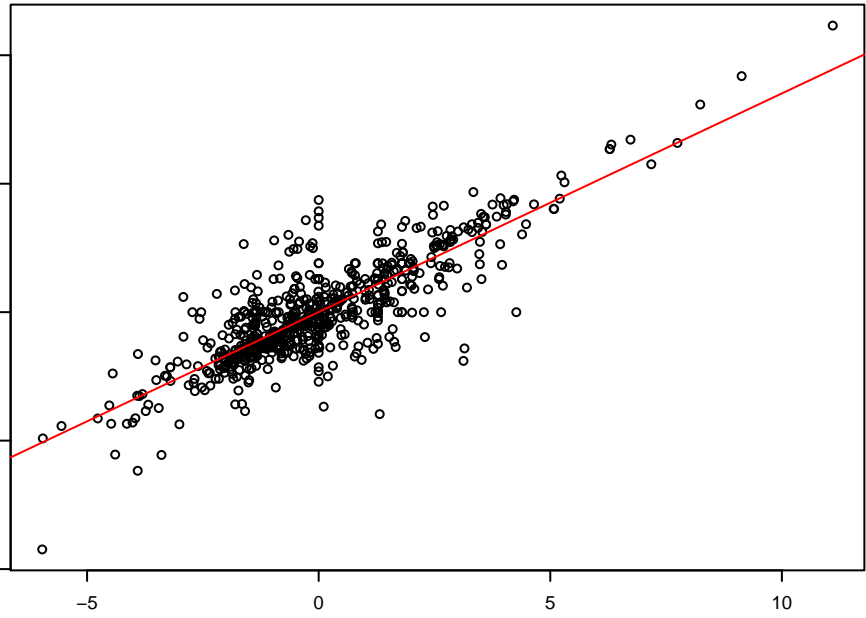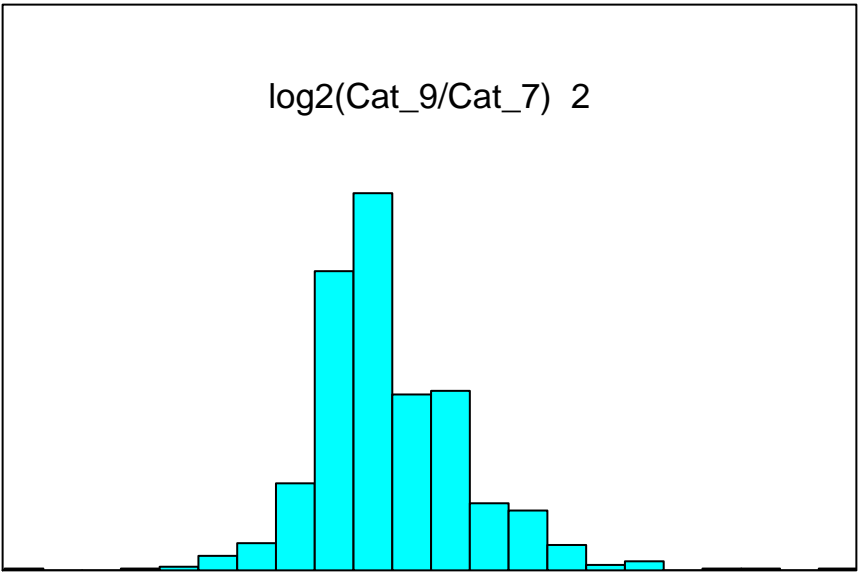

Supplement: Figure 6—source data 1. — Individual data from all figures involving small datasets displayed in individual tabs of this source file. This includes Figures 1B and 2A-F, Figure 3B, Figure 4, Figure 1—figure supplement 1 and Figure 2—figure supplement 1. [file elife-75798-fig6-data1.zip › Flores_Data/AF1_Cat_9.Cat_7-reproducibility_AFCat1.pdf]

**P-value vs Fold change**

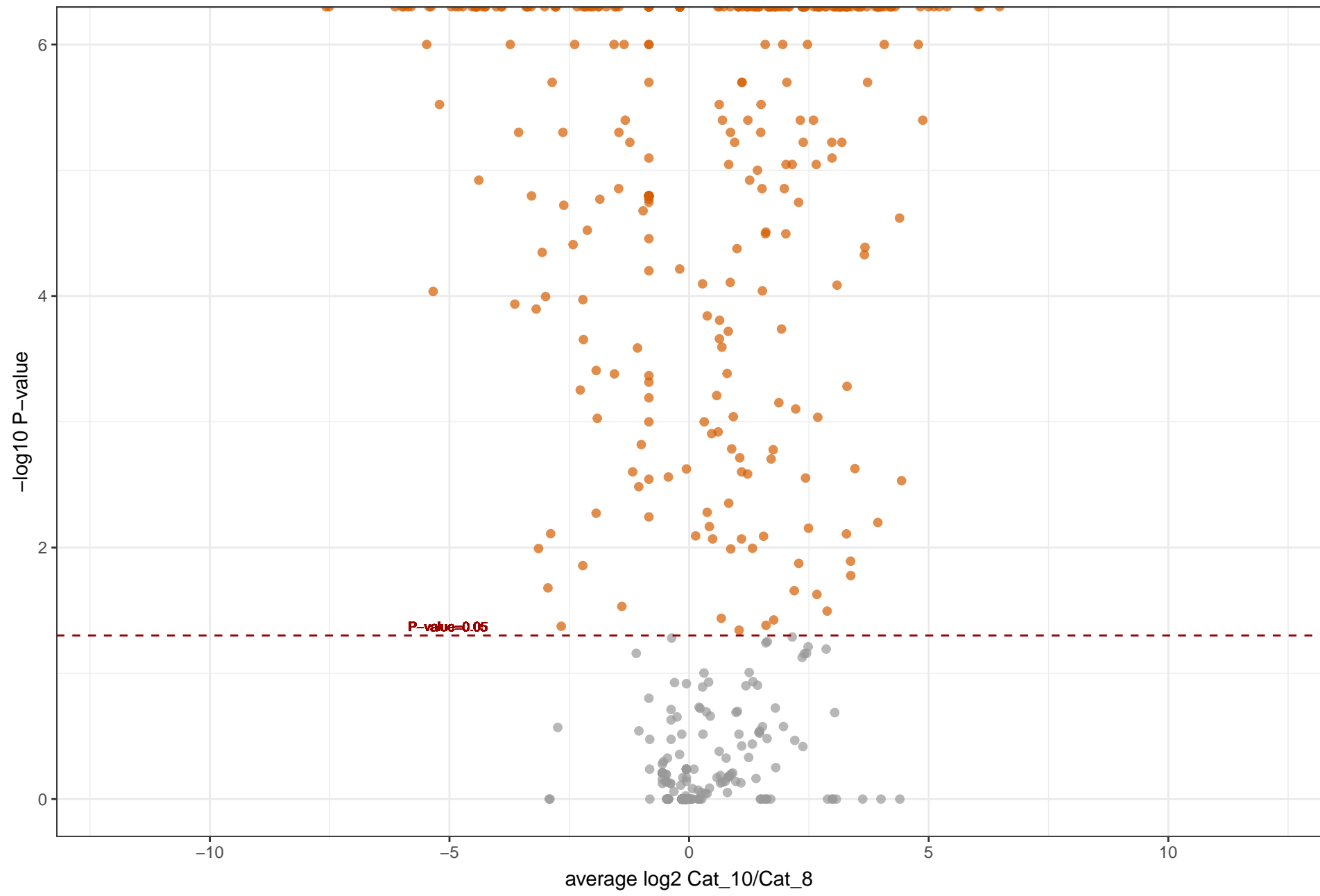

Supplement: Figure 6—source data 1. — Individual data from all figures involving small datasets displayed in individual tabs of this source file. This includes Figures 1B and 2A-F, Figure 3B, Figure 4, Figure 1—figure supplement 1 and Figure 2—figure supplement 1. [file elife-75798-fig6-data1.zip › Flores_Data/AF1_Cat_10.Cat_8-volcano_AFCat1.pdf]

Value-ordered fold change

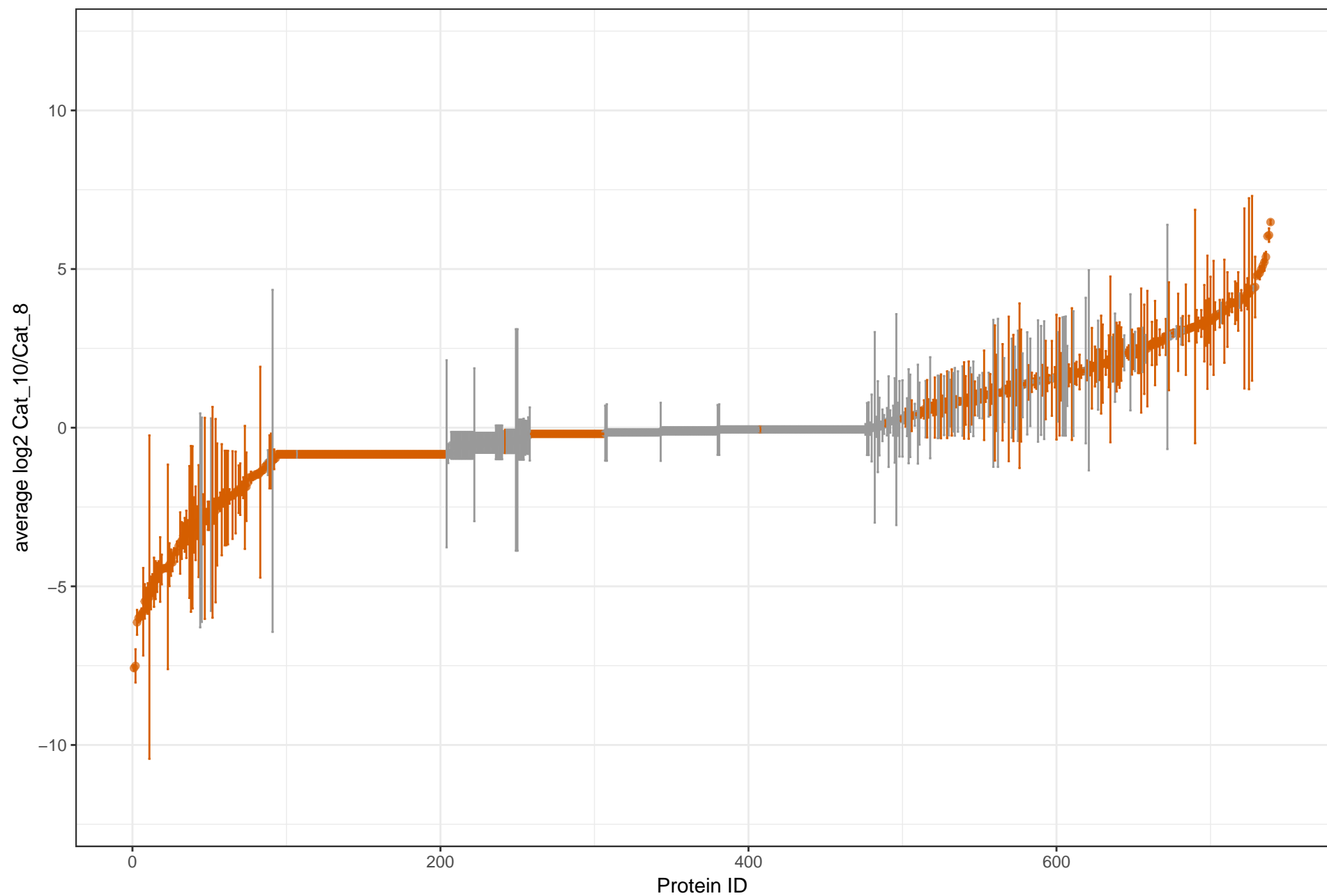

Supplement: Figure 6—source data 1. — Individual data from all figures involving small datasets displayed in individual tabs of this source file. This includes Figures 1B and 2A-F, Figure 3B, Figure 4, Figure 1—figure supplement 1 and Figure 2—figure supplement 1. [file elife-75798-fig6-data1.zip › Flores_Data/AF1_Cat_10.Cat_8-value-ordered-log-ratio_AFCat1.pdf]

MA plot

A (average log2 Cat\_10/Cat\_8)

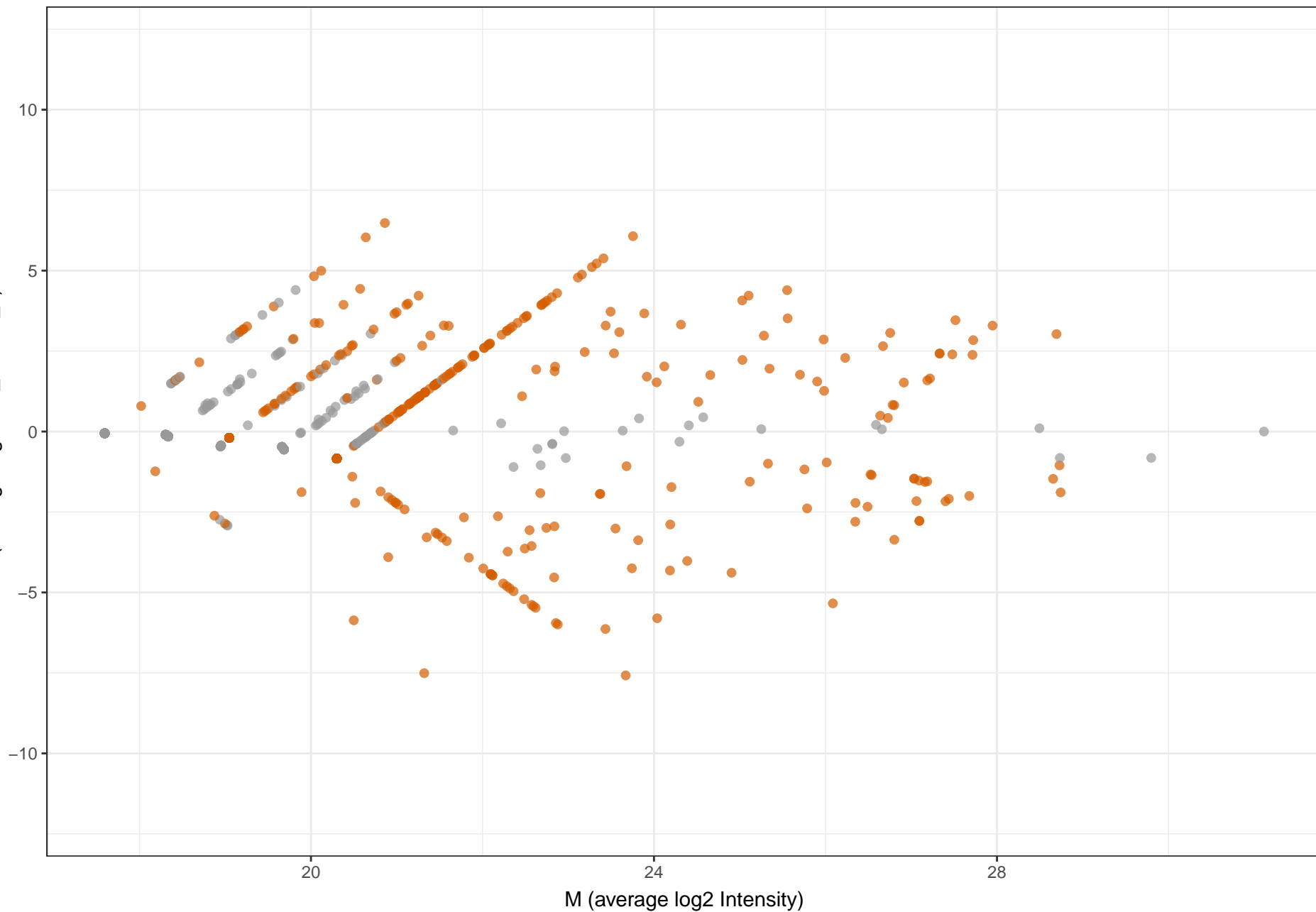

Supplement: Figure 6—source data 1. — Individual data from all figures involving small datasets displayed in individual tabs of this source file. This includes Figures 1B and 2A-F, Figure 3B, Figure 4, Figure 1—figure supplement 1 and Figure 2—figure supplement 1. [file elife-75798-fig6-data1.zip › Flores_Data/AF1_Cat_10.Cat_8-MA_AFCat1.pdf]

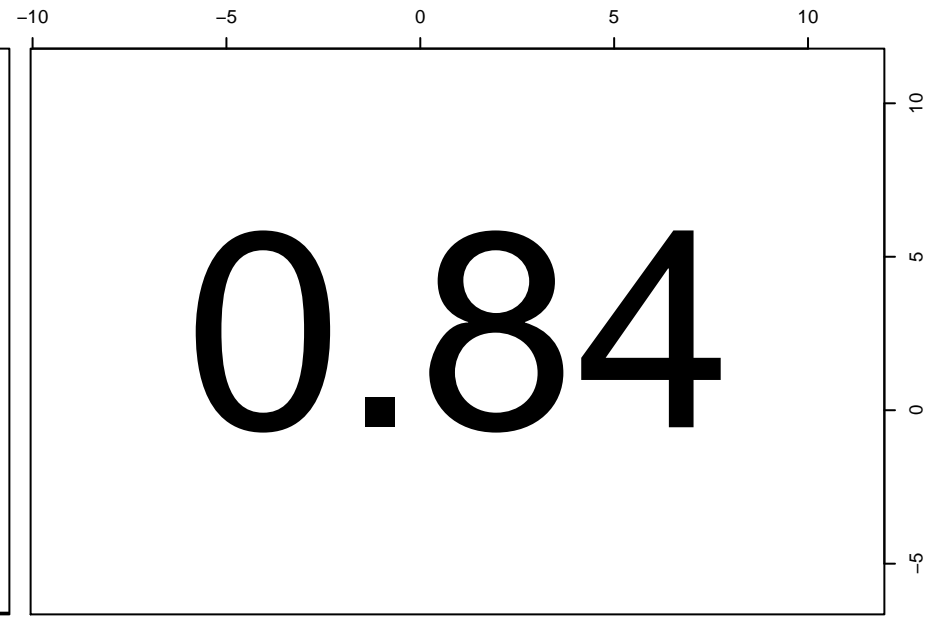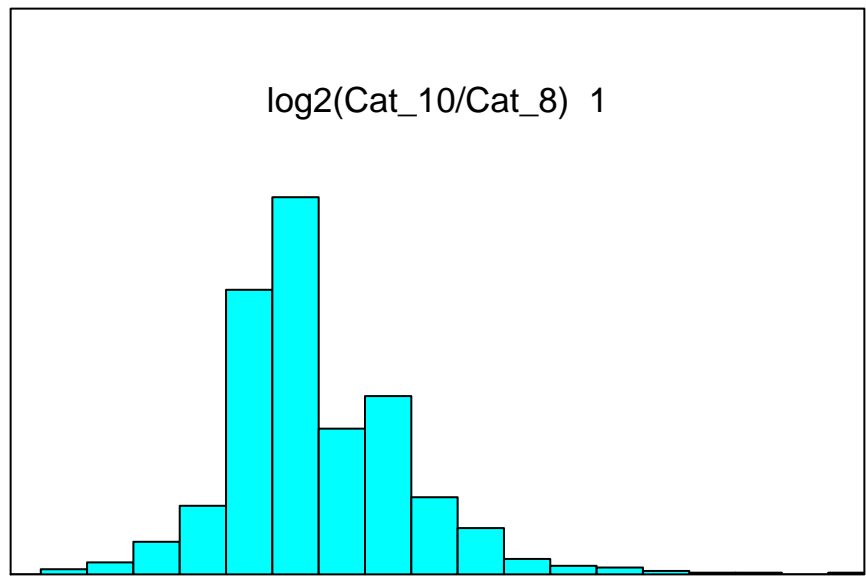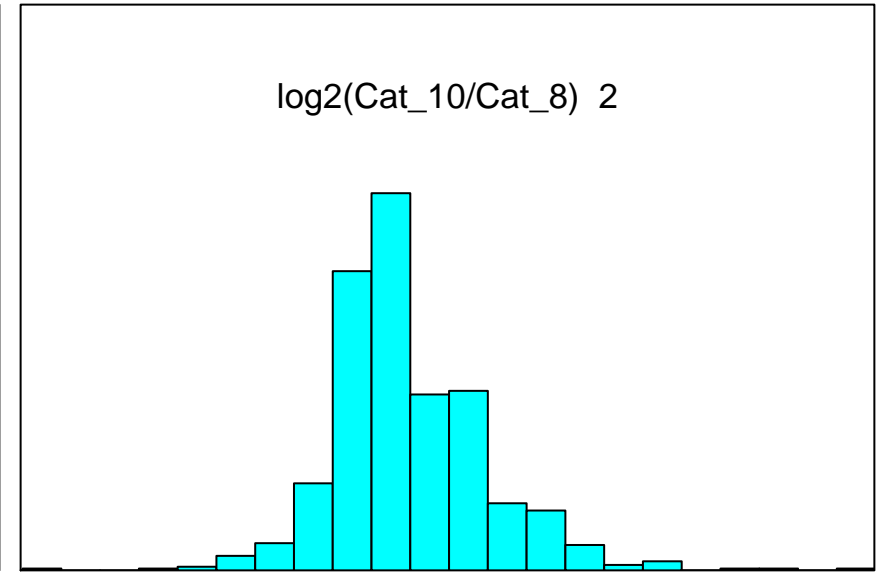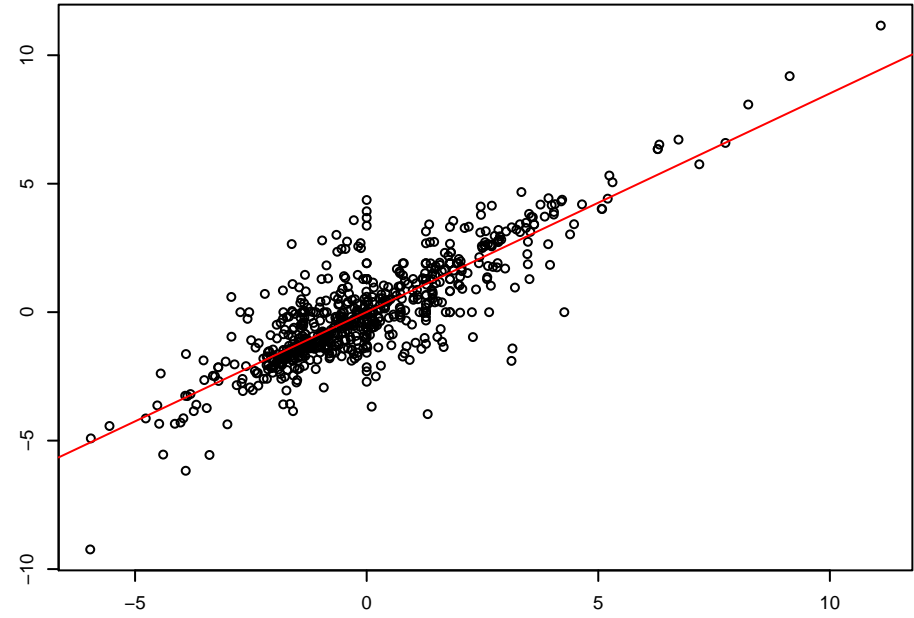

Supplement: Figure 6—source data 1. — Individual data from all figures involving small datasets displayed in individual tabs of this source file. This includes Figures 1B and 2A-F, Figure 3B, Figure 4, Figure 1—figure supplement 1 and Figure 2—figure supplement 1. [file elife-75798-fig6-data1.zip › Flores_Data/AF1_Cat_10.Cat_8-reproducibility_AFCat1.pdf]

**P-value vs Fold change**

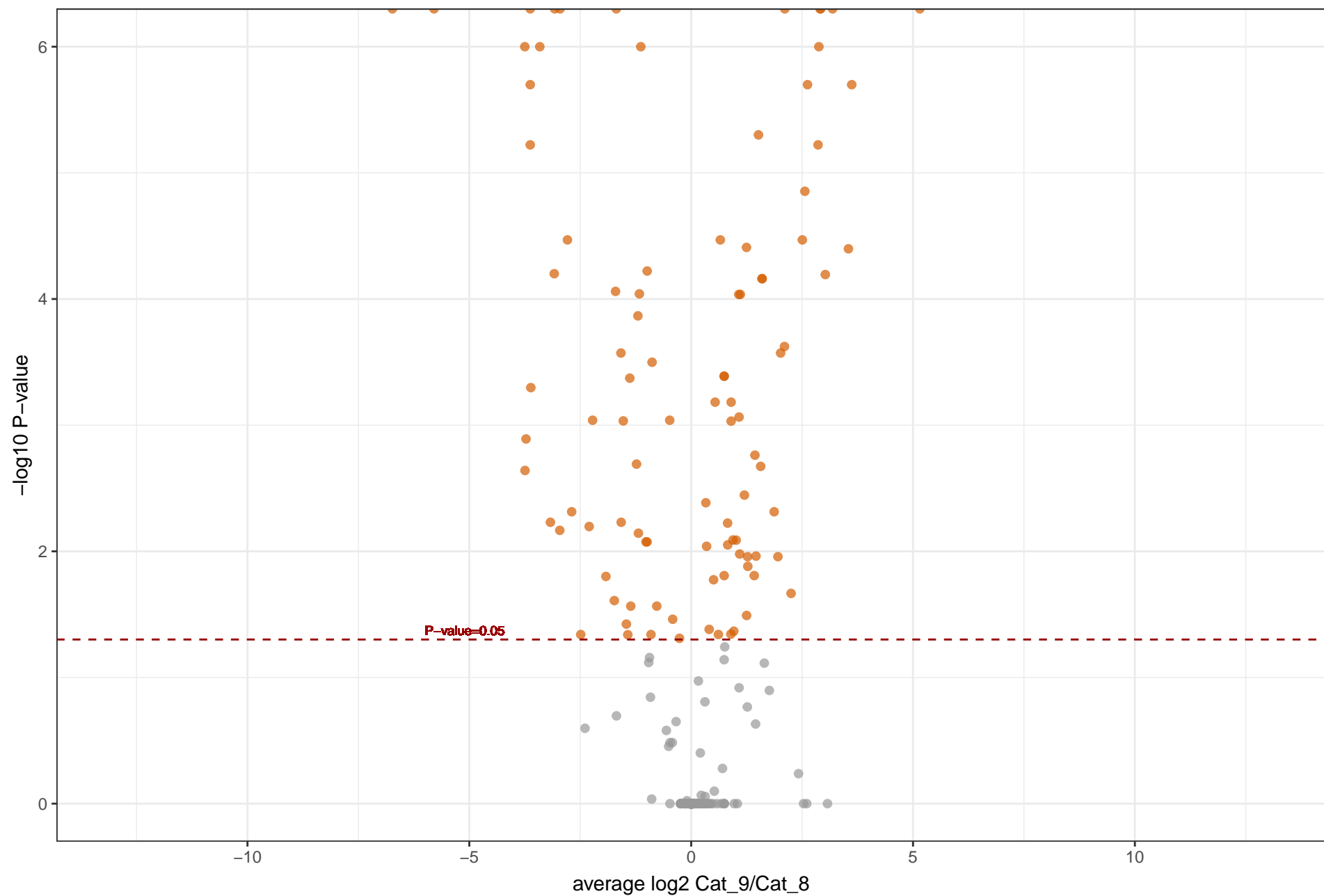

Supplement: Figure 6—source data 1. — Individual data from all figures involving small datasets displayed in individual tabs of this source file. This includes Figures 1B and 2A-F, Figure 3B, Figure 4, Figure 1—figure supplement 1 and Figure 2—figure supplement 1. [file elife-75798-fig6-data1.zip › Flores_Data/AF1_Cat_9.Cat_8-volcano_AFCat1.pdf]

Value-ordered fold change

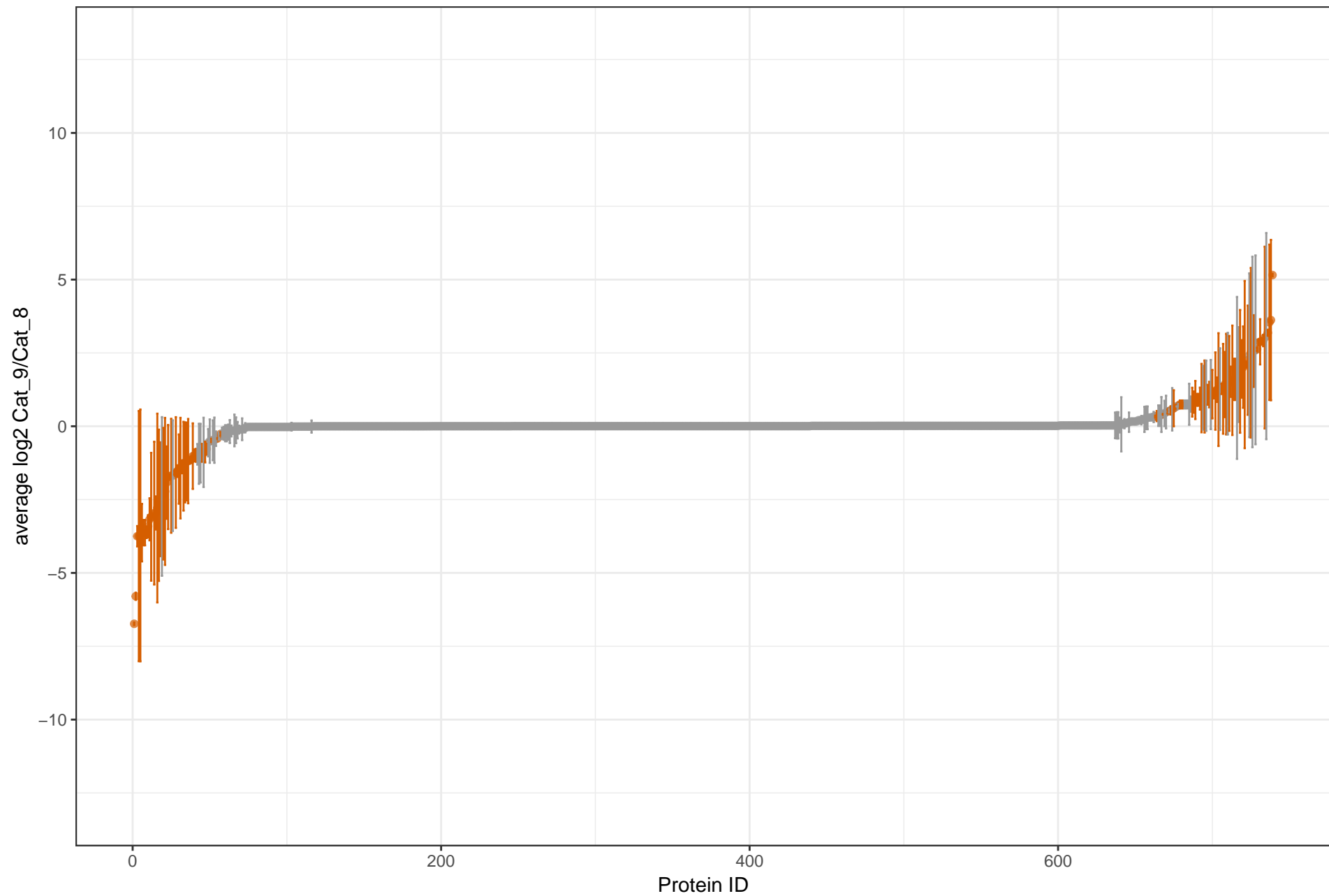

Supplement: Figure 6—source data 1. — Individual data from all figures involving small datasets displayed in individual tabs of this source file. This includes Figures 1B and 2A-F, Figure 3B, Figure 4, Figure 1—figure supplement 1 and Figure 2—figure supplement 1. [file elife-75798-fig6-data1.zip › Flores_Data/AF1_Cat_9.Cat_8-value-ordered-log-ratio_AFCat1.pdf]

P-value vs Fold change

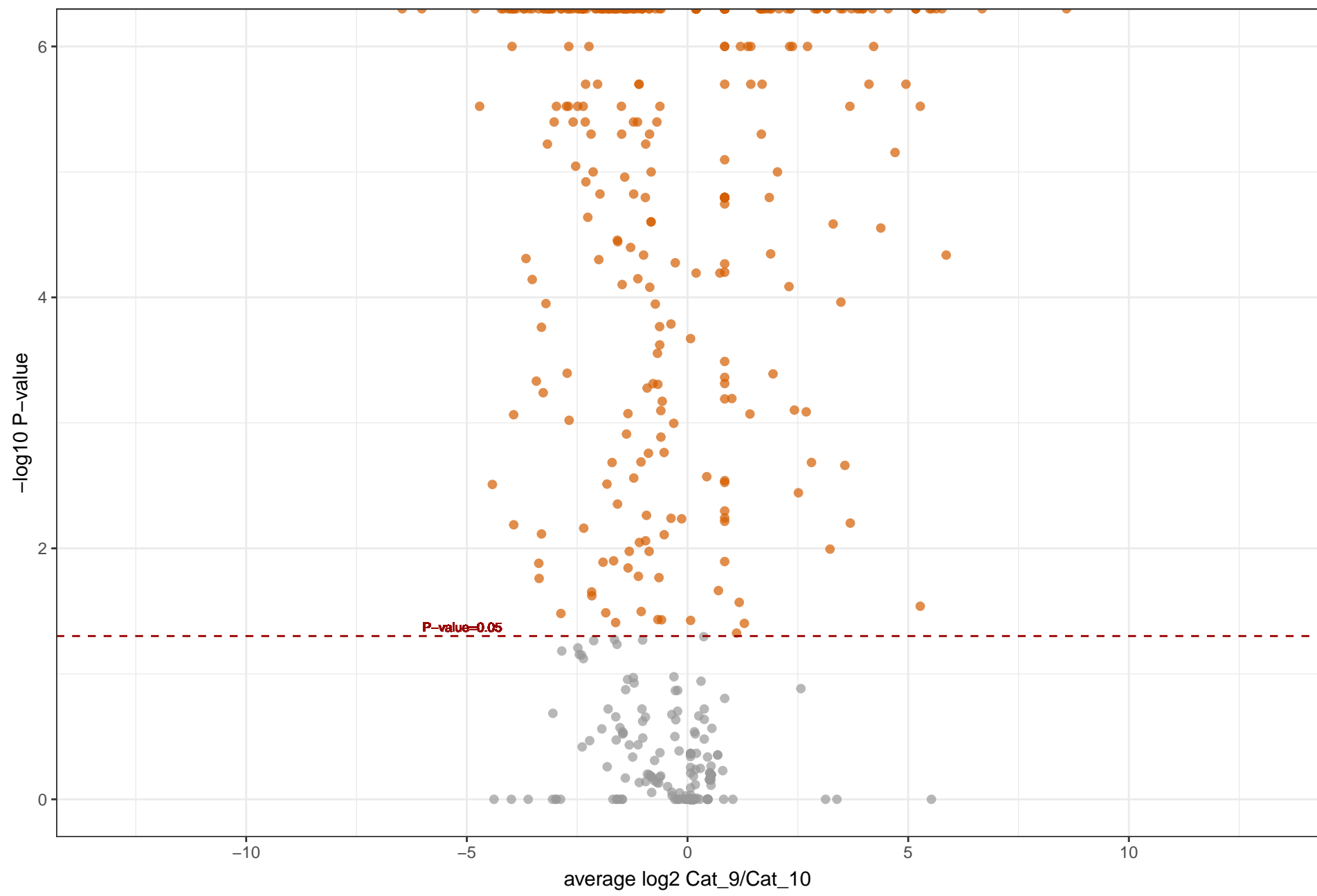

Supplement: Figure 6—source data 1. — Individual data from all figures involving small datasets displayed in individual tabs of this source file. This includes Figures 1B and 2A-F, Figure 3B, Figure 4, Figure 1—figure supplement 1 and Figure 2—figure supplement 1. [file elife-75798-fig6-data1.zip › Flores_Data/AF1_Cat_9.Cat_10-volcano_AFCat1.pdf]

MA plot

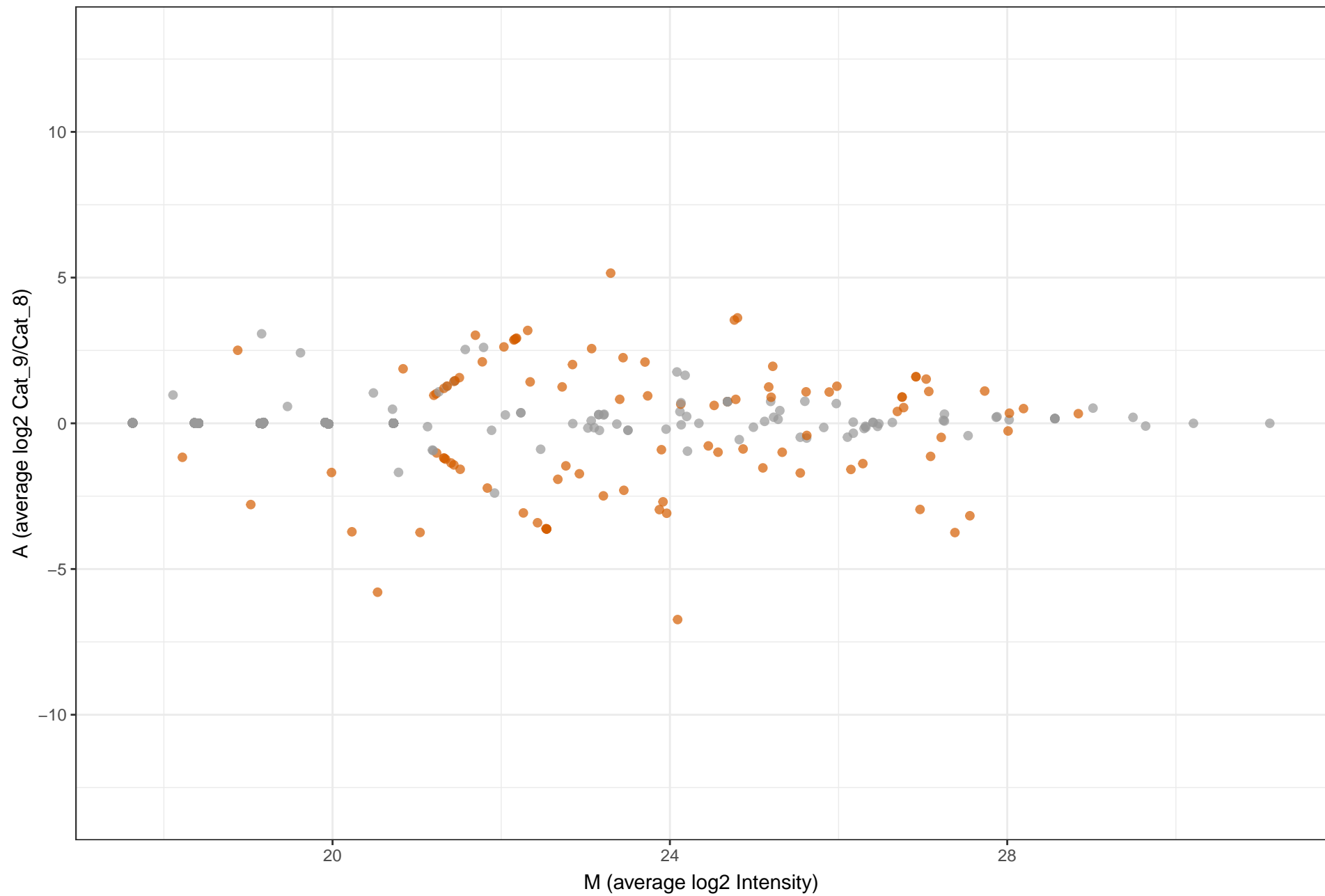

Supplement: Figure 6—source data 1. — Individual data from all figures involving small datasets displayed in individual tabs of this source file. This includes Figures 1B and 2A-F, Figure 3B, Figure 4, Figure 1—figure supplement 1 and Figure 2—figure supplement 1. [file elife-75798-fig6-data1.zip › Flores_Data/AF1_Cat_9.Cat_8-MA_AFCat1.pdf]

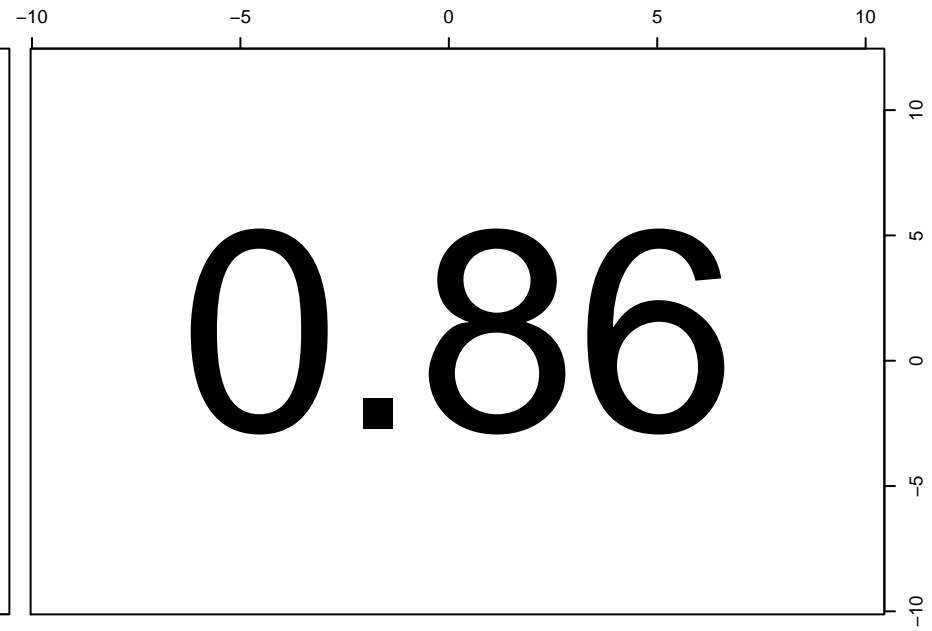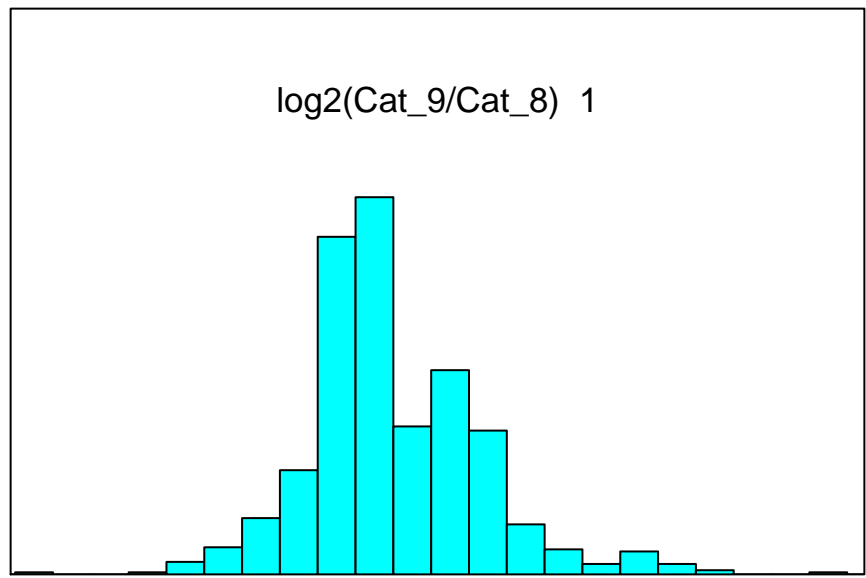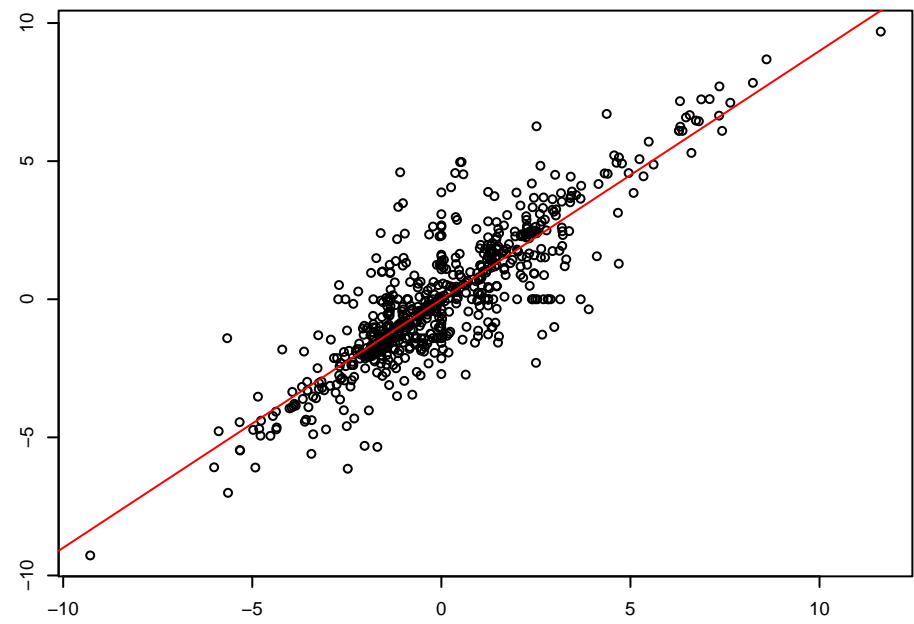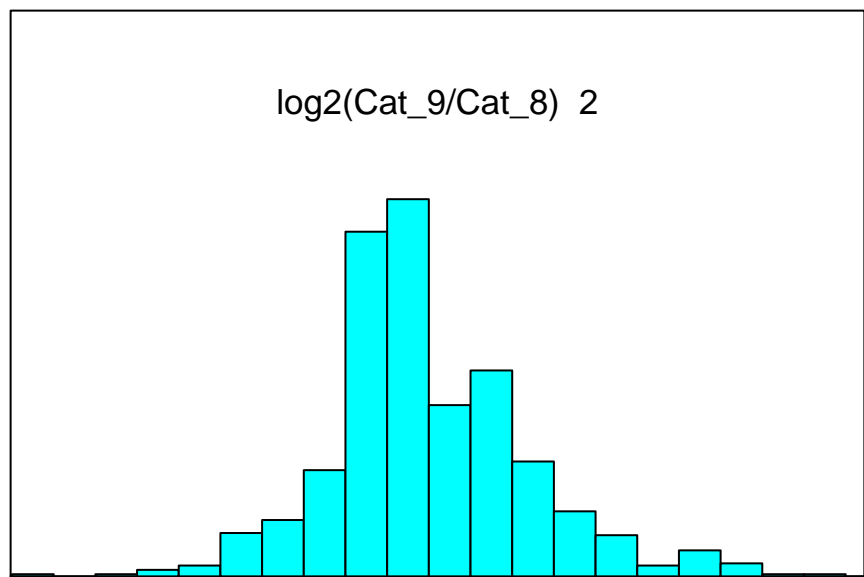

Supplement: Figure 6—source data 1. — Individual data from all figures involving small datasets displayed in individual tabs of this source file. This includes Figures 1B and 2A-F, Figure 3B, Figure 4, Figure 1—figure supplement 1 and Figure 2—figure supplement 1. [file elife-75798-fig6-data1.zip › Flores_Data/AF1_Cat_9.Cat_8-reproducibility_AFCat1.pdf]

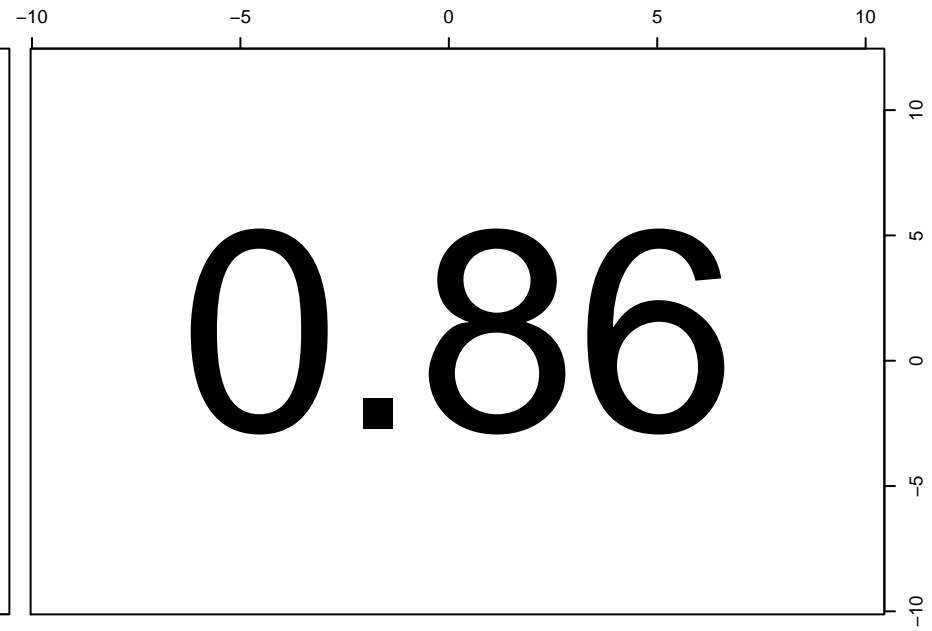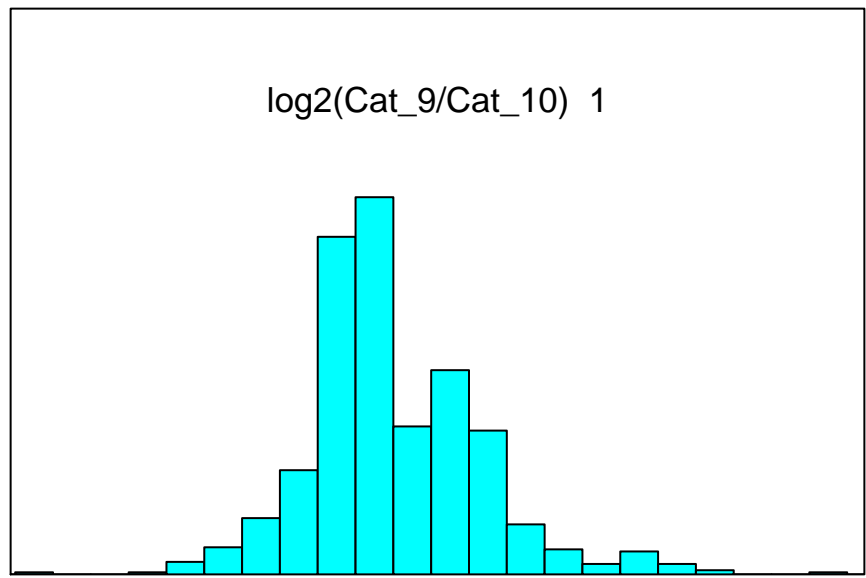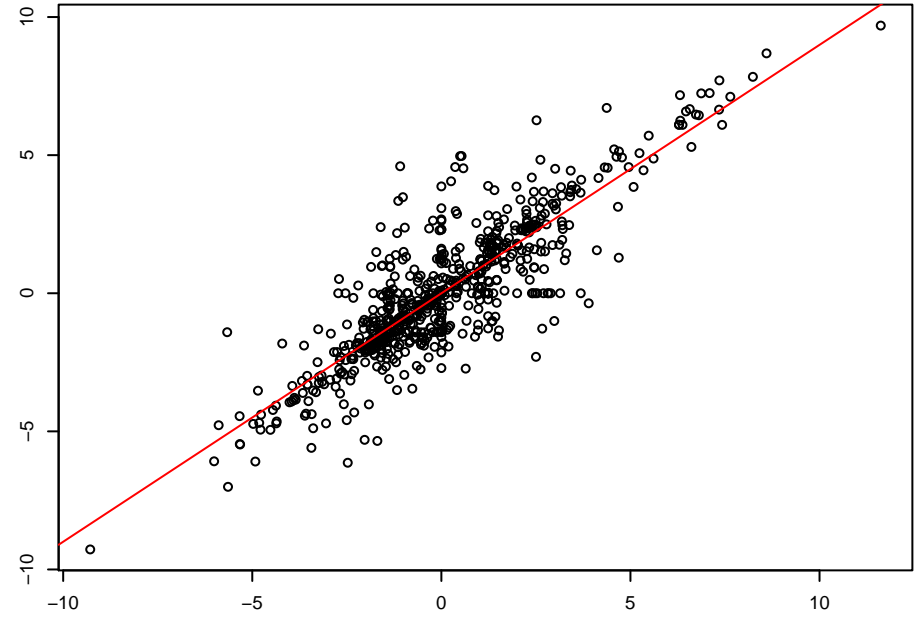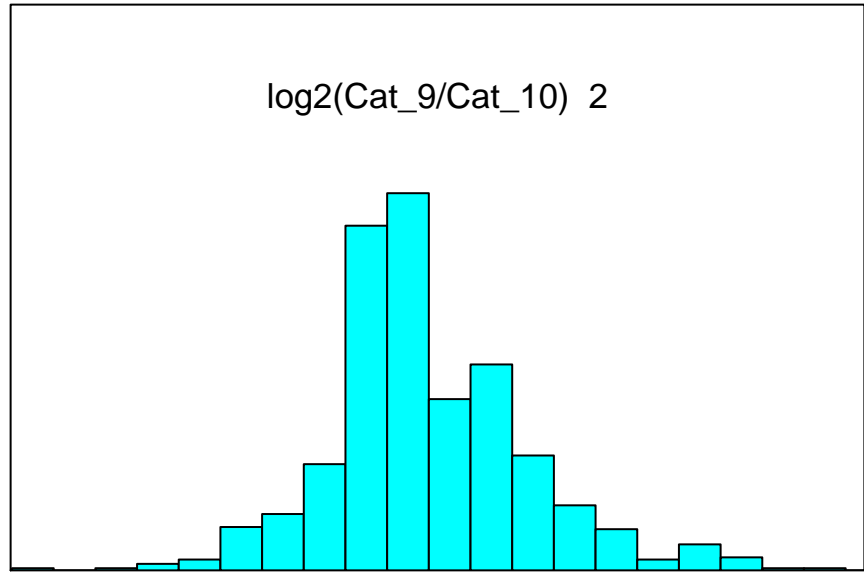

Supplement: Figure 6—source data 1. — Individual data from all figures involving small datasets displayed in individual tabs of this source file. This includes Figures 1B and 2A-F, Figure 3B, Figure 4, Figure 1—figure supplement 1 and Figure 2—figure supplement 1. [file elife-75798-fig6-data1.zip › Flores_Data/AF1_Cat_9.Cat_10-reproducibility_AFCat1.pdf]

Value-ordered fold change

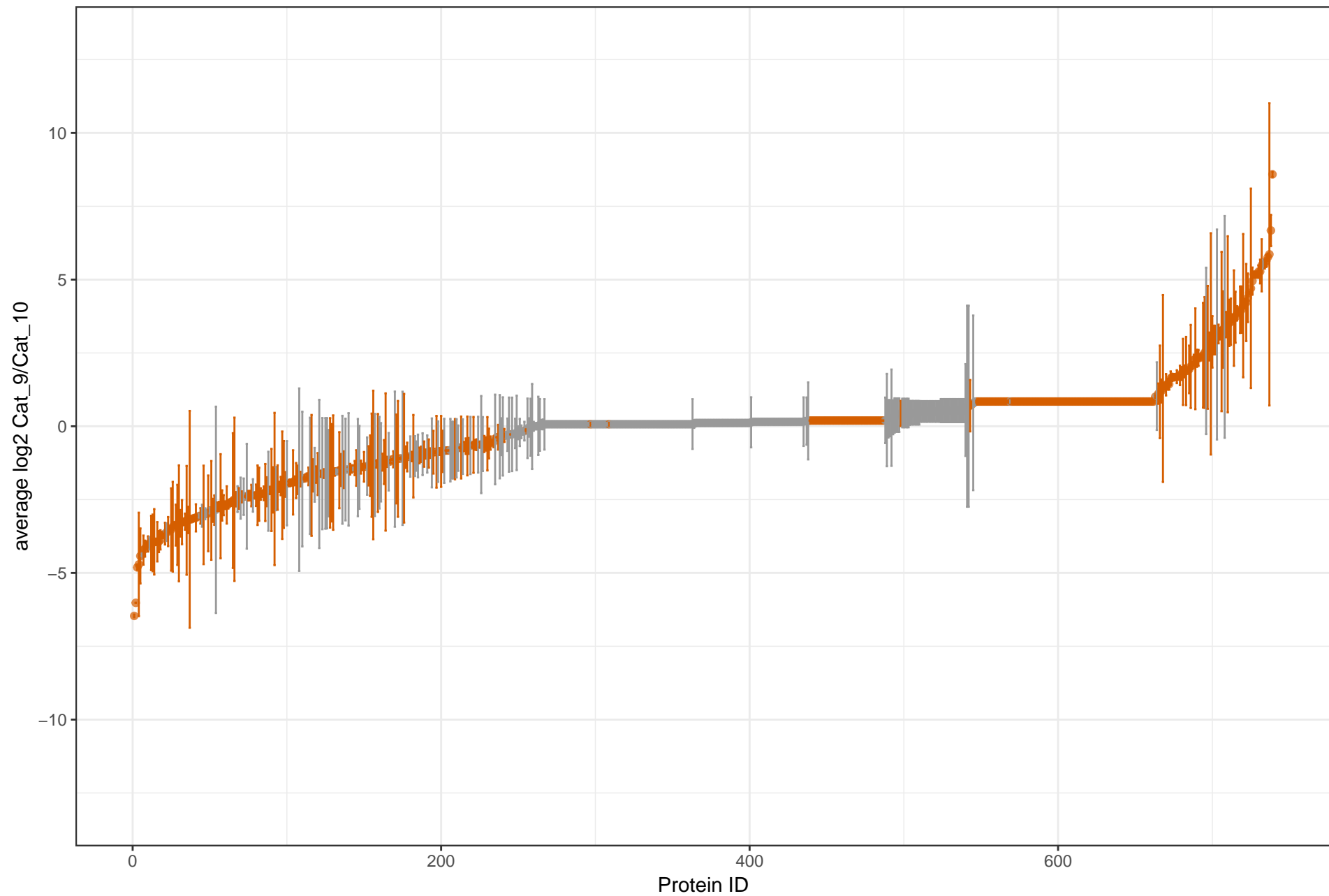

Supplement: Figure 6—source data 1. — Individual data from all figures involving small datasets displayed in individual tabs of this source file. This includes Figures 1B and 2A-F, Figure 3B, Figure 4, Figure 1—figure supplement 1 and Figure 2—figure supplement 1. [file elife-75798-fig6-data1.zip › Flores_Data/AF1_Cat_9.Cat_10-value-ordered-log-ratio_AFCat1.pdf]

MA plot

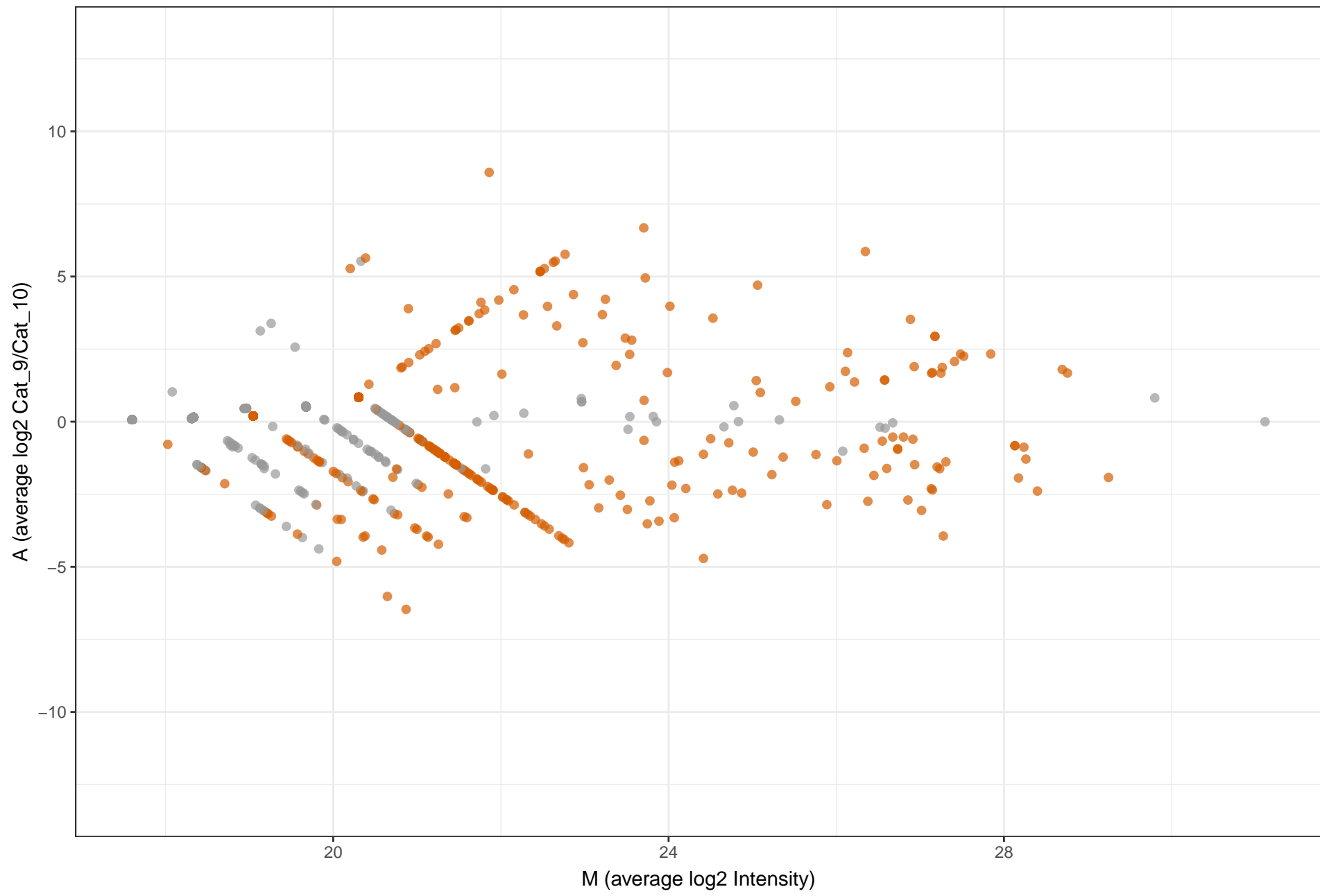

Supplement: Figure 6—source data 1. — Individual data from all figures involving small datasets displayed in individual tabs of this source file. This includes Figures 1B and 2A-F, Figure 3B, Figure 4, Figure 1—figure supplement 1 and Figure 2—figure supplement 1. [file elife-75798-fig6-data1.zip › Flores_Data/AF1_Cat_9.Cat_10-MA_AFCat1.pdf]
