# Supplementary figures and images for: Inhibiting host-protein deposition on urinary catheters reduces associated urinary tract infections (part 1 of 2)
Source: eLife. 2022 Mar 29;11:e75798. doi: 10.7554/eLife.75798 (PMC8986317; doi:10.7554/eLife.75798)

**P-value vs Fold change**

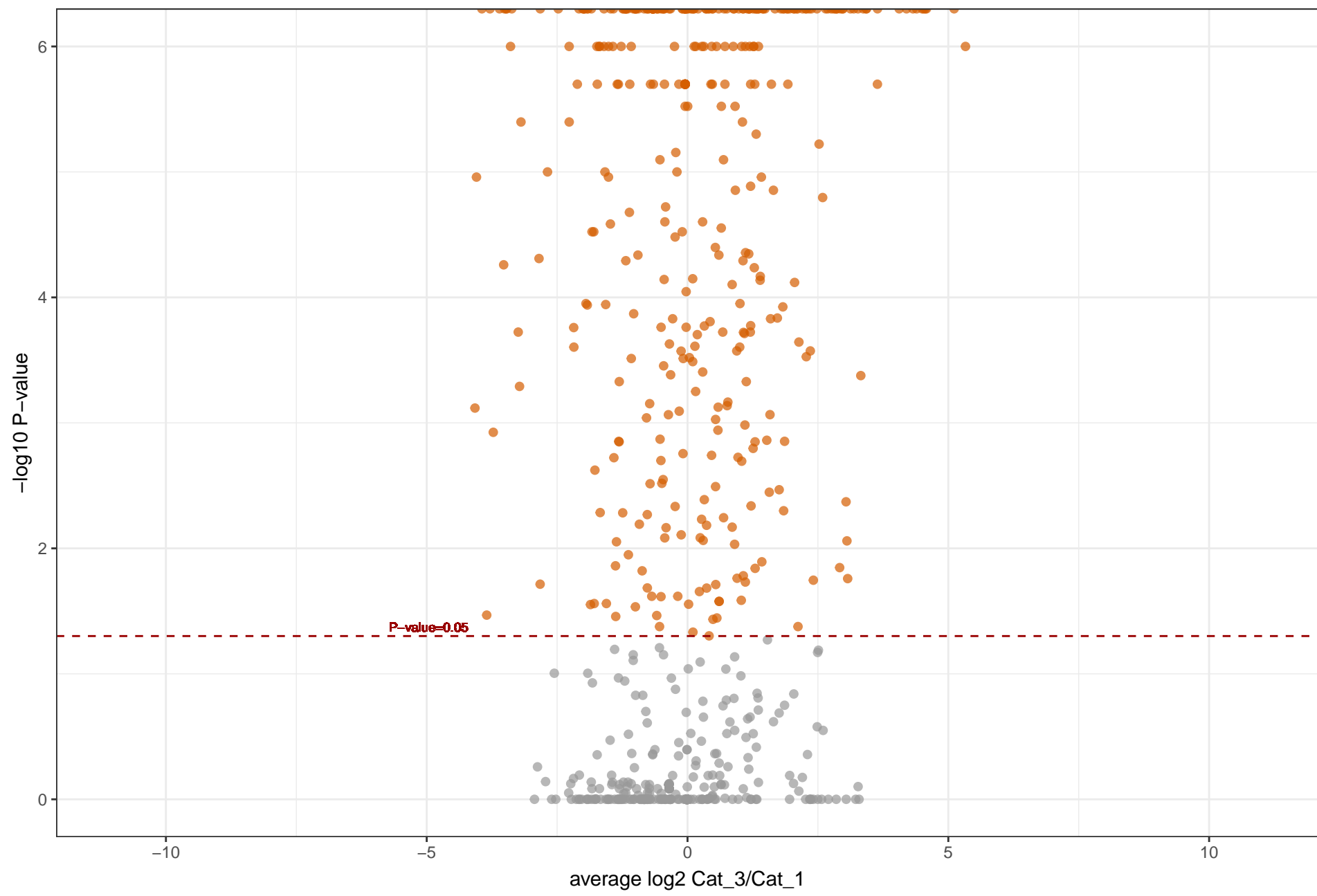

Supplement: Figure 6—source data 1. — Individual data from all figures involving small datasets displayed in individual tabs of this source file. This includes Figures 1B and 2A-F, Figure 3B, Figure 4, Figure 1—figure supplement 1 and Figure 2—figure supplement 1. [file elife-75798-fig6-data1.zip › Flores_Data/AF1_Cat_3.Cat_1-volcano_AFCat1.pdf]

Value-ordered fold change

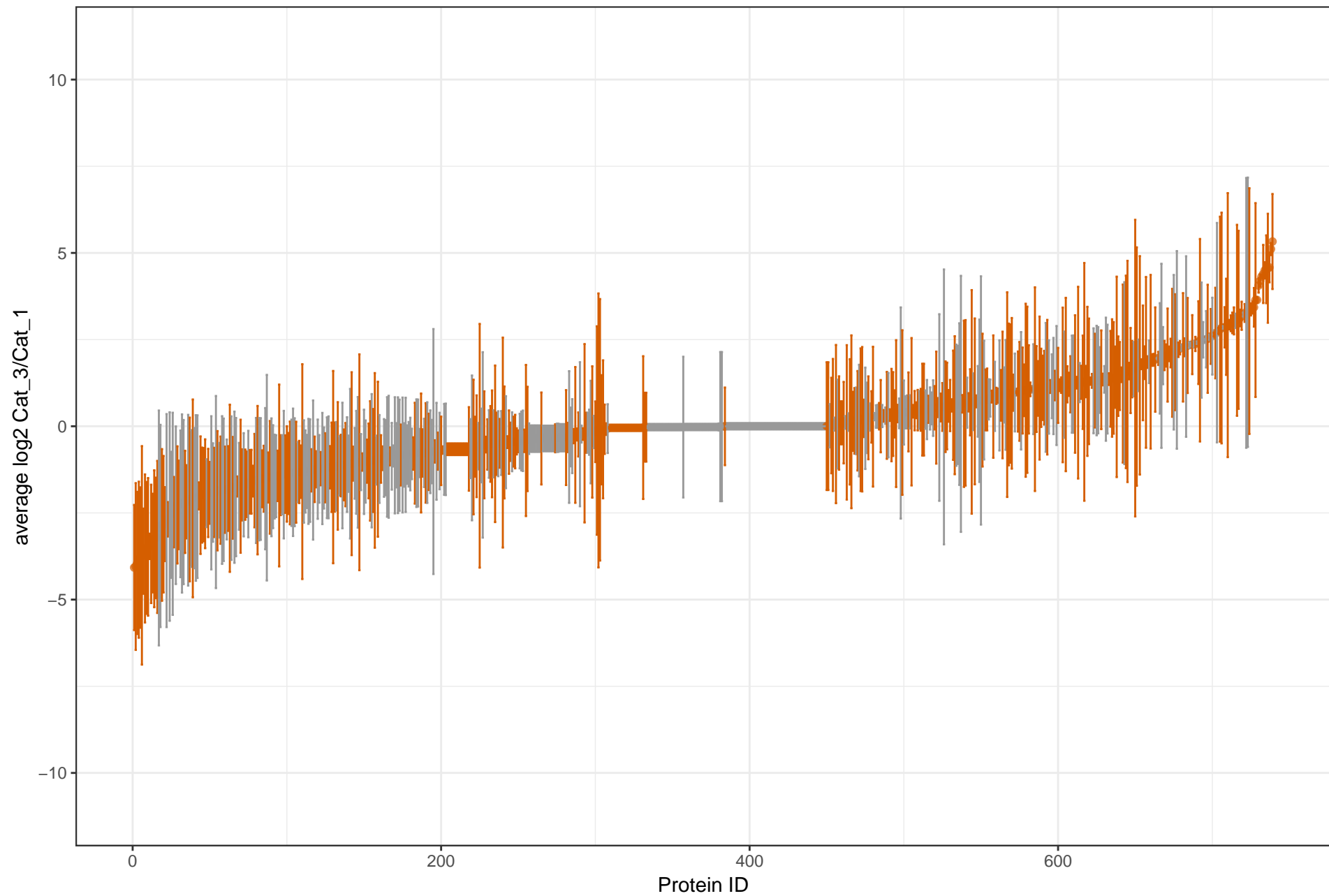

Supplement: Figure 6—source data 1. — Individual data from all figures involving small datasets displayed in individual tabs of this source file. This includes Figures 1B and 2A-F, Figure 3B, Figure 4, Figure 1—figure supplement 1 and Figure 2—figure supplement 1. [file elife-75798-fig6-data1.zip › Flores_Data/AF1_Cat_3.Cat_1-value-ordered-log-ratio_AFCat1.pdf]

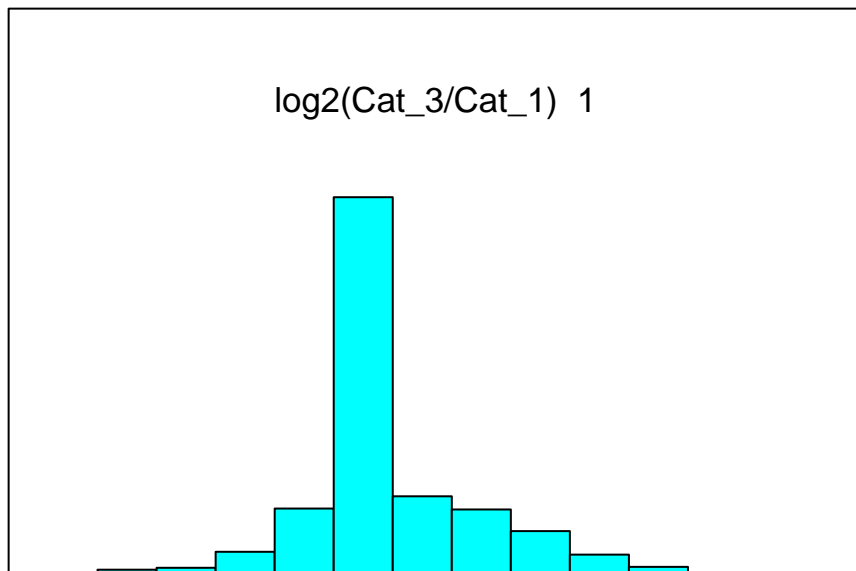

-6 -4 -2 0 2 4 6

6  
4  
2  
0  
-2  
-4  
-6

0.69

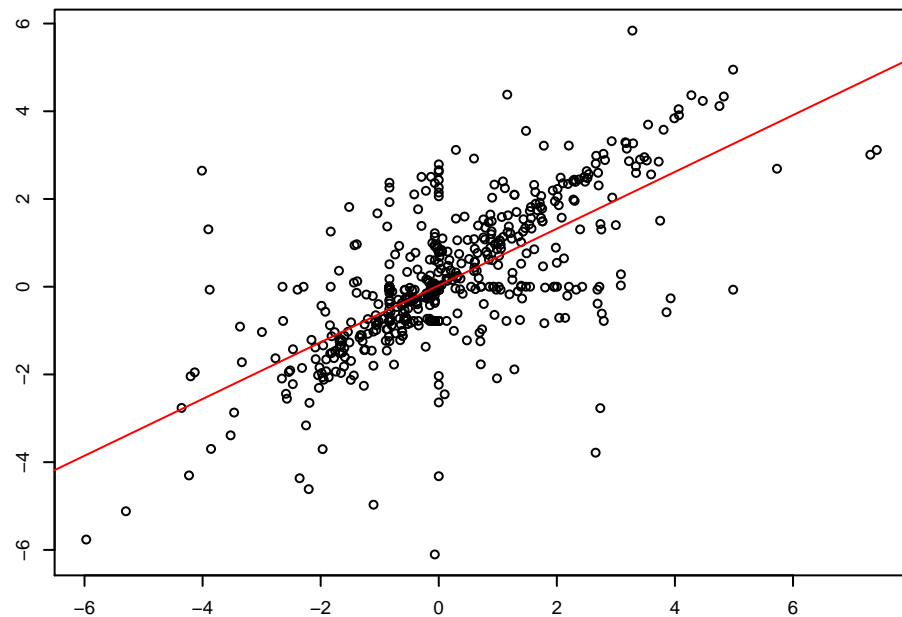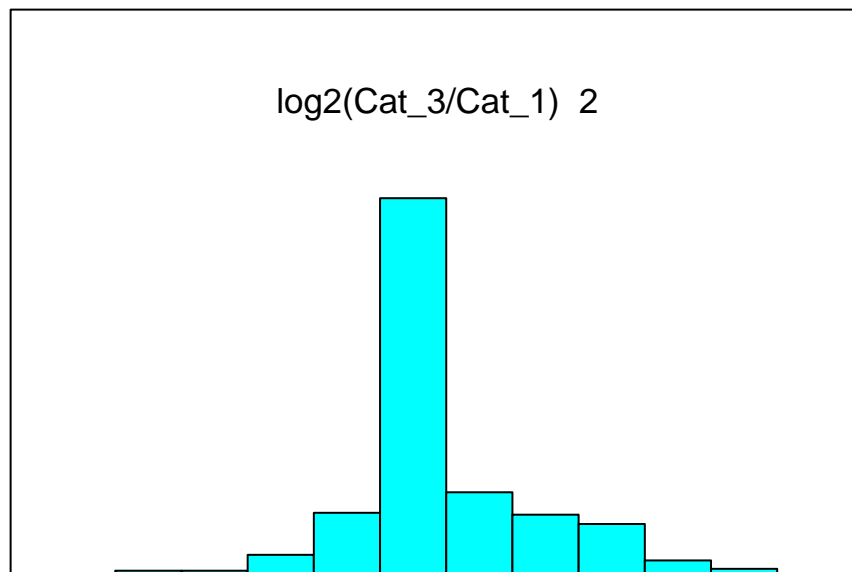

Supplement: Figure 6—source data 1. — Individual data from all figures involving small datasets displayed in individual tabs of this source file. This includes Figures 1B and 2A-F, Figure 3B, Figure 4, Figure 1—figure supplement 1 and Figure 2—figure supplement 1. [file elife-75798-fig6-data1.zip › Flores_Data/AF1_Cat_3.Cat_1-reproducibility_AFCat1.pdf]

MA plot

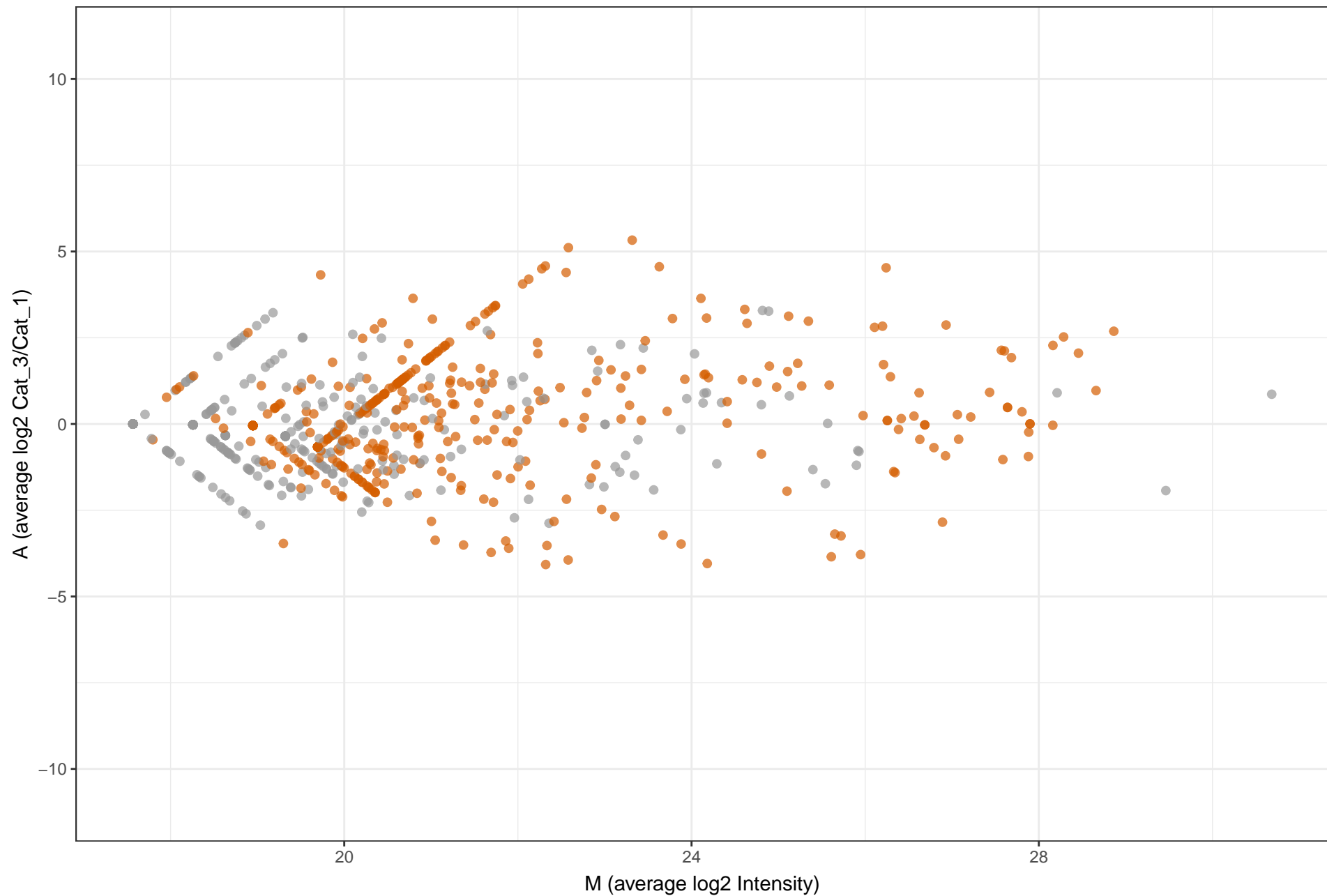

Supplement: Figure 6—source data 1. — Individual data from all figures involving small datasets displayed in individual tabs of this source file. This includes Figures 1B and 2A-F, Figure 3B, Figure 4, Figure 1—figure supplement 1 and Figure 2—figure supplement 1. [file elife-75798-fig6-data1.zip › Flores_Data/AF1_Cat_3.Cat_1-MA_AFCat1.pdf]

**P-value vs Fold change**

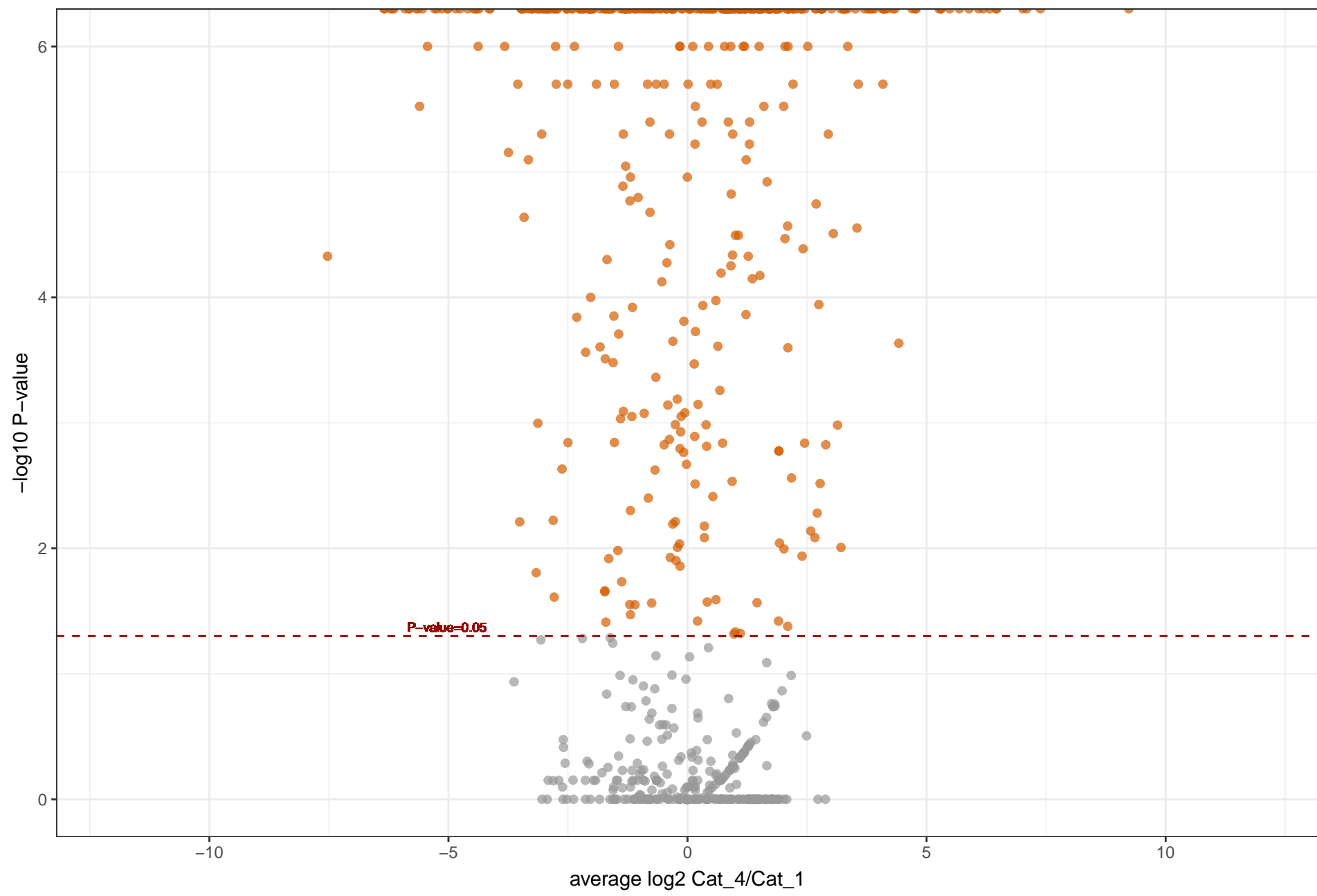

Supplement: Figure 6—source data 1. — Individual data from all figures involving small datasets displayed in individual tabs of this source file. This includes Figures 1B and 2A-F, Figure 3B, Figure 4, Figure 1—figure supplement 1 and Figure 2—figure supplement 1. [file elife-75798-fig6-data1.zip › Flores_Data/AF1_Cat_4.Cat_1-volcano_AFCat1.pdf]

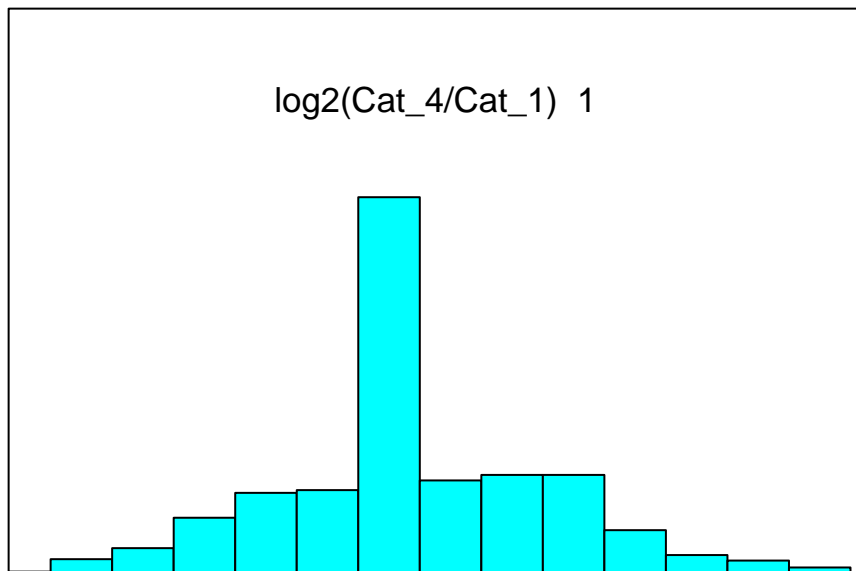

-6 -4 -2 0 2 4 6

6  
4  
2  
0  
-2  
-4  
-6

0.82

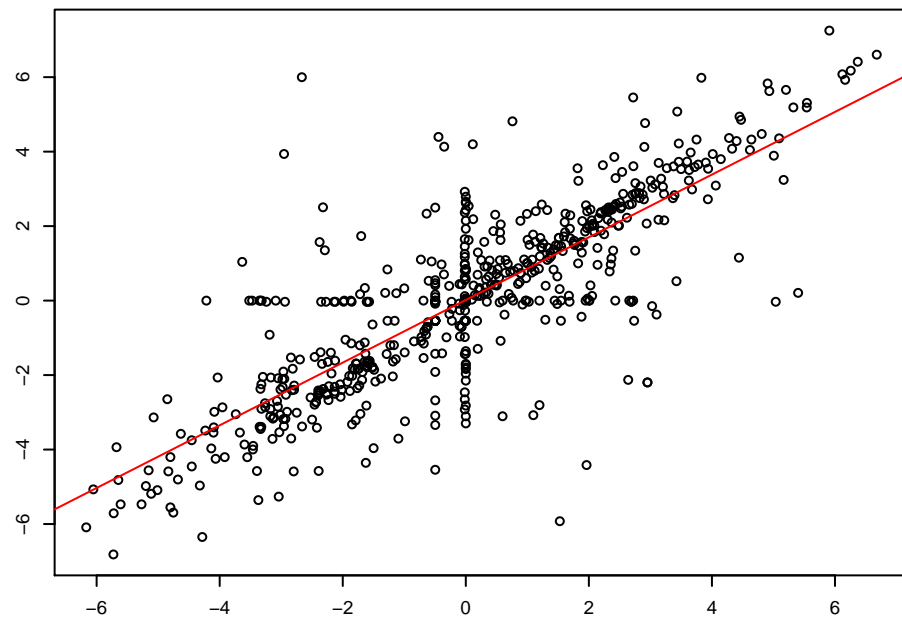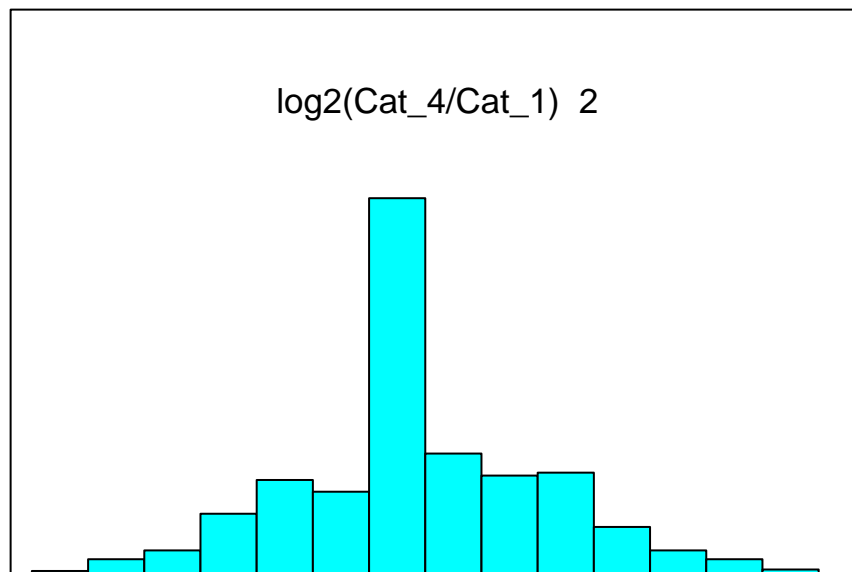

Supplement: Figure 6—source data 1. — Individual data from all figures involving small datasets displayed in individual tabs of this source file. This includes Figures 1B and 2A-F, Figure 3B, Figure 4, Figure 1—figure supplement 1 and Figure 2—figure supplement 1. [file elife-75798-fig6-data1.zip › Flores_Data/AF1_Cat_4.Cat_1-reproducibility_AFCat1.pdf]

MA plot

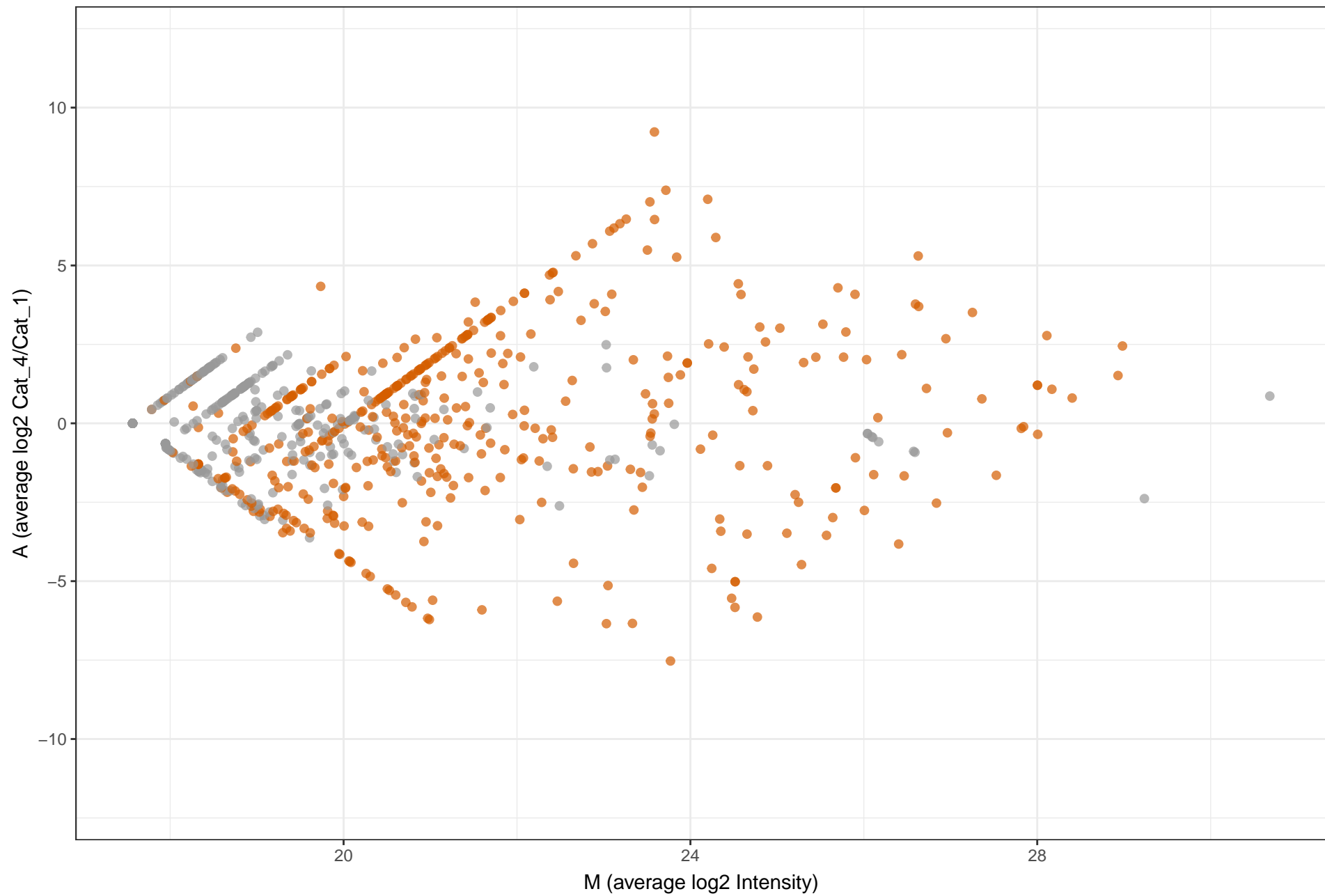

Supplement: Figure 6—source data 1. — Individual data from all figures involving small datasets displayed in individual tabs of this source file. This includes Figures 1B and 2A-F, Figure 3B, Figure 4, Figure 1—figure supplement 1 and Figure 2—figure supplement 1. [file elife-75798-fig6-data1.zip › Flores_Data/AF1_Cat_4.Cat_1-MA_AFCat1.pdf]

**P-value vs Fold change**

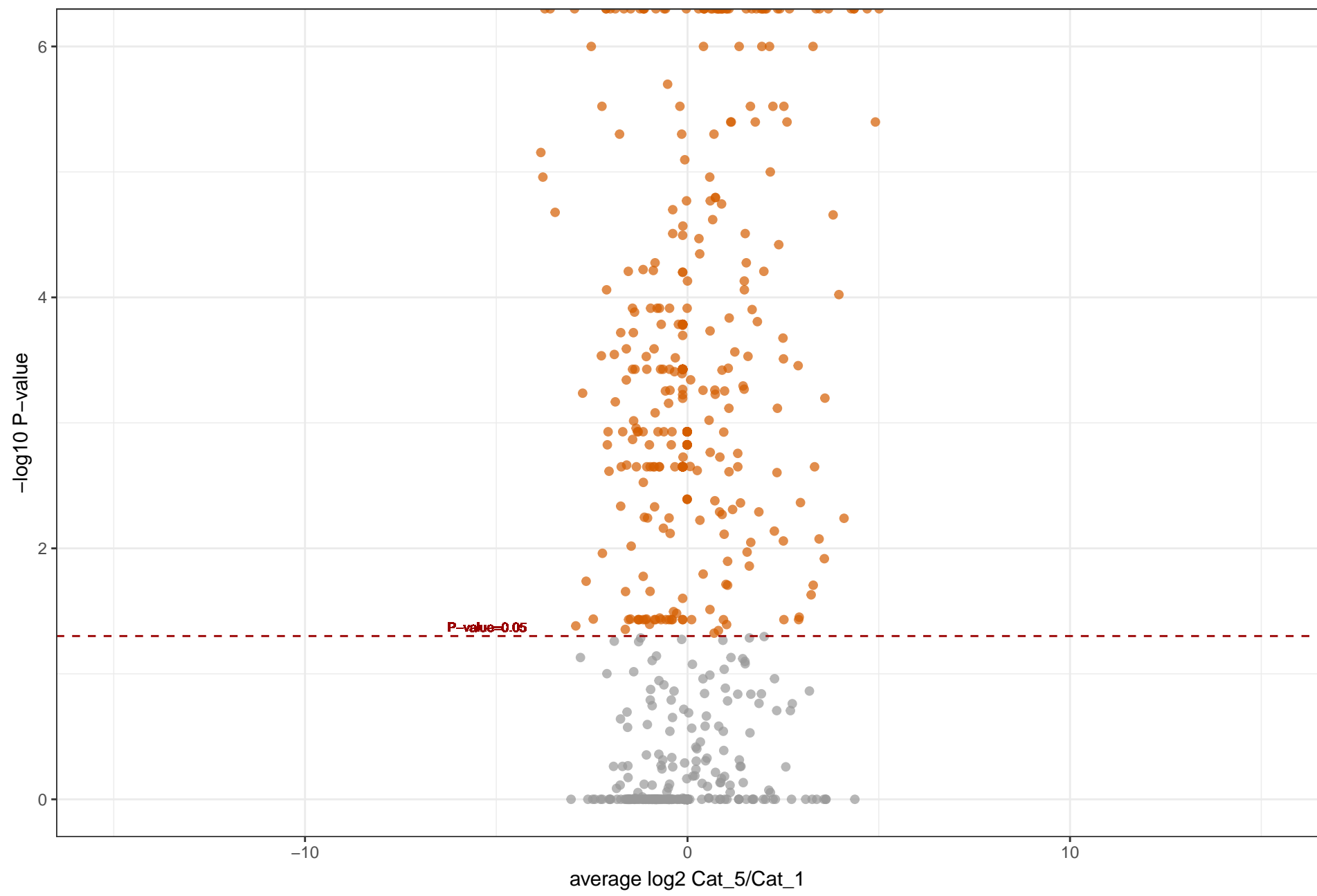

Supplement: Figure 6—source data 1. — Individual data from all figures involving small datasets displayed in individual tabs of this source file. This includes Figures 1B and 2A-F, Figure 3B, Figure 4, Figure 1—figure supplement 1 and Figure 2—figure supplement 1. [file elife-75798-fig6-data1.zip › Flores_Data/AF1_Cat_5.Cat_1-volcano_AFCat1.pdf]

Value-ordered fold change

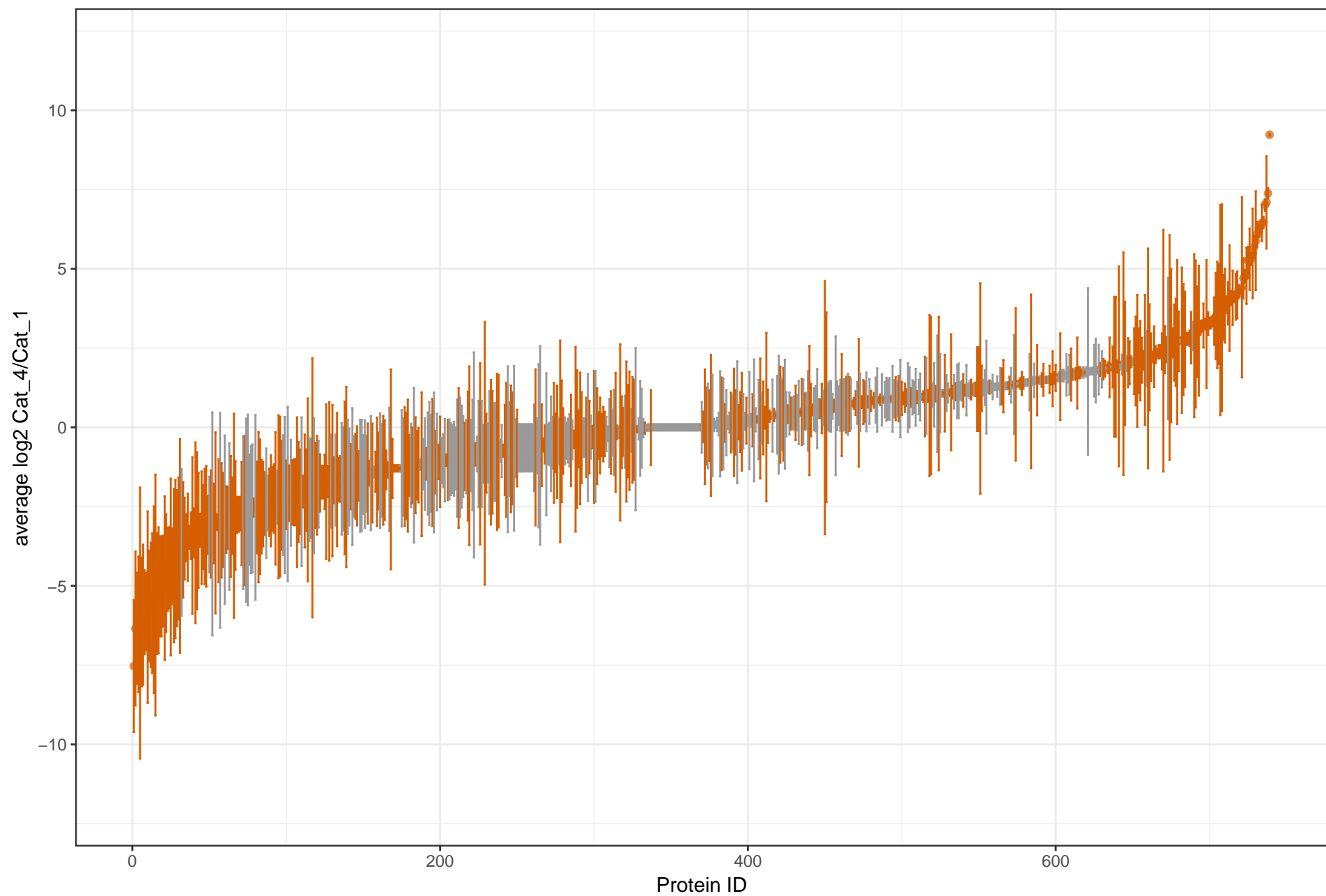

Supplement: Figure 6—source data 1. — Individual data from all figures involving small datasets displayed in individual tabs of this source file. This includes Figures 1B and 2A-F, Figure 3B, Figure 4, Figure 1—figure supplement 1 and Figure 2—figure supplement 1. [file elife-75798-fig6-data1.zip › Flores_Data/AF1_Cat_4.Cat_1-value-ordered-log-ratio_AFCat1.pdf]

MA plot

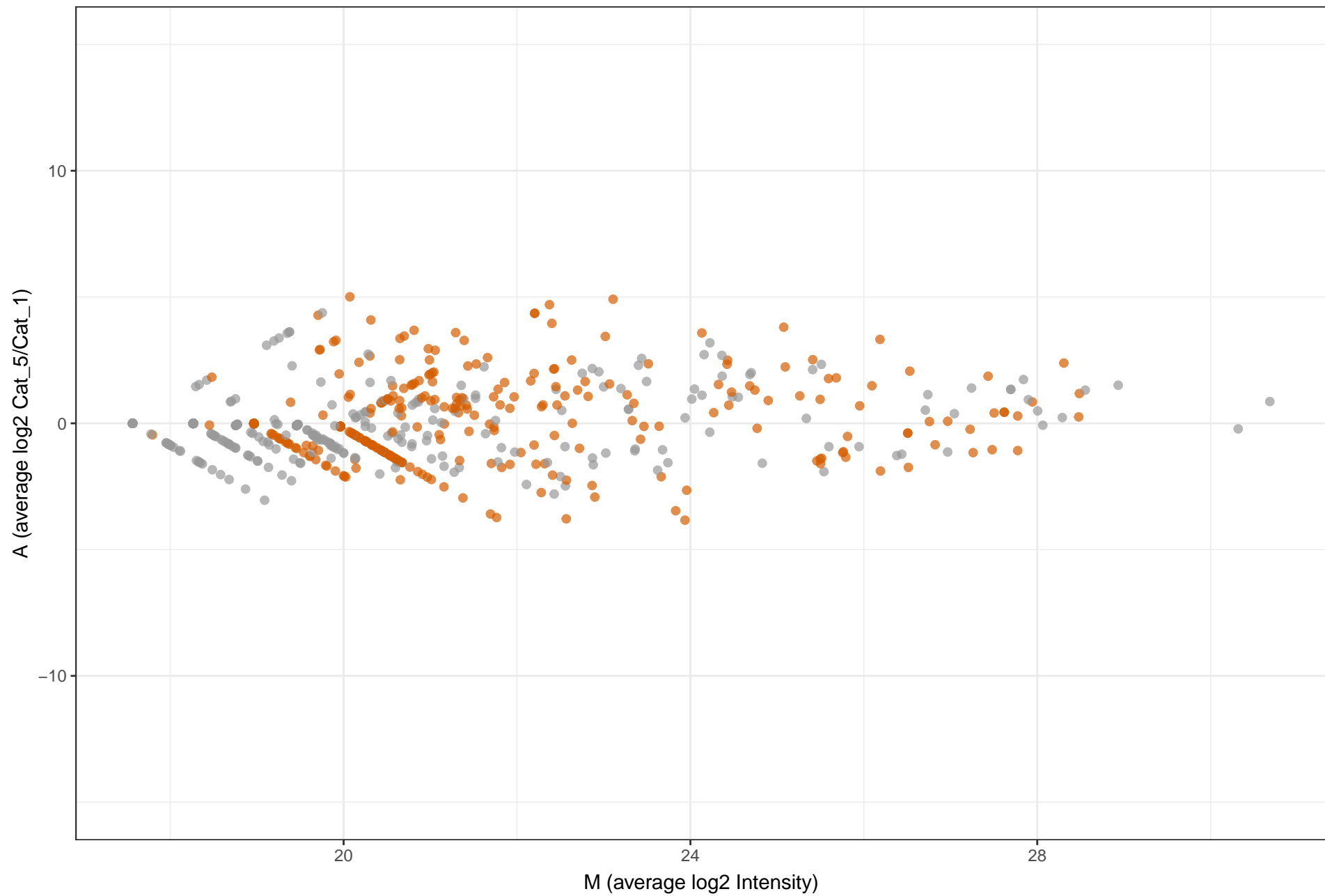

Supplement: Figure 6—source data 1. — Individual data from all figures involving small datasets displayed in individual tabs of this source file. This includes Figures 1B and 2A-F, Figure 3B, Figure 4, Figure 1—figure supplement 1 and Figure 2—figure supplement 1. [file elife-75798-fig6-data1.zip › Flores_Data/AF1_Cat_5.Cat_1-MA_AFCat1.pdf]

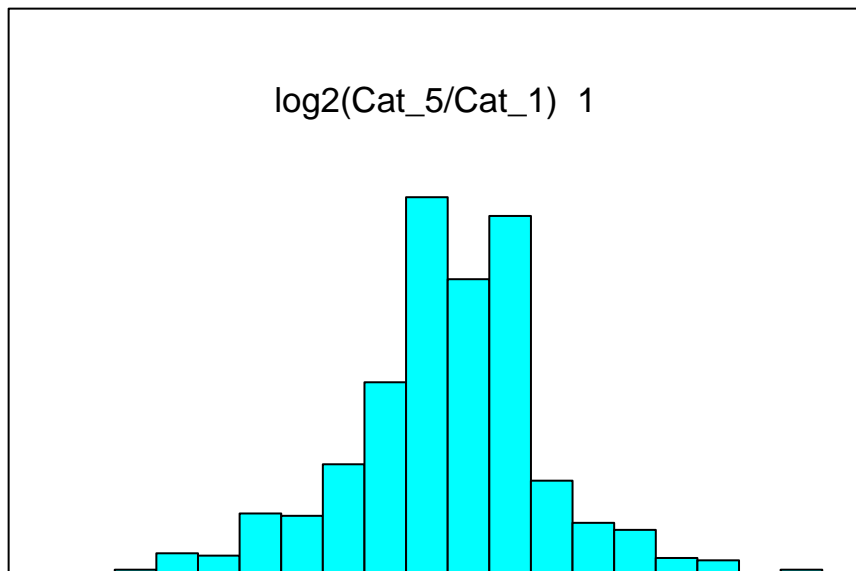

-10 -5 0 5

0.88

5 0 -5 -10

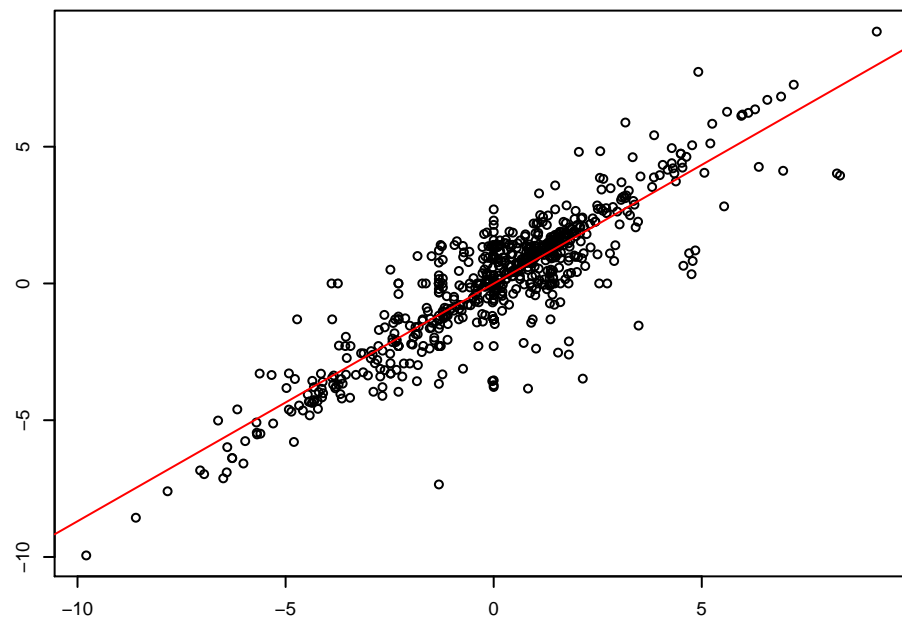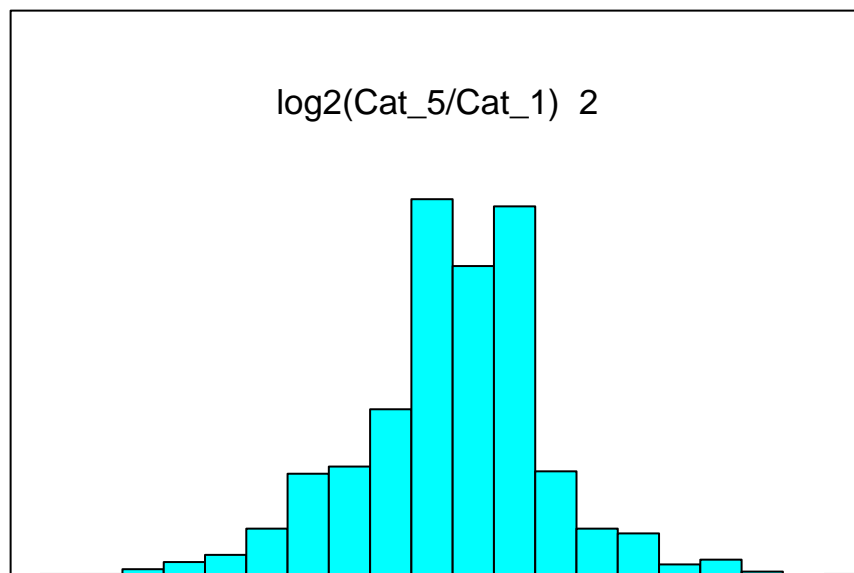

Supplement: Figure 6—source data 1. — Individual data from all figures involving small datasets displayed in individual tabs of this source file. This includes Figures 1B and 2A-F, Figure 3B, Figure 4, Figure 1—figure supplement 1 and Figure 2—figure supplement 1. [file elife-75798-fig6-data1.zip › Flores_Data/AF1_Cat_5.Cat_1-reproducibility_AFCat1.pdf]

Value-ordered fold change

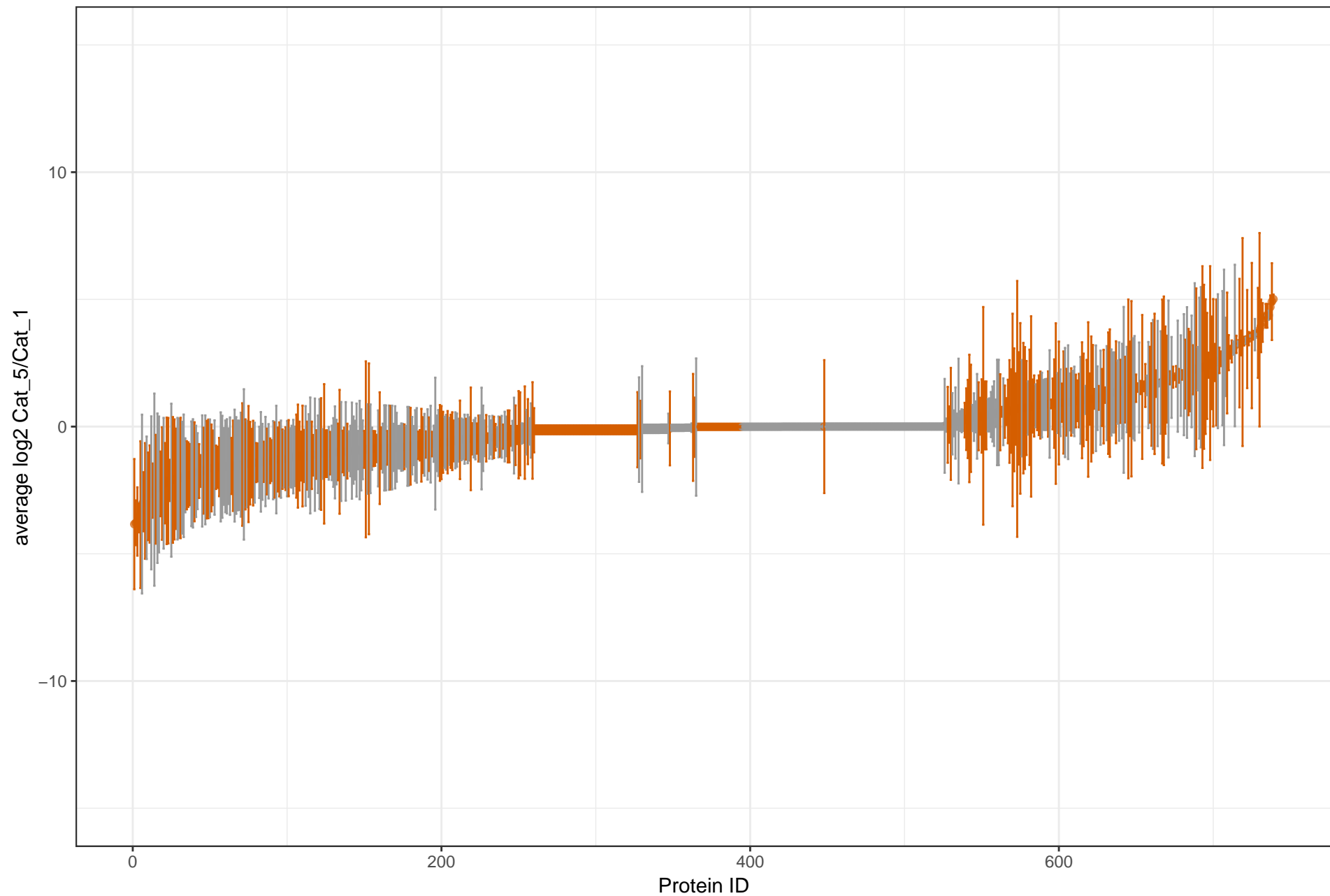

Supplement: Figure 6—source data 1. — Individual data from all figures involving small datasets displayed in individual tabs of this source file. This includes Figures 1B and 2A-F, Figure 3B, Figure 4, Figure 1—figure supplement 1 and Figure 2—figure supplement 1. [file elife-75798-fig6-data1.zip › Flores_Data/AF1_Cat_5.Cat_1-value-ordered-log-ratio_AFCat1.pdf]

P-value vs Fold change

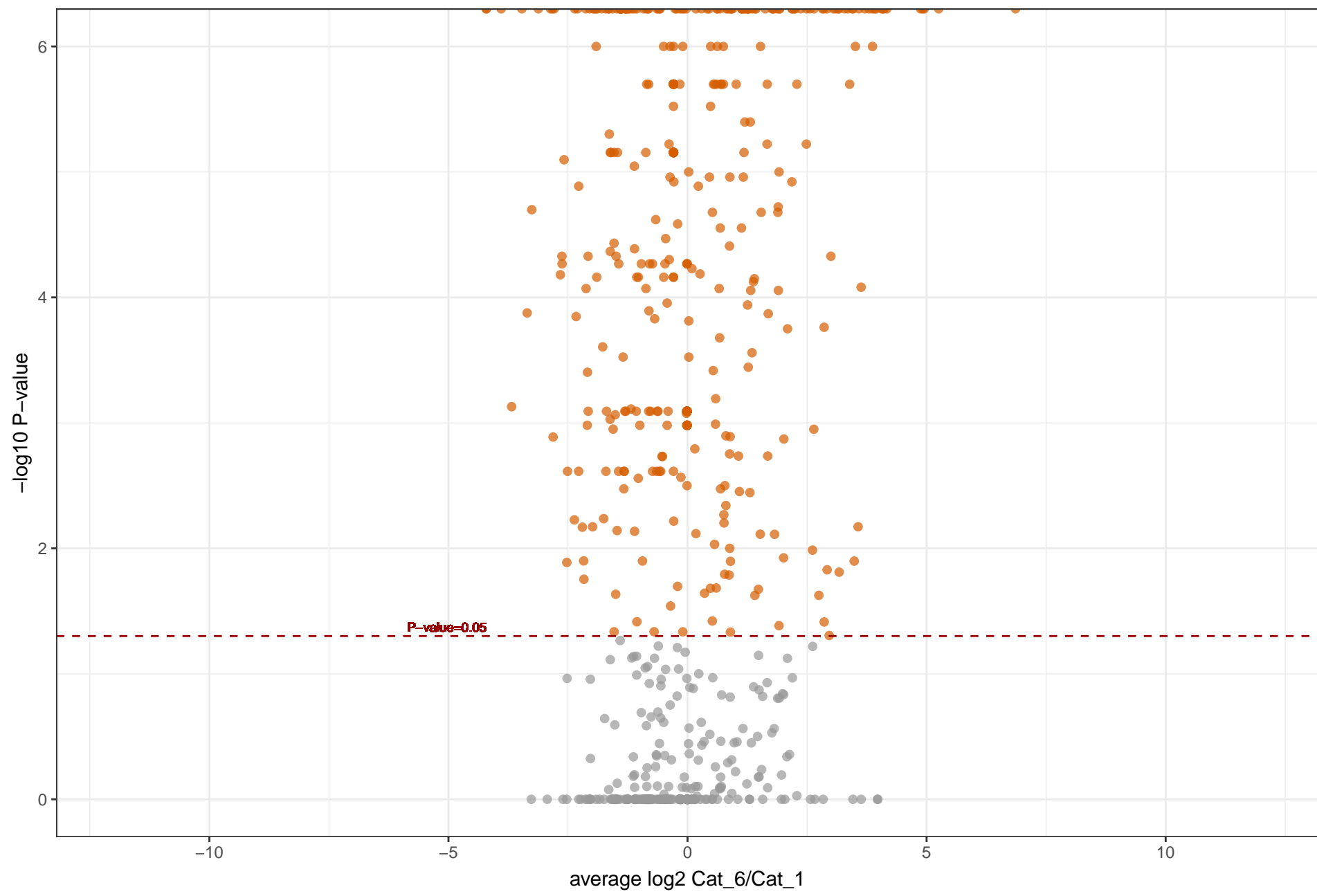

Supplement: Figure 6—source data 1. — Individual data from all figures involving small datasets displayed in individual tabs of this source file. This includes Figures 1B and 2A-F, Figure 3B, Figure 4, Figure 1—figure supplement 1 and Figure 2—figure supplement 1. [file elife-75798-fig6-data1.zip › Flores_Data/AF1_Cat_6.Cat_1-volcano_AFCat1.pdf]

MA plot

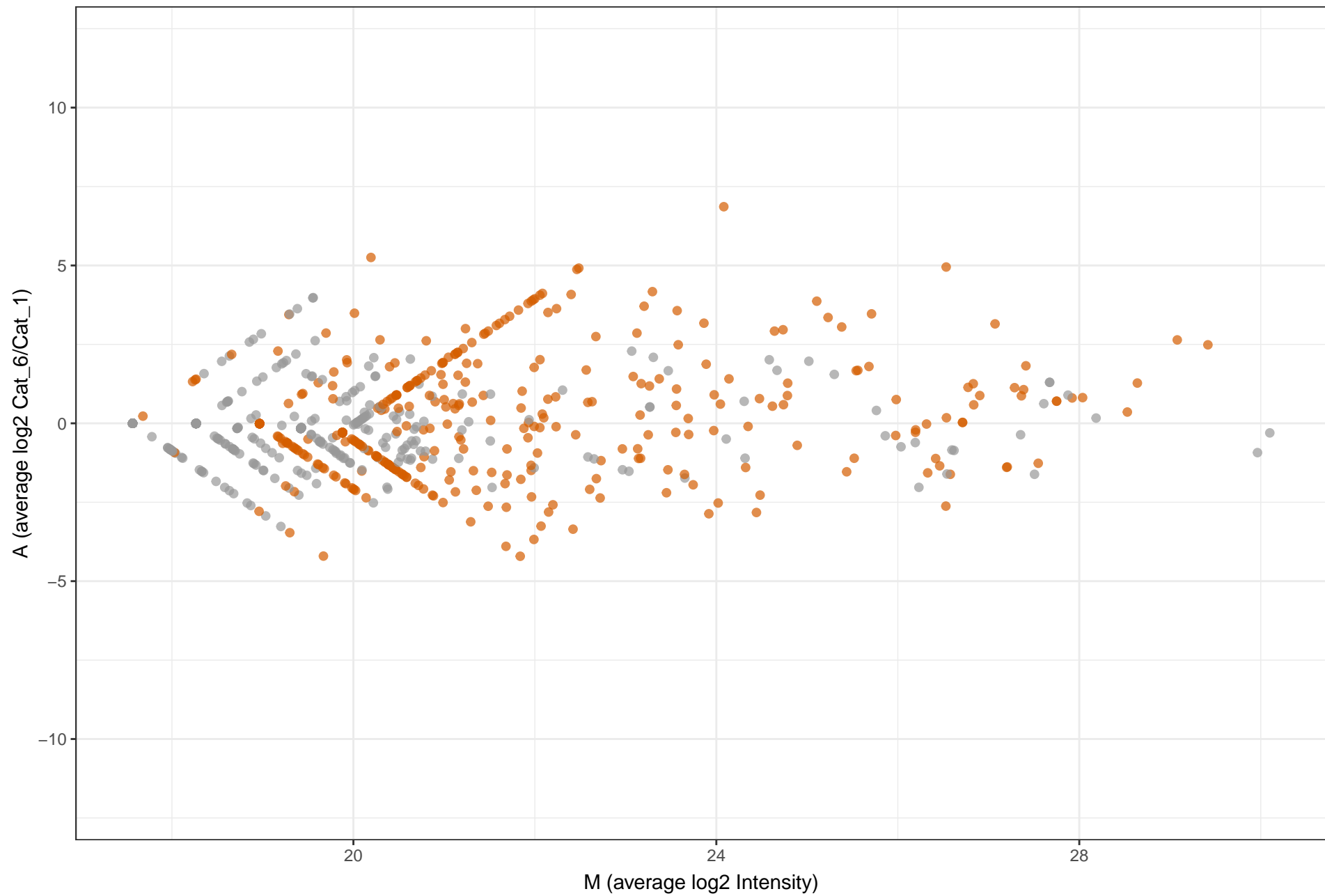

Supplement: Figure 6—source data 1. — Individual data from all figures involving small datasets displayed in individual tabs of this source file. This includes Figures 1B and 2A-F, Figure 3B, Figure 4, Figure 1—figure supplement 1 and Figure 2—figure supplement 1. [file elife-75798-fig6-data1.zip › Flores_Data/AF1_Cat_6.Cat_1-MA_AFCat1.pdf]

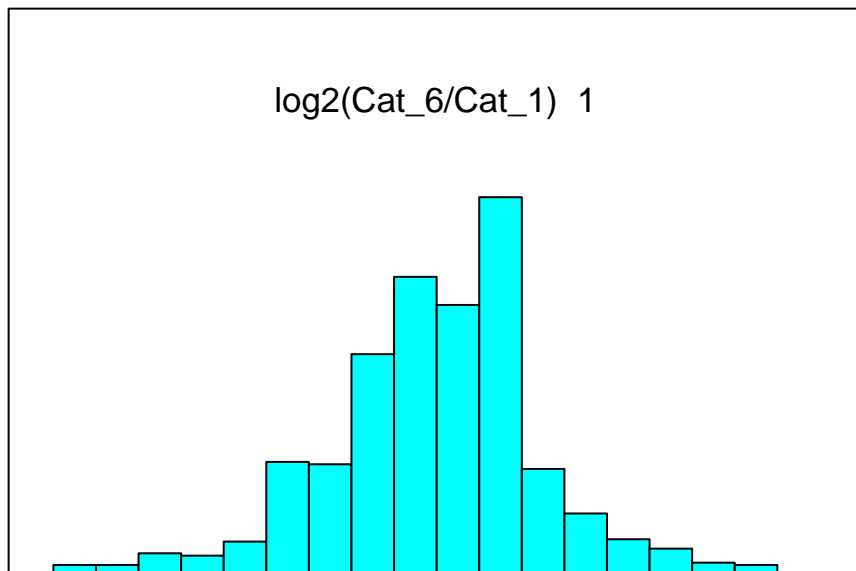

-5 0 5

0.85

5  
0  
-5  
-10

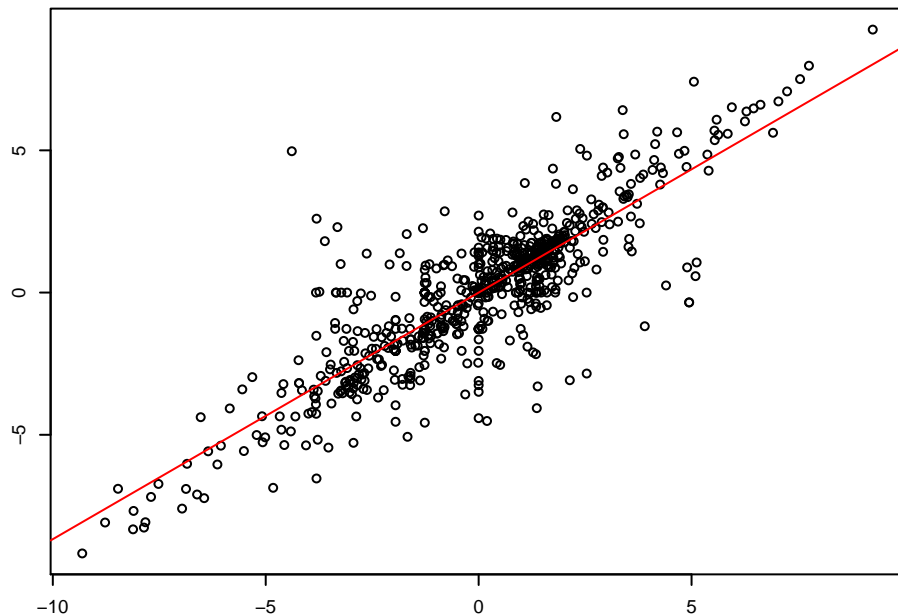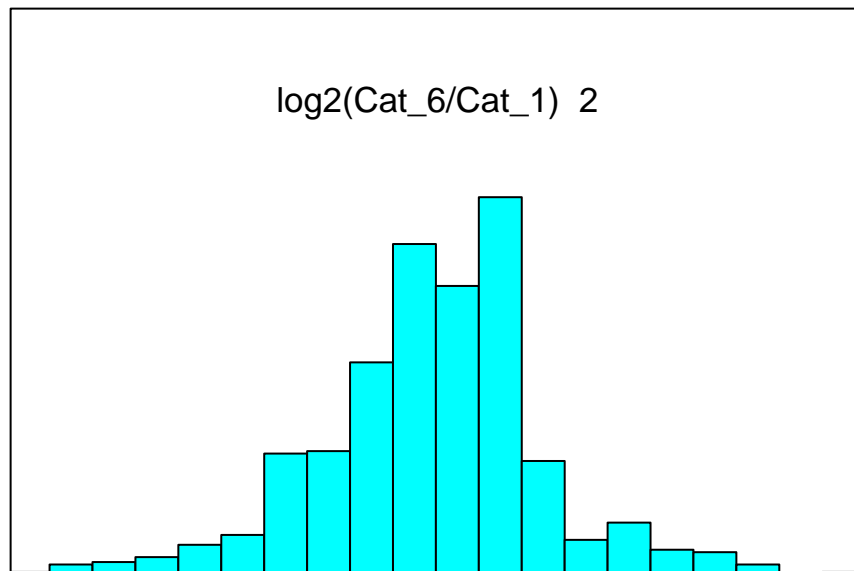

Supplement: Figure 6—source data 1. — Individual data from all figures involving small datasets displayed in individual tabs of this source file. This includes Figures 1B and 2A-F, Figure 3B, Figure 4, Figure 1—figure supplement 1 and Figure 2—figure supplement 1. [file elife-75798-fig6-data1.zip › Flores_Data/AF1_Cat_6.Cat_1-reproducibility_AFCat1.pdf]

Value-ordered fold change

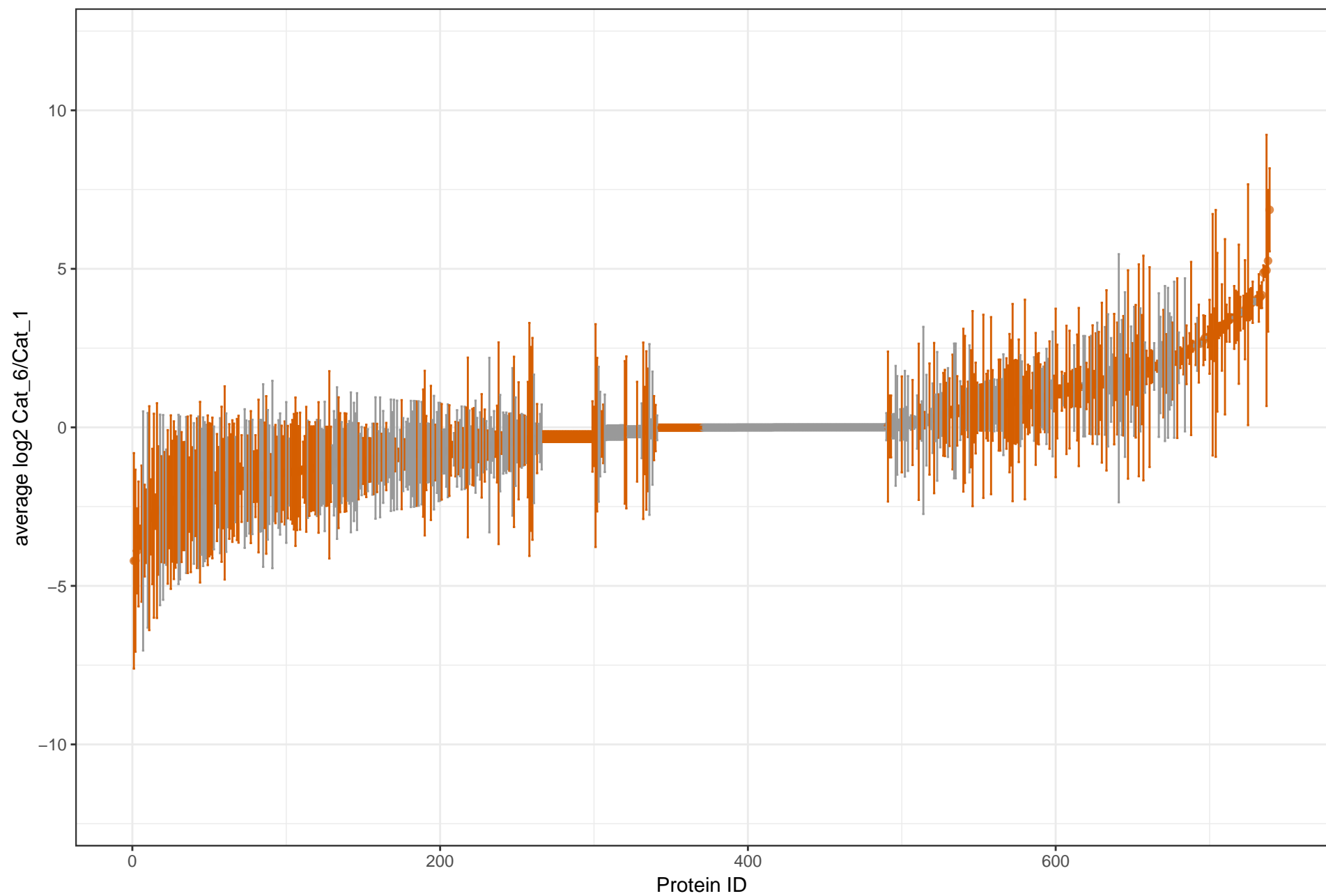

Supplement: Figure 6—source data 1. — Individual data from all figures involving small datasets displayed in individual tabs of this source file. This includes Figures 1B and 2A-F, Figure 3B, Figure 4, Figure 1—figure supplement 1 and Figure 2—figure supplement 1. [file elife-75798-fig6-data1.zip › Flores_Data/AF1_Cat_6.Cat_1-value-ordered-log-ratio_AFCat1.pdf]

P-value vs Fold change

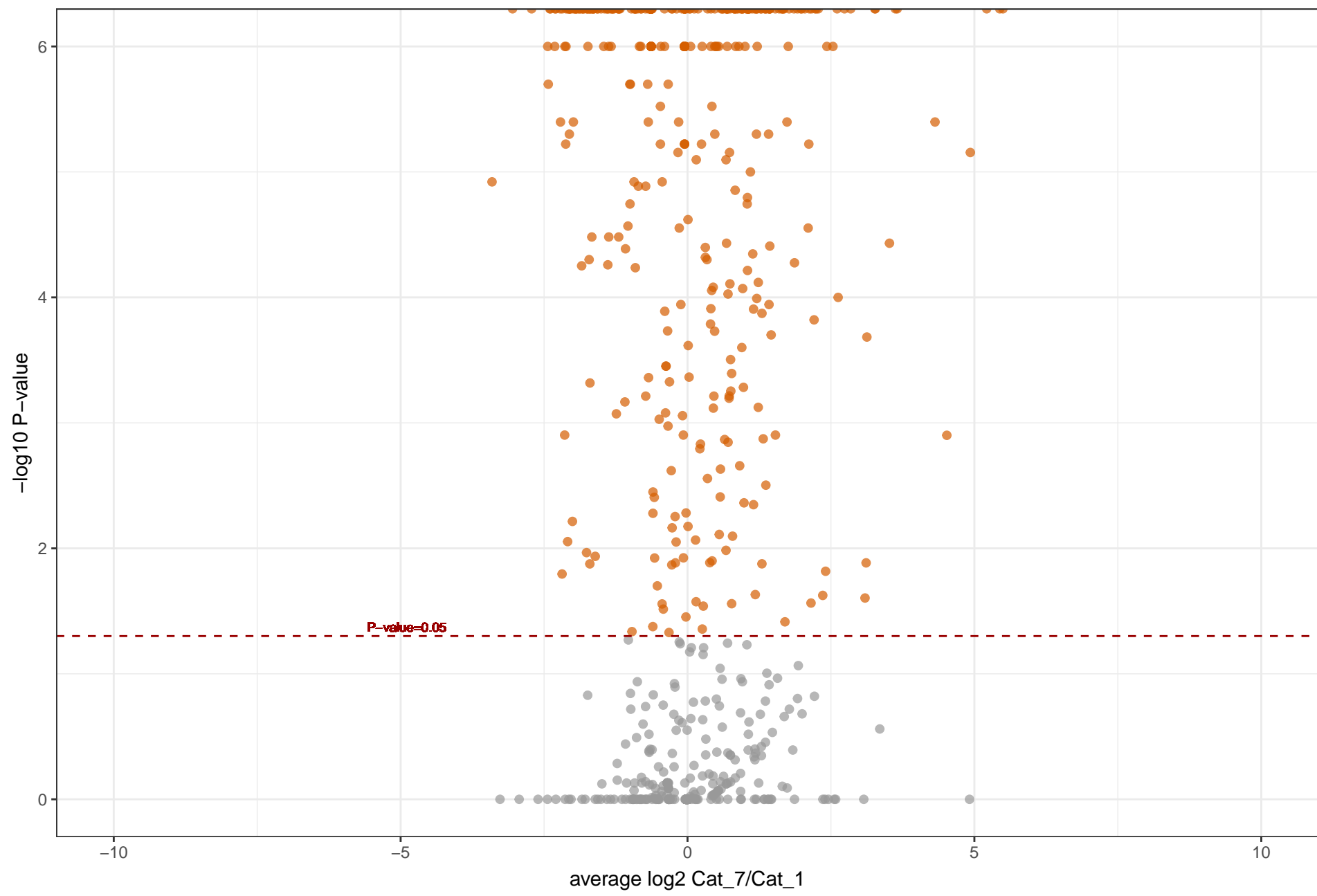

Supplement: Figure 6—source data 1. — Individual data from all figures involving small datasets displayed in individual tabs of this source file. This includes Figures 1B and 2A-F, Figure 3B, Figure 4, Figure 1—figure supplement 1 and Figure 2—figure supplement 1. [file elife-75798-fig6-data1.zip › Flores_Data/AF1_Cat_7.Cat_1-volcano_AFCat1.pdf]

Value-ordered fold change

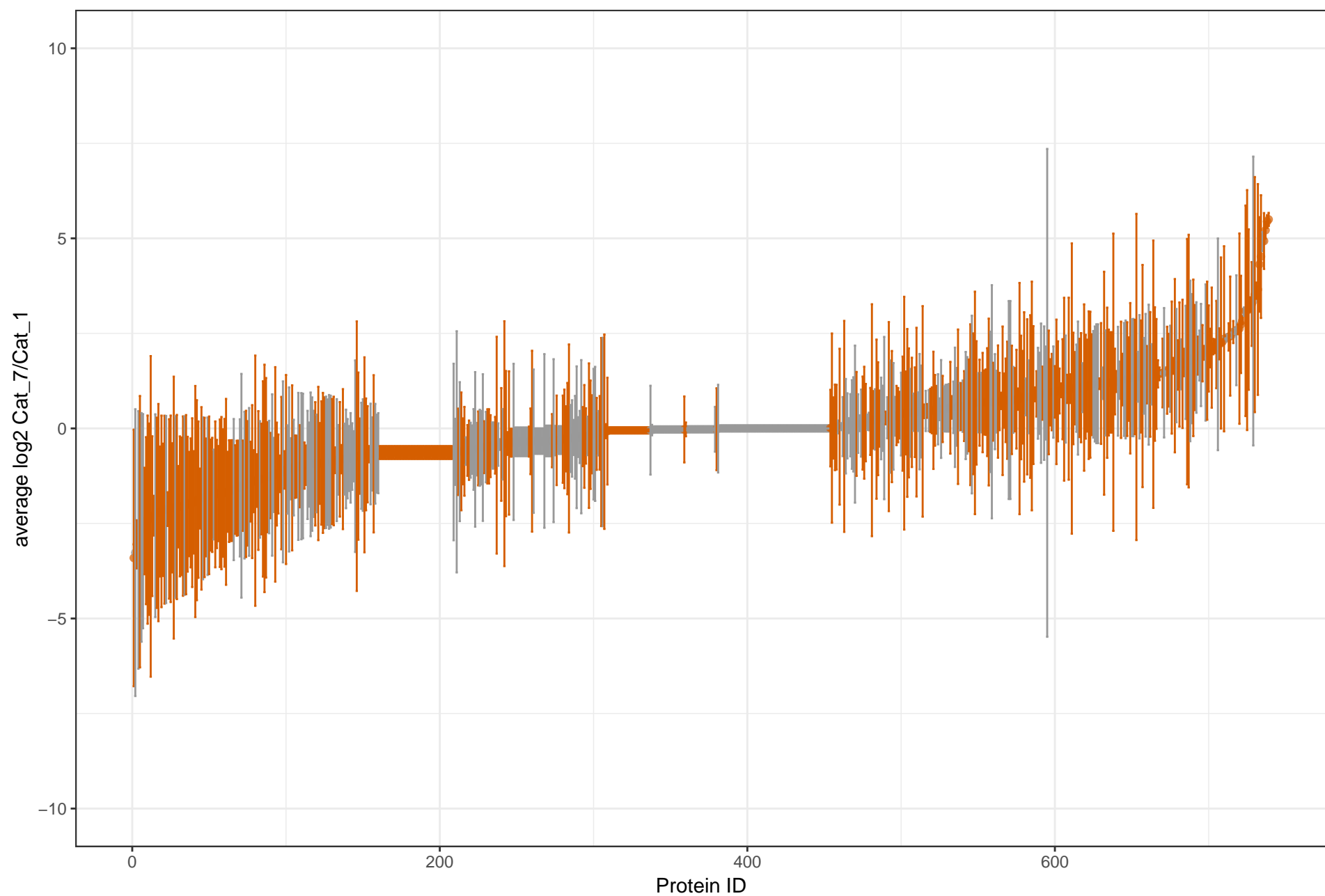

Supplement: Figure 6—source data 1. — Individual data from all figures involving small datasets displayed in individual tabs of this source file. This includes Figures 1B and 2A-F, Figure 3B, Figure 4, Figure 1—figure supplement 1 and Figure 2—figure supplement 1. [file elife-75798-fig6-data1.zip › Flores_Data/AF1_Cat_7.Cat_1-value-ordered-log-ratio_AFCat1.pdf]

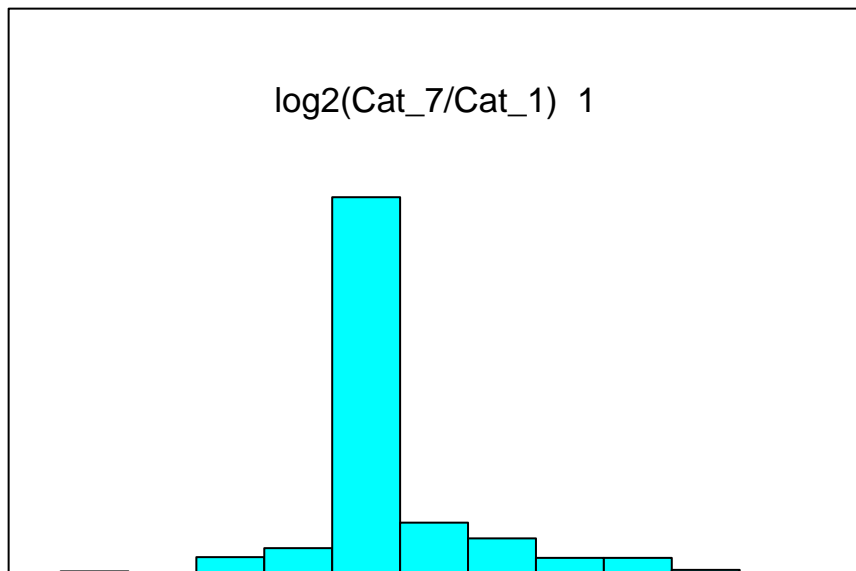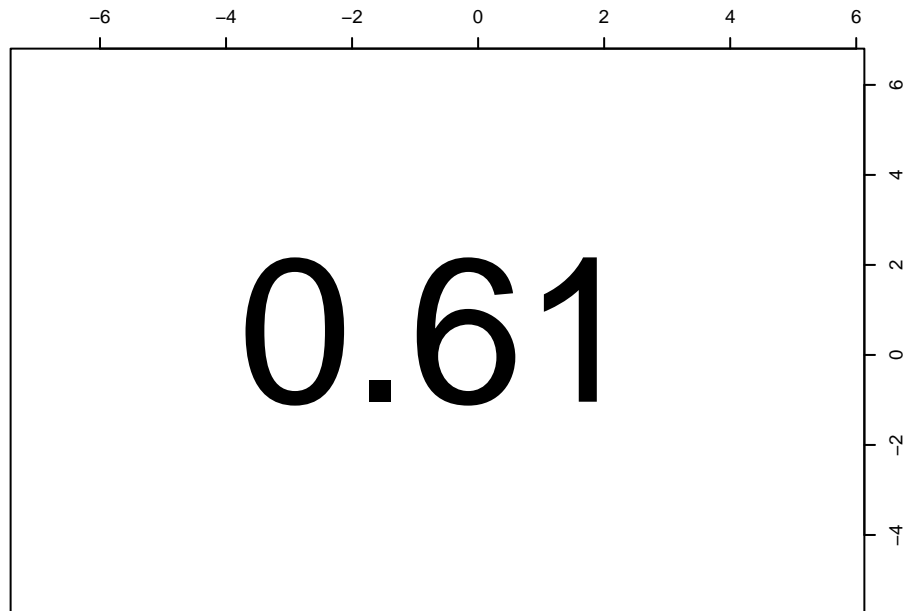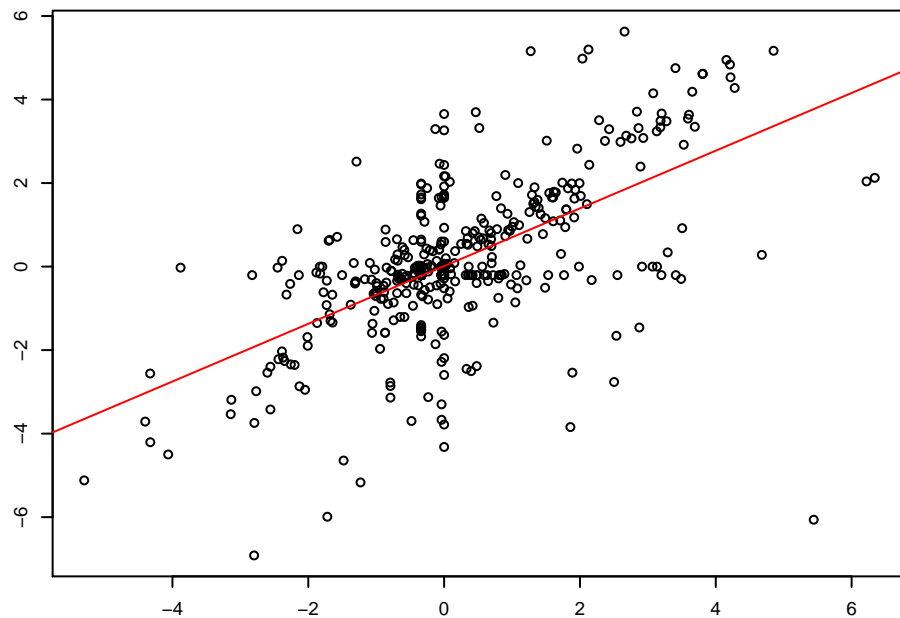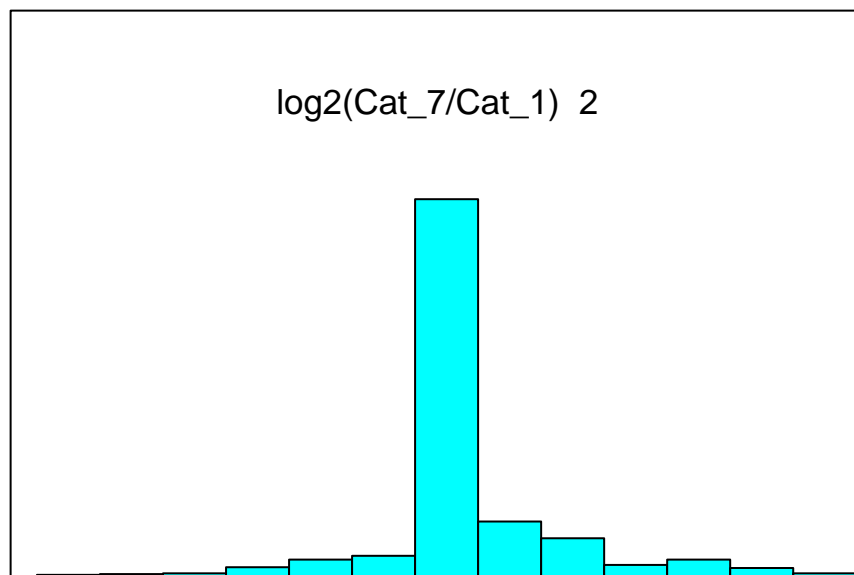

Supplement: Figure 6—source data 1. — Individual data from all figures involving small datasets displayed in individual tabs of this source file. This includes Figures 1B and 2A-F, Figure 3B, Figure 4, Figure 1—figure supplement 1 and Figure 2—figure supplement 1. [file elife-75798-fig6-data1.zip › Flores_Data/AF1_Cat_7.Cat_1-reproducibility_AFCat1.pdf]

MA plot

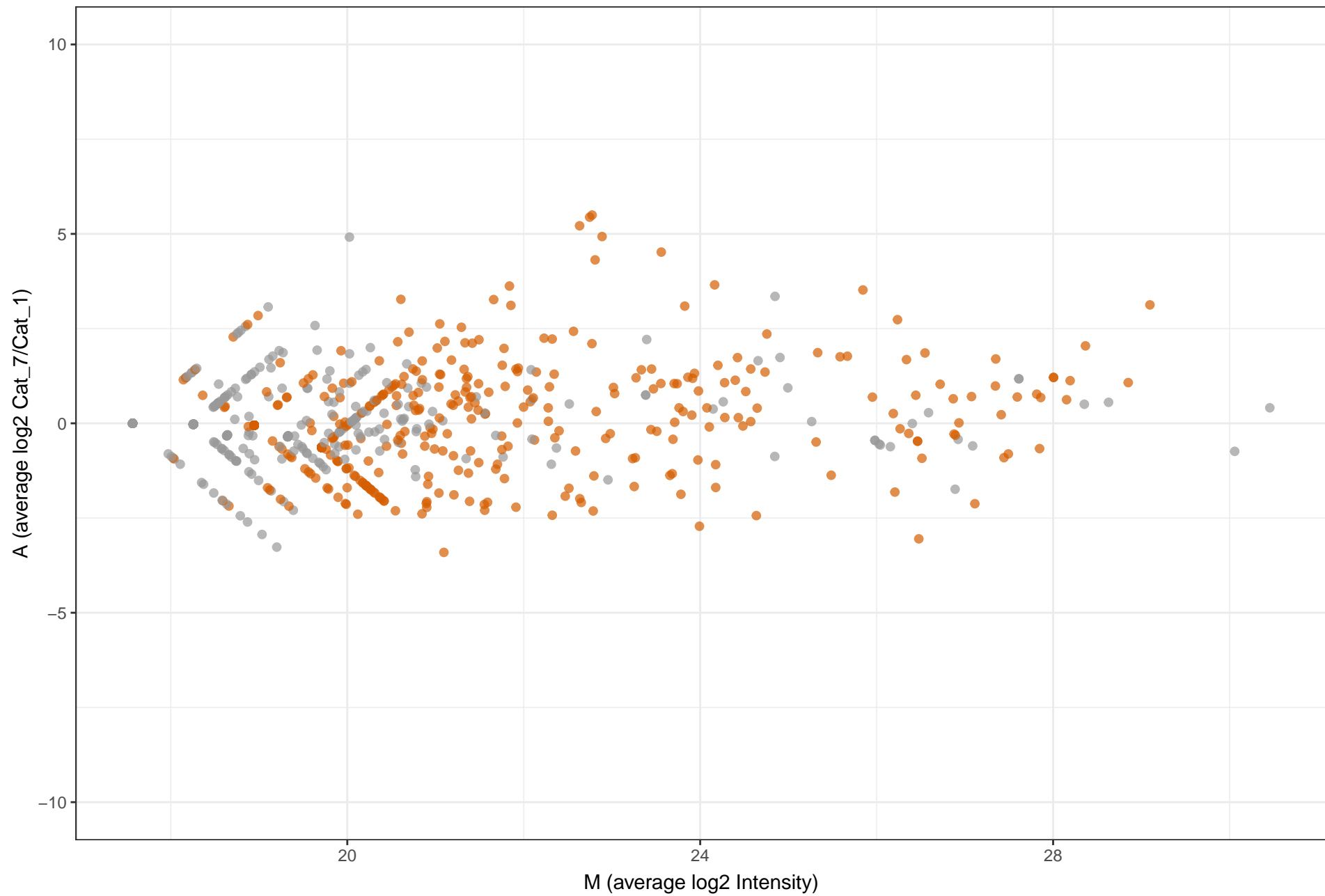

Supplement: Figure 6—source data 1. — Individual data from all figures involving small datasets displayed in individual tabs of this source file. This includes Figures 1B and 2A-F, Figure 3B, Figure 4, Figure 1—figure supplement 1 and Figure 2—figure supplement 1. [file elife-75798-fig6-data1.zip › Flores_Data/AF1_Cat_7.Cat_1-MA_AFCat1.pdf]

**P-value vs Fold change**

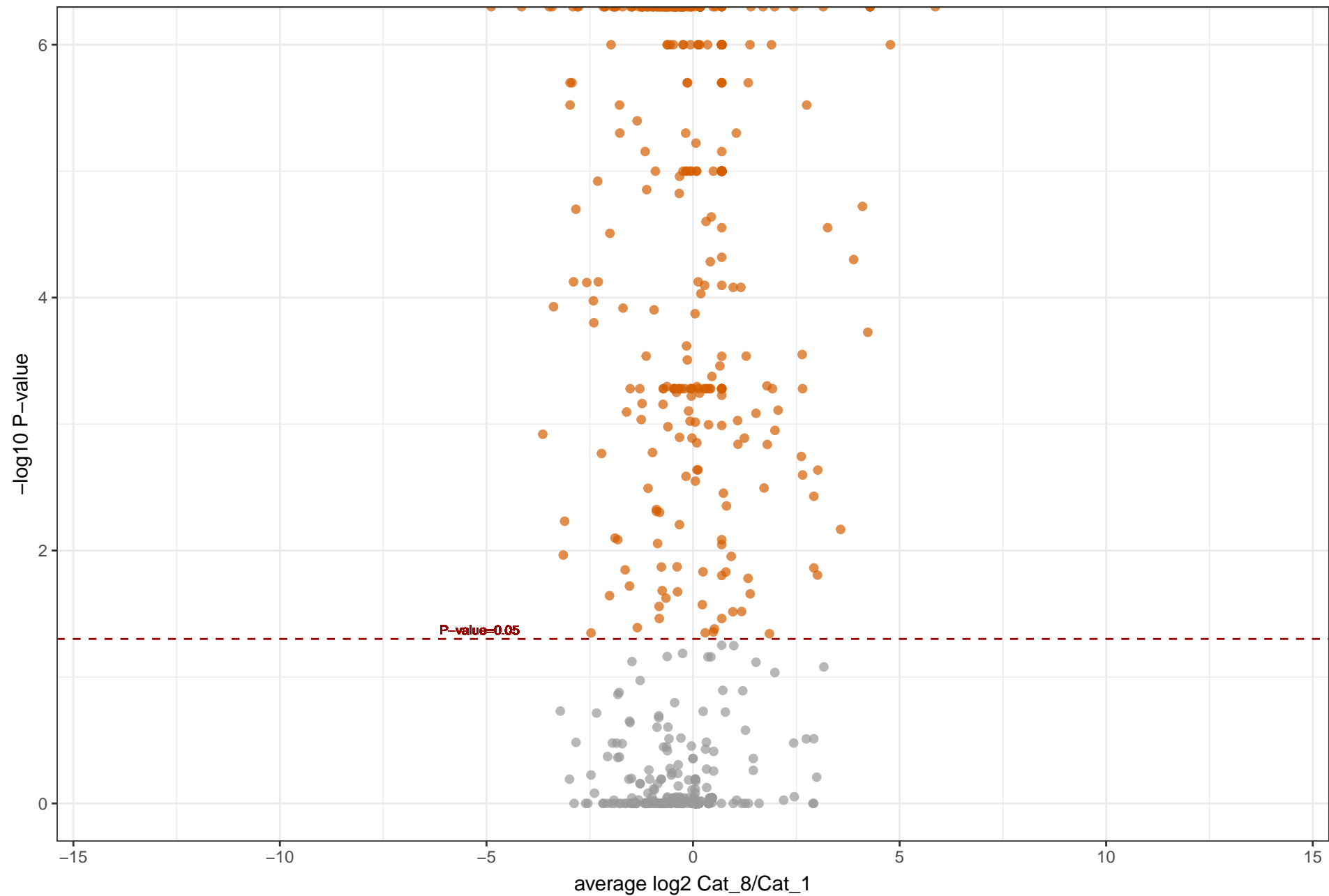

Supplement: Figure 6—source data 1. — Individual data from all figures involving small datasets displayed in individual tabs of this source file. This includes Figures 1B and 2A-F, Figure 3B, Figure 4, Figure 1—figure supplement 1 and Figure 2—figure supplement 1. [file elife-75798-fig6-data1.zip › Flores_Data/AF1_Cat_8.Cat_1-volcano_AFCat1.pdf]

Value-ordered fold change

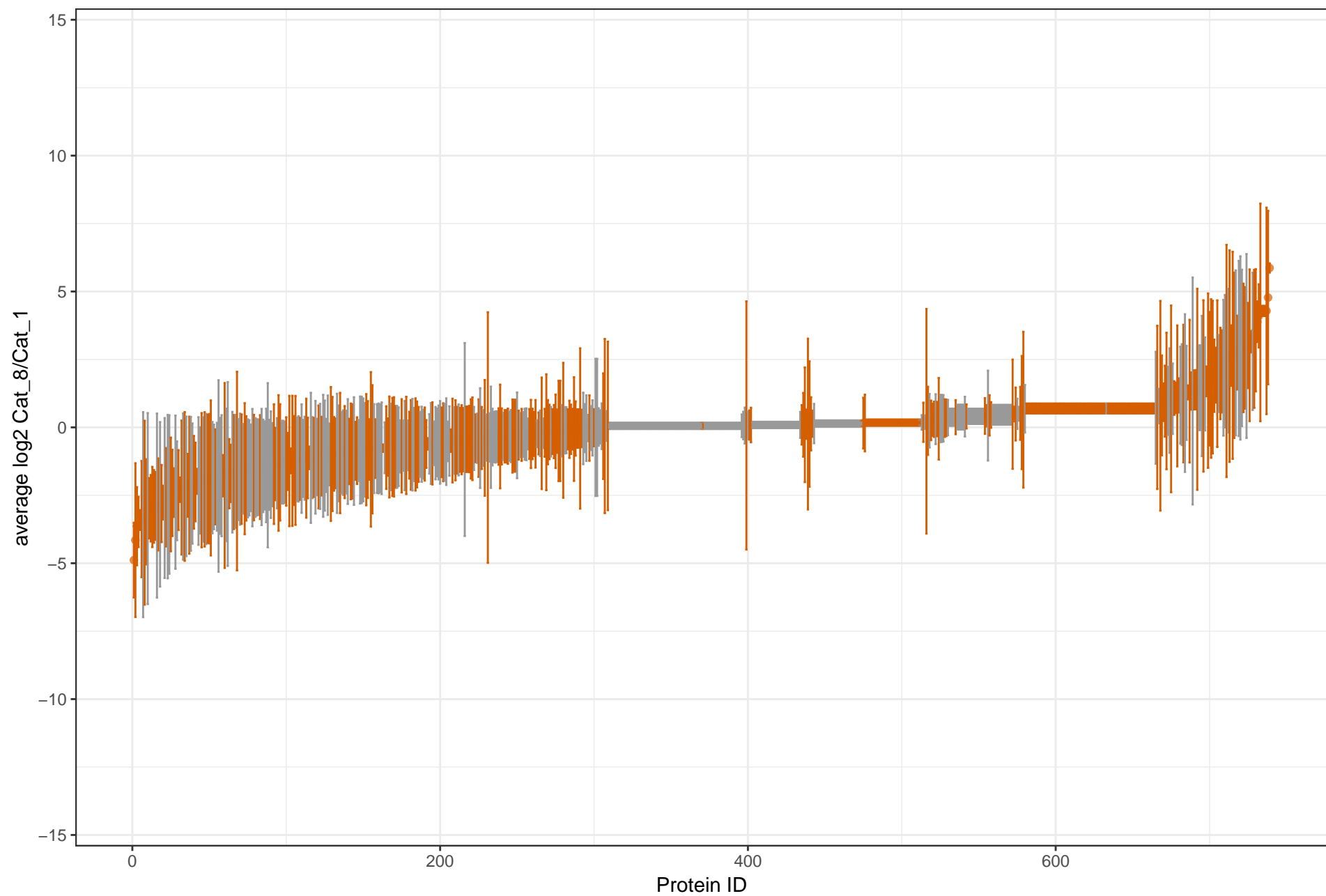

Supplement: Figure 6—source data 1. — Individual data from all figures involving small datasets displayed in individual tabs of this source file. This includes Figures 1B and 2A-F, Figure 3B, Figure 4, Figure 1—figure supplement 1 and Figure 2—figure supplement 1. [file elife-75798-fig6-data1.zip › Flores_Data/AF1_Cat_8.Cat_1-value-ordered-log-ratio_AFCat1.pdf]

MA plot

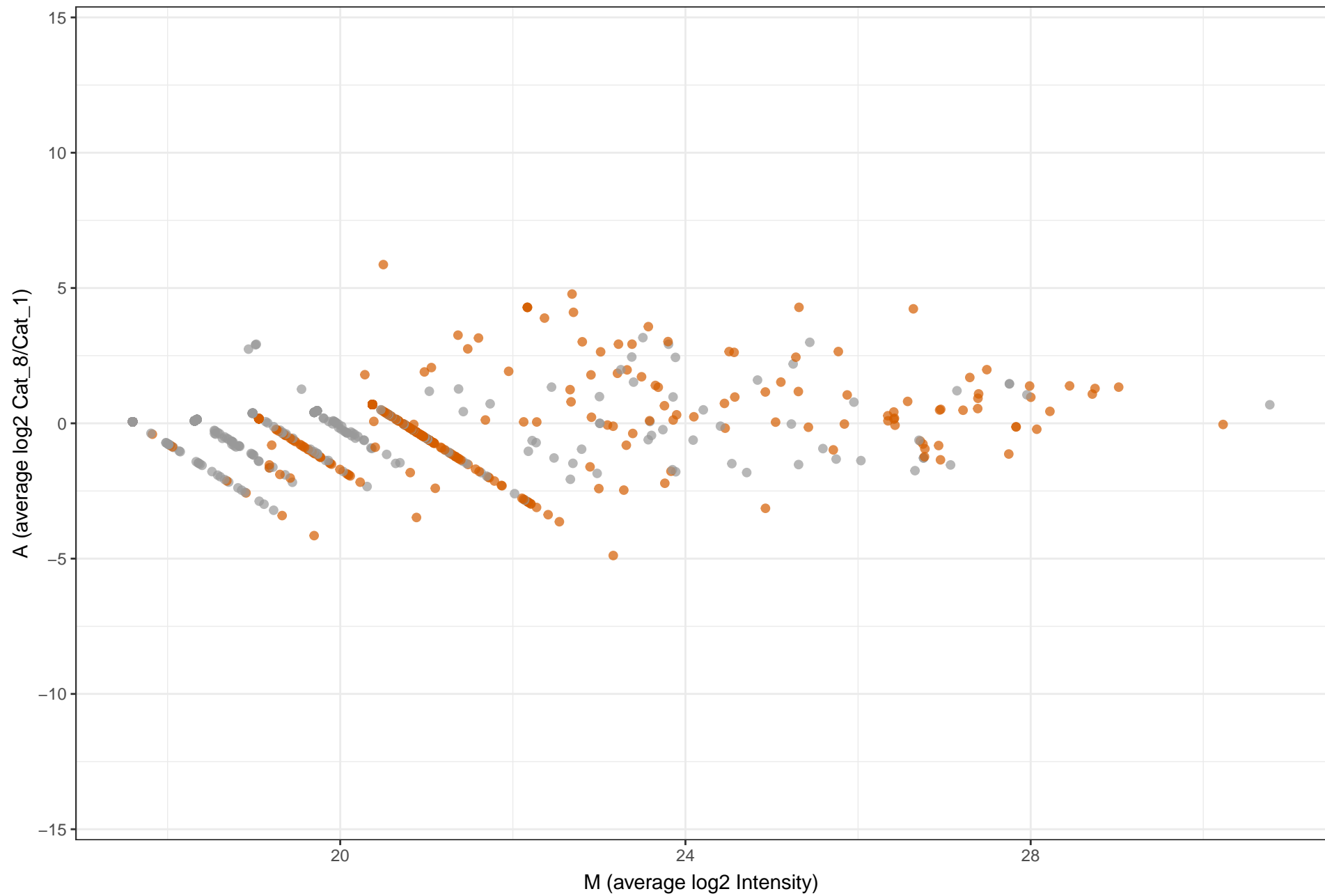

Supplement: Figure 6—source data 1. — Individual data from all figures involving small datasets displayed in individual tabs of this source file. This includes Figures 1B and 2A-F, Figure 3B, Figure 4, Figure 1—figure supplement 1 and Figure 2—figure supplement 1. [file elife-75798-fig6-data1.zip › Flores_Data/AF1_Cat_8.Cat_1-MA_AFCat1.pdf]

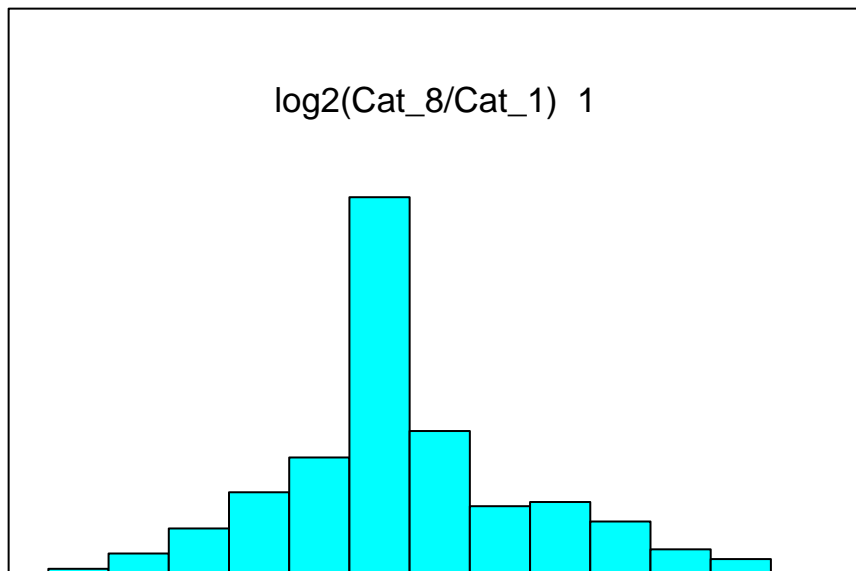

-5 0 5

6  
4  
2  
0  
-2  
-4  
-6

0.81

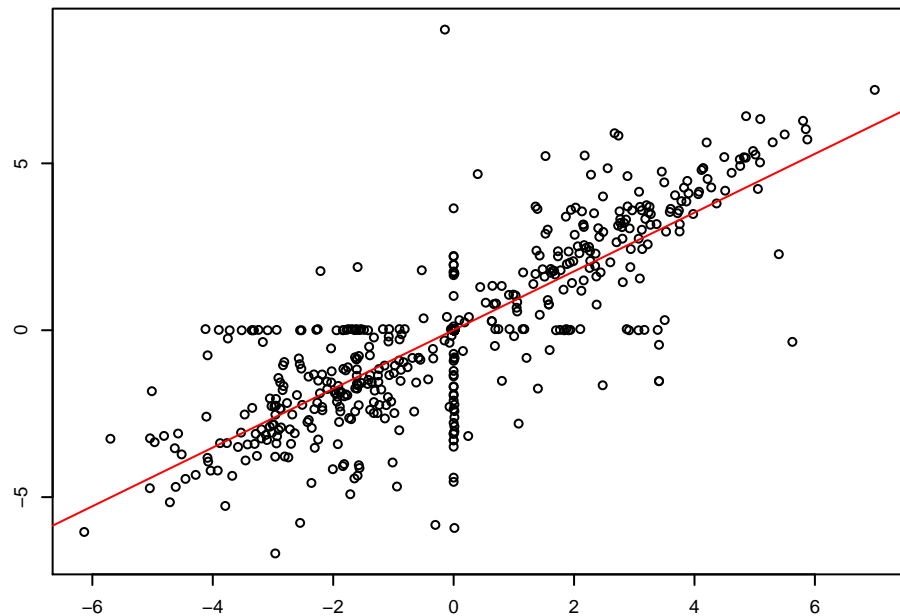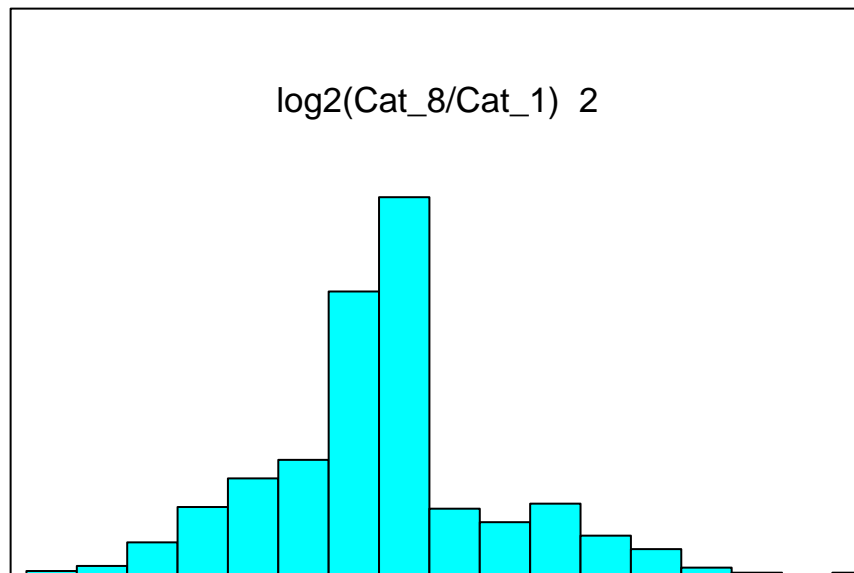

Supplement: Figure 6—source data 1. — Individual data from all figures involving small datasets displayed in individual tabs of this source file. This includes Figures 1B and 2A-F, Figure 3B, Figure 4, Figure 1—figure supplement 1 and Figure 2—figure supplement 1. [file elife-75798-fig6-data1.zip › Flores_Data/AF1_Cat_8.Cat_1-reproducibility_AFCat1.pdf]

P-value vs Fold change

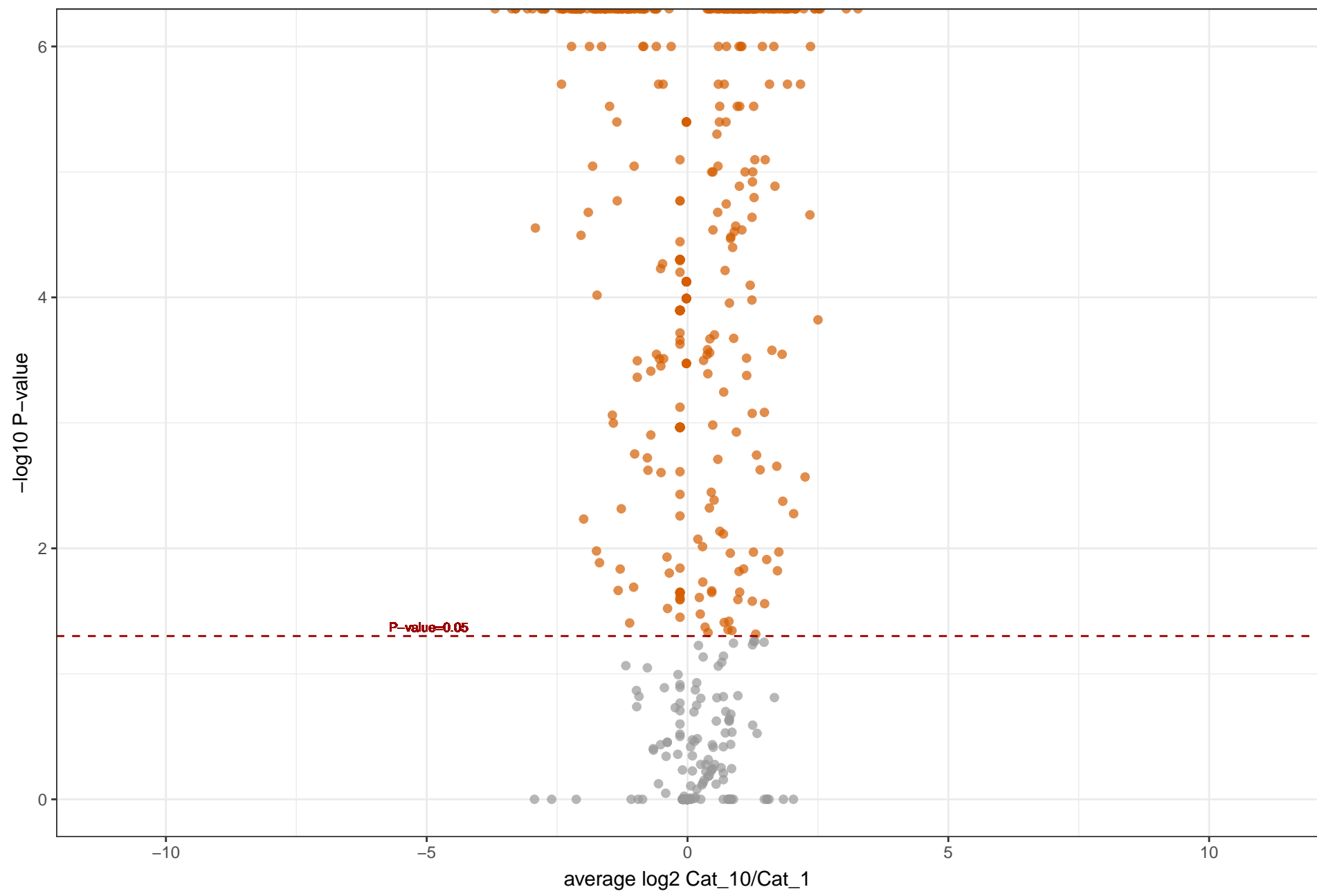

Supplement: Figure 6—source data 1. — Individual data from all figures involving small datasets displayed in individual tabs of this source file. This includes Figures 1B and 2A-F, Figure 3B, Figure 4, Figure 1—figure supplement 1 and Figure 2—figure supplement 1. [file elife-75798-fig6-data1.zip › Flores_Data/AF1_Cat_10.Cat_1-volcano_AFCat1.pdf]

Value-ordered fold change

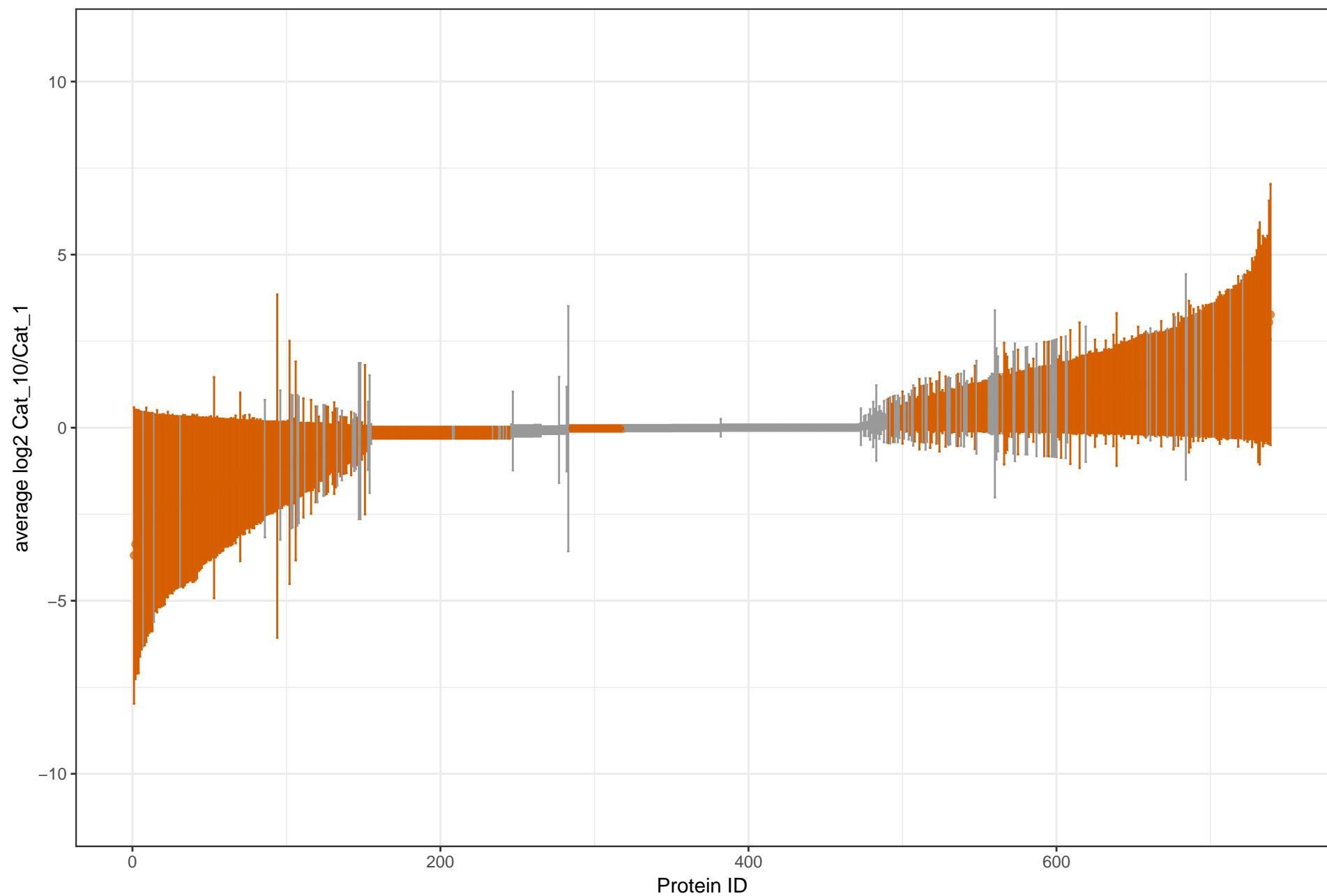

Supplement: Figure 6—source data 1. — Individual data from all figures involving small datasets displayed in individual tabs of this source file. This includes Figures 1B and 2A-F, Figure 3B, Figure 4, Figure 1—figure supplement 1 and Figure 2—figure supplement 1. [file elife-75798-fig6-data1.zip › Flores_Data/AF1_Cat_10.Cat_1-value-ordered-log-ratio_AFCat1.pdf]

MA plot

A (average log2 Cat\_10/Cat\_1)

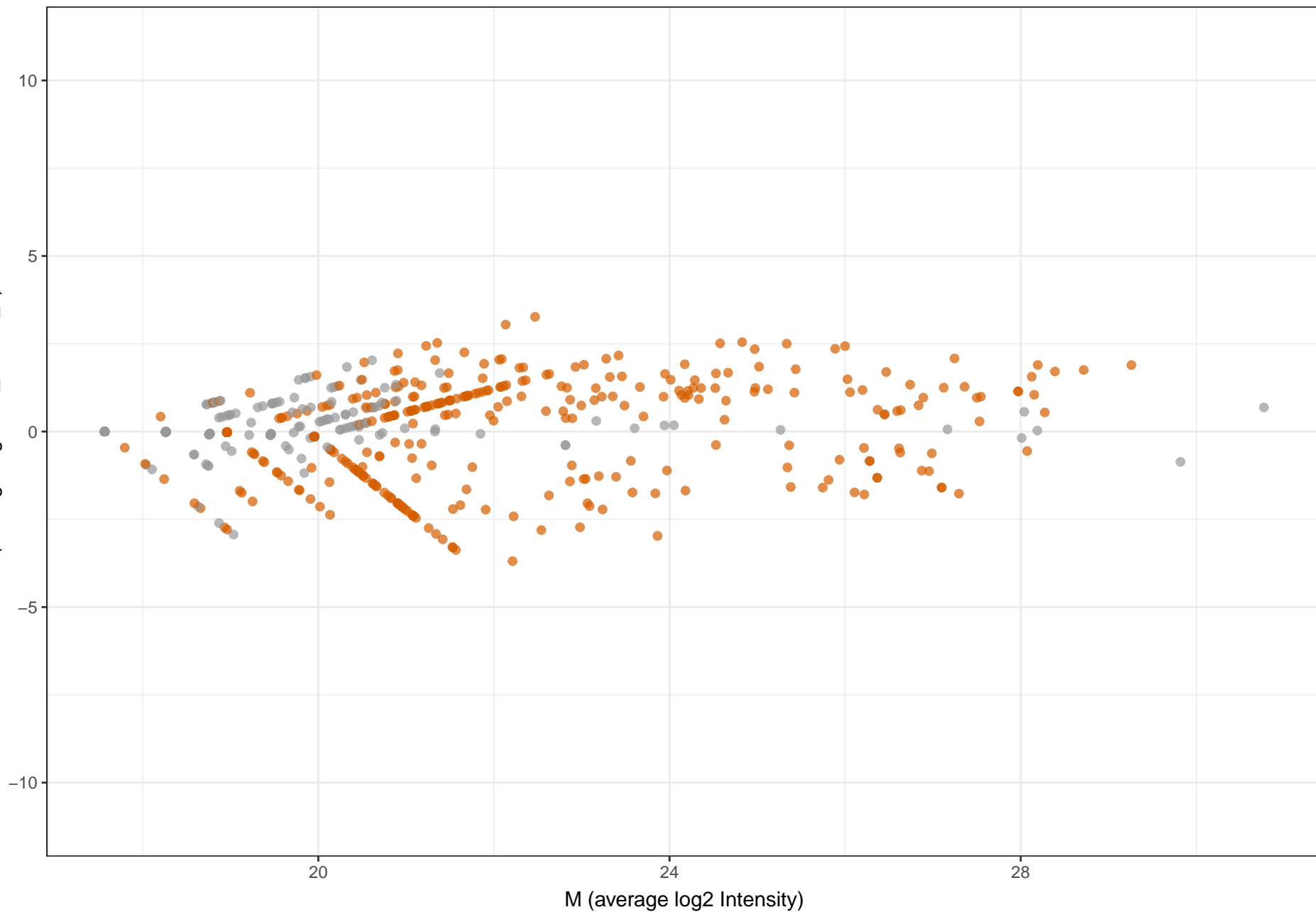

Supplement: Figure 6—source data 1. — Individual data from all figures involving small datasets displayed in individual tabs of this source file. This includes Figures 1B and 2A-F, Figure 3B, Figure 4, Figure 1—figure supplement 1 and Figure 2—figure supplement 1. [file elife-75798-fig6-data1.zip › Flores_Data/AF1_Cat_10.Cat_1-MA_AFCat1.pdf]

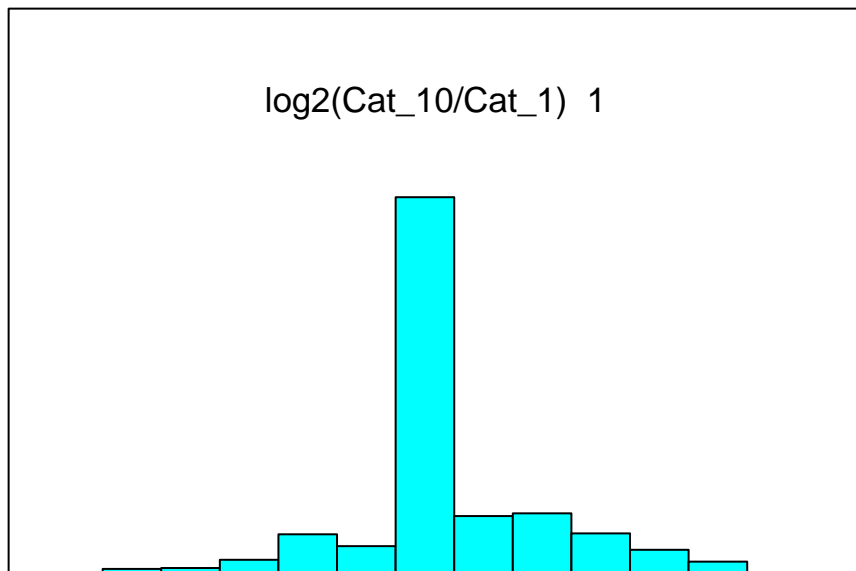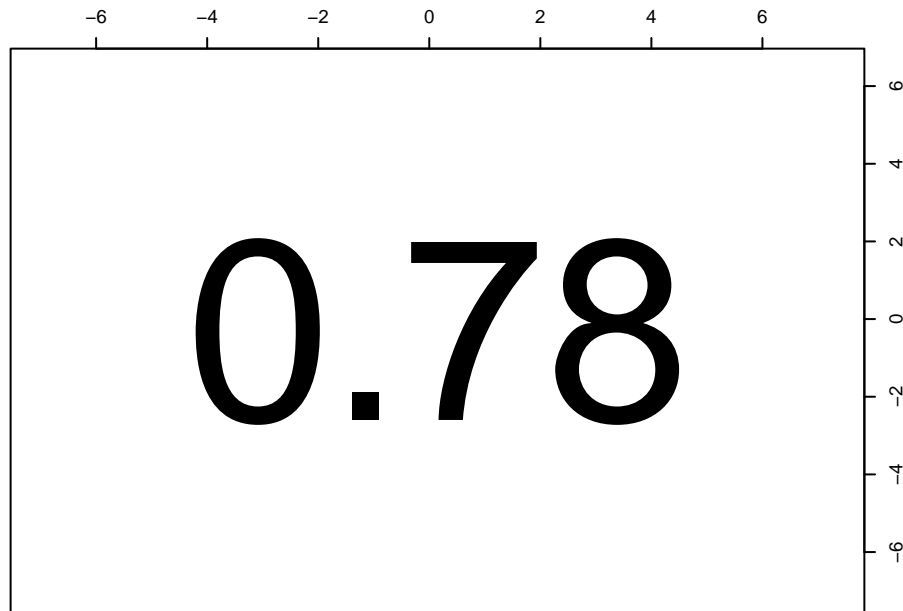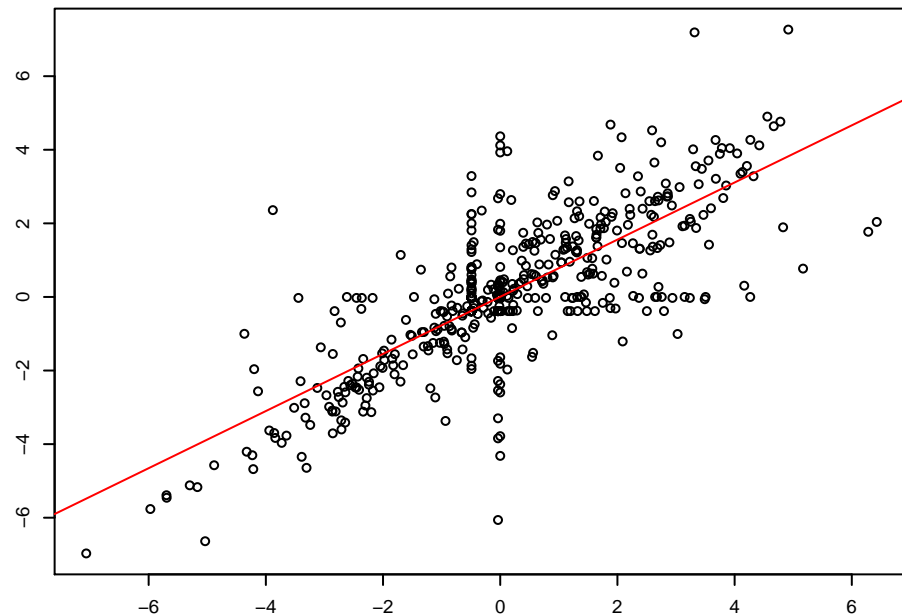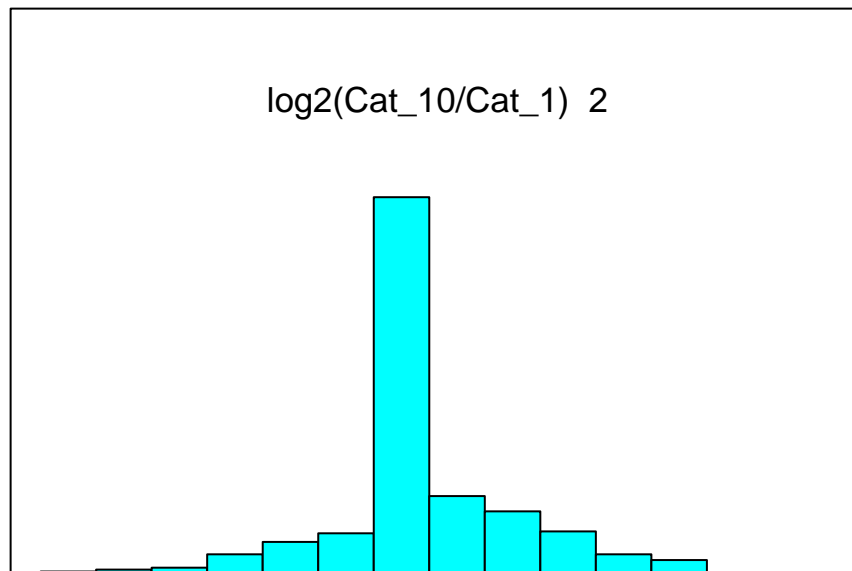

Supplement: Figure 6—source data 1. — Individual data from all figures involving small datasets displayed in individual tabs of this source file. This includes Figures 1B and 2A-F, Figure 3B, Figure 4, Figure 1—figure supplement 1 and Figure 2—figure supplement 1. [file elife-75798-fig6-data1.zip › Flores_Data/AF1_Cat_10.Cat_1-reproducibility_AFCat1.pdf]

**P-value vs Fold change**

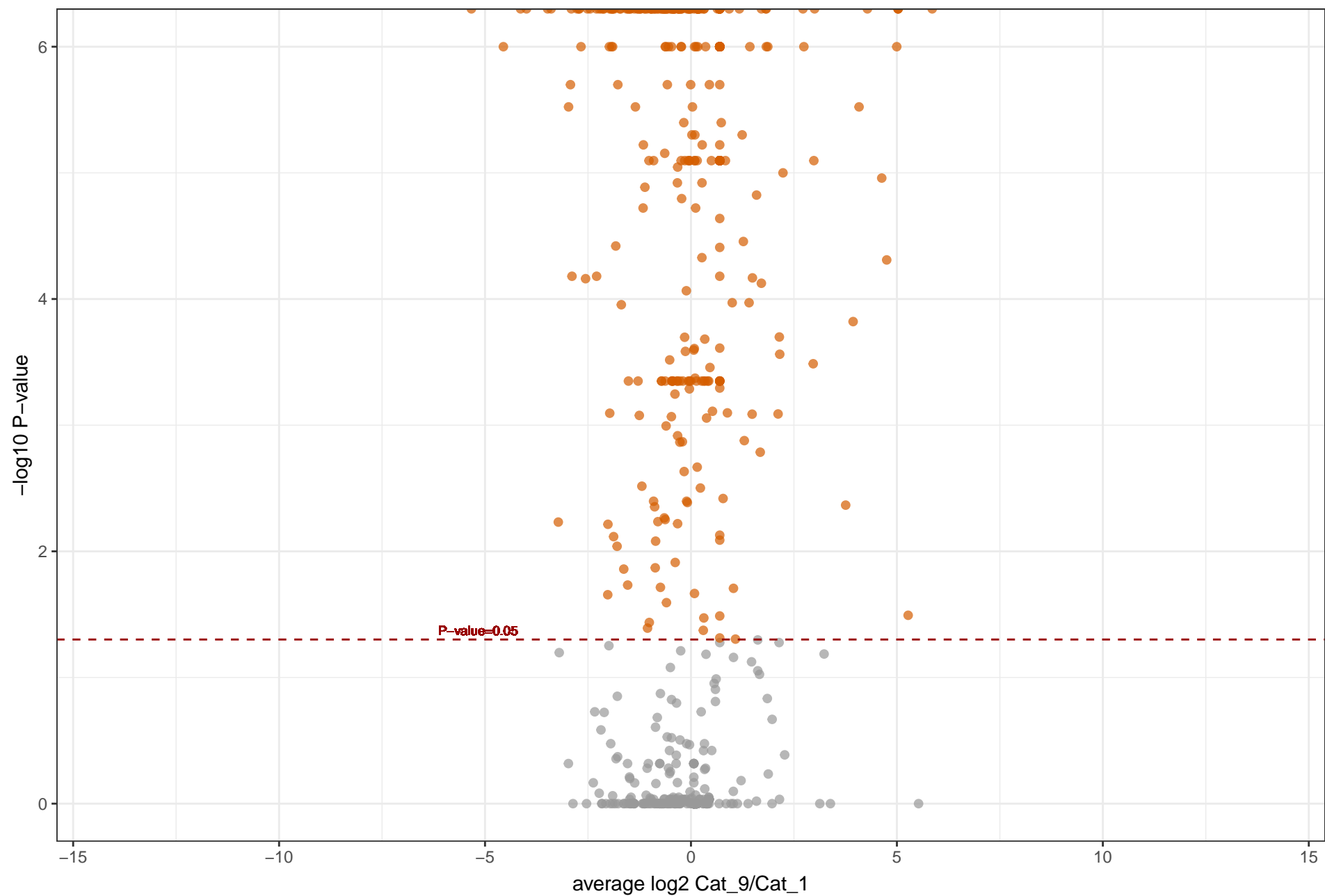

Supplement: Figure 6—source data 1. — Individual data from all figures involving small datasets displayed in individual tabs of this source file. This includes Figures 1B and 2A-F, Figure 3B, Figure 4, Figure 1—figure supplement 1 and Figure 2—figure supplement 1. [file elife-75798-fig6-data1.zip › Flores_Data/AF1_Cat_9.Cat_1-volcano_AFCat1.pdf]

Value-ordered fold change

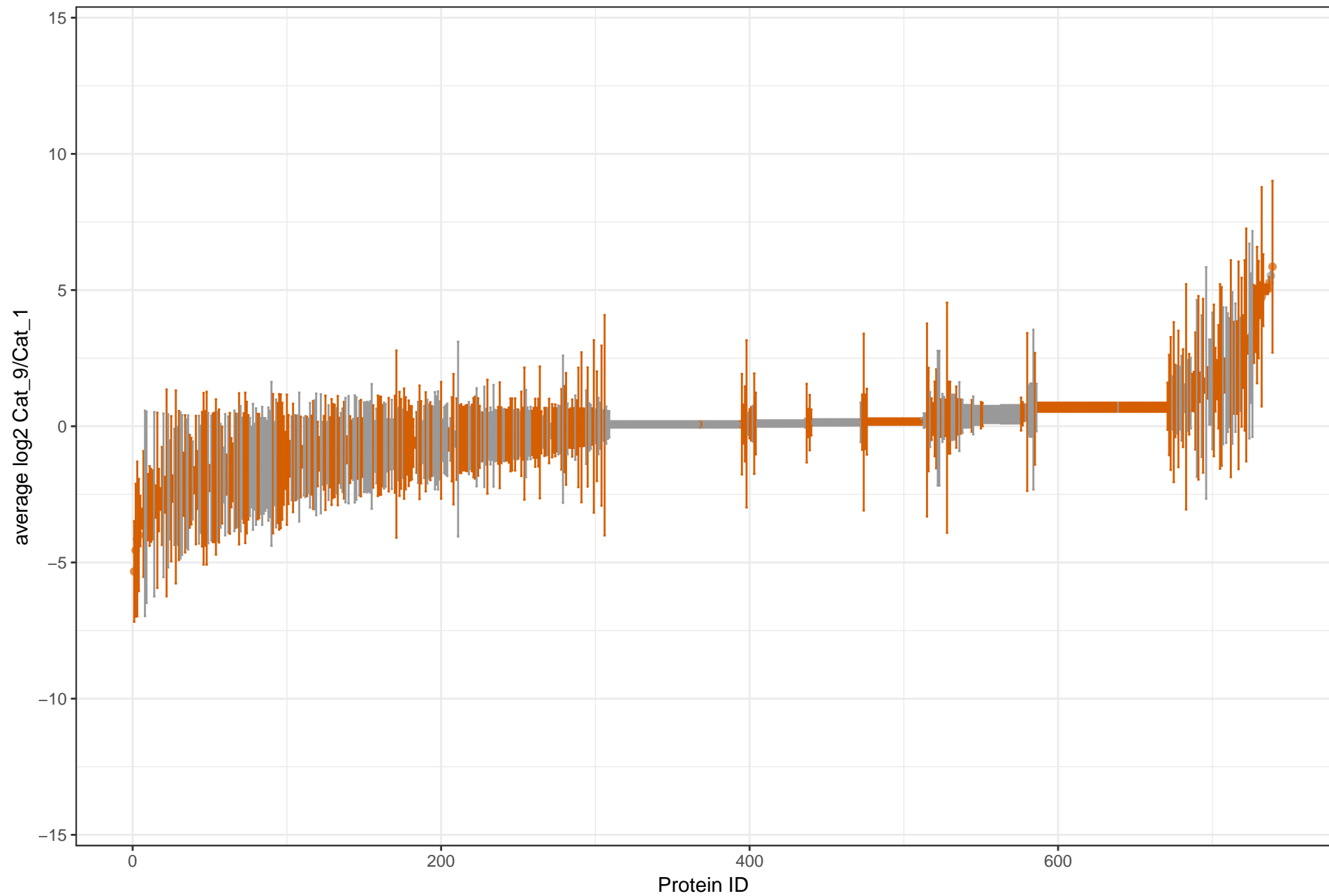

Supplement: Figure 6—source data 1. — Individual data from all figures involving small datasets displayed in individual tabs of this source file. This includes Figures 1B and 2A-F, Figure 3B, Figure 4, Figure 1—figure supplement 1 and Figure 2—figure supplement 1. [file elife-75798-fig6-data1.zip › Flores_Data/AF1_Cat_9.Cat_1-value-ordered-log-ratio_AFCat1.pdf]

P-value vs Fold change

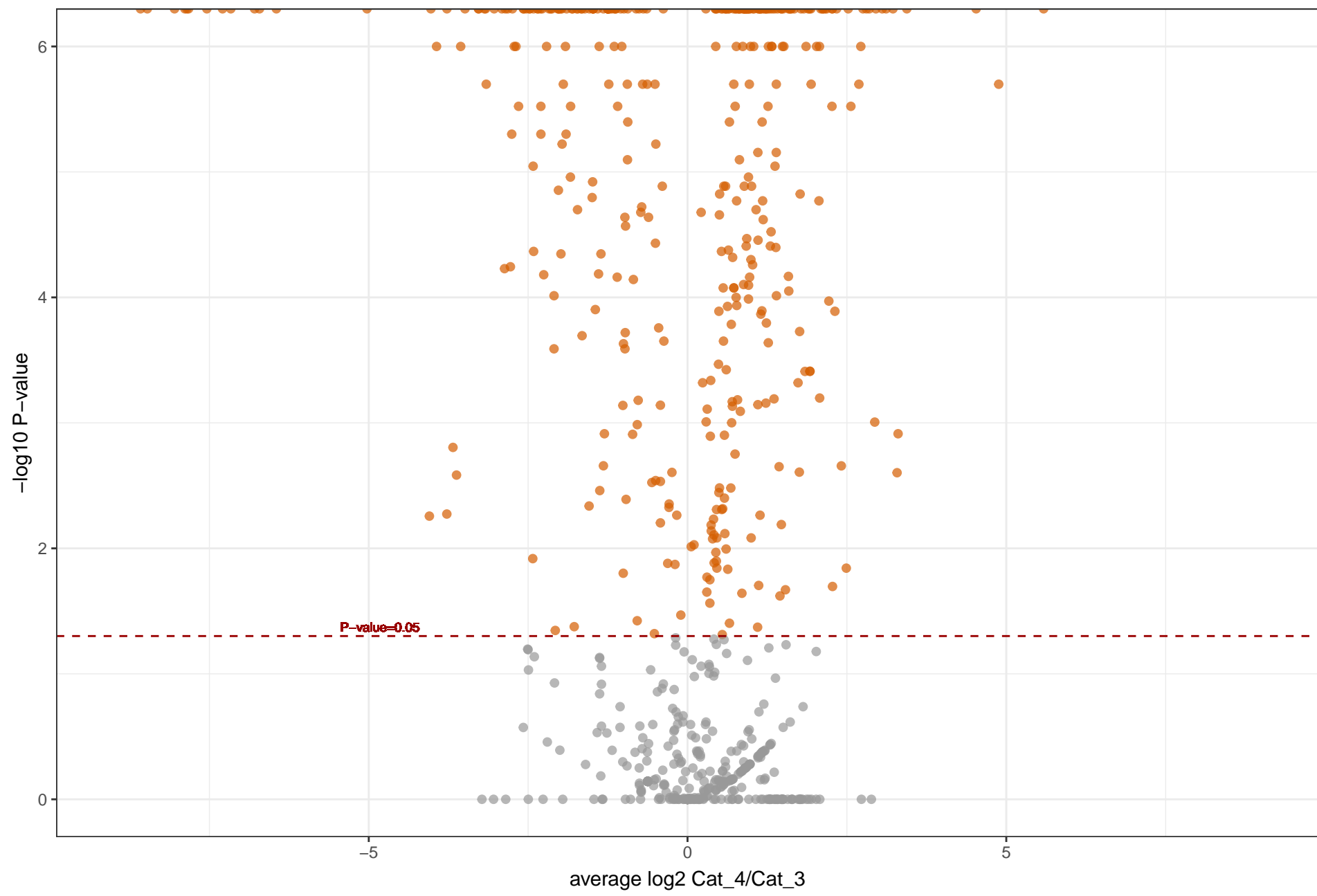

Supplement: Figure 6—source data 1. — Individual data from all figures involving small datasets displayed in individual tabs of this source file. This includes Figures 1B and 2A-F, Figure 3B, Figure 4, Figure 1—figure supplement 1 and Figure 2—figure supplement 1. [file elife-75798-fig6-data1.zip › Flores_Data/AF1_Cat_4.Cat_3-volcano_AFCat1.pdf]

MA plot

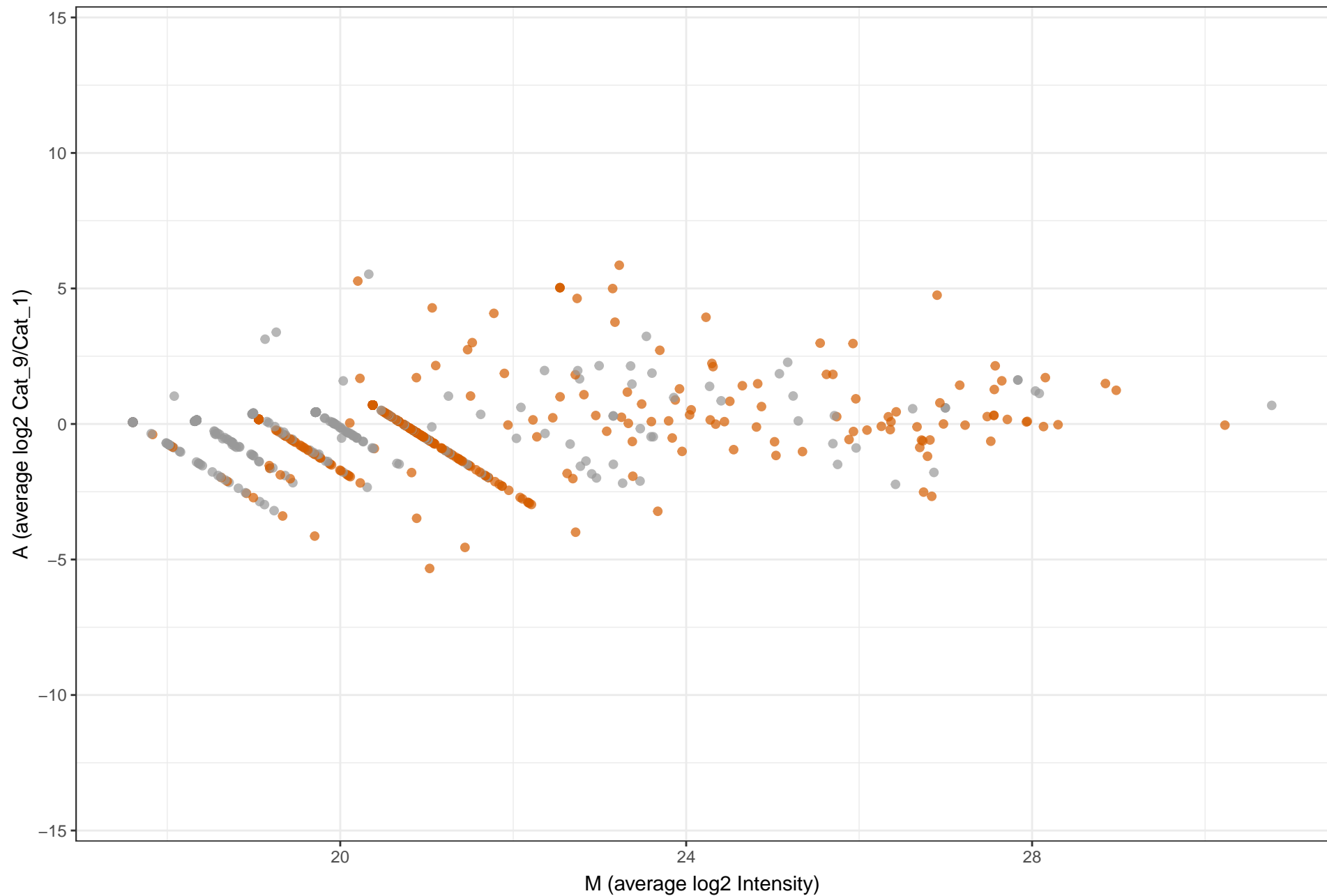

Supplement: Figure 6—source data 1. — Individual data from all figures involving small datasets displayed in individual tabs of this source file. This includes Figures 1B and 2A-F, Figure 3B, Figure 4, Figure 1—figure supplement 1 and Figure 2—figure supplement 1. [file elife-75798-fig6-data1.zip › Flores_Data/AF1_Cat_9.Cat_1-MA_AFCat1.pdf]

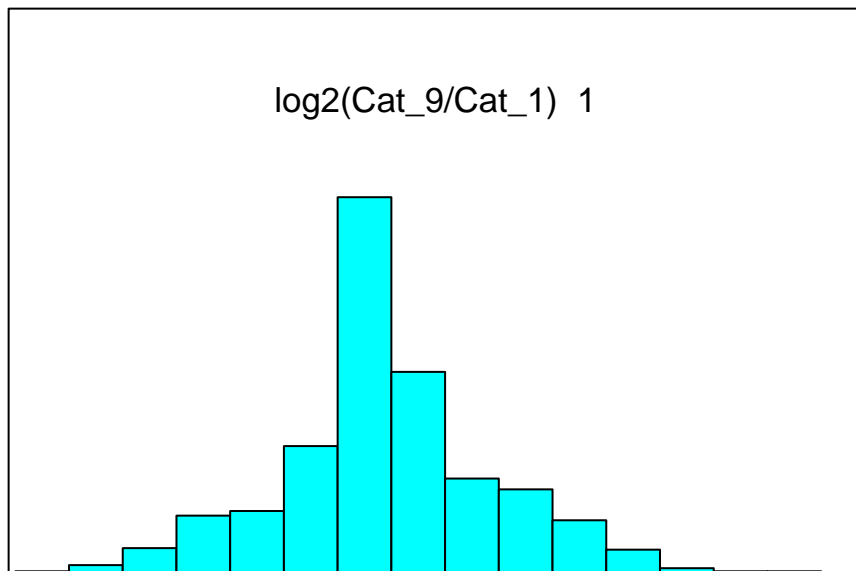

-5 0 5

5  
0  
-5

0.78

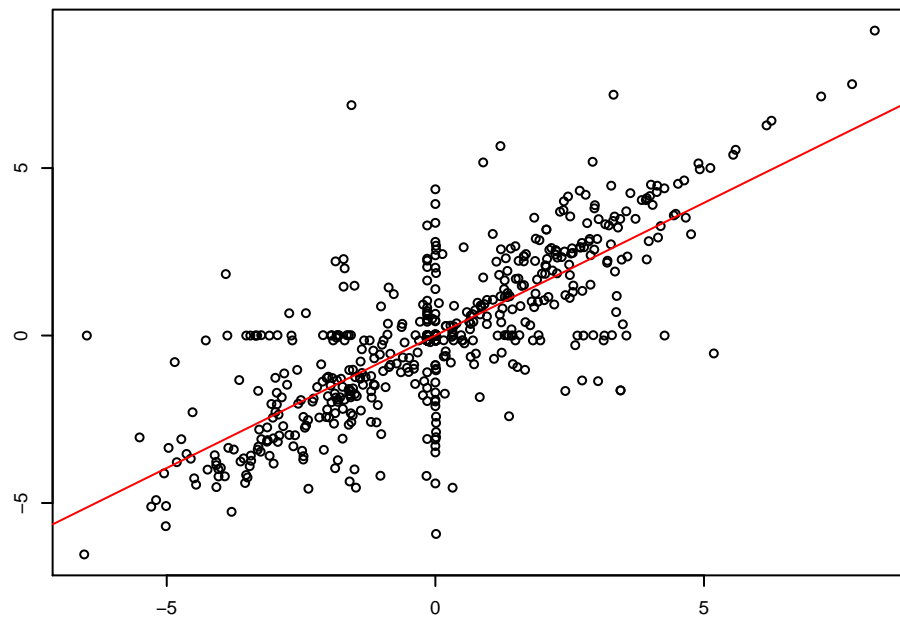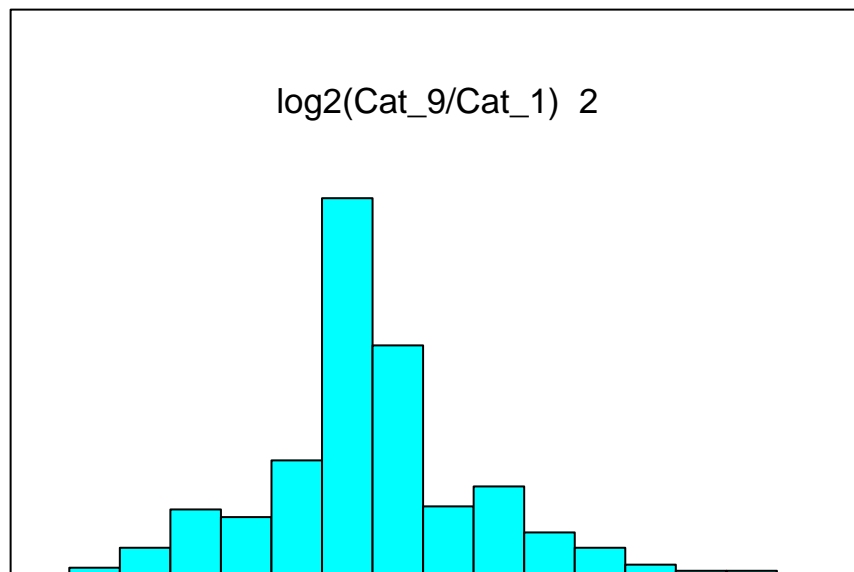

Supplement: Figure 6—source data 1. — Individual data from all figures involving small datasets displayed in individual tabs of this source file. This includes Figures 1B and 2A-F, Figure 3B, Figure 4, Figure 1—figure supplement 1 and Figure 2—figure supplement 1. [file elife-75798-fig6-data1.zip › Flores_Data/AF1_Cat_9.Cat_1-reproducibility_AFCat1.pdf]

Value-ordered fold change

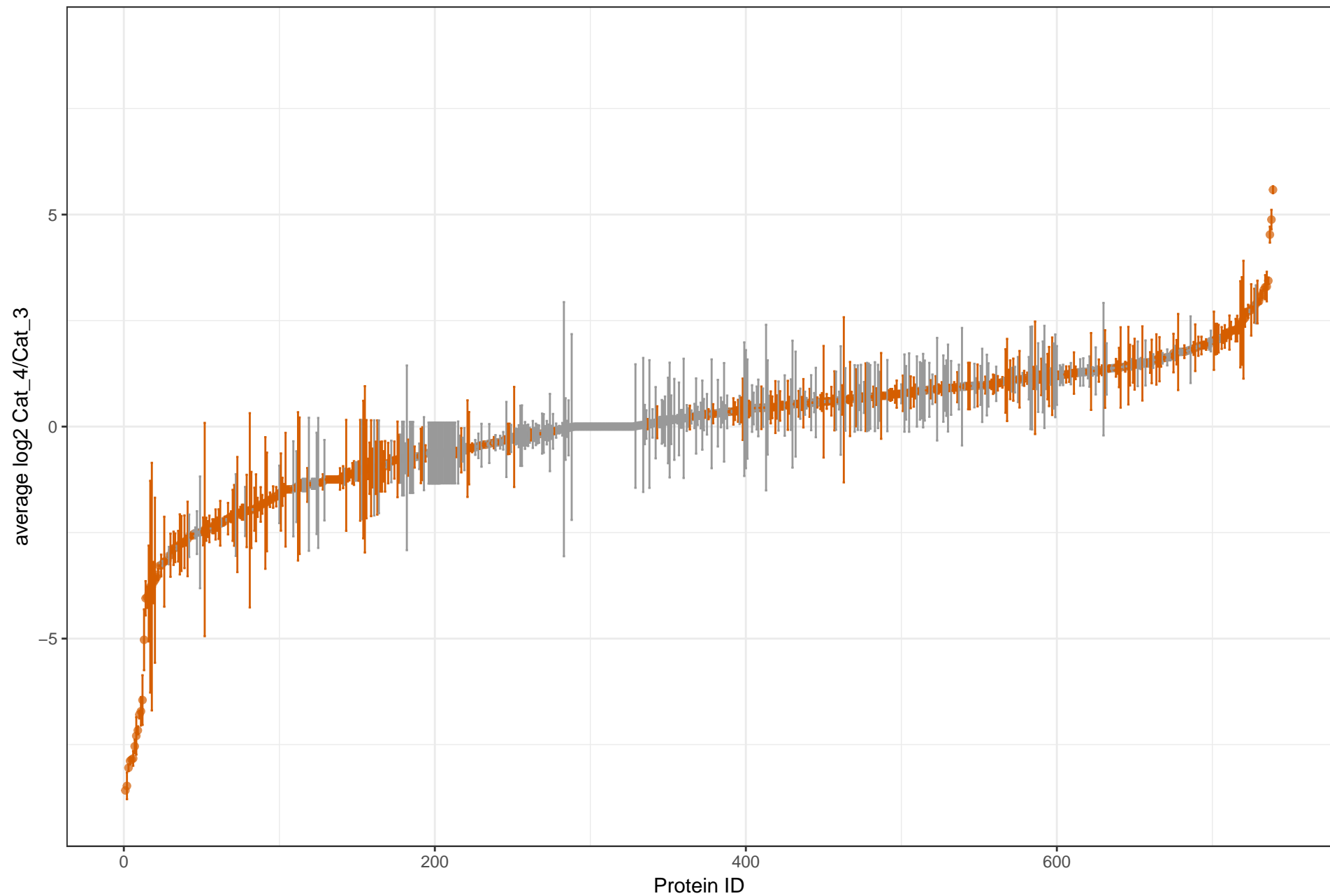

Supplement: Figure 6—source data 1. — Individual data from all figures involving small datasets displayed in individual tabs of this source file. This includes Figures 1B and 2A-F, Figure 3B, Figure 4, Figure 1—figure supplement 1 and Figure 2—figure supplement 1. [file elife-75798-fig6-data1.zip › Flores_Data/AF1_Cat_4.Cat_3-value-ordered-log-ratio_AFCat1.pdf]

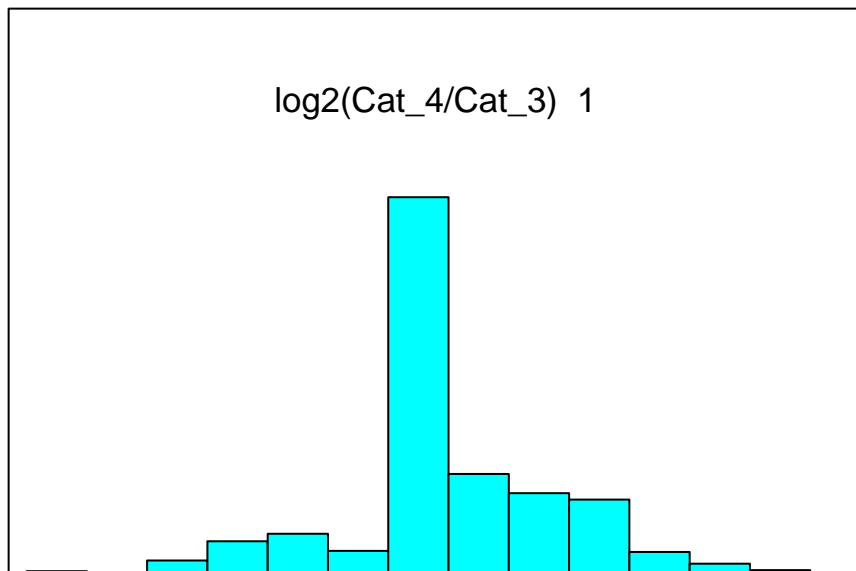

-6 -4 -2 0 2 4 6

6  
4  
2  
0  
-2  
-4  
-6

0.81

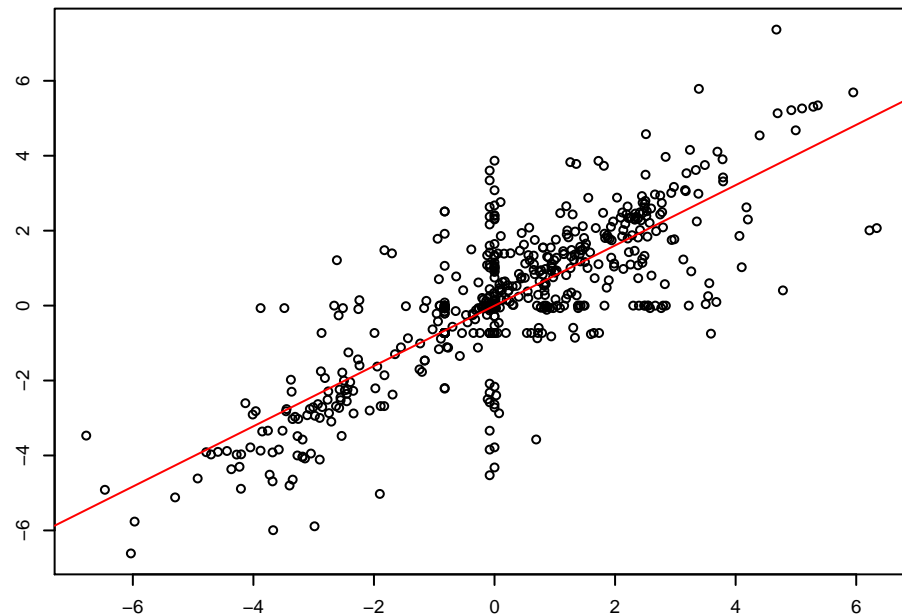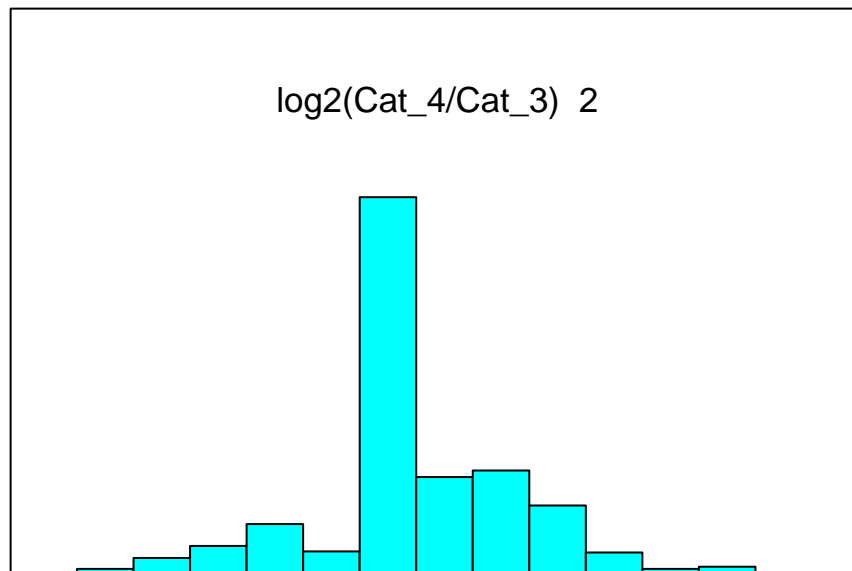

Supplement: Figure 6—source data 1. — Individual data from all figures involving small datasets displayed in individual tabs of this source file. This includes Figures 1B and 2A-F, Figure 3B, Figure 4, Figure 1—figure supplement 1 and Figure 2—figure supplement 1. [file elife-75798-fig6-data1.zip › Flores_Data/AF1_Cat_4.Cat_3-reproducibility_AFCat1.pdf]

MA plot

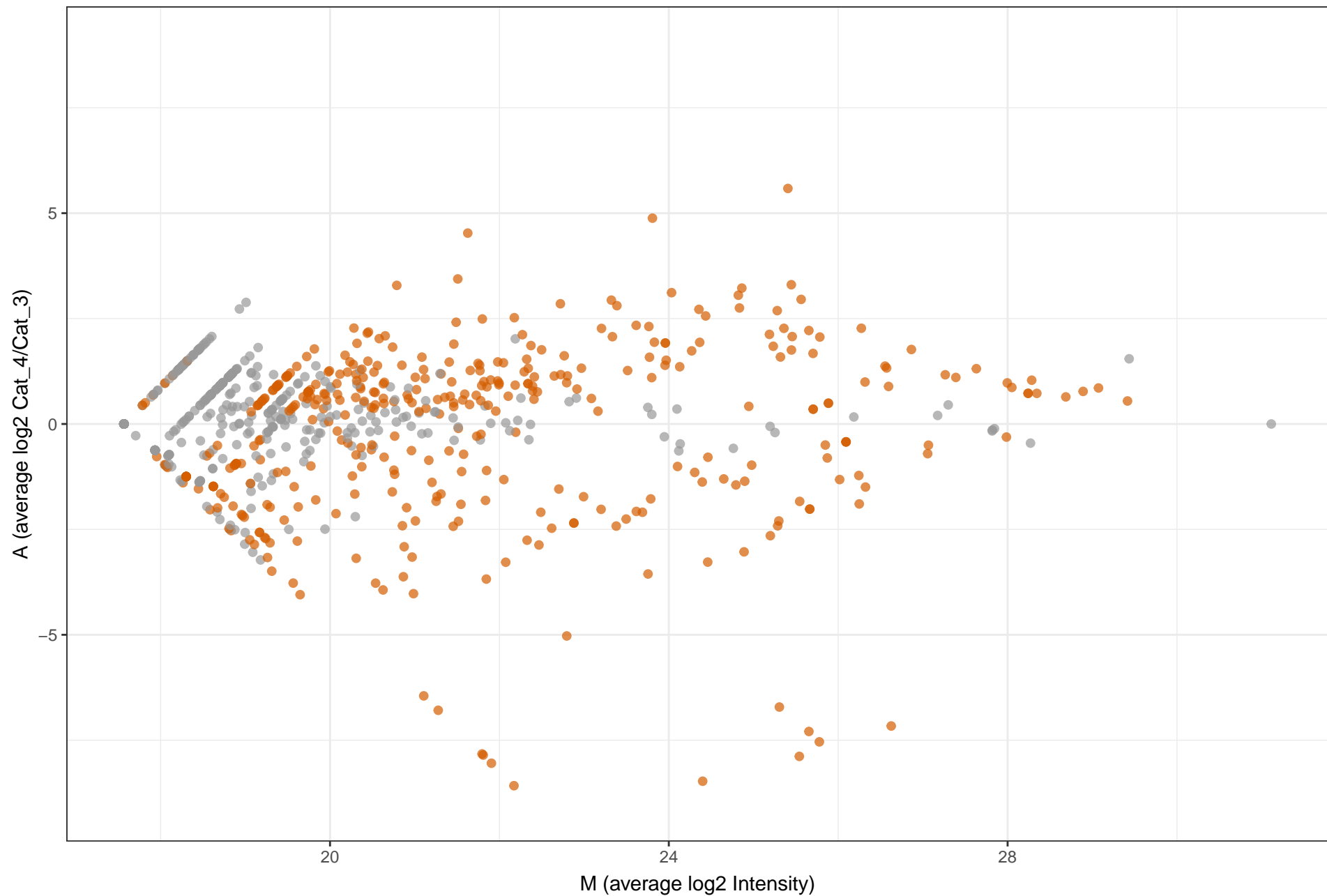

Supplement: Figure 6—source data 1. — Individual data from all figures involving small datasets displayed in individual tabs of this source file. This includes Figures 1B and 2A-F, Figure 3B, Figure 4, Figure 1—figure supplement 1 and Figure 2—figure supplement 1. [file elife-75798-fig6-data1.zip › Flores_Data/AF1_Cat_4.Cat_3-MA_AFCat1.pdf]

P-value vs Fold change

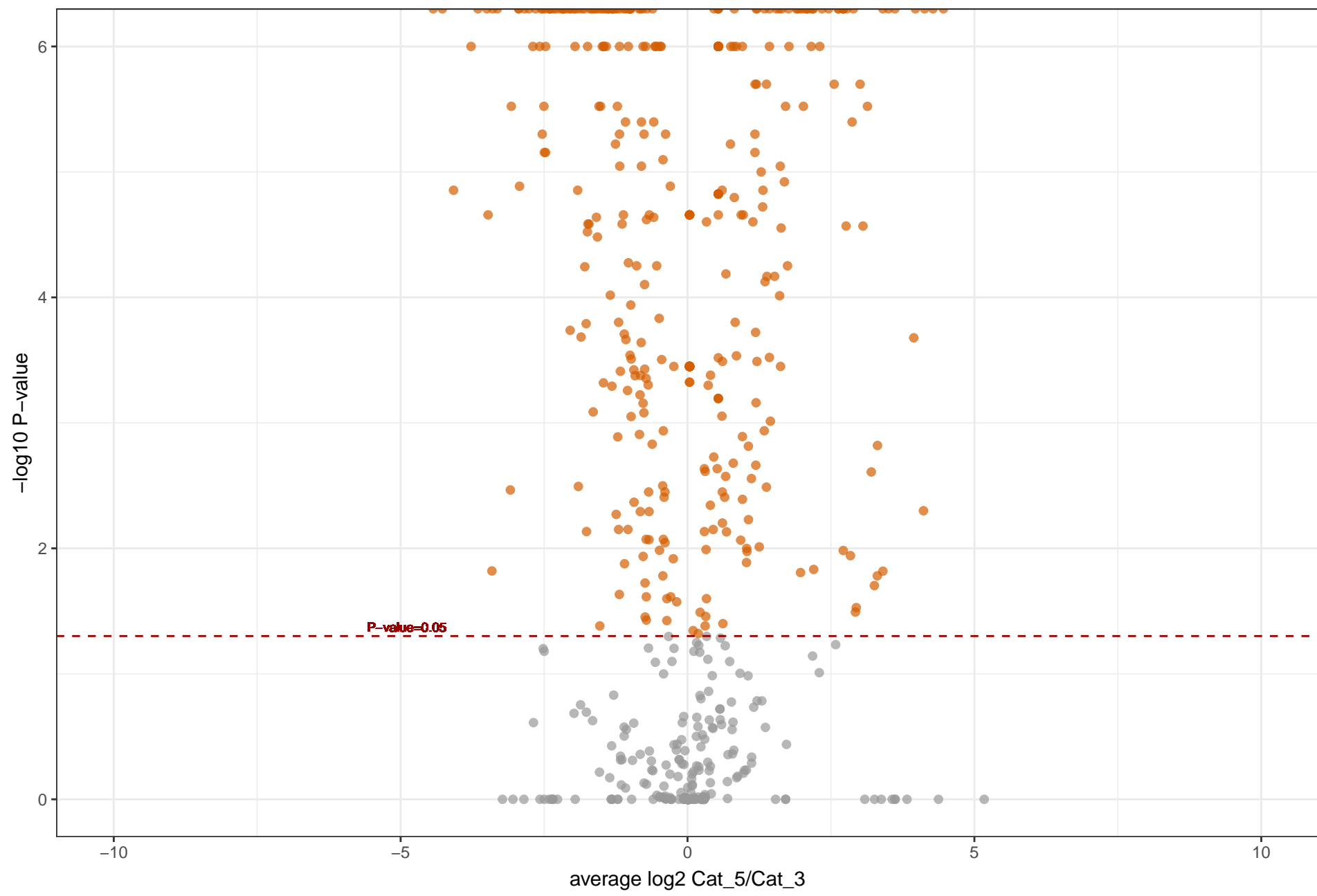

Supplement: Figure 6—source data 1. — Individual data from all figures involving small datasets displayed in individual tabs of this source file. This includes Figures 1B and 2A-F, Figure 3B, Figure 4, Figure 1—figure supplement 1 and Figure 2—figure supplement 1. [file elife-75798-fig6-data1.zip › Flores_Data/AF1_Cat_5.Cat_3-volcano_AFCat1.pdf]

Value-ordered fold change

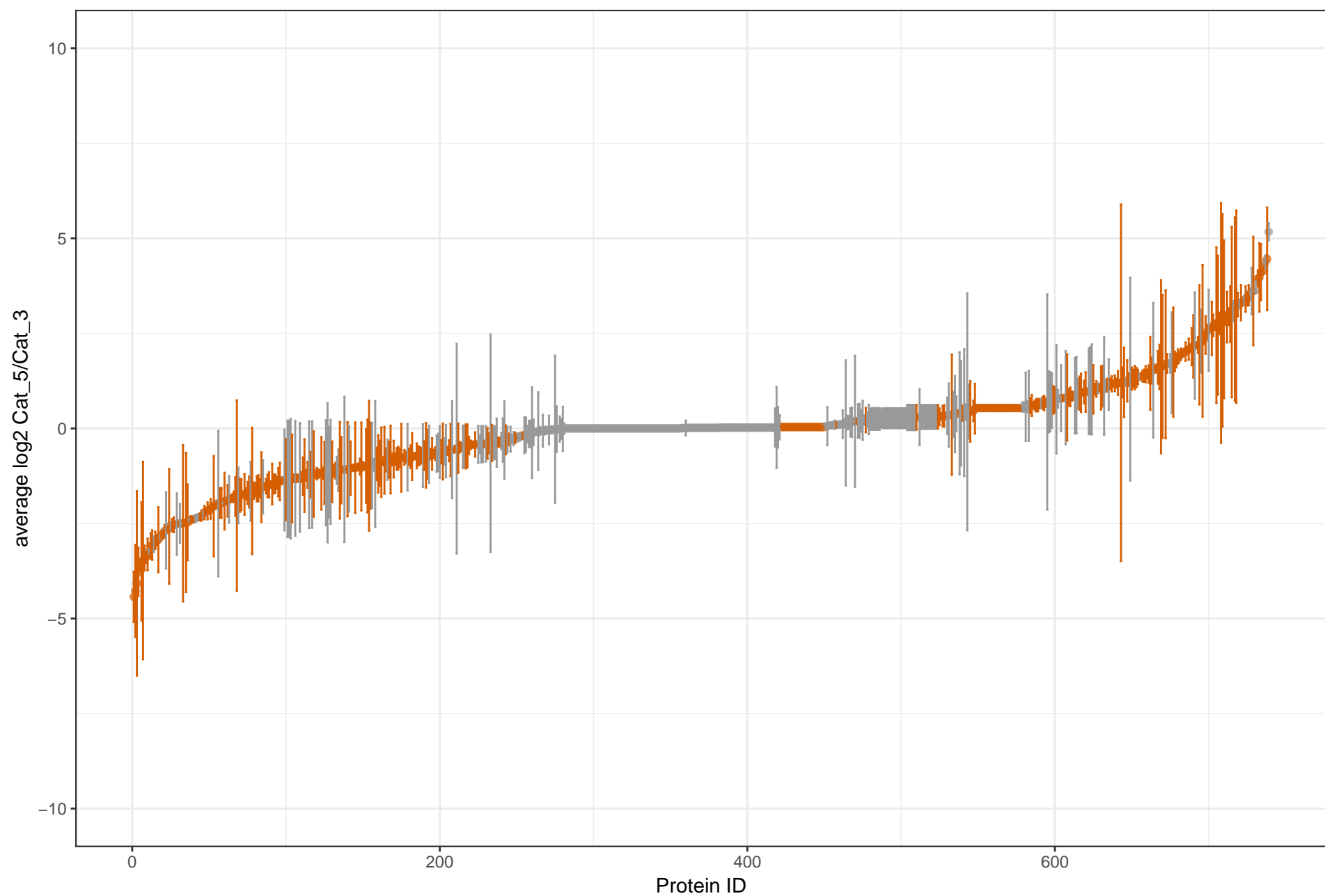

Supplement: Figure 6—source data 1. — Individual data from all figures involving small datasets displayed in individual tabs of this source file. This includes Figures 1B and 2A-F, Figure 3B, Figure 4, Figure 1—figure supplement 1 and Figure 2—figure supplement 1. [file elife-75798-fig6-data1.zip › Flores_Data/AF1_Cat_5.Cat_3-value-ordered-log-ratio_AFCat1.pdf]

**P-value vs Fold change**

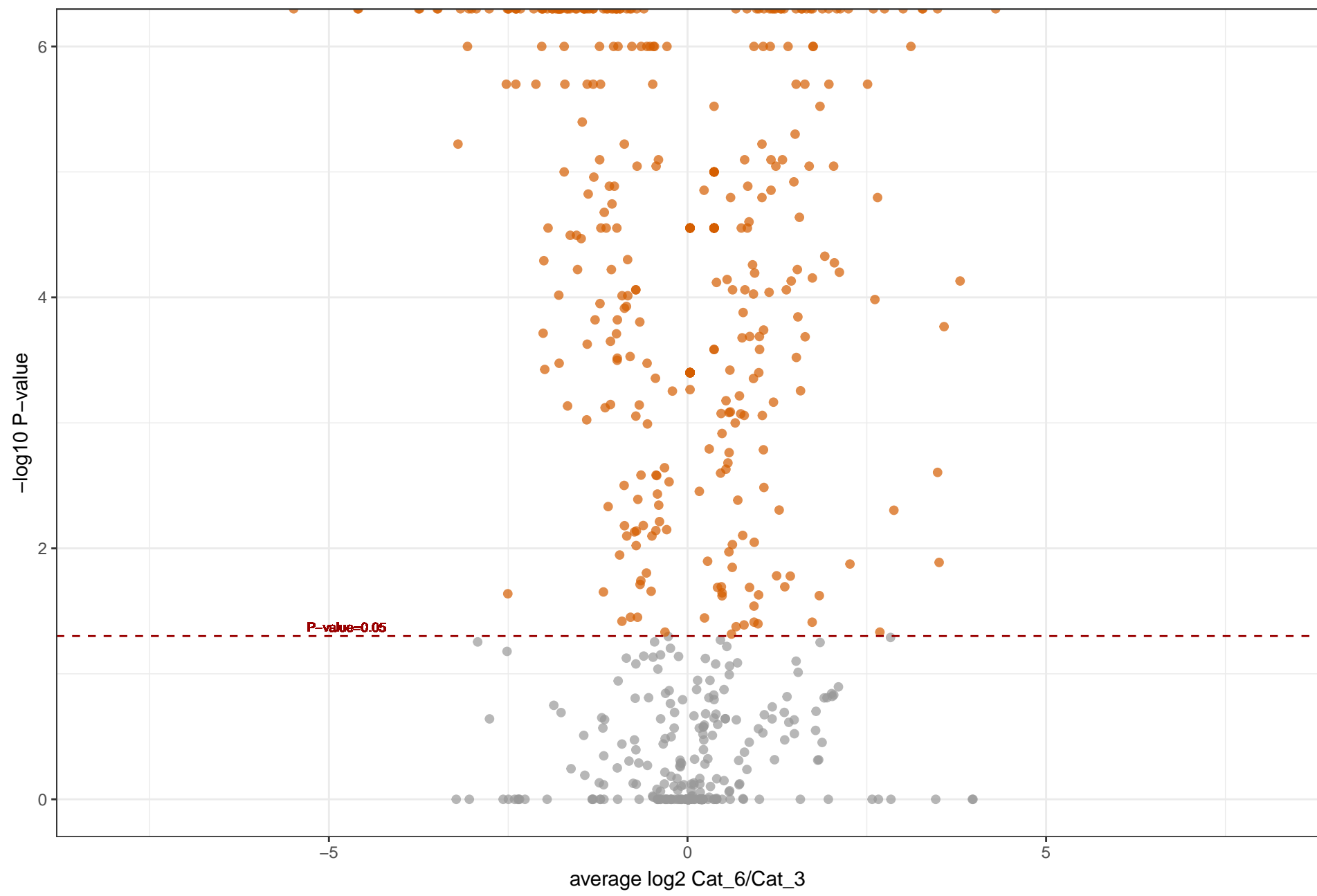

Supplement: Figure 6—source data 1. — Individual data from all figures involving small datasets displayed in individual tabs of this source file. This includes Figures 1B and 2A-F, Figure 3B, Figure 4, Figure 1—figure supplement 1 and Figure 2—figure supplement 1. [file elife-75798-fig6-data1.zip › Flores_Data/AF1_Cat_6.Cat_3-volcano_AFCat1.pdf]

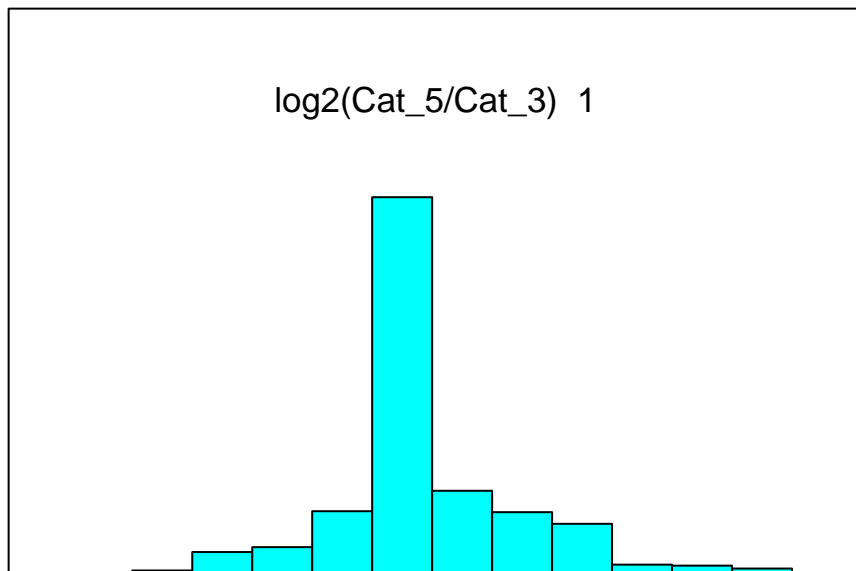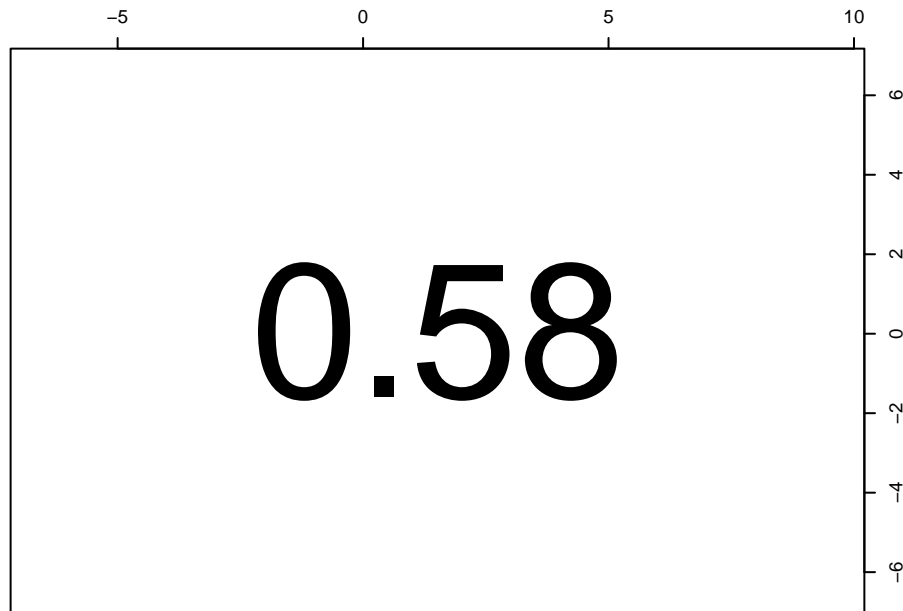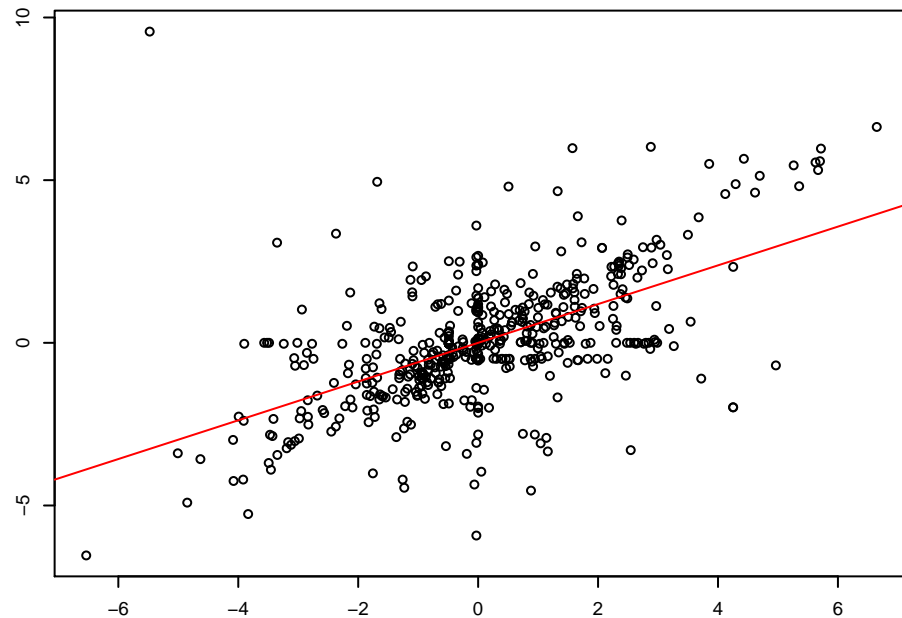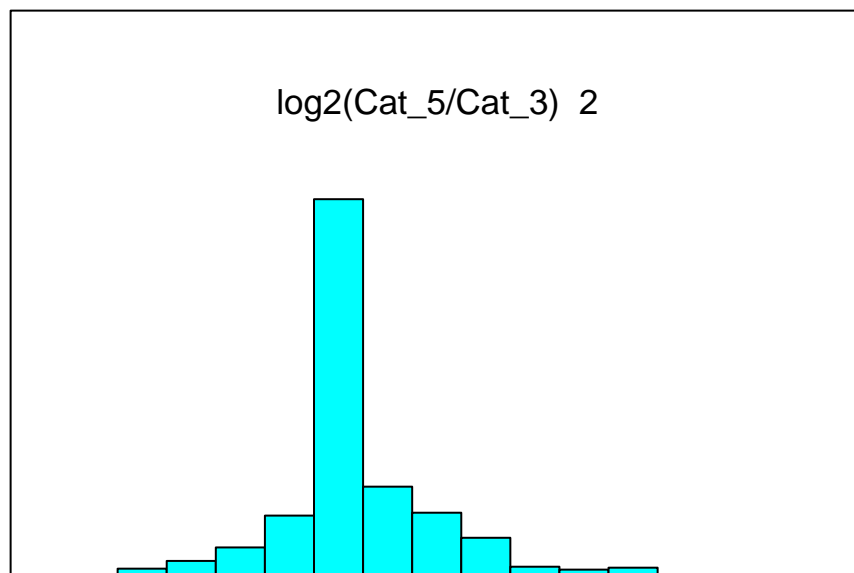

Supplement: Figure 6—source data 1. — Individual data from all figures involving small datasets displayed in individual tabs of this source file. This includes Figures 1B and 2A-F, Figure 3B, Figure 4, Figure 1—figure supplement 1 and Figure 2—figure supplement 1. [file elife-75798-fig6-data1.zip › Flores_Data/AF1_Cat_5.Cat_3-reproducibility_AFCat1.pdf]

MA plot

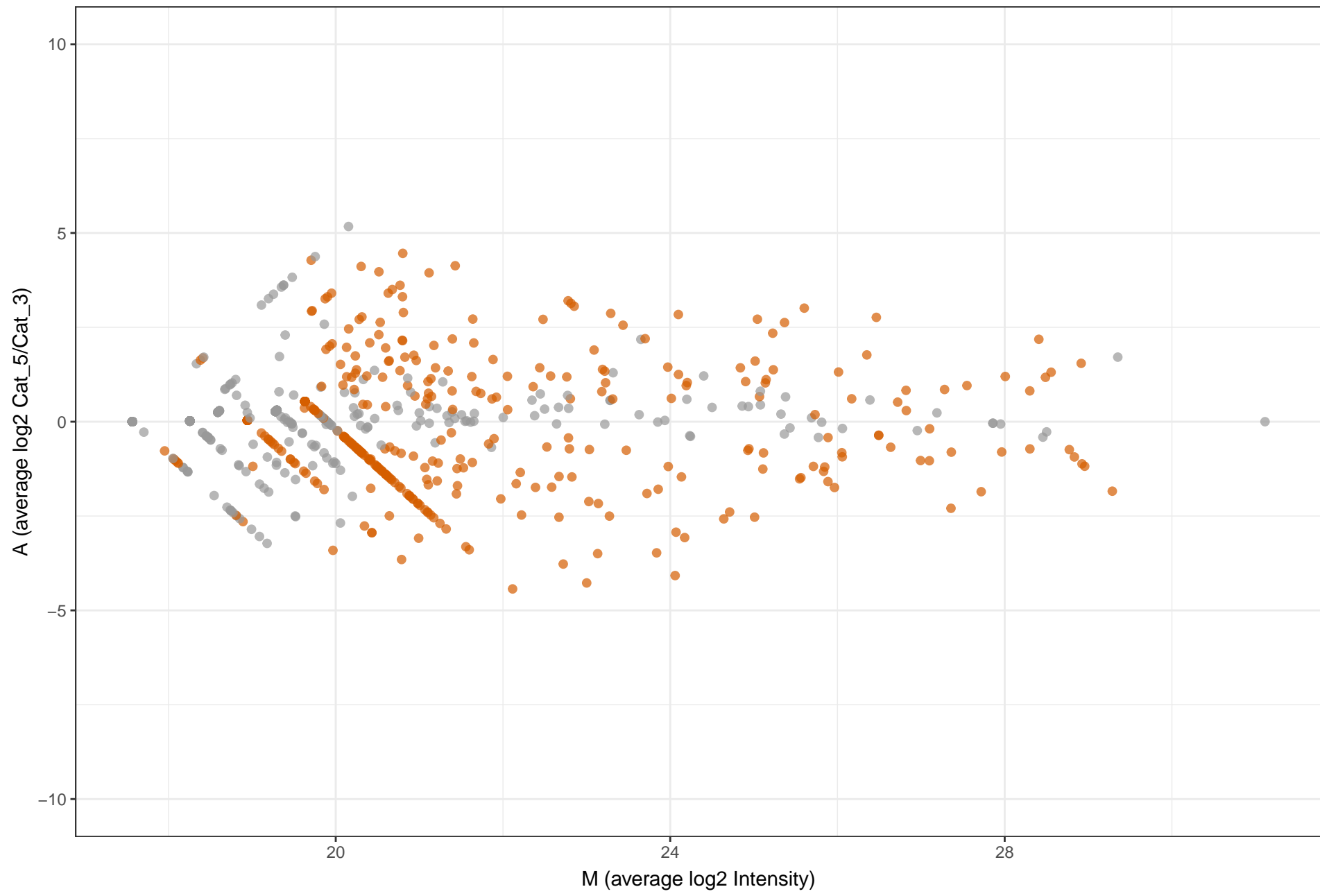

Supplement: Figure 6—source data 1. — Individual data from all figures involving small datasets displayed in individual tabs of this source file. This includes Figures 1B and 2A-F, Figure 3B, Figure 4, Figure 1—figure supplement 1 and Figure 2—figure supplement 1. [file elife-75798-fig6-data1.zip › Flores_Data/AF1_Cat_5.Cat_3-MA_AFCat1.pdf]

Value-ordered fold change

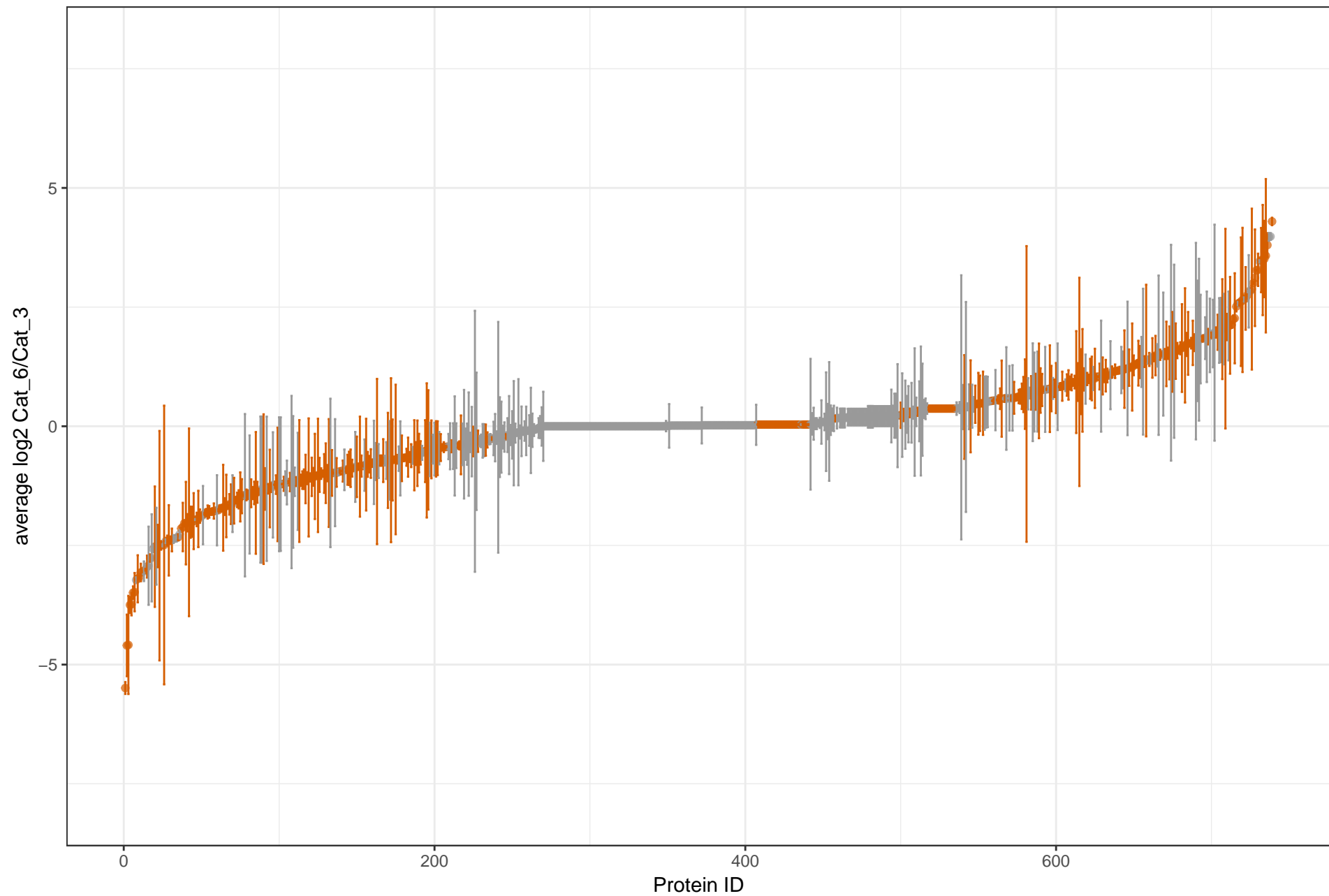

Supplement: Figure 6—source data 1. — Individual data from all figures involving small datasets displayed in individual tabs of this source file. This includes Figures 1B and 2A-F, Figure 3B, Figure 4, Figure 1—figure supplement 1 and Figure 2—figure supplement 1. [file elife-75798-fig6-data1.zip › Flores_Data/AF1_Cat_6.Cat_3-value-ordered-log-ratio_AFCat1.pdf]

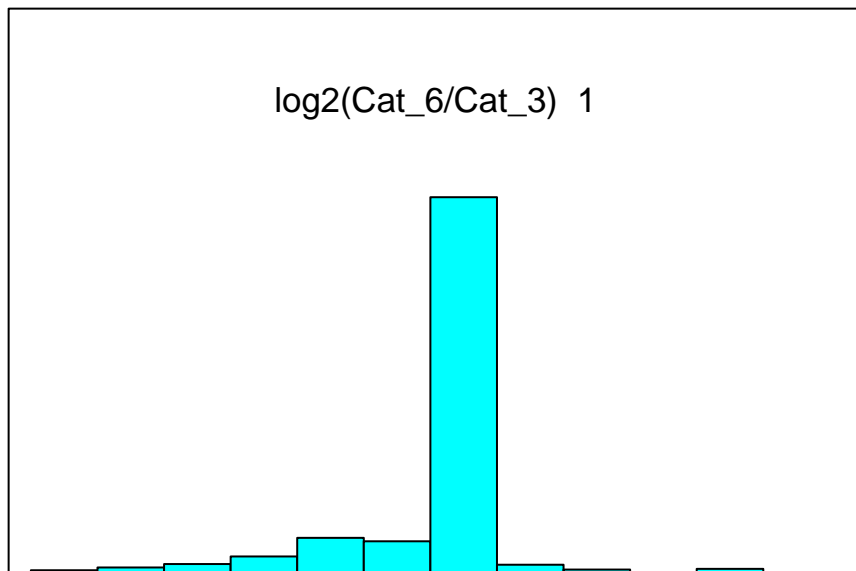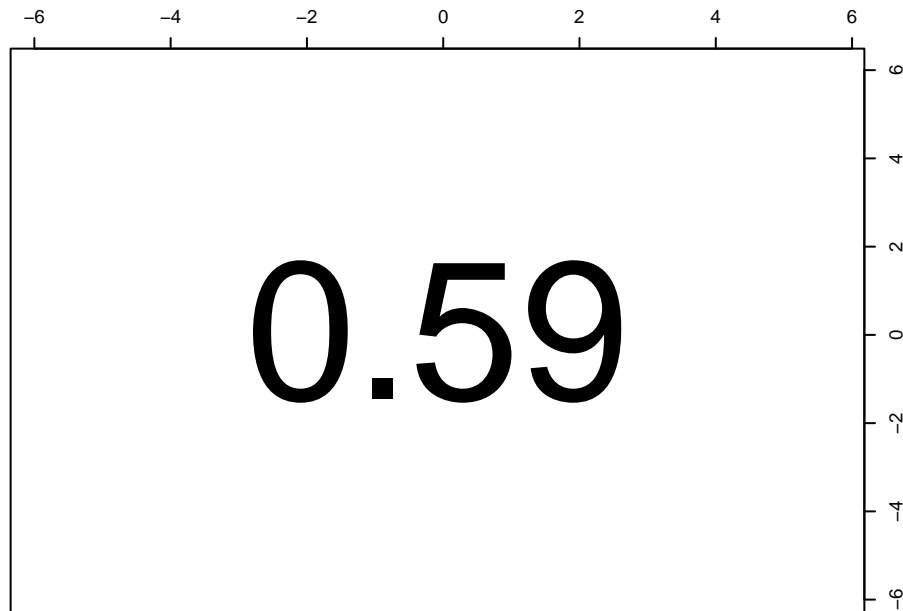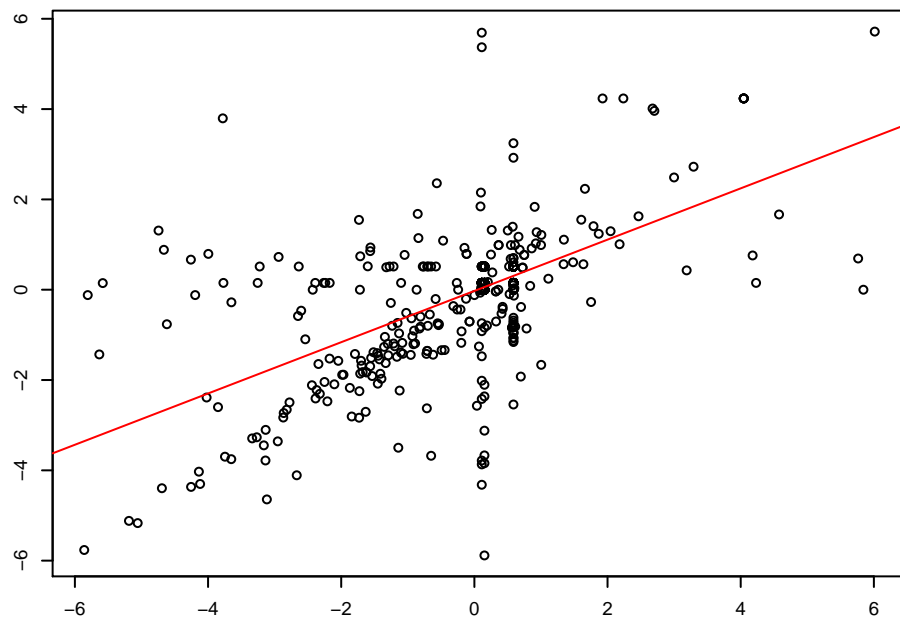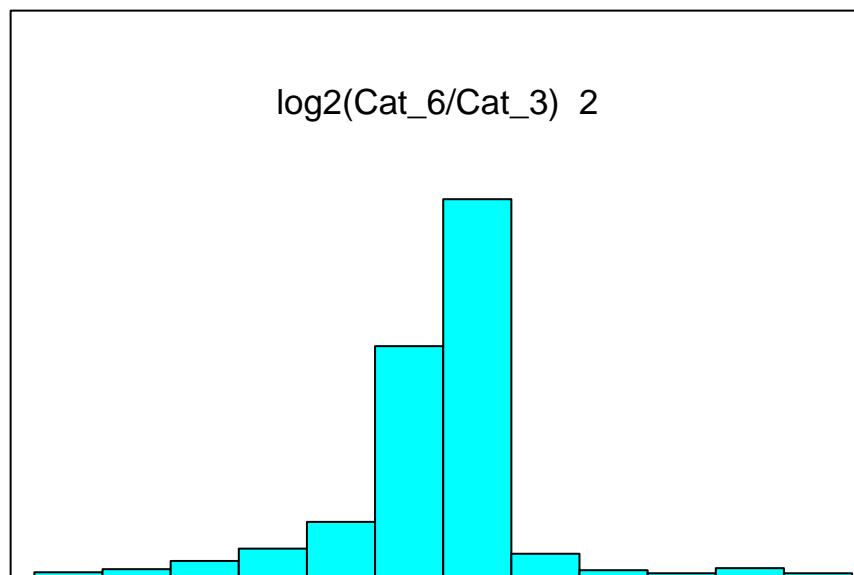

Supplement: Figure 6—source data 1. — Individual data from all figures involving small datasets displayed in individual tabs of this source file. This includes Figures 1B and 2A-F, Figure 3B, Figure 4, Figure 1—figure supplement 1 and Figure 2—figure supplement 1. [file elife-75798-fig6-data1.zip › Flores_Data/AF1_Cat_6.Cat_3-reproducibility_AFCat1.pdf]

MA plot

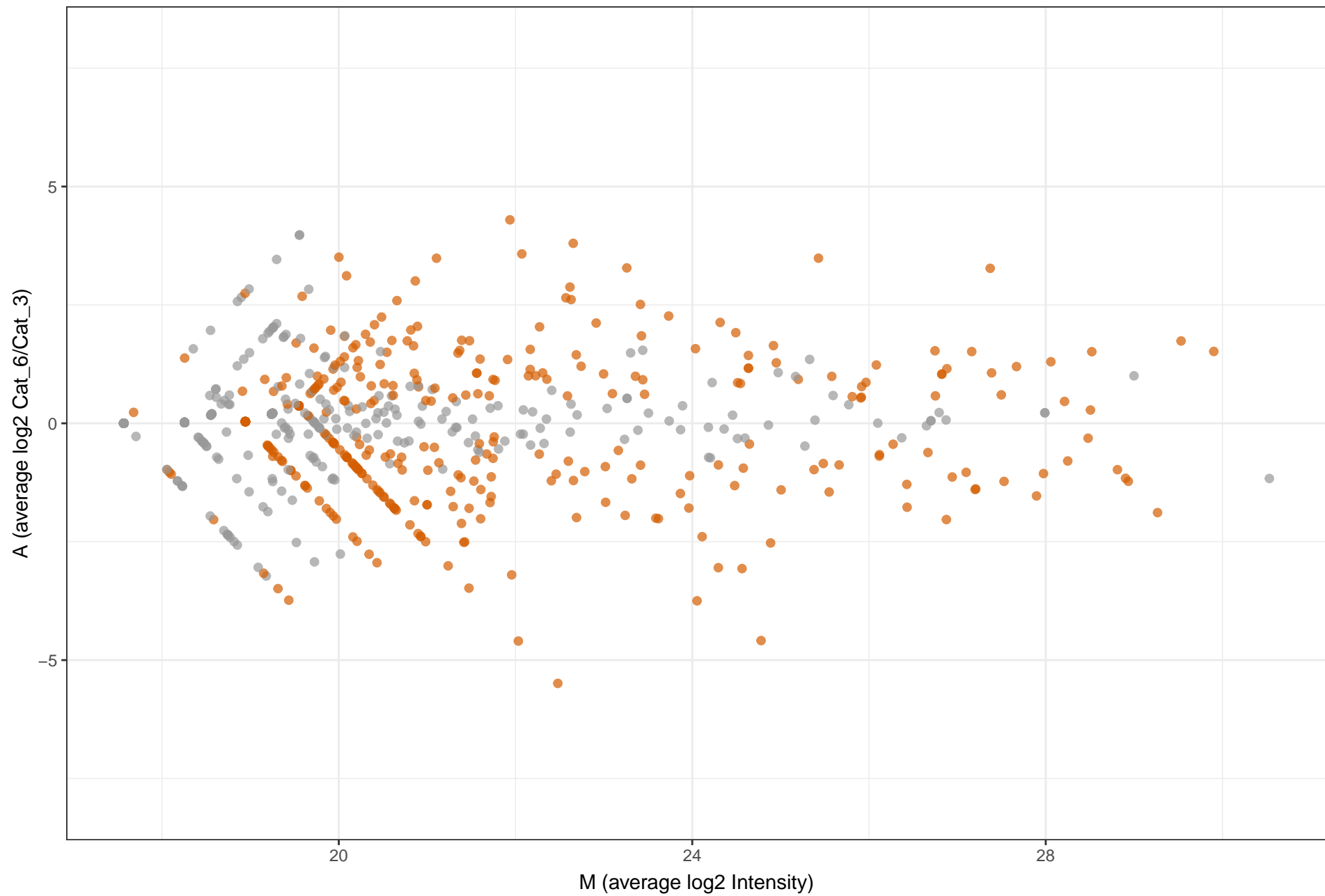

Supplement: Figure 6—source data 1. — Individual data from all figures involving small datasets displayed in individual tabs of this source file. This includes Figures 1B and 2A-F, Figure 3B, Figure 4, Figure 1—figure supplement 1 and Figure 2—figure supplement 1. [file elife-75798-fig6-data1.zip › Flores_Data/AF1_Cat_6.Cat_3-MA_AFCat1.pdf]

P-value vs Fold change

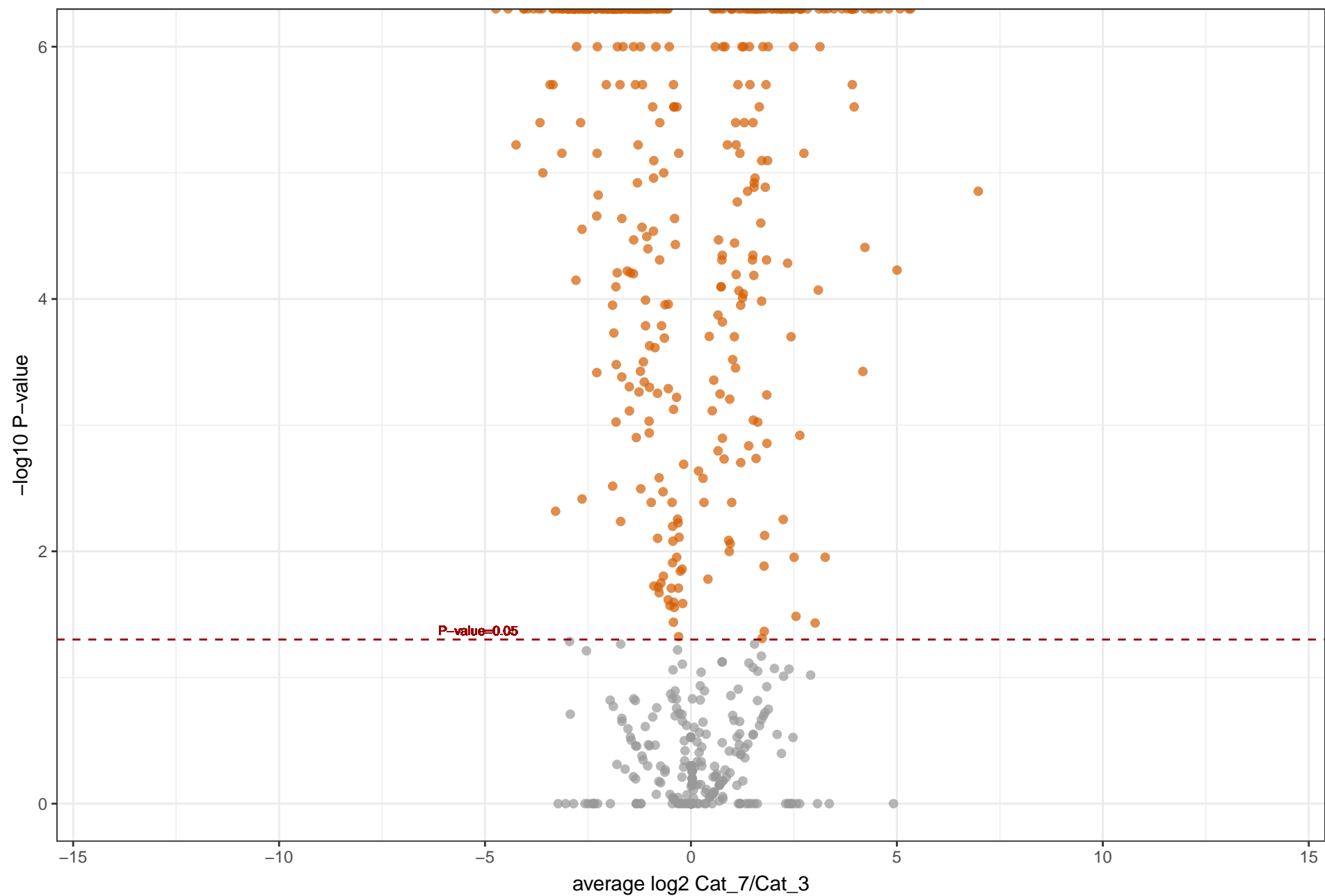

Supplement: Figure 6—source data 1. — Individual data from all figures involving small datasets displayed in individual tabs of this source file. This includes Figures 1B and 2A-F, Figure 3B, Figure 4, Figure 1—figure supplement 1 and Figure 2—figure supplement 1. [file elife-75798-fig6-data1.zip › Flores_Data/AF1_Cat_7.Cat_3-volcano_AFCat1.pdf]

Value-ordered fold change

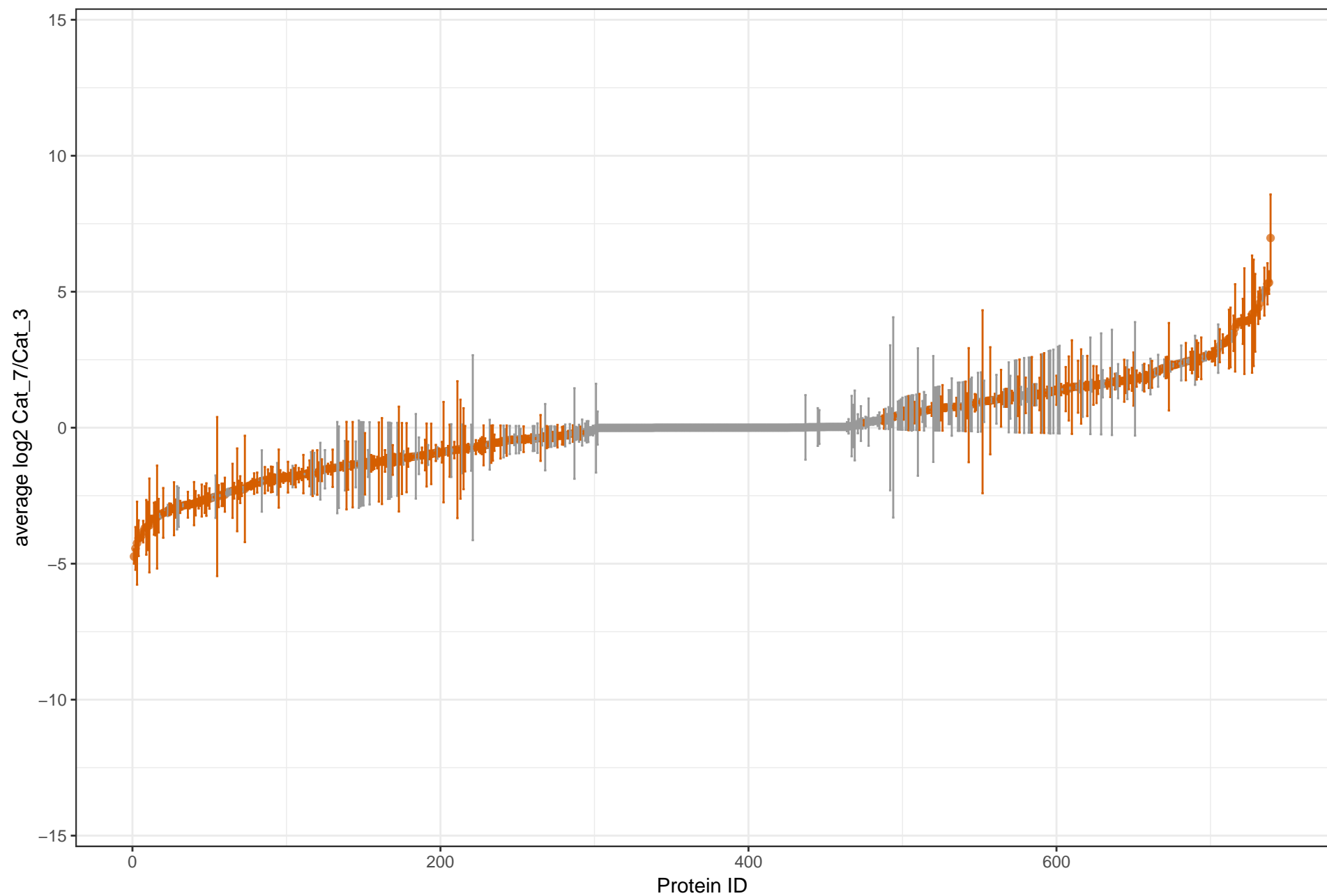

Supplement: Figure 6—source data 1. — Individual data from all figures involving small datasets displayed in individual tabs of this source file. This includes Figures 1B and 2A-F, Figure 3B, Figure 4, Figure 1—figure supplement 1 and Figure 2—figure supplement 1. [file elife-75798-fig6-data1.zip › Flores_Data/AF1_Cat_7.Cat_3-value-ordered-log-ratio_AFCat1.pdf]

MA plot

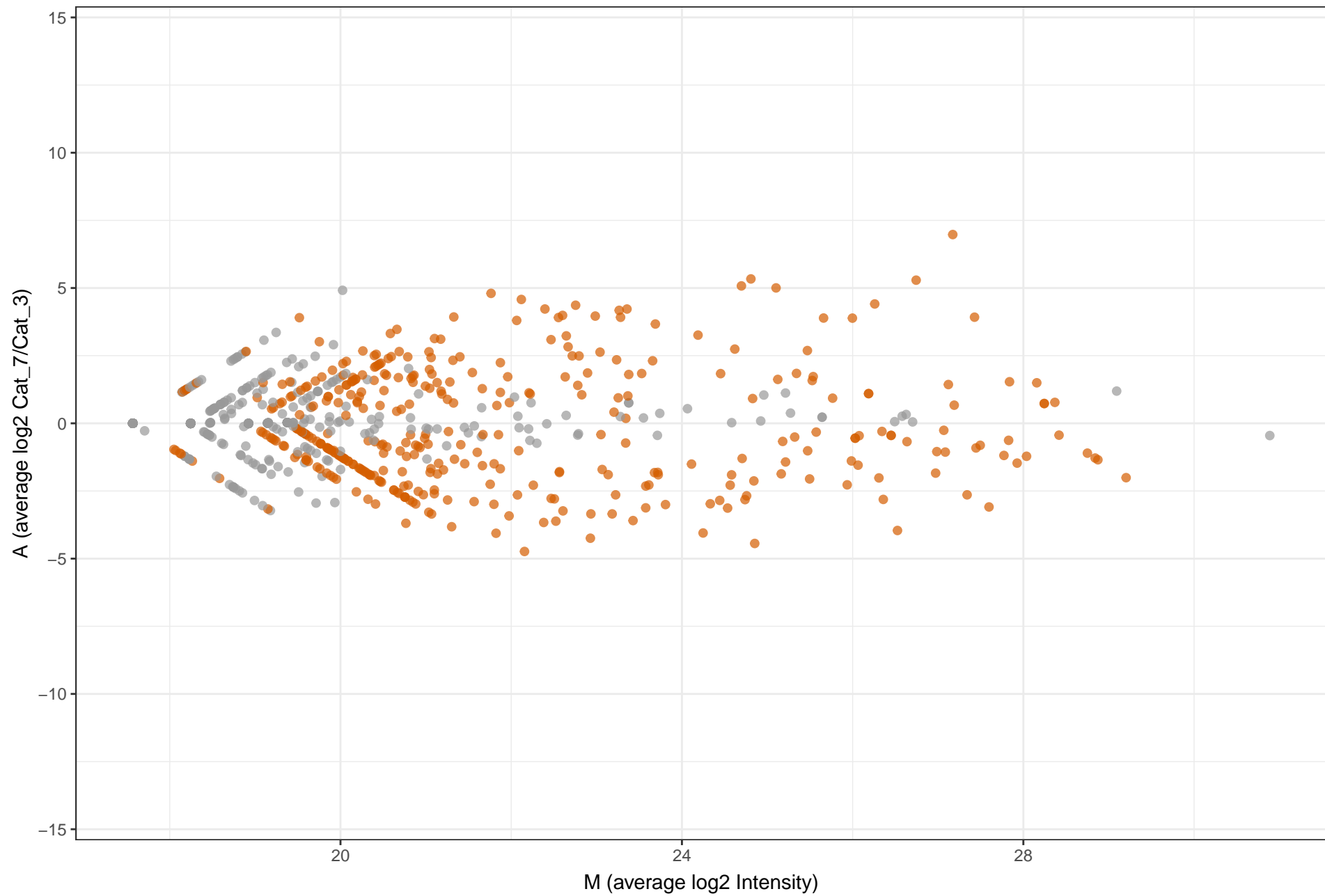

Supplement: Figure 6—source data 1. — Individual data from all figures involving small datasets displayed in individual tabs of this source file. This includes Figures 1B and 2A-F, Figure 3B, Figure 4, Figure 1—figure supplement 1 and Figure 2—figure supplement 1. [file elife-75798-fig6-data1.zip › Flores_Data/AF1_Cat_7.Cat_3-MA_AFCat1.pdf]

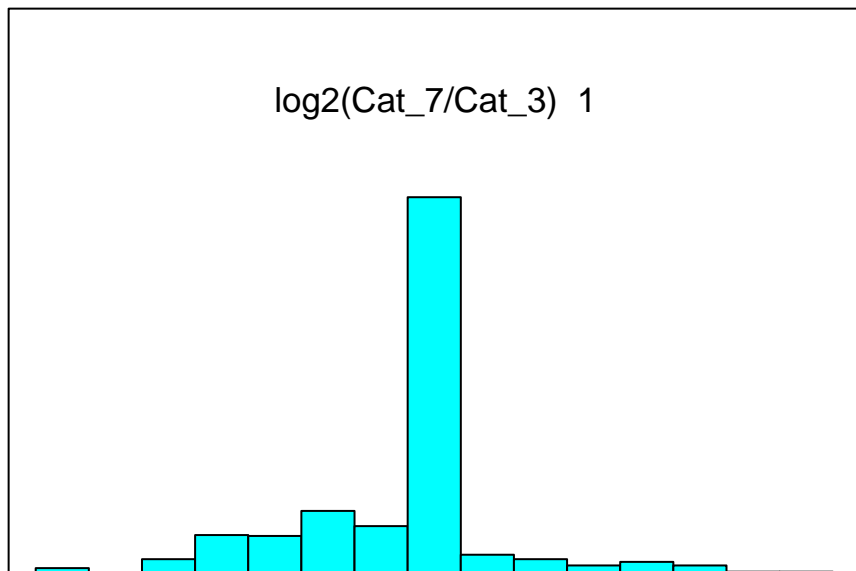

-5 0 5 10

5  
0  
-5

0.73

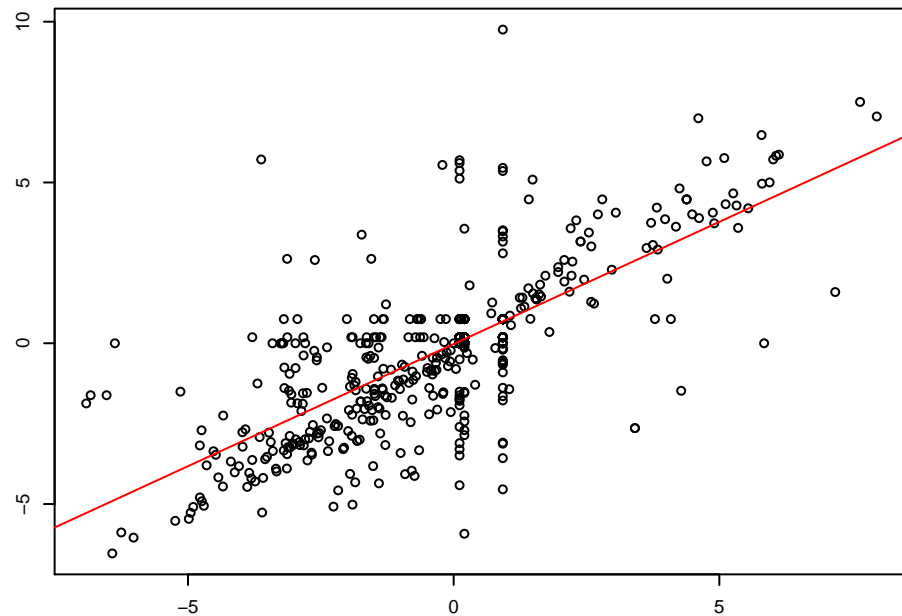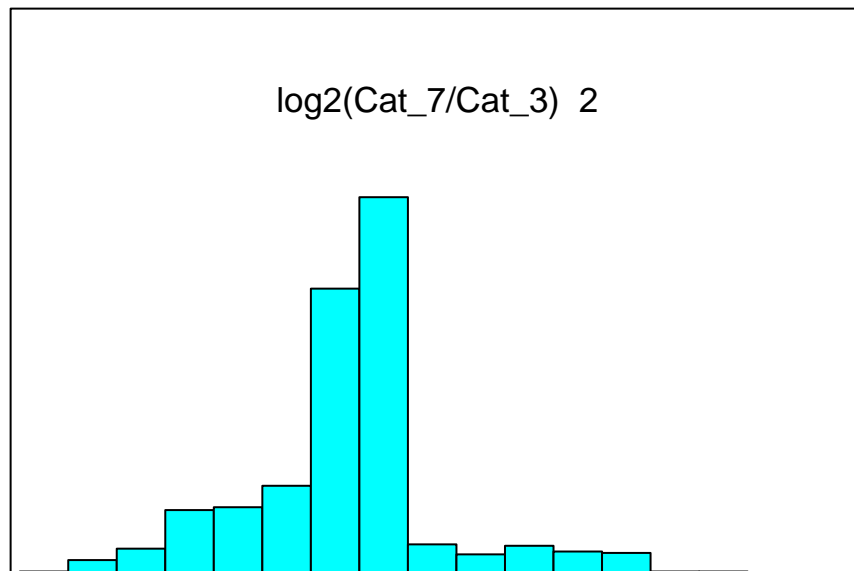

Supplement: Figure 6—source data 1. — Individual data from all figures involving small datasets displayed in individual tabs of this source file. This includes Figures 1B and 2A-F, Figure 3B, Figure 4, Figure 1—figure supplement 1 and Figure 2—figure supplement 1. [file elife-75798-fig6-data1.zip › Flores_Data/AF1_Cat_7.Cat_3-reproducibility_AFCat1.pdf]

**P-value vs Fold change**

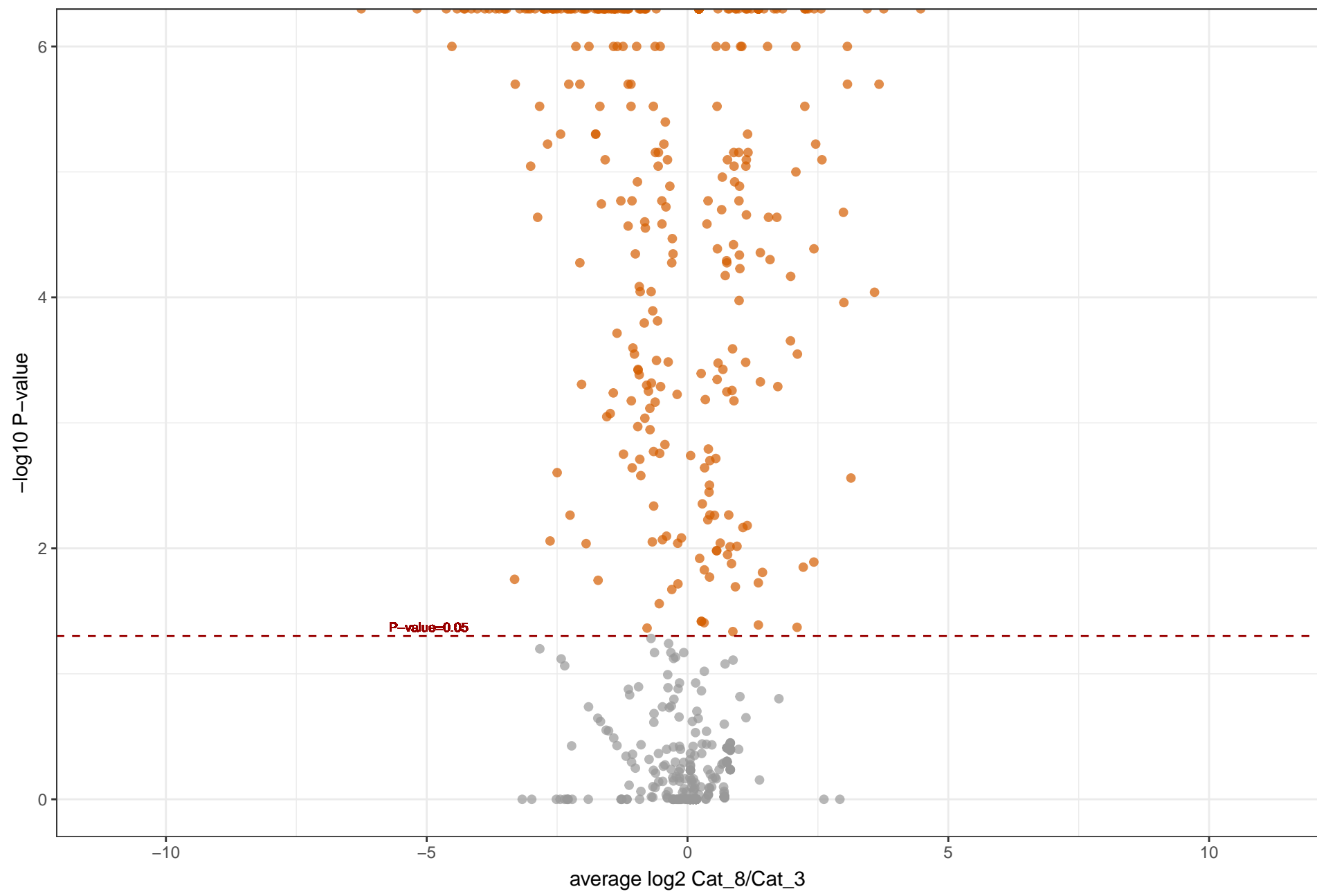

Supplement: Figure 6—source data 1. — Individual data from all figures involving small datasets displayed in individual tabs of this source file. This includes Figures 1B and 2A-F, Figure 3B, Figure 4, Figure 1—figure supplement 1 and Figure 2—figure supplement 1. [file elife-75798-fig6-data1.zip › Flores_Data/AF1_Cat_8.Cat_3-volcano_AFCat1.pdf]

Value-ordered fold change

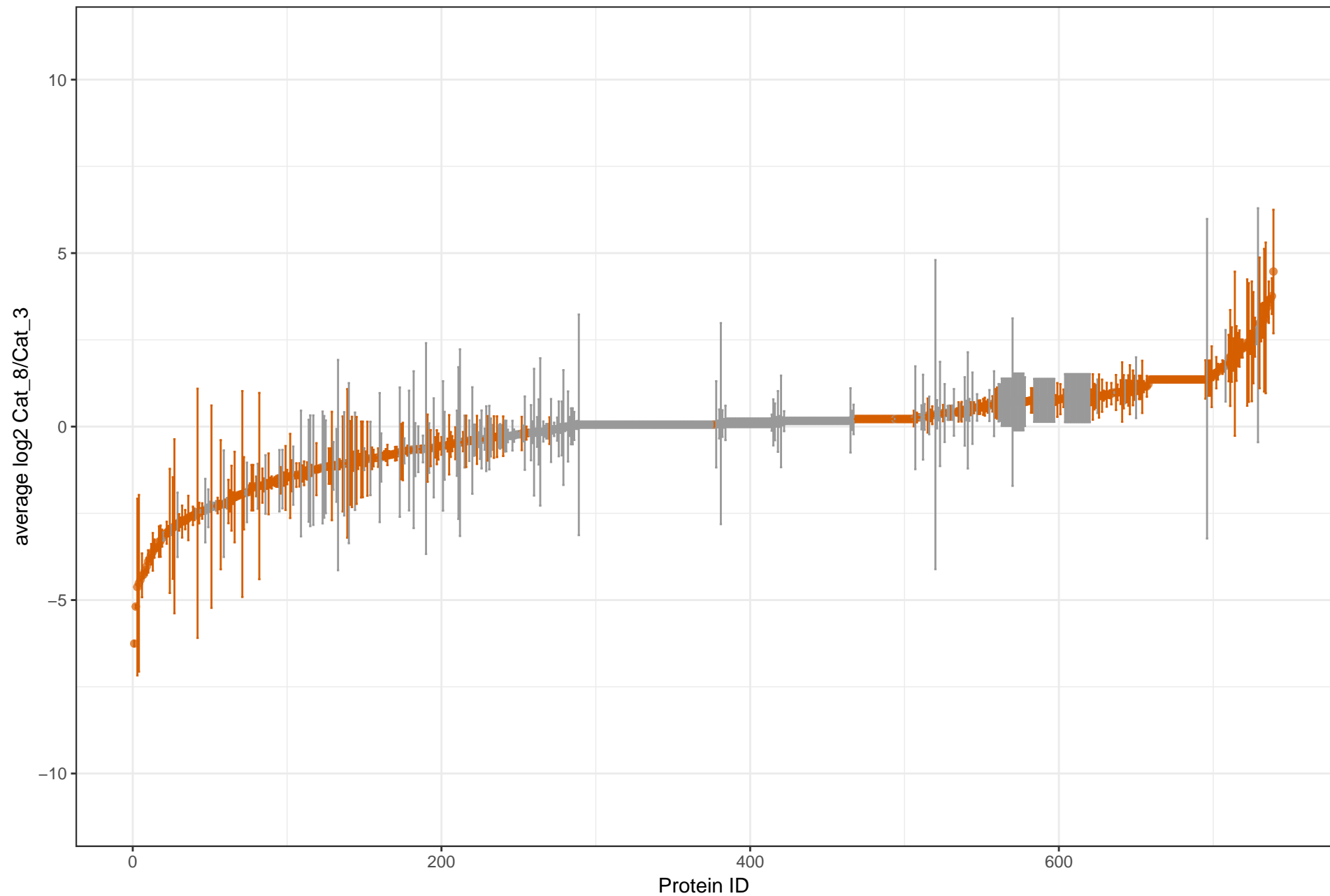

Supplement: Figure 6—source data 1. — Individual data from all figures involving small datasets displayed in individual tabs of this source file. This includes Figures 1B and 2A-F, Figure 3B, Figure 4, Figure 1—figure supplement 1 and Figure 2—figure supplement 1. [file elife-75798-fig6-data1.zip › Flores_Data/AF1_Cat_8.Cat_3-value-ordered-log-ratio_AFCat1.pdf]

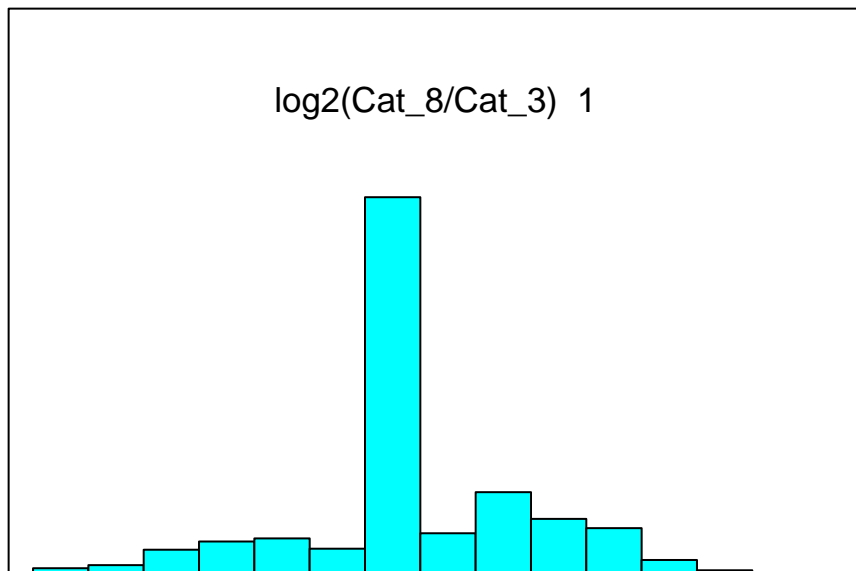

-5 0 5

6  
4  
2  
0  
-2  
-4  
-6

0.78

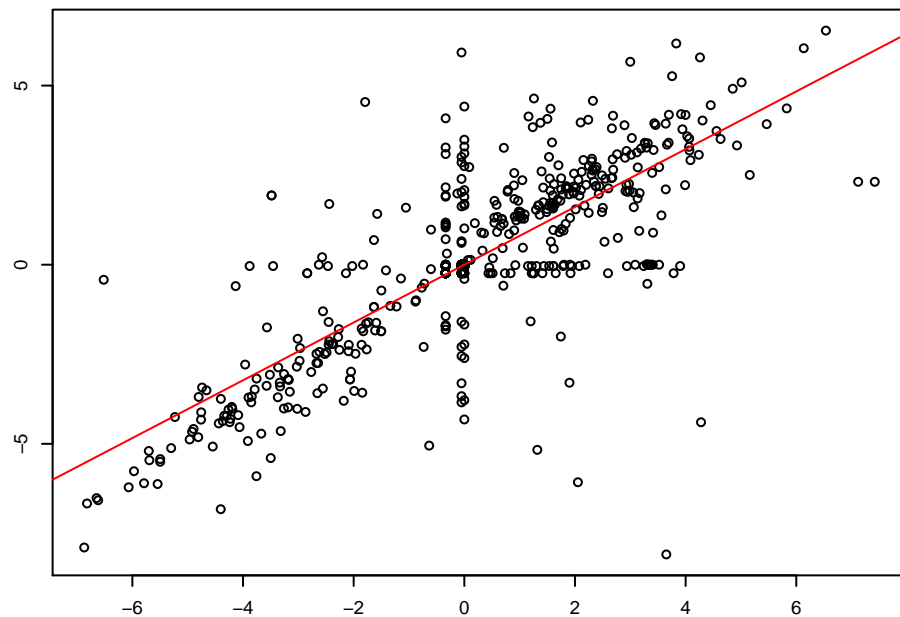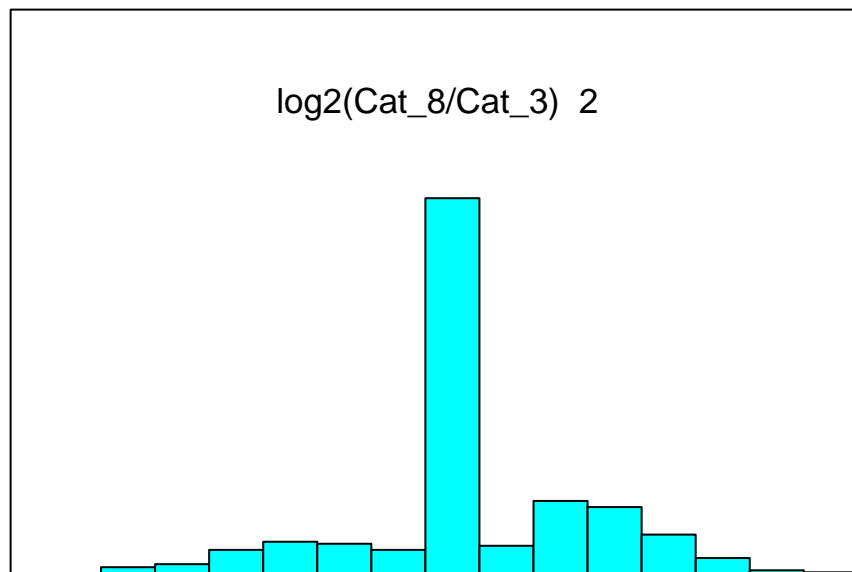

Supplement: Figure 6—source data 1. — Individual data from all figures involving small datasets displayed in individual tabs of this source file. This includes Figures 1B and 2A-F, Figure 3B, Figure 4, Figure 1—figure supplement 1 and Figure 2—figure supplement 1. [file elife-75798-fig6-data1.zip › Flores_Data/AF1_Cat_8.Cat_3-reproducibility_AFCat1.pdf]

MA plot

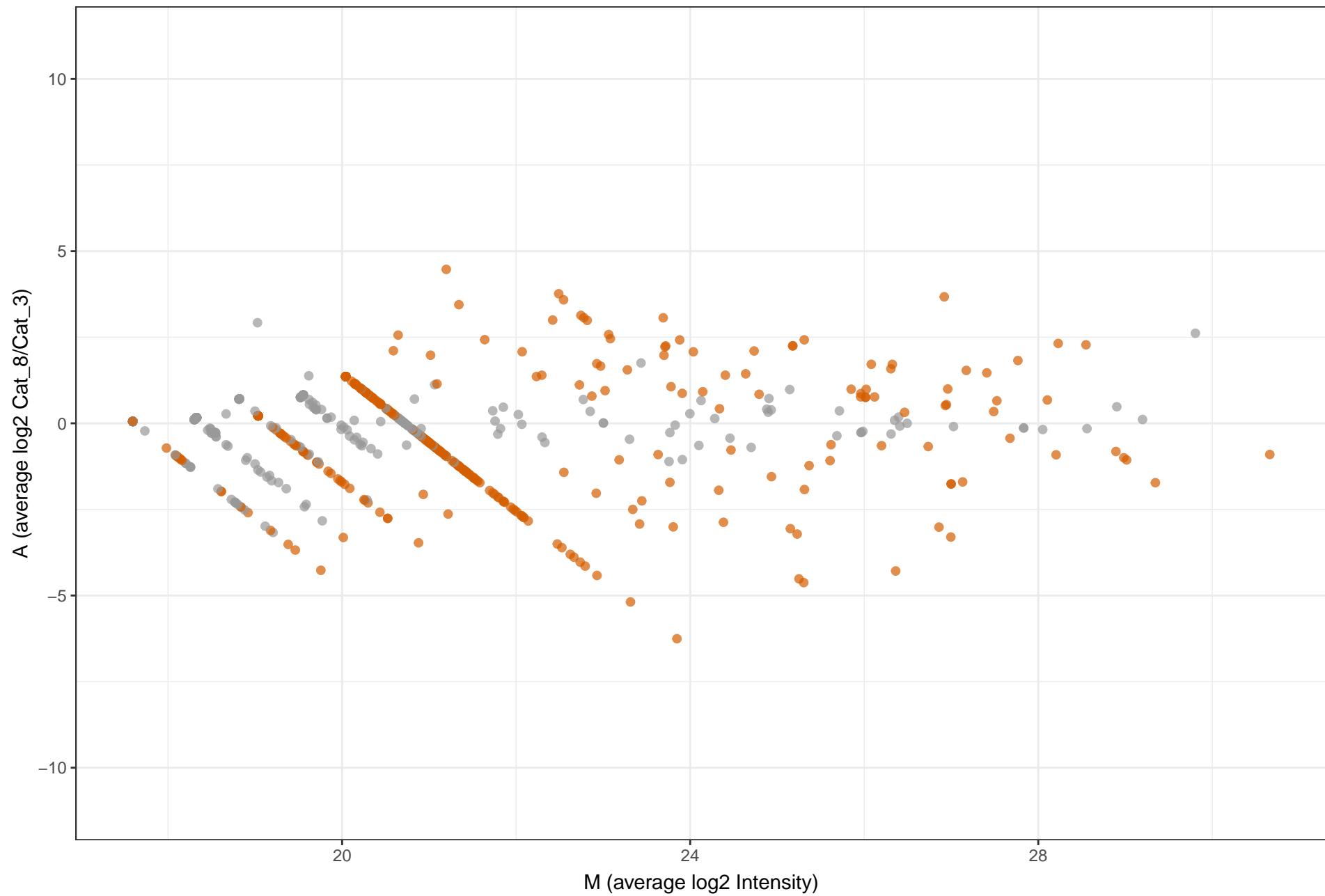

Supplement: Figure 6—source data 1. — Individual data from all figures involving small datasets displayed in individual tabs of this source file. This includes Figures 1B and 2A-F, Figure 3B, Figure 4, Figure 1—figure supplement 1 and Figure 2—figure supplement 1. [file elife-75798-fig6-data1.zip › Flores_Data/AF1_Cat_8.Cat_3-MA_AFCat1.pdf]

**P-value vs Fold change**

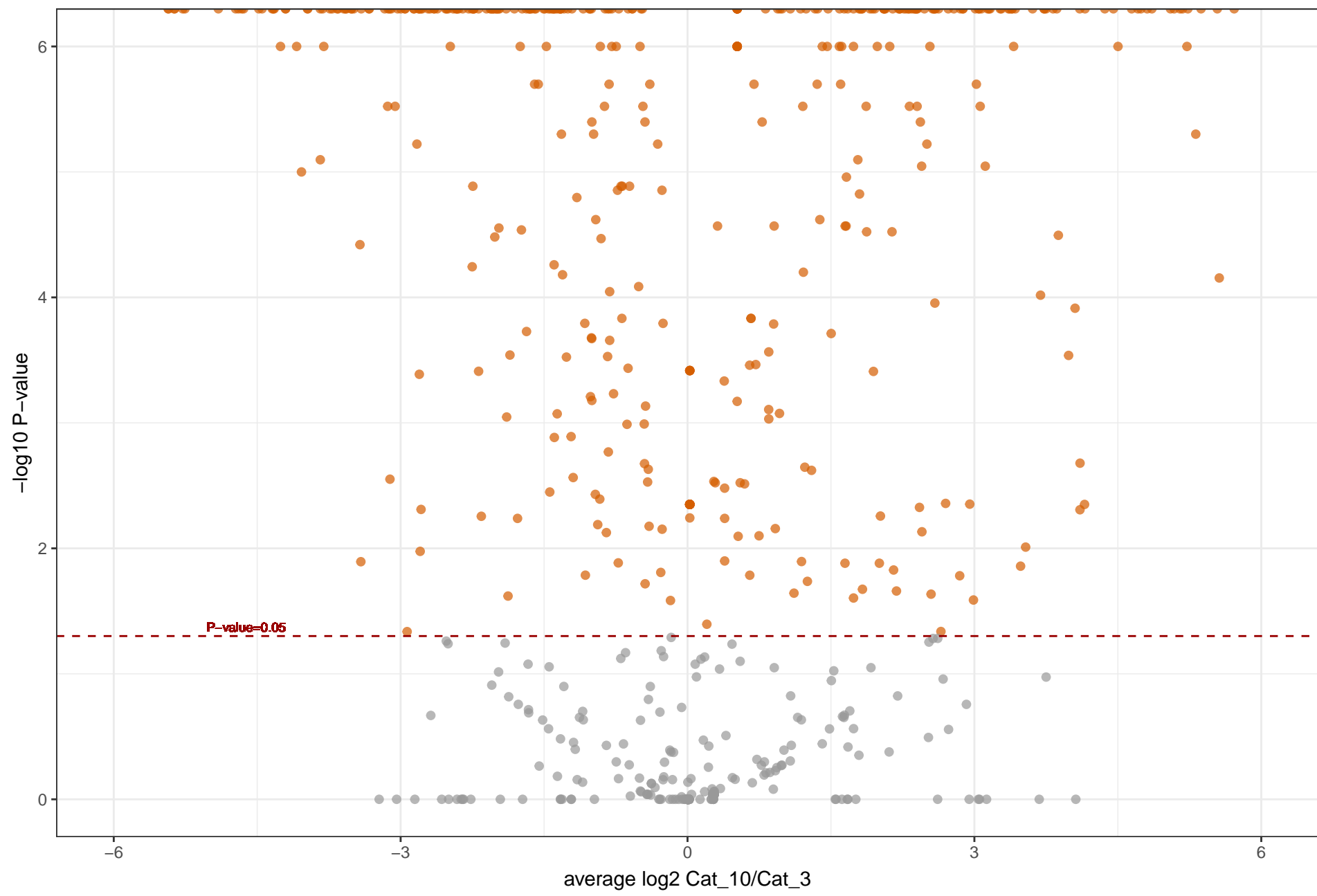

Supplement: Figure 6—source data 1. — Individual data from all figures involving small datasets displayed in individual tabs of this source file. This includes Figures 1B and 2A-F, Figure 3B, Figure 4, Figure 1—figure supplement 1 and Figure 2—figure supplement 1. [file elife-75798-fig6-data1.zip › Flores_Data/AF1_Cat_10.Cat_3-volcano_AFCat1.pdf]

Value-ordered fold change

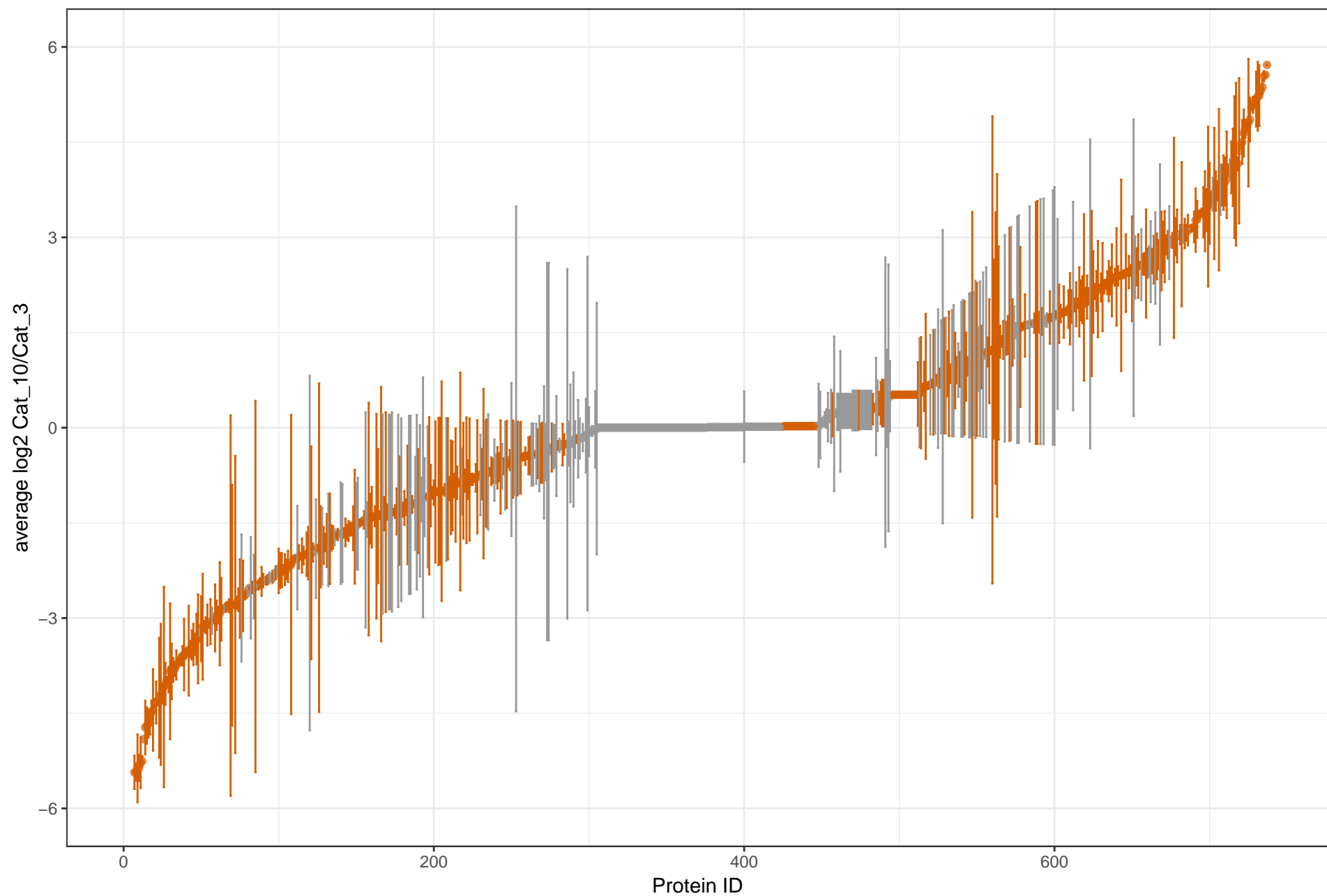

Supplement: Figure 6—source data 1. — Individual data from all figures involving small datasets displayed in individual tabs of this source file. This includes Figures 1B and 2A-F, Figure 3B, Figure 4, Figure 1—figure supplement 1 and Figure 2—figure supplement 1. [file elife-75798-fig6-data1.zip › Flores_Data/AF1_Cat_10.Cat_3-value-ordered-log-ratio_AFCat1.pdf]

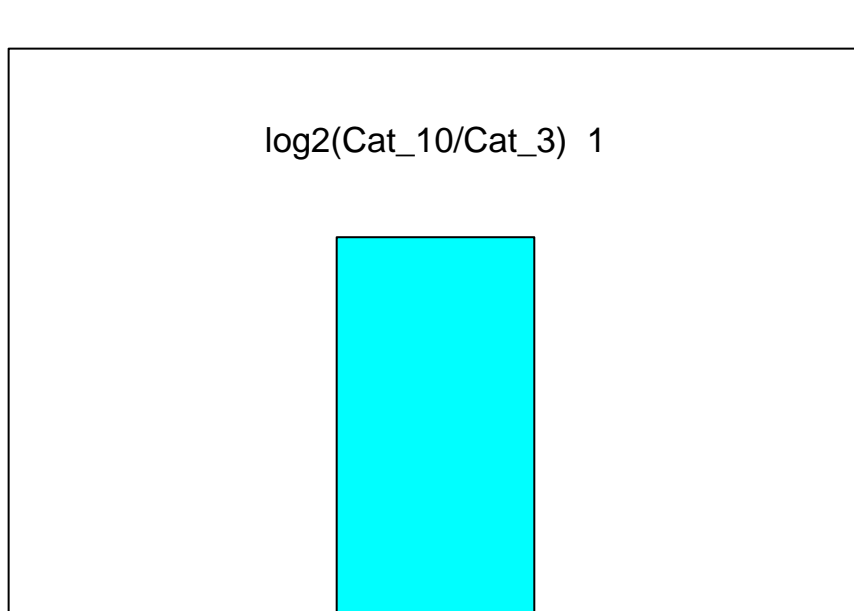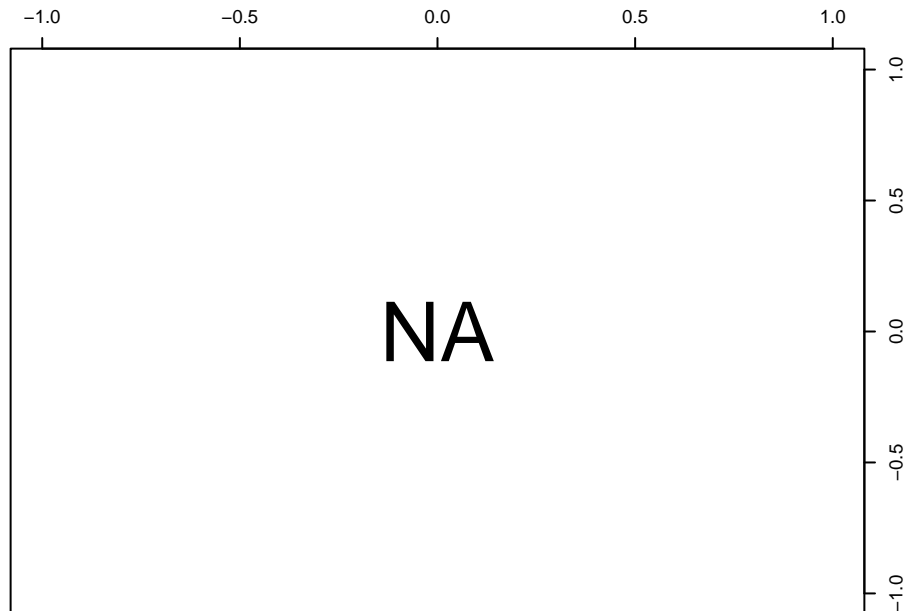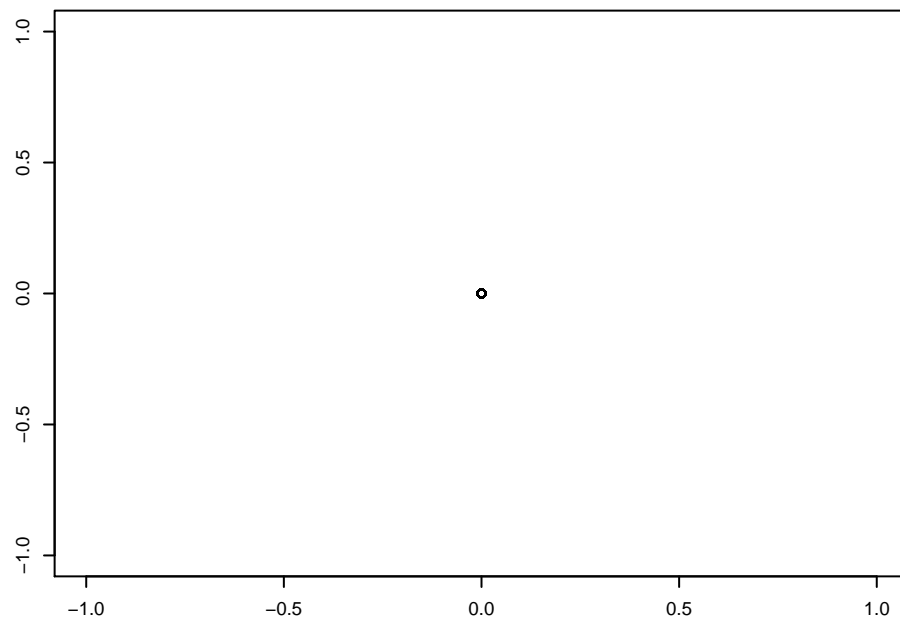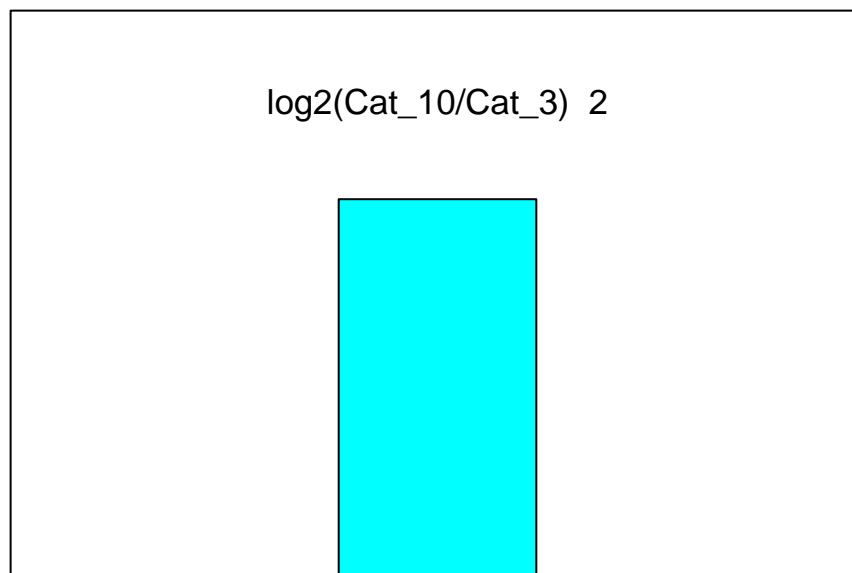

Supplement: Figure 6—source data 1. — Individual data from all figures involving small datasets displayed in individual tabs of this source file. This includes Figures 1B and 2A-F, Figure 3B, Figure 4, Figure 1—figure supplement 1 and Figure 2—figure supplement 1. [file elife-75798-fig6-data1.zip › Flores_Data/AF1_Cat_10.Cat_3-reproducibility_AFCat1.pdf]

MA plot

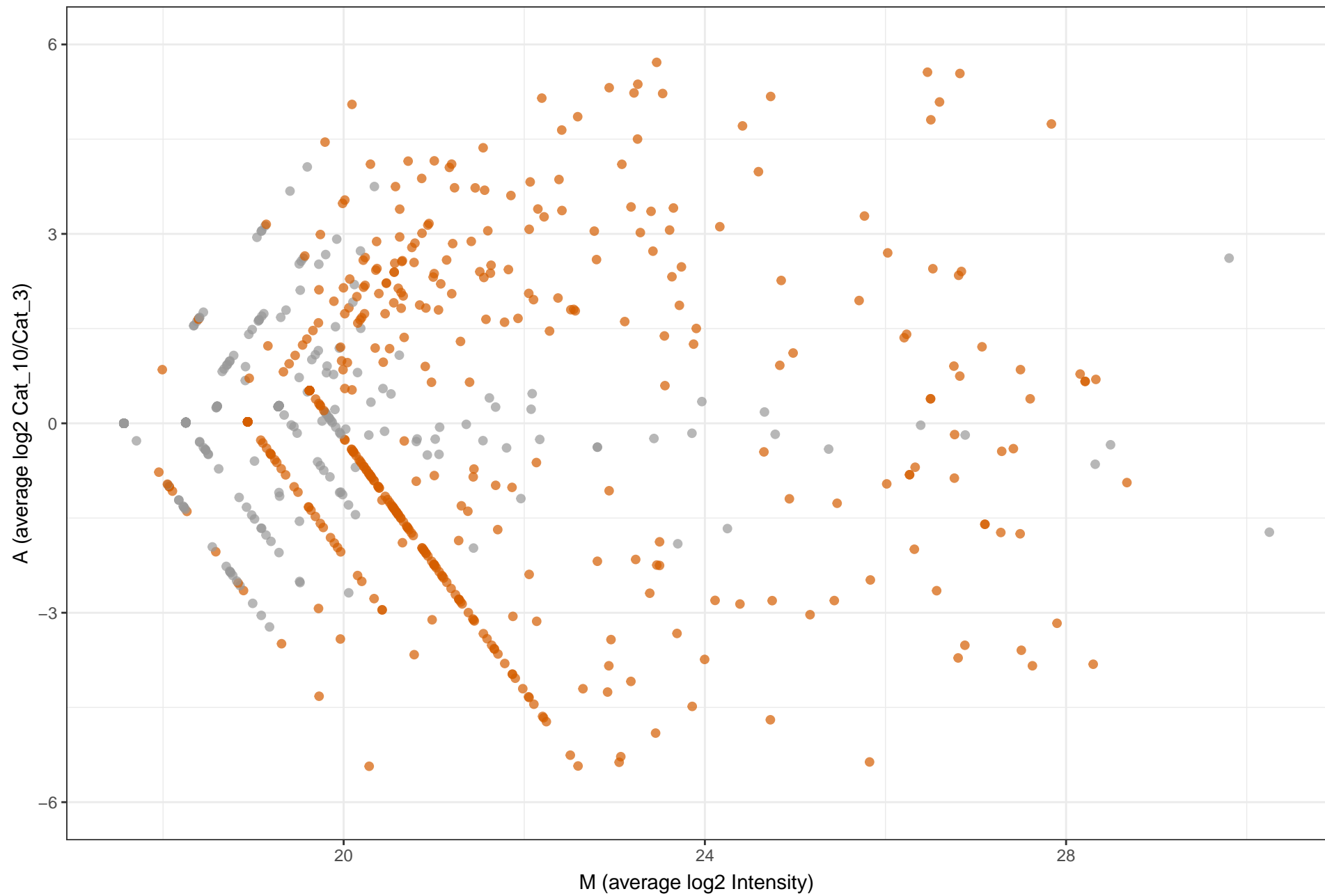

Supplement: Figure 6—source data 1. — Individual data from all figures involving small datasets displayed in individual tabs of this source file. This includes Figures 1B and 2A-F, Figure 3B, Figure 4, Figure 1—figure supplement 1 and Figure 2—figure supplement 1. [file elife-75798-fig6-data1.zip › Flores_Data/AF1_Cat_10.Cat_3-MA_AFCat1.pdf]

**P-value vs Fold change**

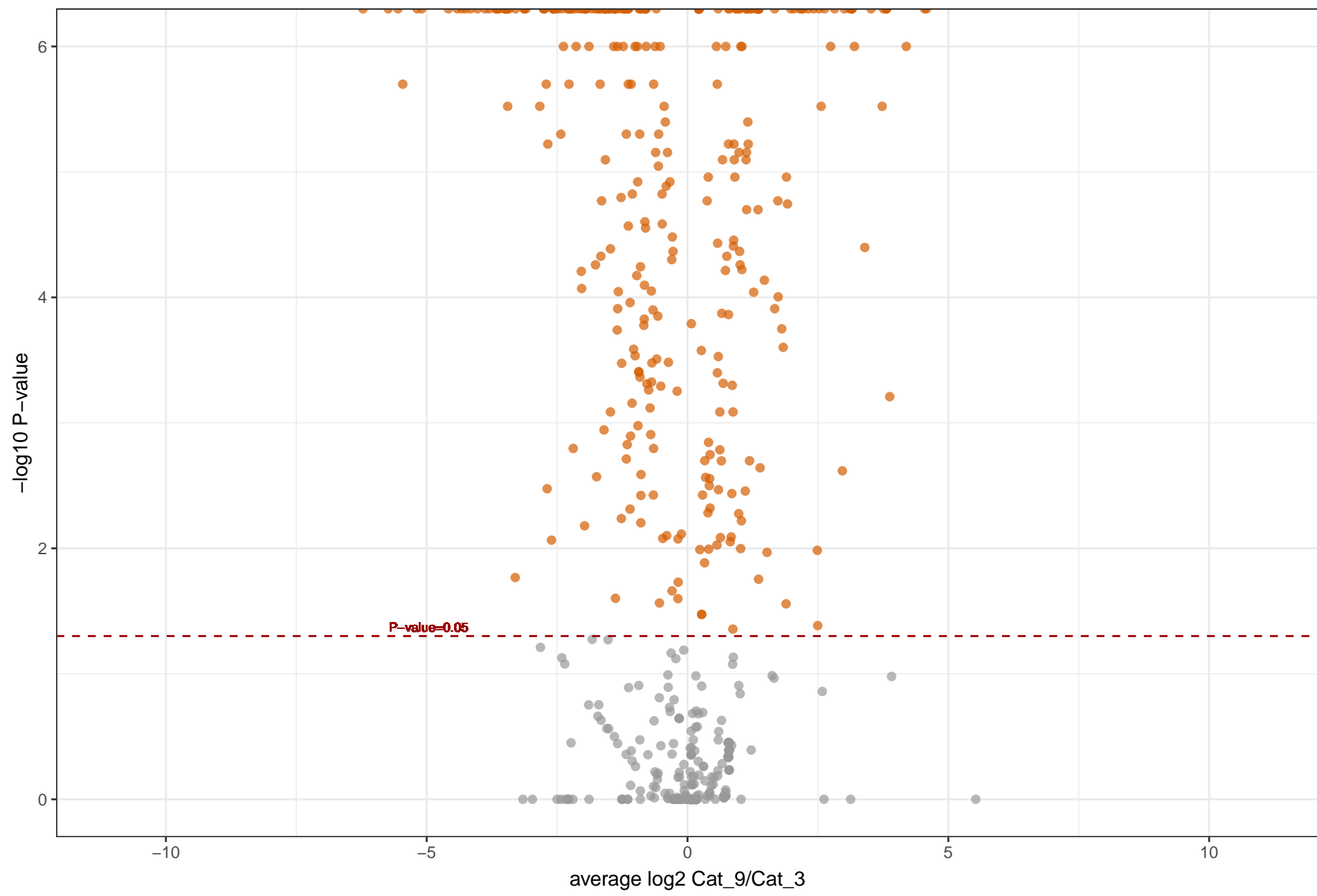

Supplement: Figure 6—source data 1. — Individual data from all figures involving small datasets displayed in individual tabs of this source file. This includes Figures 1B and 2A-F, Figure 3B, Figure 4, Figure 1—figure supplement 1 and Figure 2—figure supplement 1. [file elife-75798-fig6-data1.zip › Flores_Data/AF1_Cat_9.Cat_3-volcano_AFCat1.pdf]

Value-ordered fold change

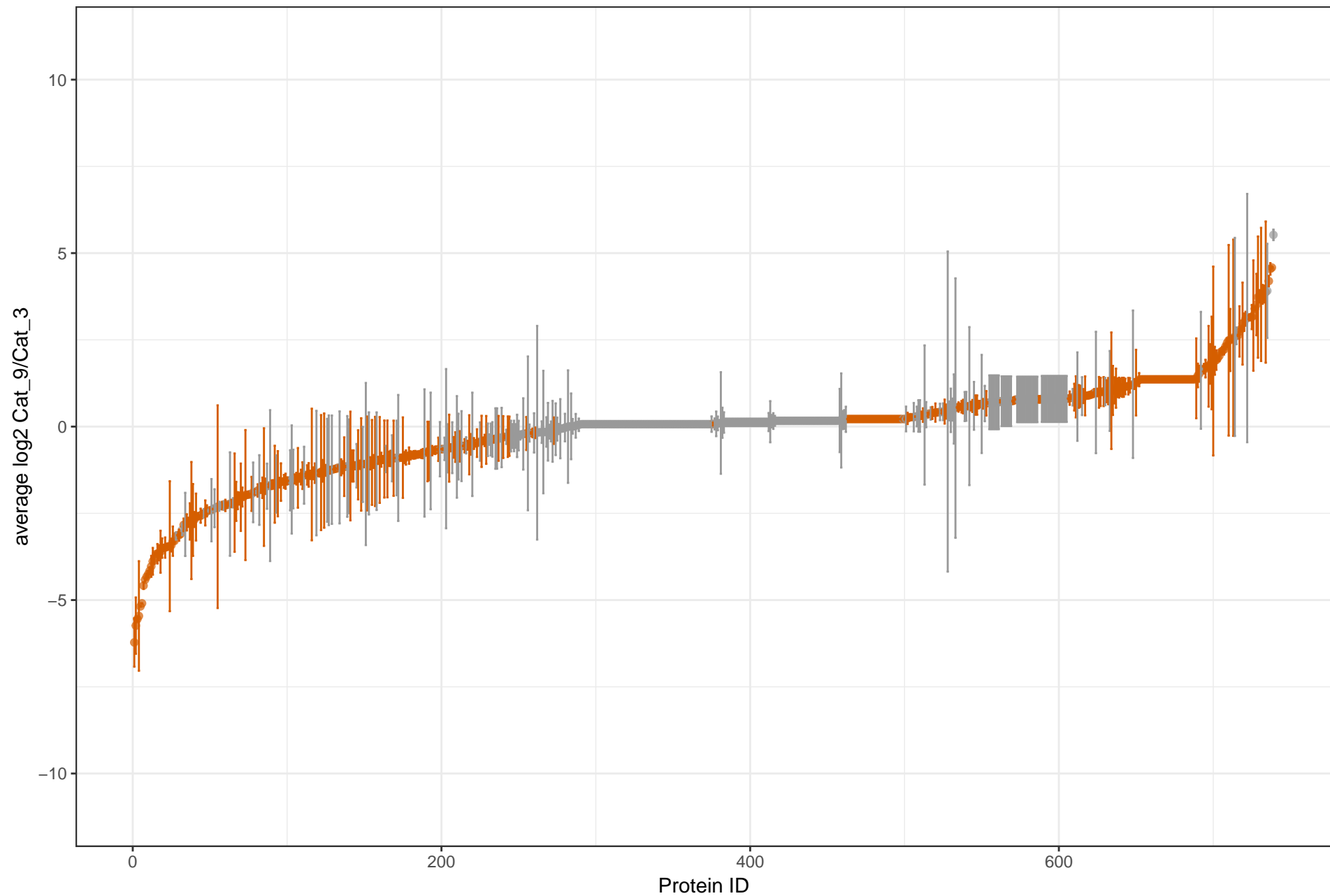

Supplement: Figure 6—source data 1. — Individual data from all figures involving small datasets displayed in individual tabs of this source file. This includes Figures 1B and 2A-F, Figure 3B, Figure 4, Figure 1—figure supplement 1 and Figure 2—figure supplement 1. [file elife-75798-fig6-data1.zip › Flores_Data/AF1_Cat_9.Cat_3-value-ordered-log-ratio_AFCat1.pdf]

MA plot

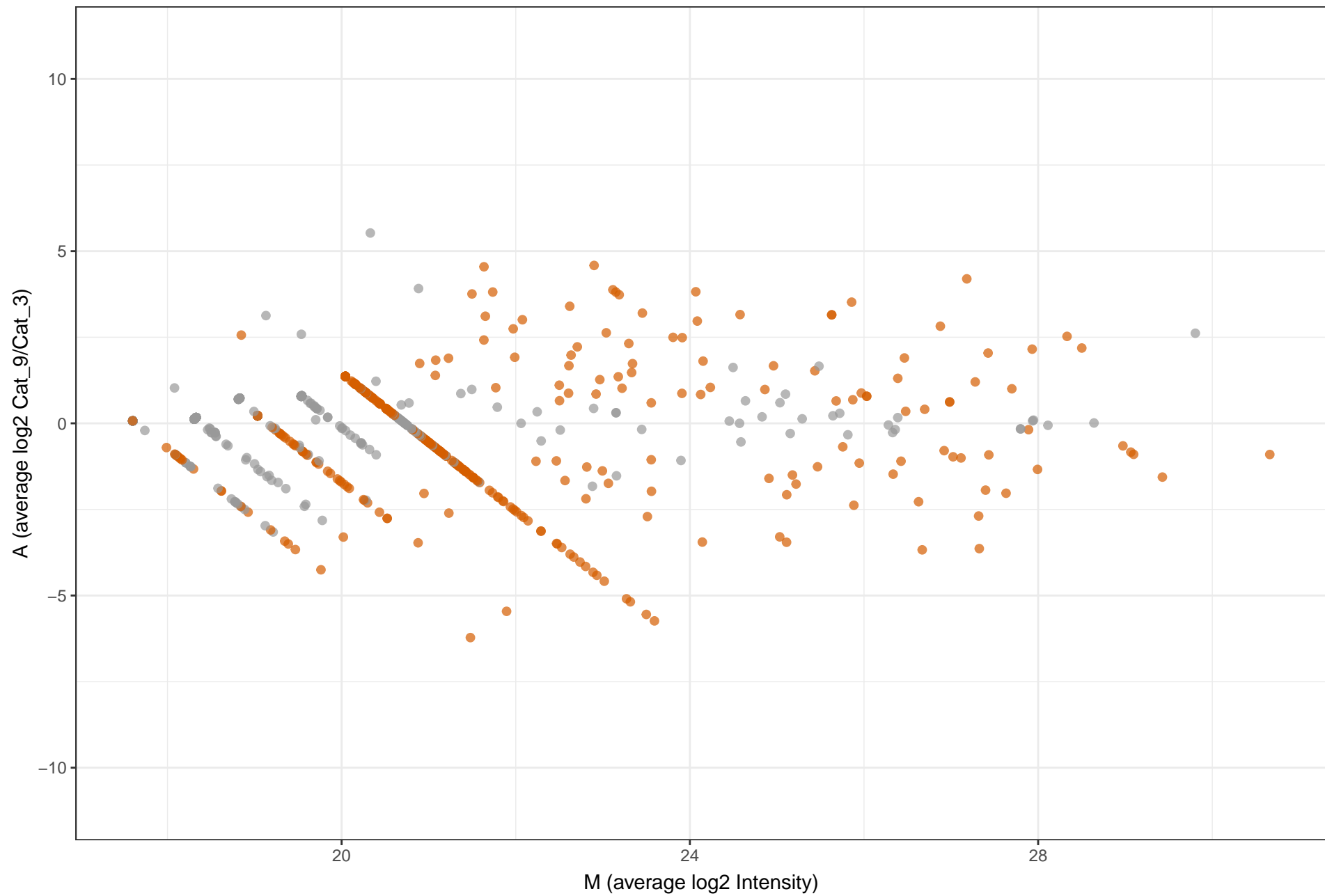

Supplement: Figure 6—source data 1. — Individual data from all figures involving small datasets displayed in individual tabs of this source file. This includes Figures 1B and 2A-F, Figure 3B, Figure 4, Figure 1—figure supplement 1 and Figure 2—figure supplement 1. [file elife-75798-fig6-data1.zip › Flores_Data/AF1_Cat_9.Cat_3-MA_AFCat1.pdf]

P-value vs Fold change

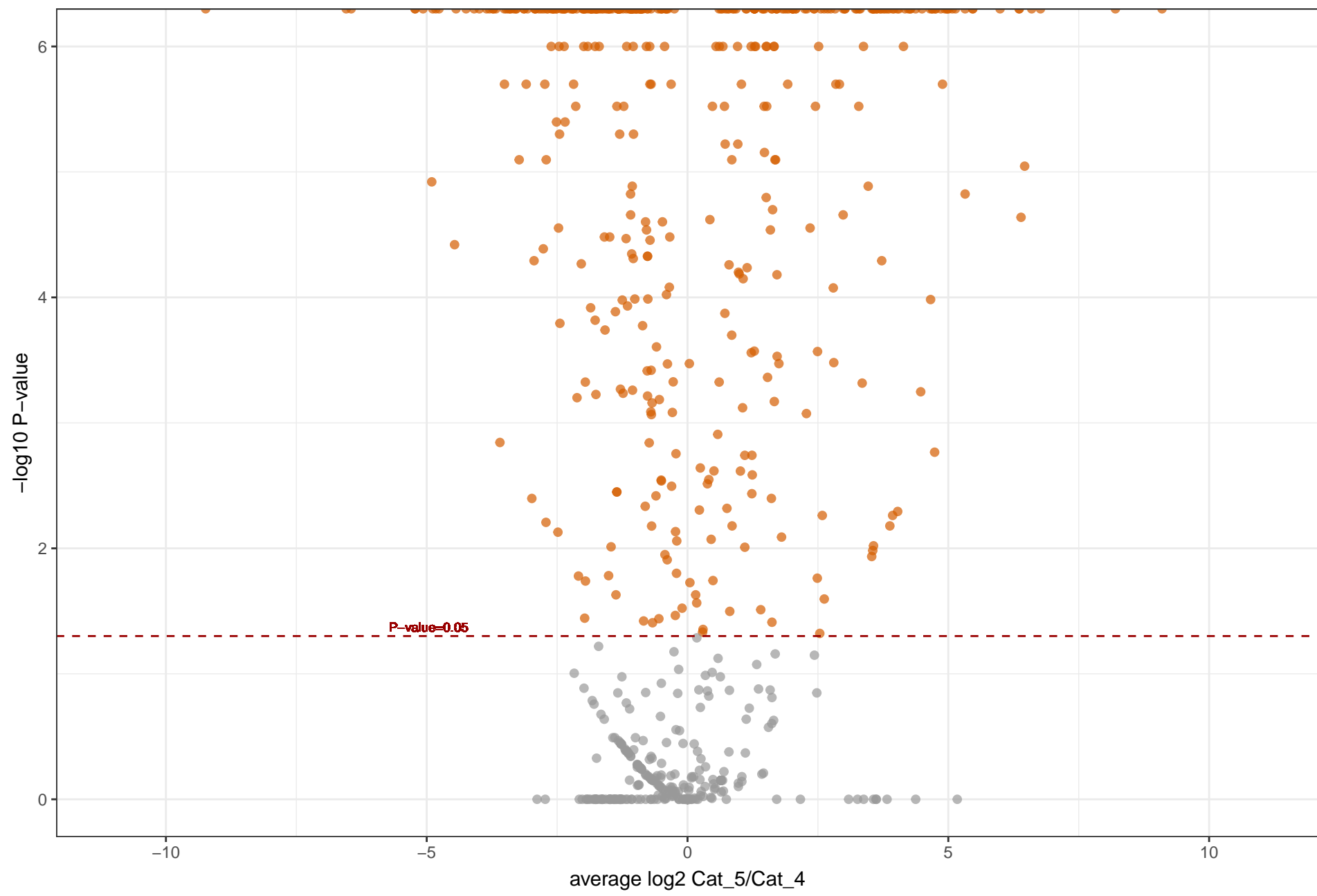

Supplement: Figure 6—source data 1. — Individual data from all figures involving small datasets displayed in individual tabs of this source file. This includes Figures 1B and 2A-F, Figure 3B, Figure 4, Figure 1—figure supplement 1 and Figure 2—figure supplement 1. [file elife-75798-fig6-data1.zip › Flores_Data/AF1_Cat_5.Cat_4-volcano_AFCat1.pdf]

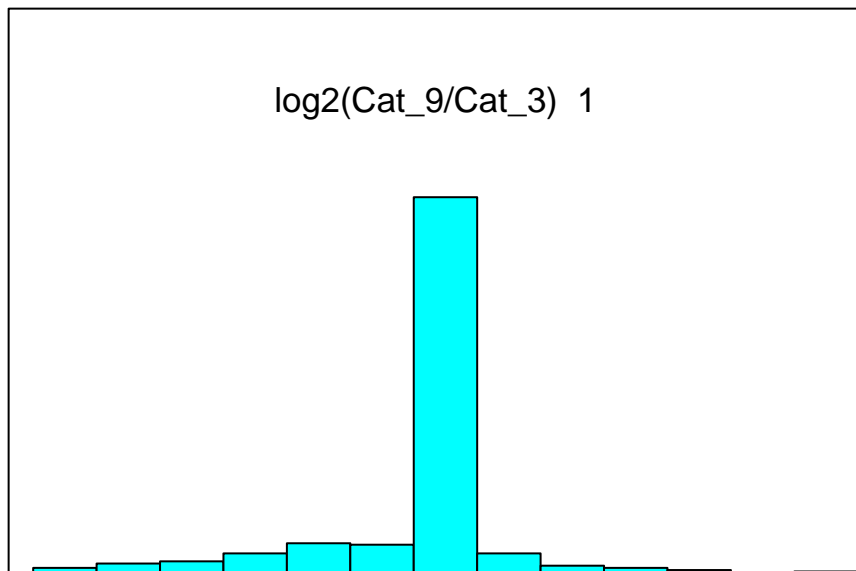

0.72

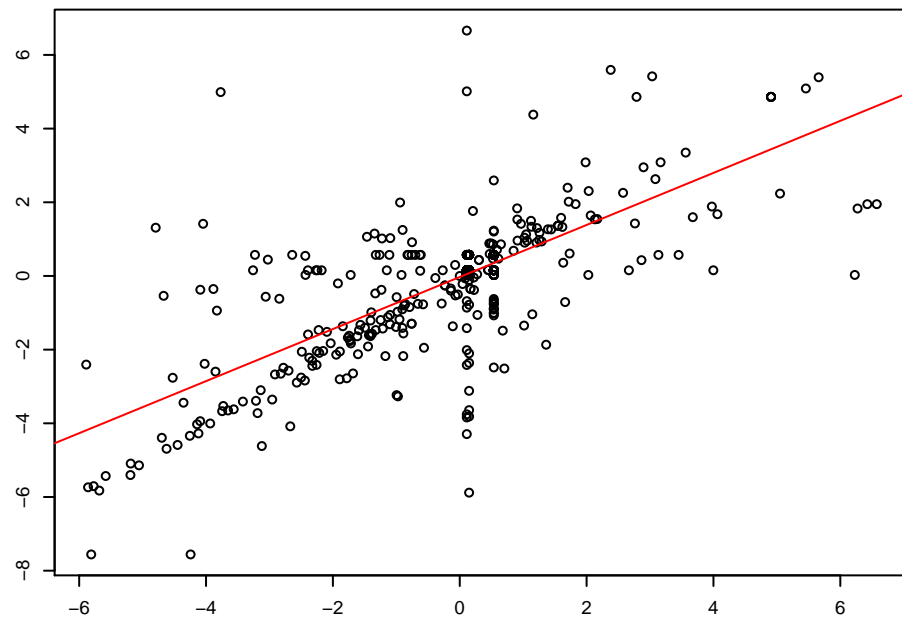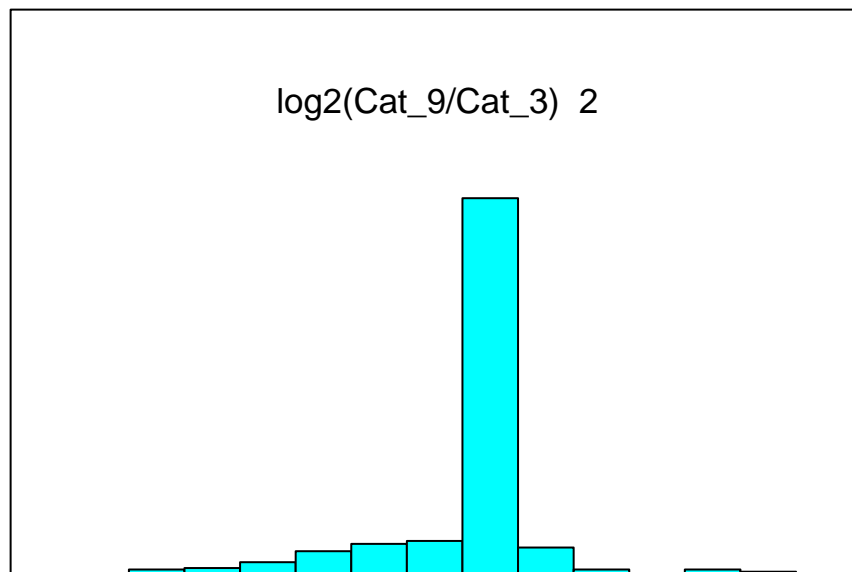

Supplement: Figure 6—source data 1. — Individual data from all figures involving small datasets displayed in individual tabs of this source file. This includes Figures 1B and 2A-F, Figure 3B, Figure 4, Figure 1—figure supplement 1 and Figure 2—figure supplement 1. [file elife-75798-fig6-data1.zip › Flores_Data/AF1_Cat_9.Cat_3-reproducibility_AFCat1.pdf]

Value-ordered fold change

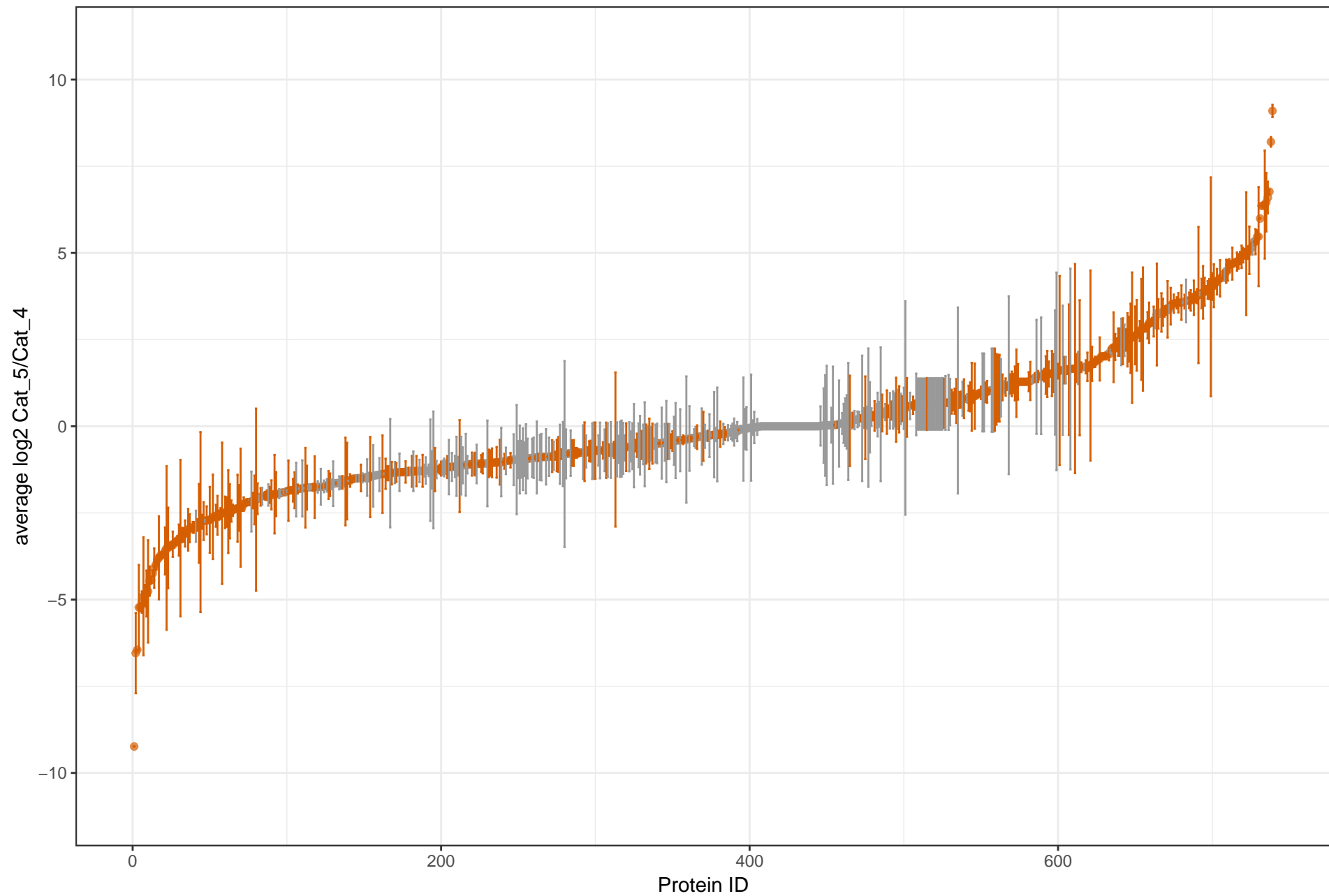

Supplement: Figure 6—source data 1. — Individual data from all figures involving small datasets displayed in individual tabs of this source file. This includes Figures 1B and 2A-F, Figure 3B, Figure 4, Figure 1—figure supplement 1 and Figure 2—figure supplement 1. [file elife-75798-fig6-data1.zip › Flores_Data/AF1_Cat_5.Cat_4-value-ordered-log-ratio_AFCat1.pdf]

MA plot

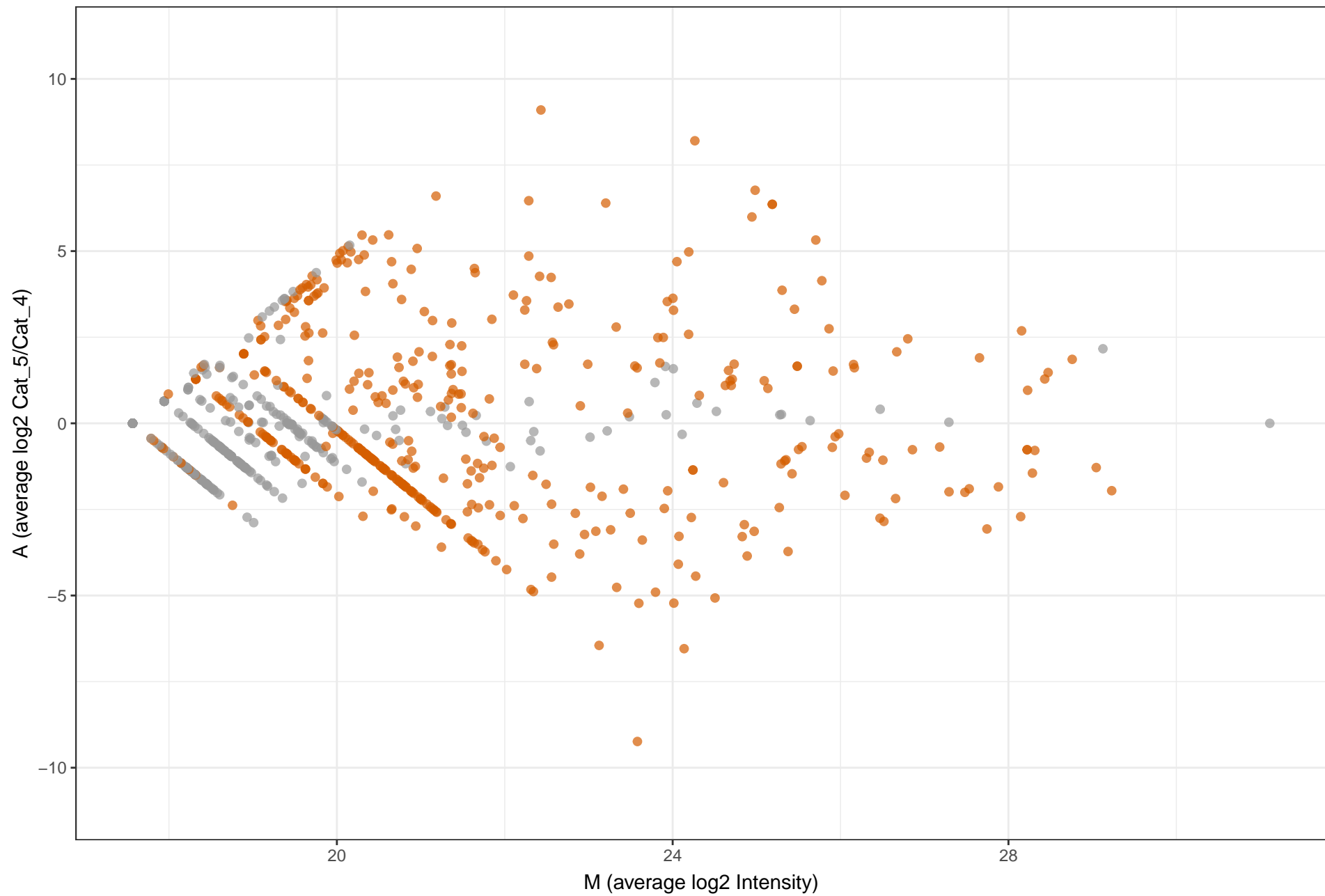

Supplement: Figure 6—source data 1. — Individual data from all figures involving small datasets displayed in individual tabs of this source file. This includes Figures 1B and 2A-F, Figure 3B, Figure 4, Figure 1—figure supplement 1 and Figure 2—figure supplement 1. [file elife-75798-fig6-data1.zip › Flores_Data/AF1_Cat_5.Cat_4-MA_AFCat1.pdf]

P-value vs Fold change

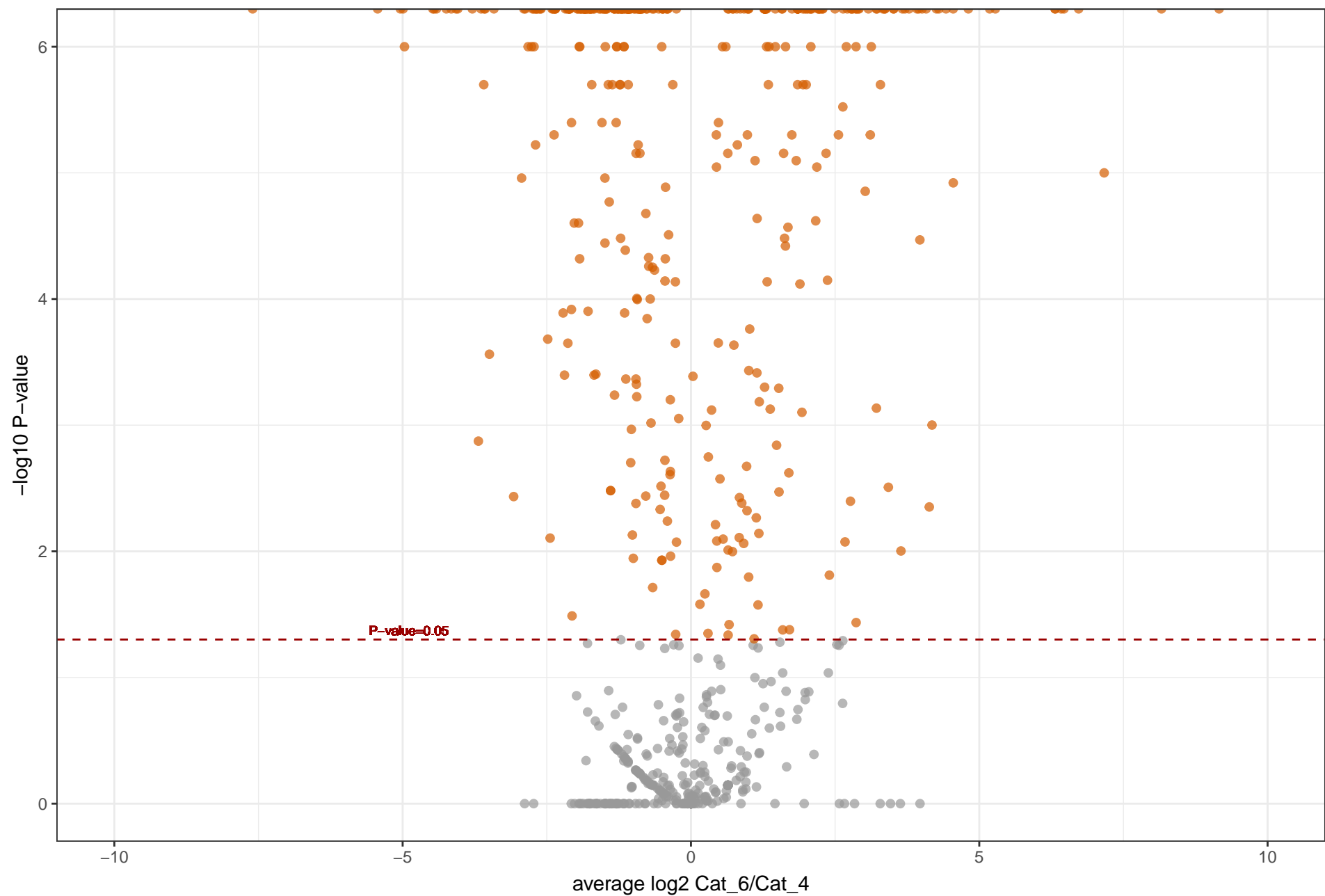

Supplement: Figure 6—source data 1. — Individual data from all figures involving small datasets displayed in individual tabs of this source file. This includes Figures 1B and 2A-F, Figure 3B, Figure 4, Figure 1—figure supplement 1 and Figure 2—figure supplement 1. [file elife-75798-fig6-data1.zip › Flores_Data/AF1_Cat_6.Cat_4-volcano_AFCat1.pdf]

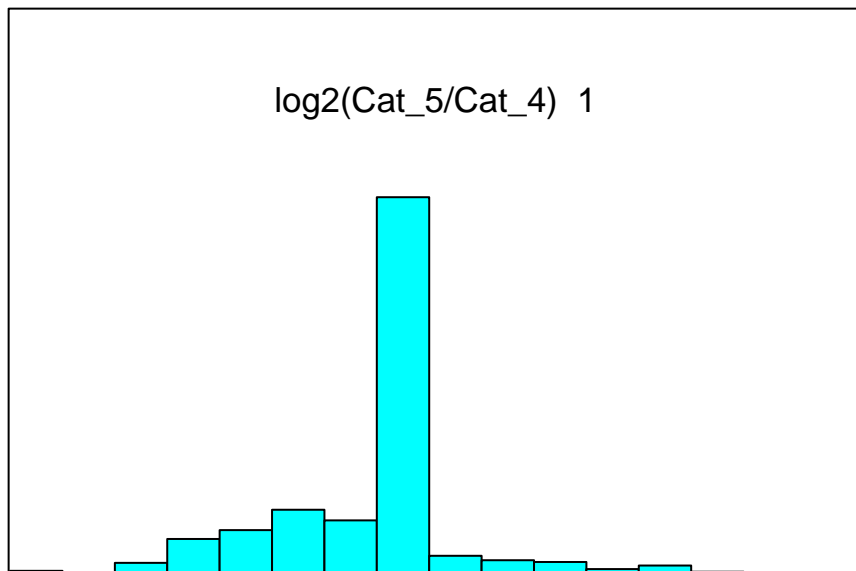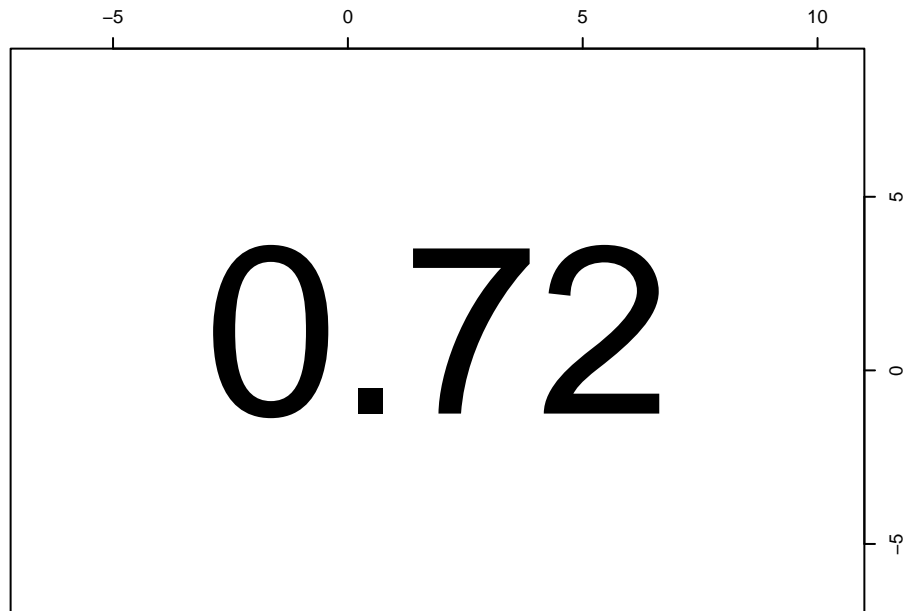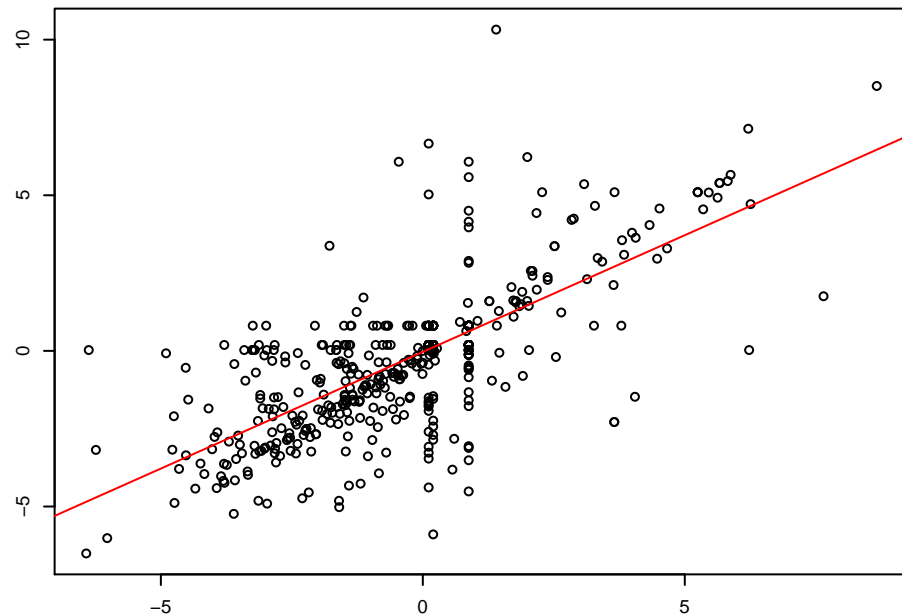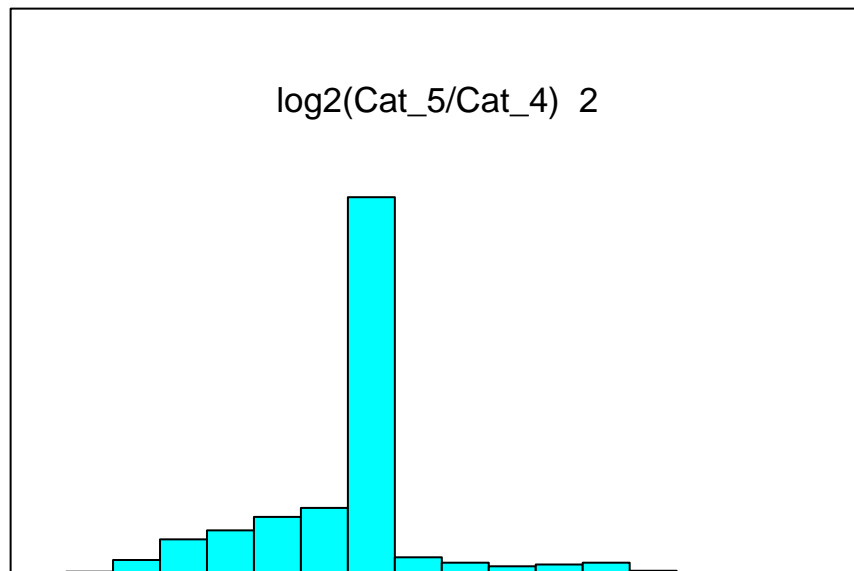

Supplement: Figure 6—source data 1. — Individual data from all figures involving small datasets displayed in individual tabs of this source file. This includes Figures 1B and 2A-F, Figure 3B, Figure 4, Figure 1—figure supplement 1 and Figure 2—figure supplement 1. [file elife-75798-fig6-data1.zip › Flores_Data/AF1_Cat_5.Cat_4-reproducibility_AFCat1.pdf]

Value-ordered fold change

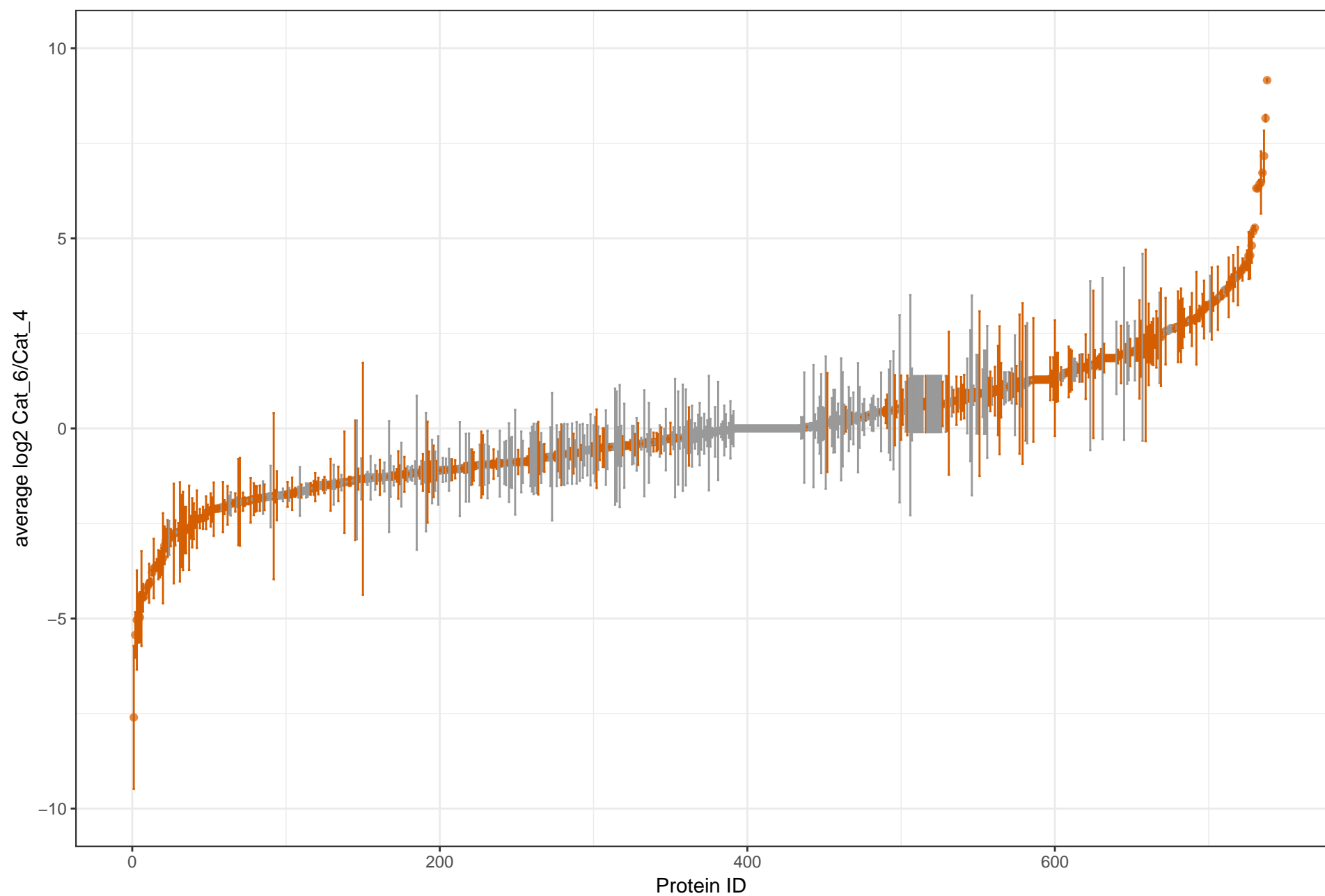

Supplement: Figure 6—source data 1. — Individual data from all figures involving small datasets displayed in individual tabs of this source file. This includes Figures 1B and 2A-F, Figure 3B, Figure 4, Figure 1—figure supplement 1 and Figure 2—figure supplement 1. [file elife-75798-fig6-data1.zip › Flores_Data/AF1_Cat_6.Cat_4-value-ordered-log-ratio_AFCat1.pdf]

P-value vs Fold change

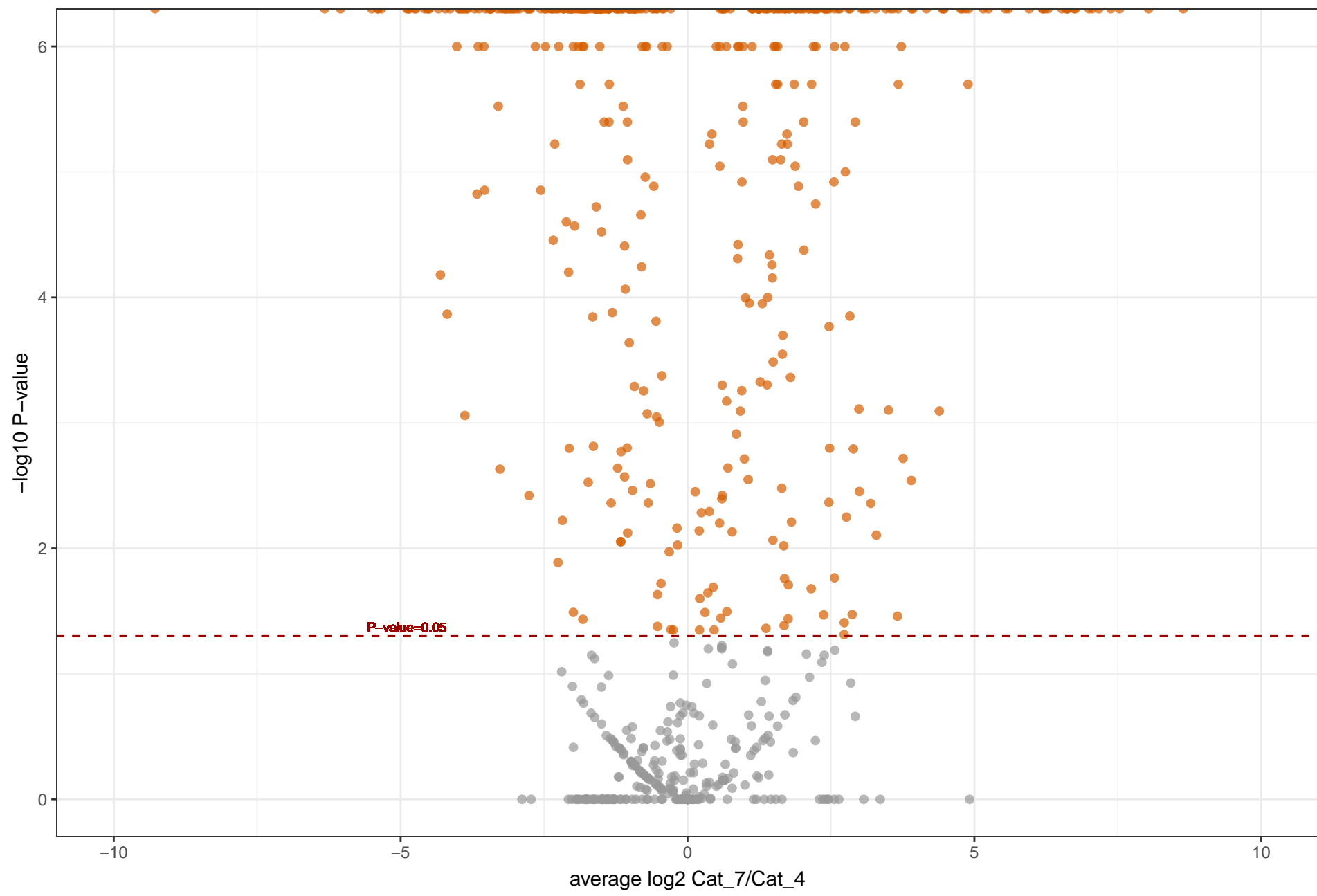

Supplement: Figure 6—source data 1. — Individual data from all figures involving small datasets displayed in individual tabs of this source file. This includes Figures 1B and 2A-F, Figure 3B, Figure 4, Figure 1—figure supplement 1 and Figure 2—figure supplement 1. [file elife-75798-fig6-data1.zip › Flores_Data/AF1_Cat_7.Cat_4-volcano_AFCat1.pdf]

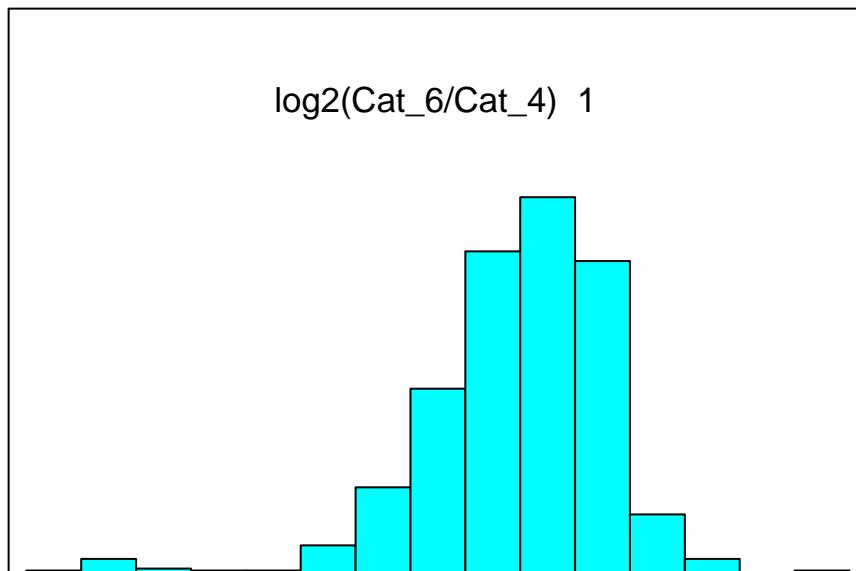

-5 0 5

5  
0  
-5

0.84

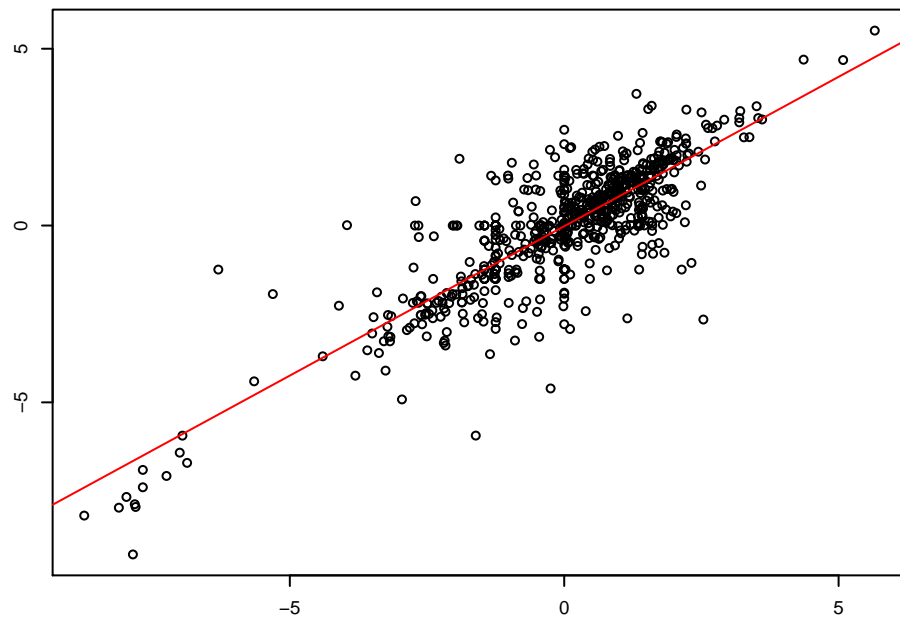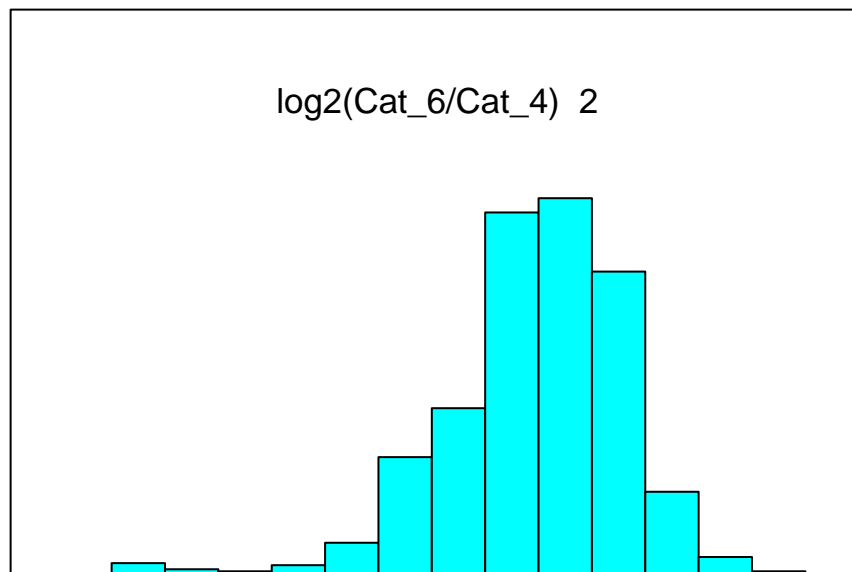

Supplement: Figure 6—source data 1. — Individual data from all figures involving small datasets displayed in individual tabs of this source file. This includes Figures 1B and 2A-F, Figure 3B, Figure 4, Figure 1—figure supplement 1 and Figure 2—figure supplement 1. [file elife-75798-fig6-data1.zip › Flores_Data/AF1_Cat_6.Cat_4-reproducibility_AFCat1.pdf]

MA plot

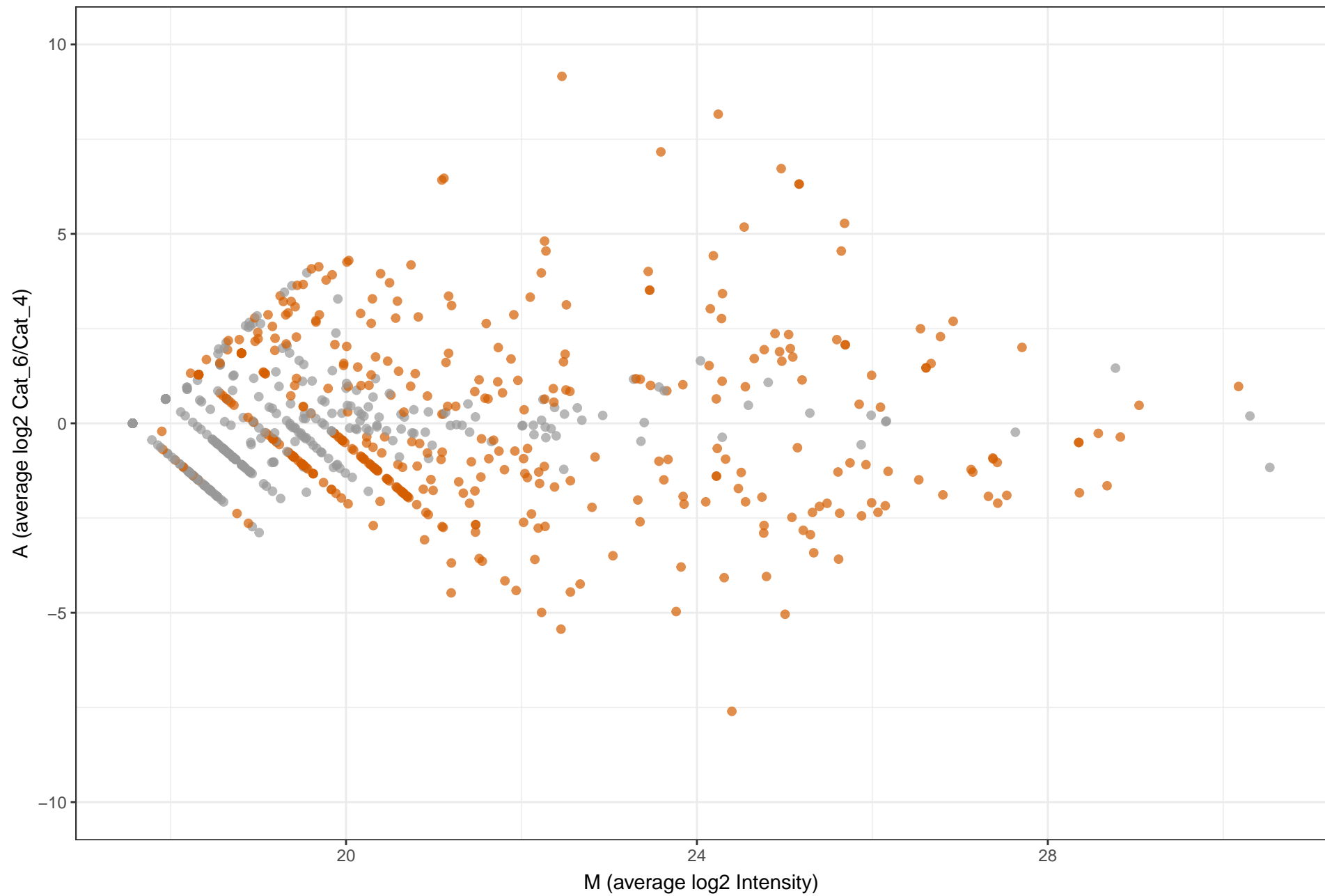

Supplement: Figure 6—source data 1. — Individual data from all figures involving small datasets displayed in individual tabs of this source file. This includes Figures 1B and 2A-F, Figure 3B, Figure 4, Figure 1—figure supplement 1 and Figure 2—figure supplement 1. [file elife-75798-fig6-data1.zip › Flores_Data/AF1_Cat_6.Cat_4-MA_AFCat1.pdf]

Value-ordered fold change

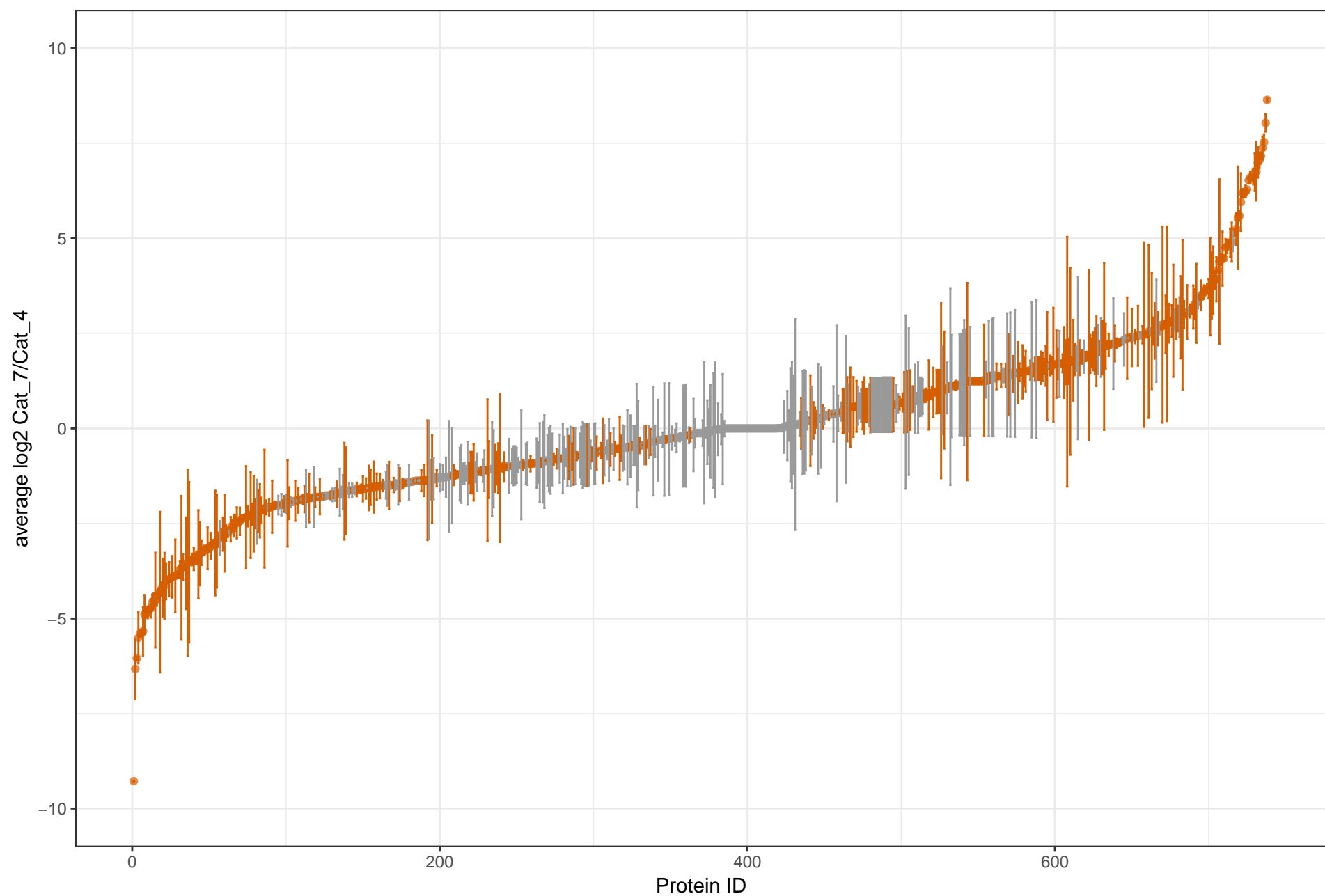

Supplement: Figure 6—source data 1. — Individual data from all figures involving small datasets displayed in individual tabs of this source file. This includes Figures 1B and 2A-F, Figure 3B, Figure 4, Figure 1—figure supplement 1 and Figure 2—figure supplement 1. [file elife-75798-fig6-data1.zip › Flores_Data/AF1_Cat_7.Cat_4-value-ordered-log-ratio_AFCat1.pdf]

MA plot

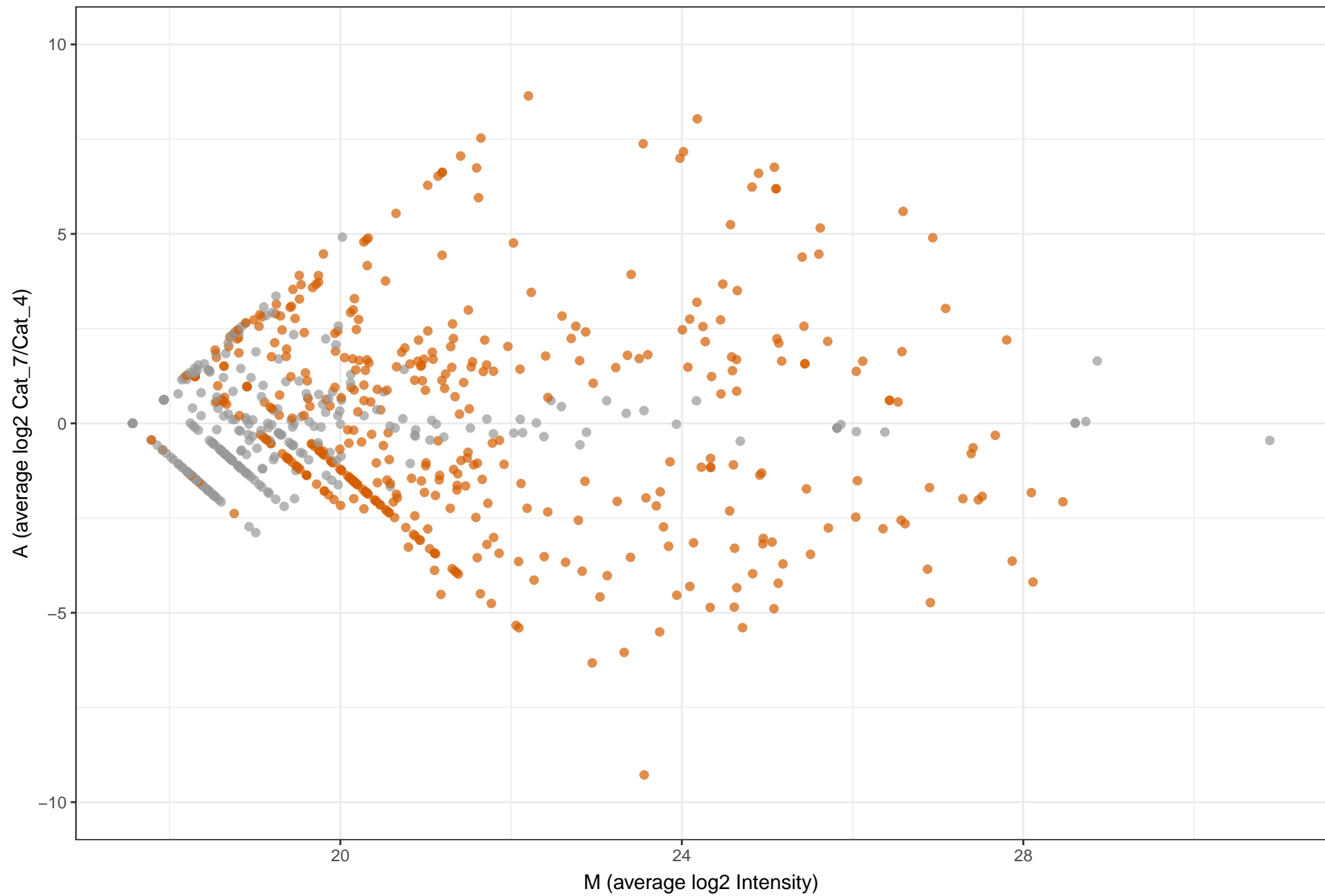

Supplement: Figure 6—source data 1. — Individual data from all figures involving small datasets displayed in individual tabs of this source file. This includes Figures 1B and 2A-F, Figure 3B, Figure 4, Figure 1—figure supplement 1 and Figure 2—figure supplement 1. [file elife-75798-fig6-data1.zip › Flores_Data/AF1_Cat_7.Cat_4-MA_AFCat1.pdf]

P-value vs Fold change

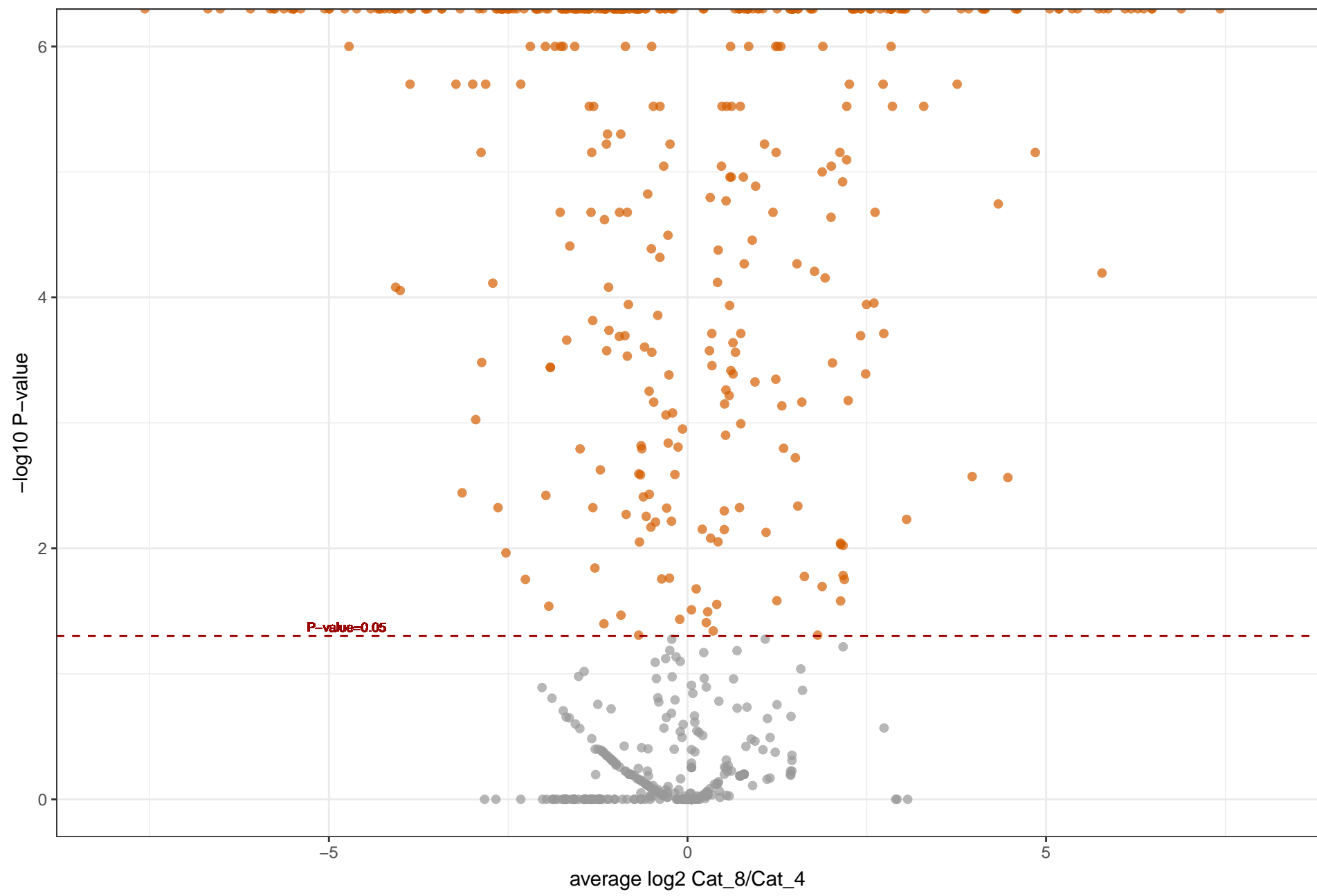

Supplement: Figure 6—source data 1. — Individual data from all figures involving small datasets displayed in individual tabs of this source file. This includes Figures 1B and 2A-F, Figure 3B, Figure 4, Figure 1—figure supplement 1 and Figure 2—figure supplement 1. [file elife-75798-fig6-data1.zip › Flores_Data/AF1_Cat_8.Cat_4-volcano_AFCat1.pdf]

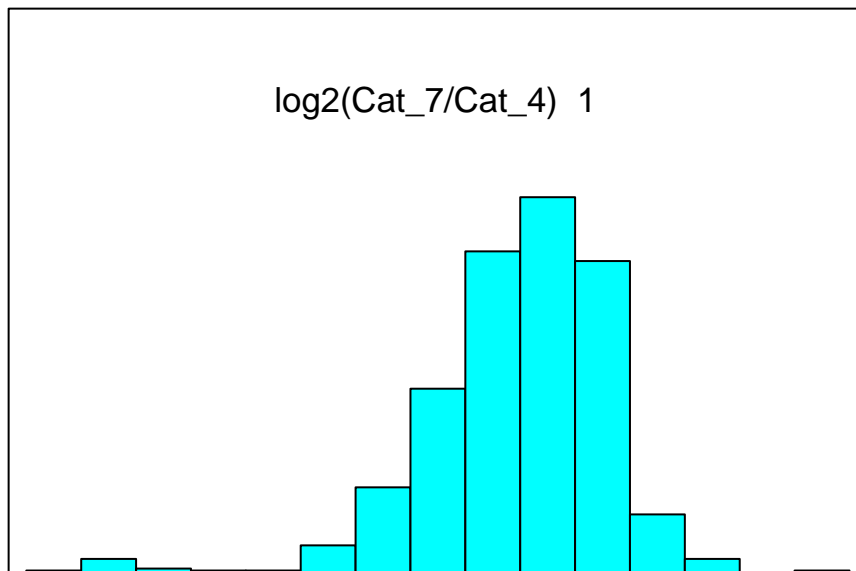

-5 0 5

5  
0  
-5

0.84

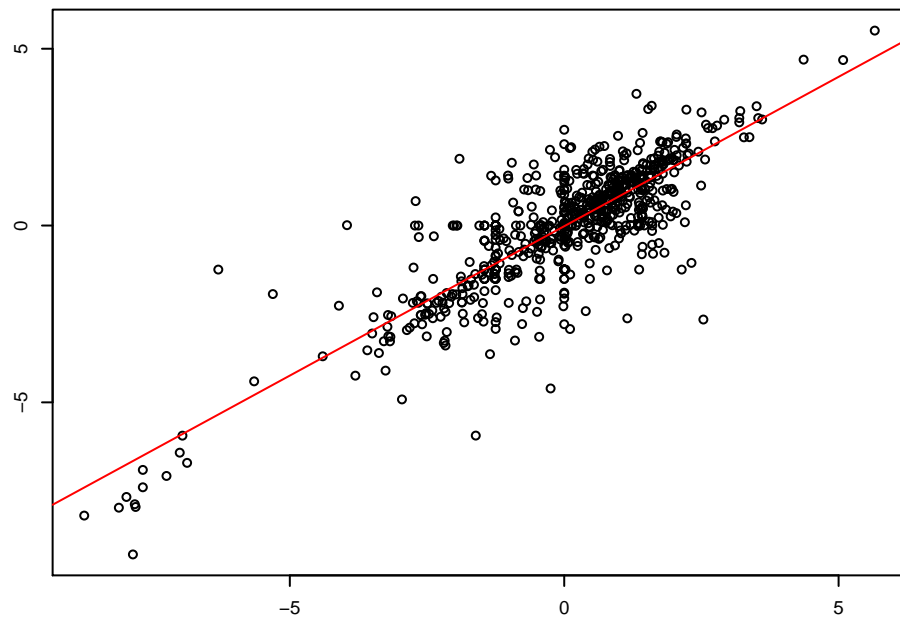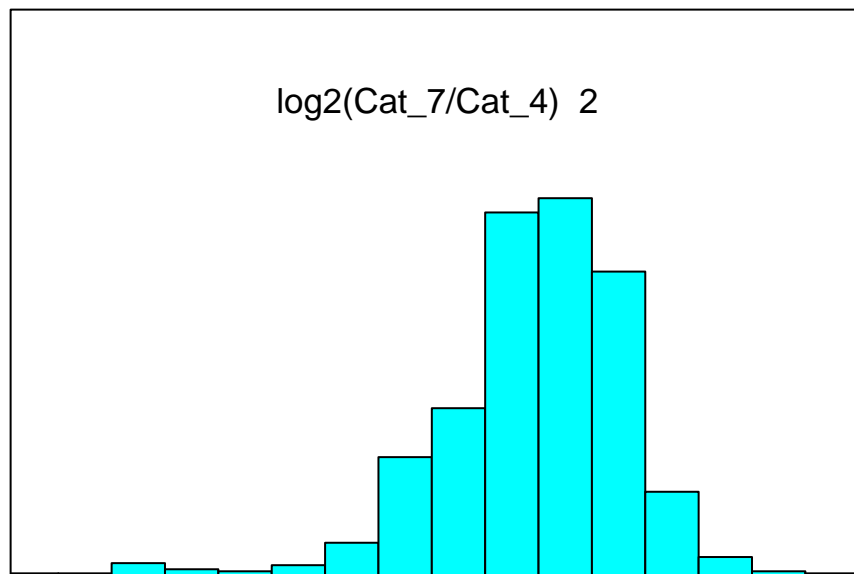

Supplement: Figure 6—source data 1. — Individual data from all figures involving small datasets displayed in individual tabs of this source file. This includes Figures 1B and 2A-F, Figure 3B, Figure 4, Figure 1—figure supplement 1 and Figure 2—figure supplement 1. [file elife-75798-fig6-data1.zip › Flores_Data/AF1_Cat_7.Cat_4-reproducibility_AFCat1.pdf]

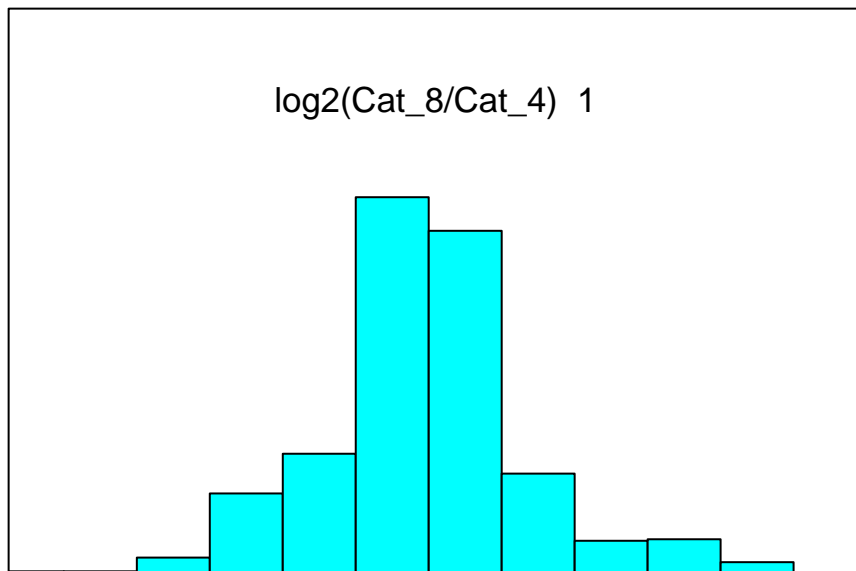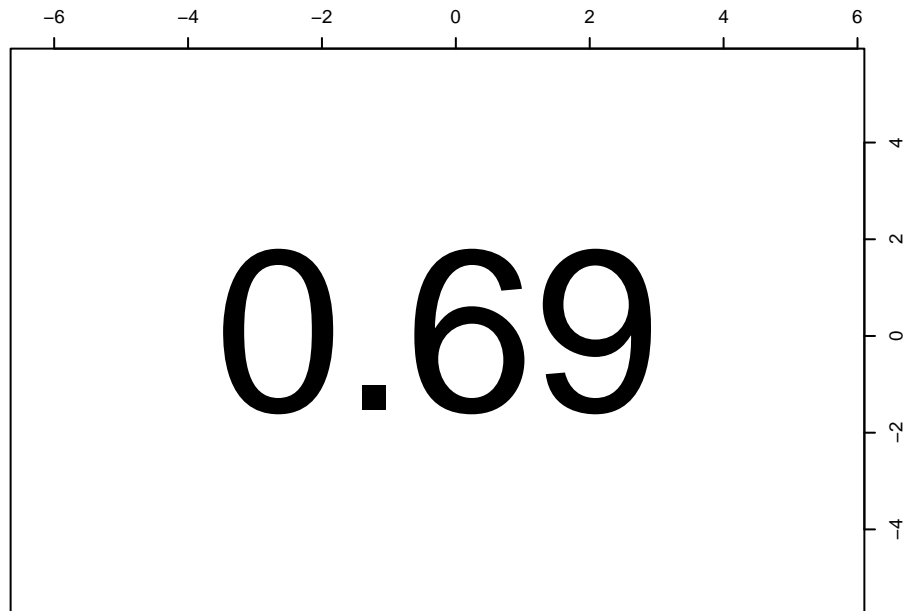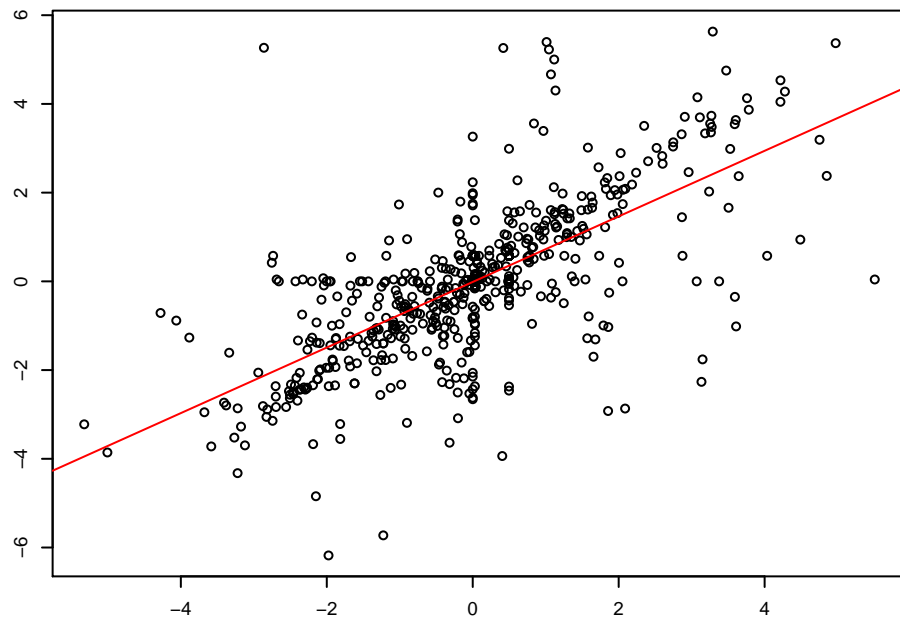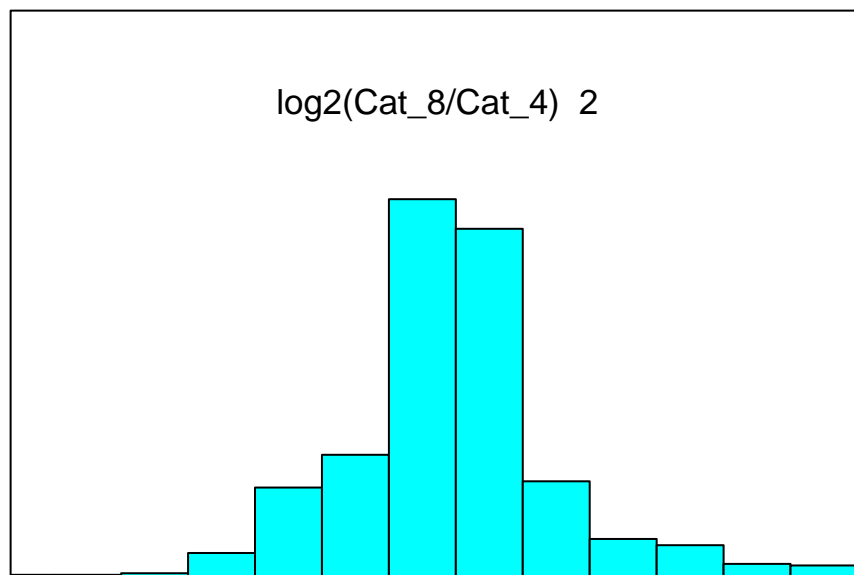

Supplement: Figure 6—source data 1. — Individual data from all figures involving small datasets displayed in individual tabs of this source file. This includes Figures 1B and 2A-F, Figure 3B, Figure 4, Figure 1—figure supplement 1 and Figure 2—figure supplement 1. [file elife-75798-fig6-data1.zip › Flores_Data/AF1_Cat_8.Cat_4-reproducibility_AFCat1.pdf]

MA plot

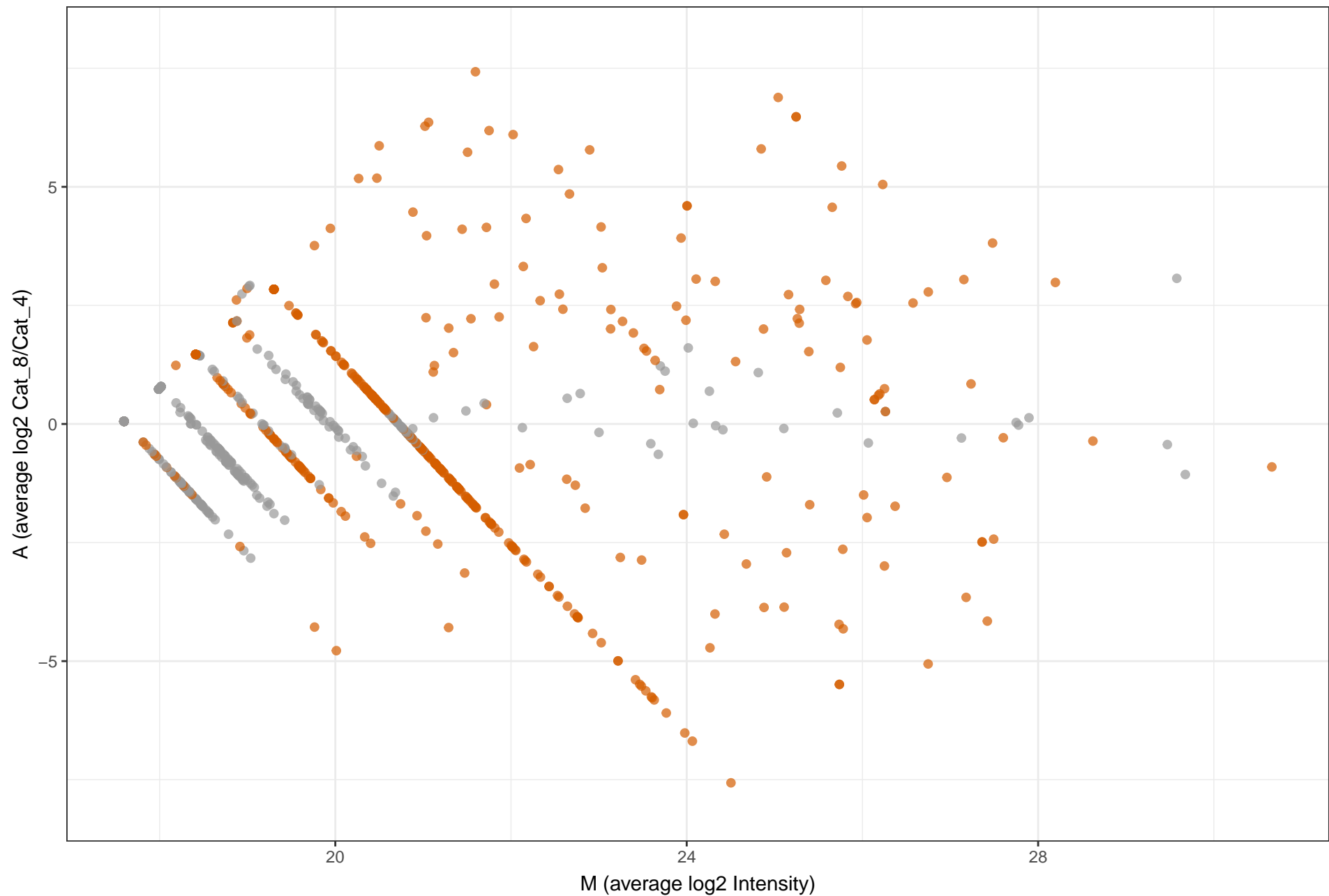

Supplement: Figure 6—source data 1. — Individual data from all figures involving small datasets displayed in individual tabs of this source file. This includes Figures 1B and 2A-F, Figure 3B, Figure 4, Figure 1—figure supplement 1 and Figure 2—figure supplement 1. [file elife-75798-fig6-data1.zip › Flores_Data/AF1_Cat_8.Cat_4-MA_AFCat1.pdf]

P-value vs Fold change

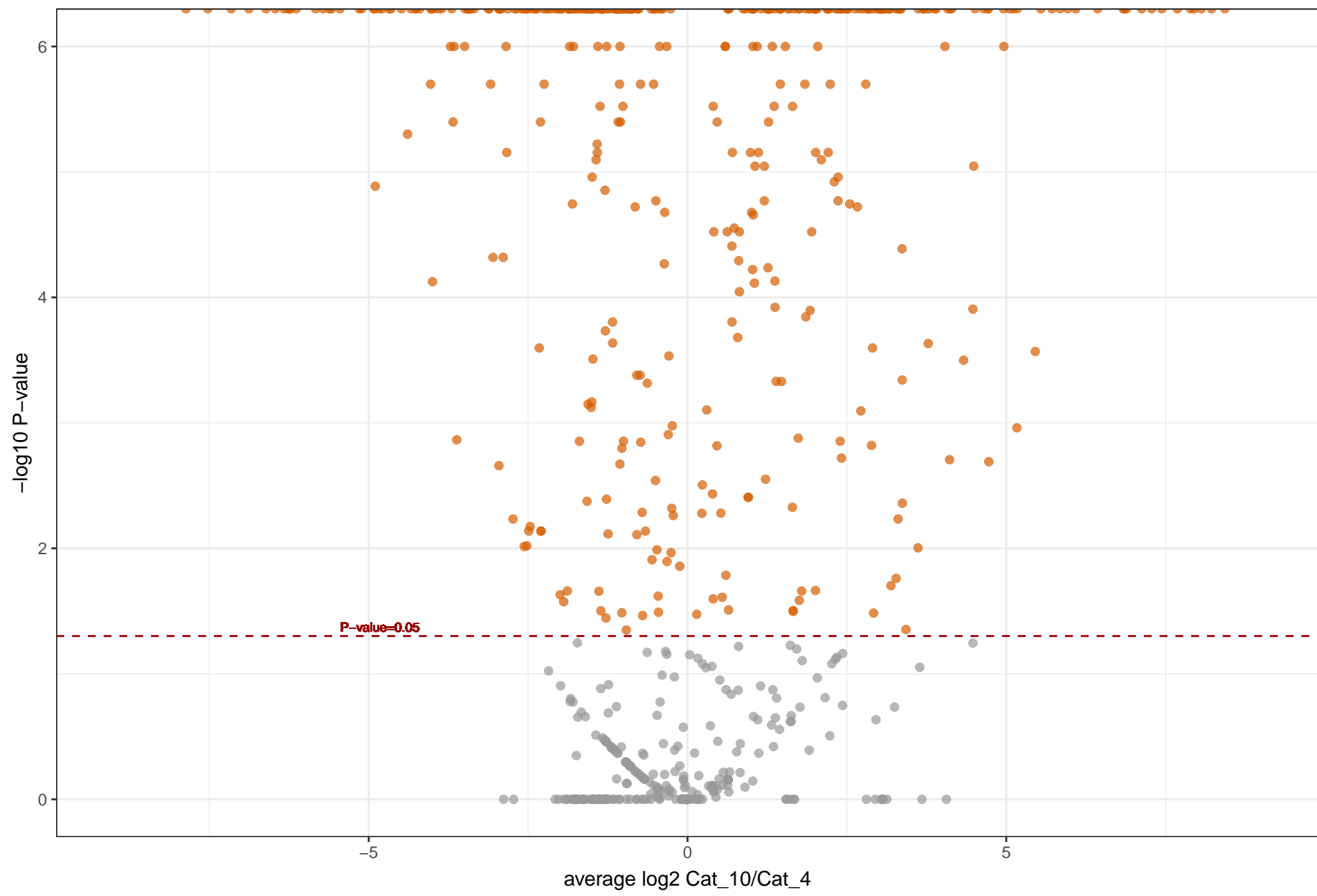

Supplement: Figure 6—source data 1. — Individual data from all figures involving small datasets displayed in individual tabs of this source file. This includes Figures 1B and 2A-F, Figure 3B, Figure 4, Figure 1—figure supplement 1 and Figure 2—figure supplement 1. [file elife-75798-fig6-data1.zip › Flores_Data/AF1_Cat_10.Cat_4-volcano_AFCat1.pdf]

Value-ordered fold change

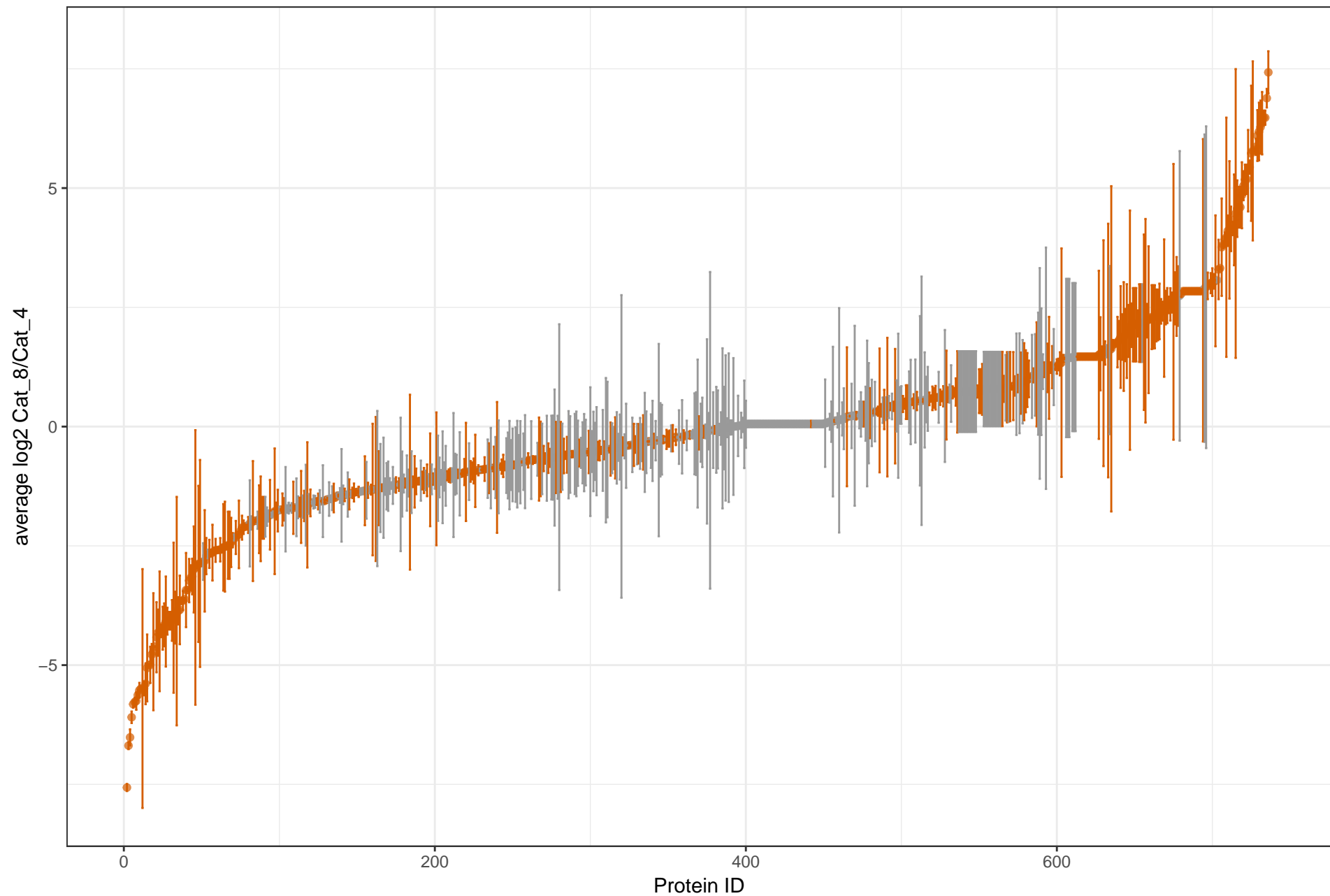

Supplement: Figure 6—source data 1. — Individual data from all figures involving small datasets displayed in individual tabs of this source file. This includes Figures 1B and 2A-F, Figure 3B, Figure 4, Figure 1—figure supplement 1 and Figure 2—figure supplement 1. [file elife-75798-fig6-data1.zip › Flores_Data/AF1_Cat_8.Cat_4-value-ordered-log-ratio_AFCat1.pdf]

MA plot

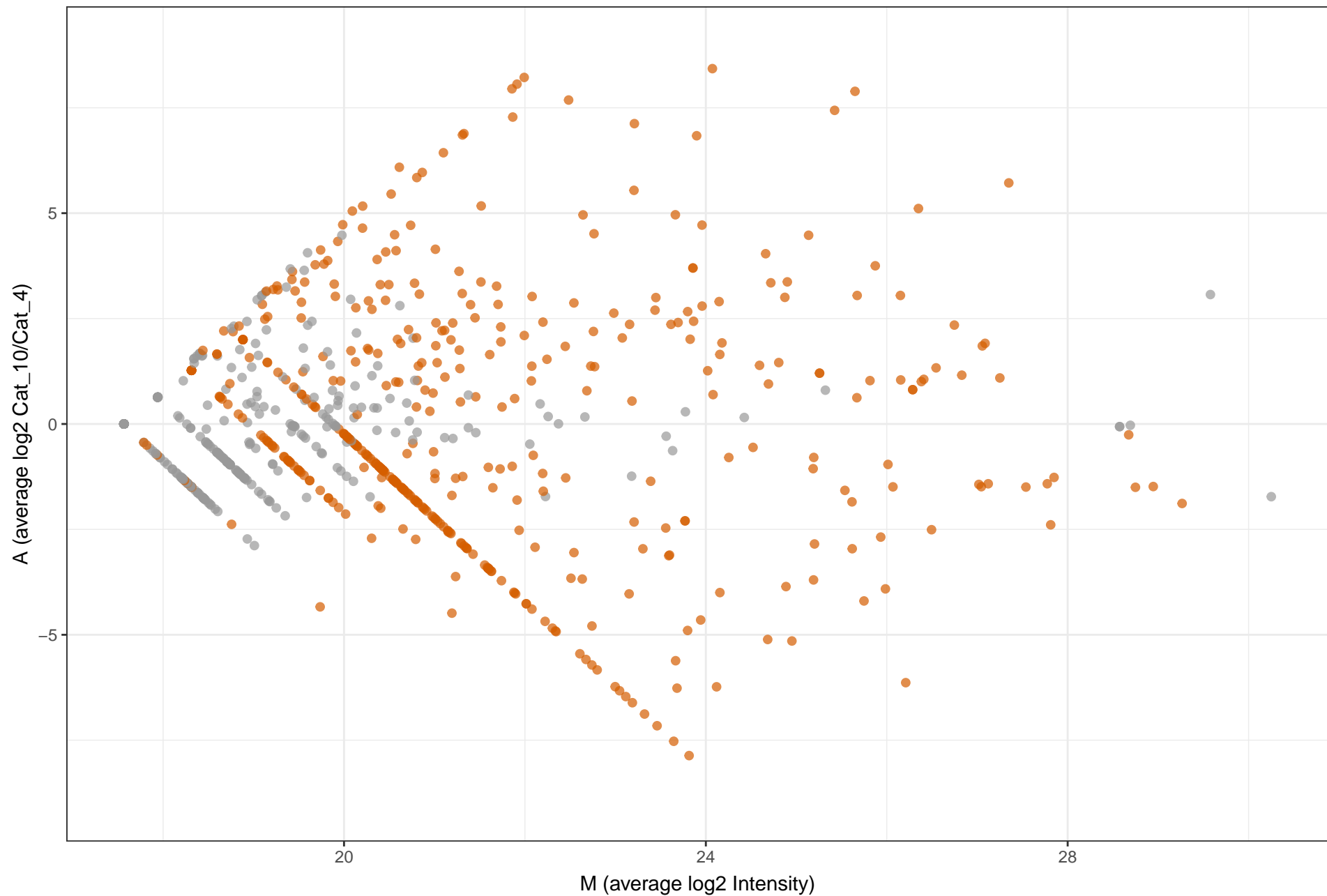

Supplement: Figure 6—source data 1. — Individual data from all figures involving small datasets displayed in individual tabs of this source file. This includes Figures 1B and 2A-F, Figure 3B, Figure 4, Figure 1—figure supplement 1 and Figure 2—figure supplement 1. [file elife-75798-fig6-data1.zip › Flores_Data/AF1_Cat_10.Cat_4-MA_AFCat1.pdf]

P-value vs Fold change

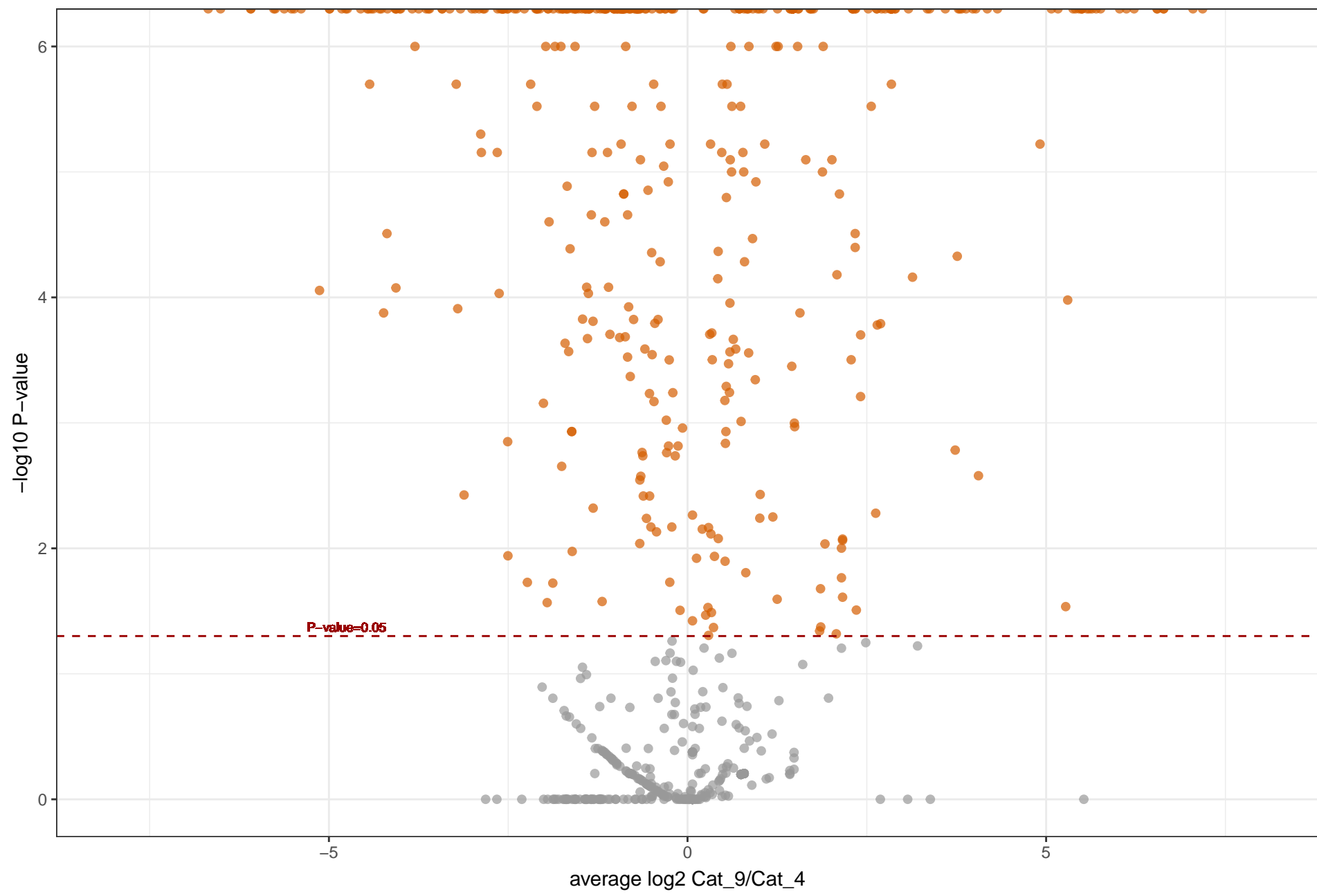

Supplement: Figure 6—source data 1. — Individual data from all figures involving small datasets displayed in individual tabs of this source file. This includes Figures 1B and 2A-F, Figure 3B, Figure 4, Figure 1—figure supplement 1 and Figure 2—figure supplement 1. [file elife-75798-fig6-data1.zip › Flores_Data/AF1_Cat_9.Cat_4-volcano_AFCat1.pdf]

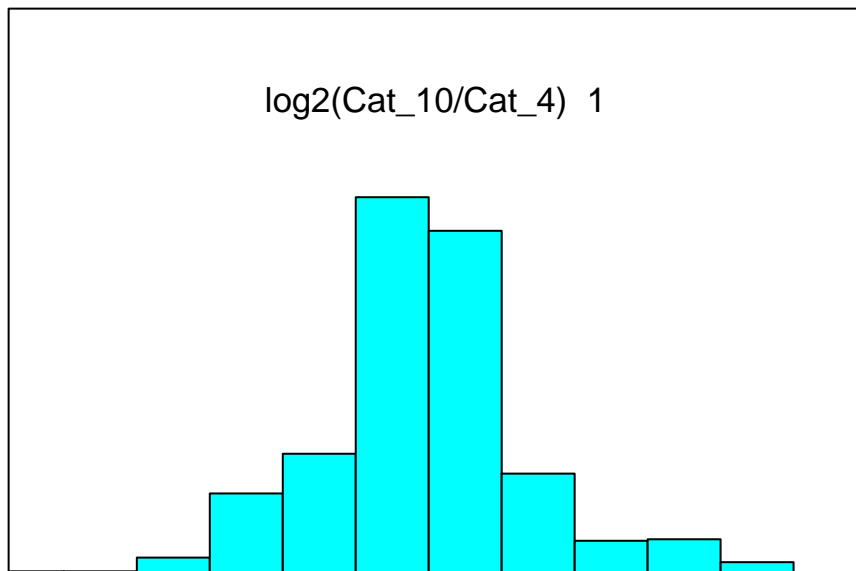

-6 -4 -2 0 2 4 6

4  
2  
0  
-2  
-4

0.69

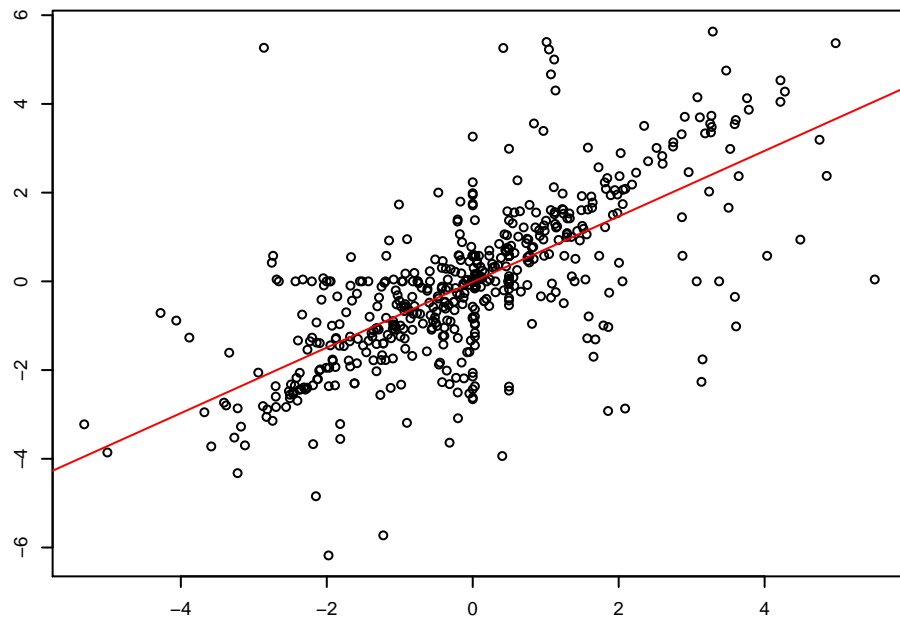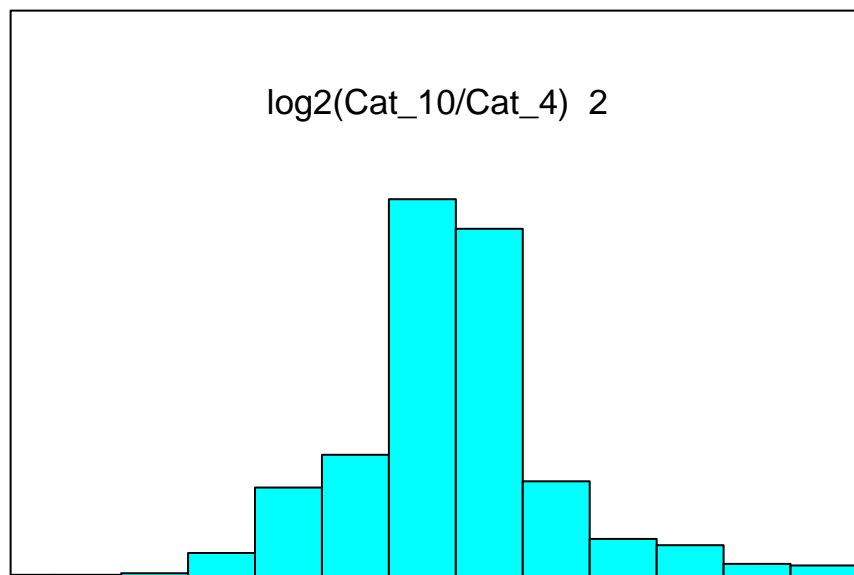

Supplement: Figure 6—source data 1. — Individual data from all figures involving small datasets displayed in individual tabs of this source file. This includes Figures 1B and 2A-F, Figure 3B, Figure 4, Figure 1—figure supplement 1 and Figure 2—figure supplement 1. [file elife-75798-fig6-data1.zip › Flores_Data/AF1_Cat_10.Cat_4-reproducibility_AFCat1.pdf]

Value-ordered fold change

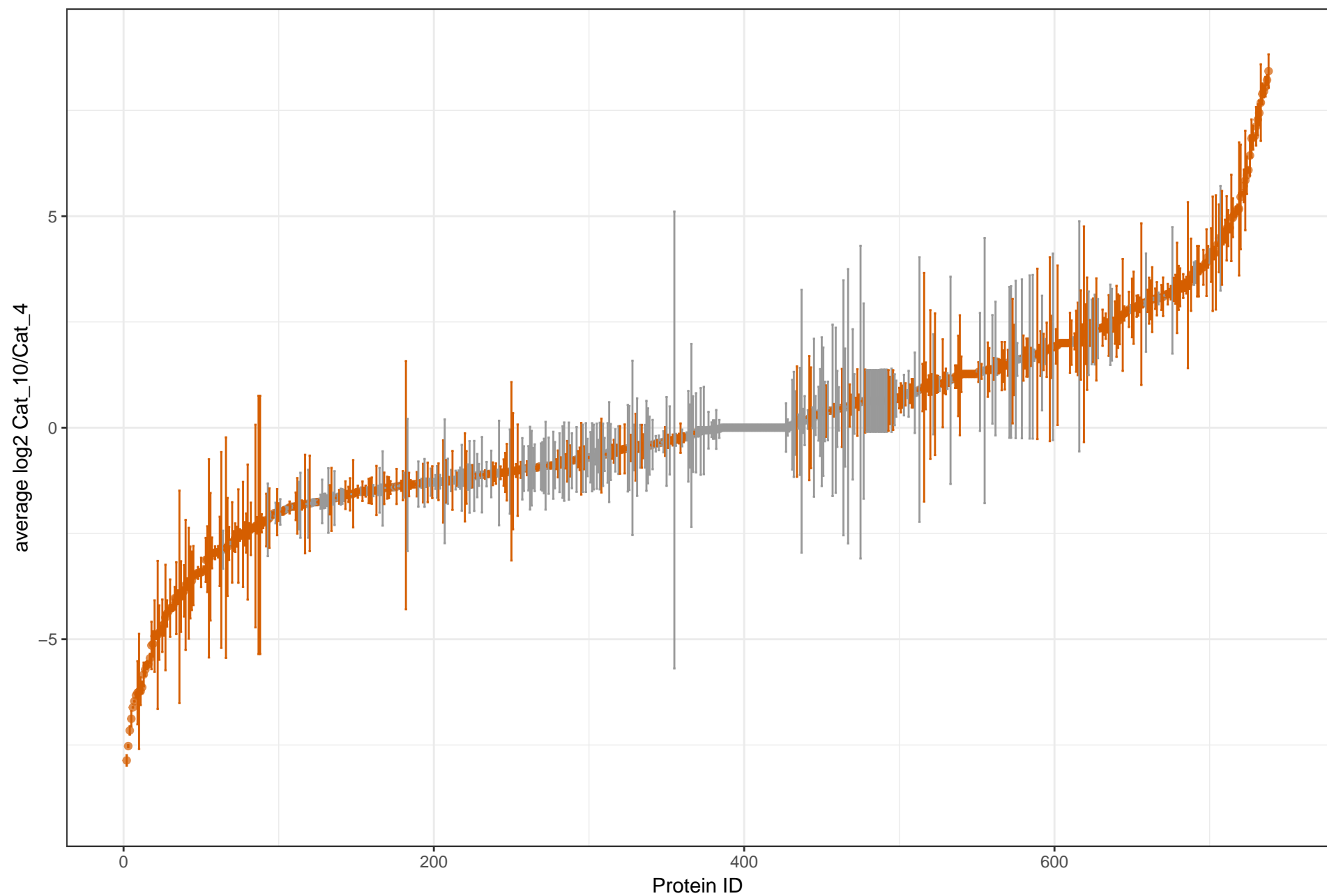

Supplement: Figure 6—source data 1. — Individual data from all figures involving small datasets displayed in individual tabs of this source file. This includes Figures 1B and 2A-F, Figure 3B, Figure 4, Figure 1—figure supplement 1 and Figure 2—figure supplement 1. [file elife-75798-fig6-data1.zip › Flores_Data/AF1_Cat_10.Cat_4-value-ordered-log-ratio_AFCat1.pdf]

MA plot

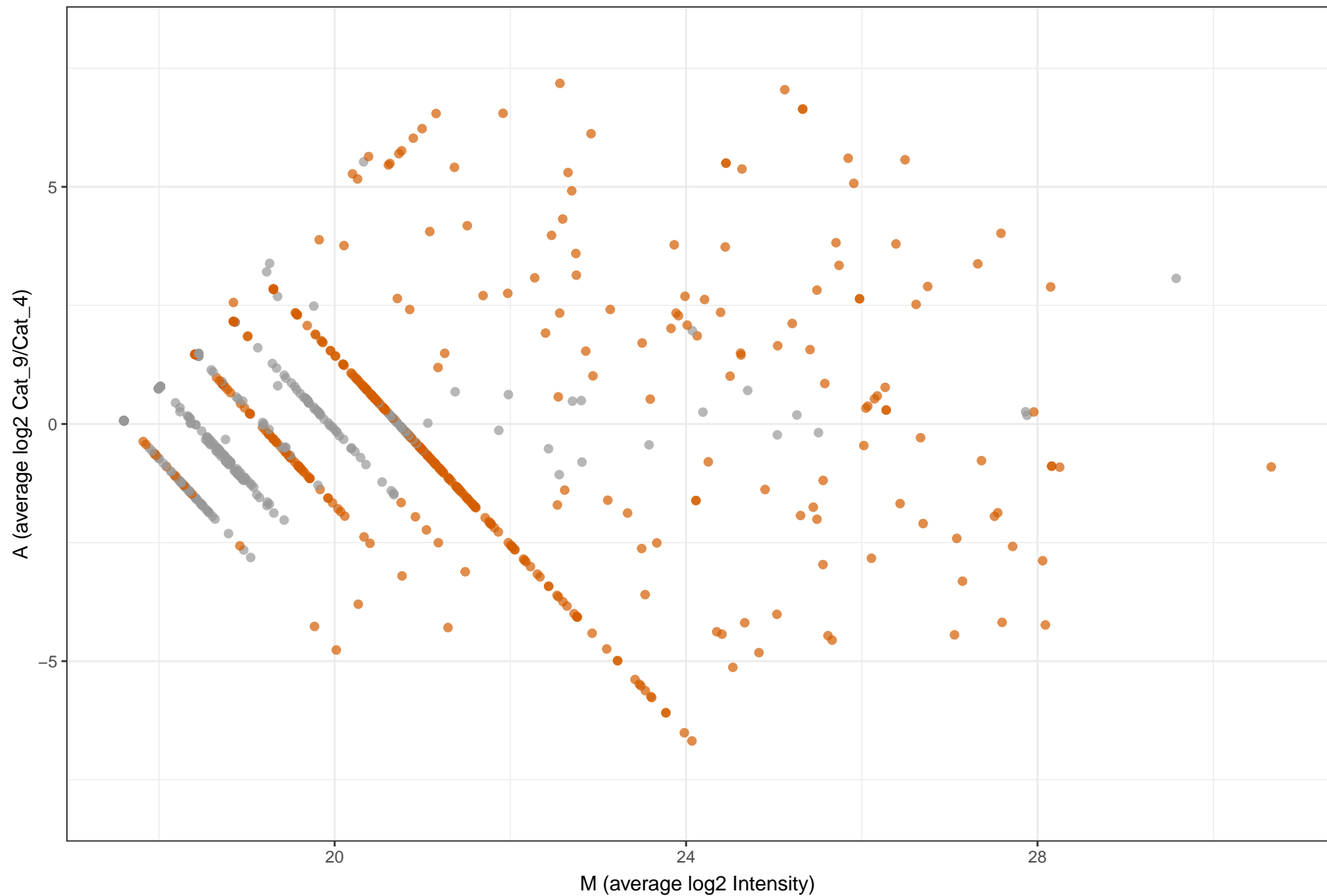

Supplement: Figure 6—source data 1. — Individual data from all figures involving small datasets displayed in individual tabs of this source file. This includes Figures 1B and 2A-F, Figure 3B, Figure 4, Figure 1—figure supplement 1 and Figure 2—figure supplement 1. [file elife-75798-fig6-data1.zip › Flores_Data/AF1_Cat_9.Cat_4-MA_AFCat1.pdf]

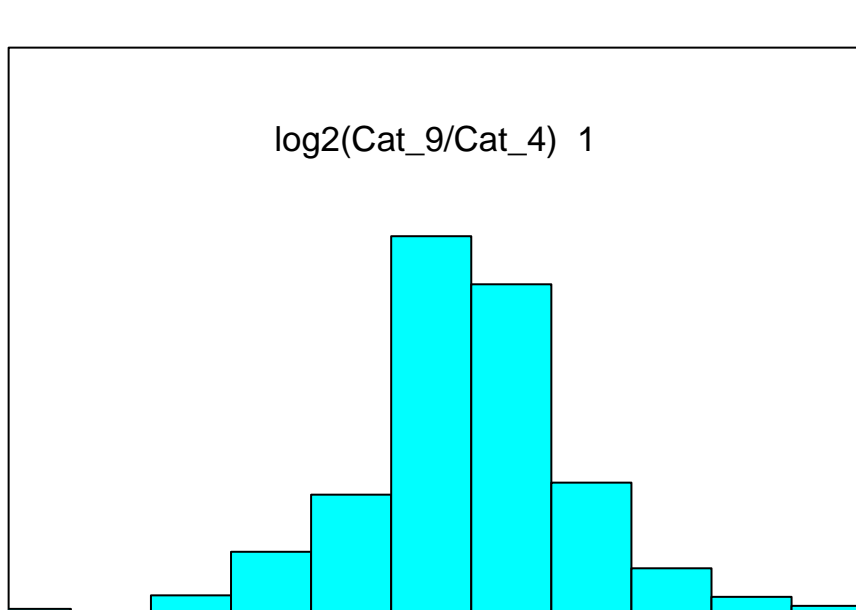

0.70

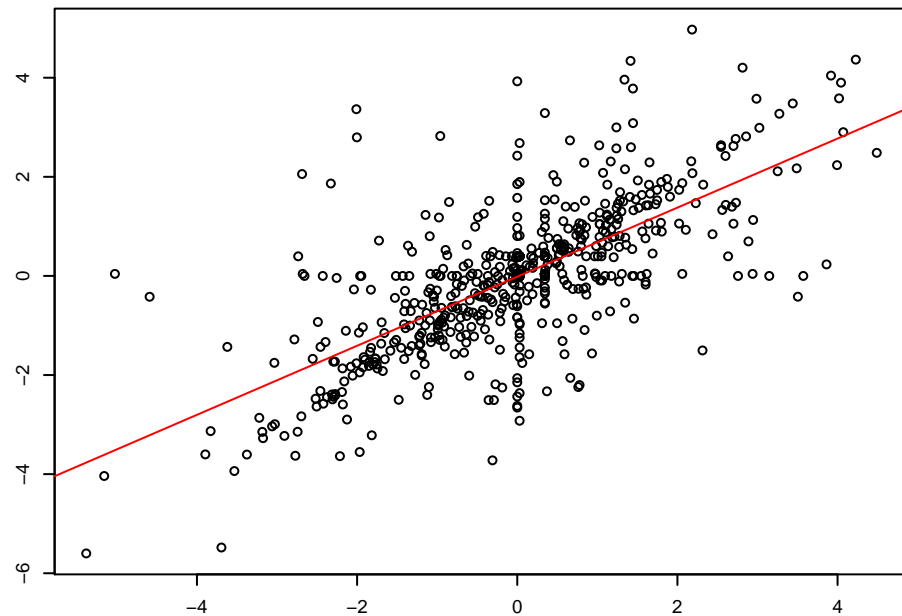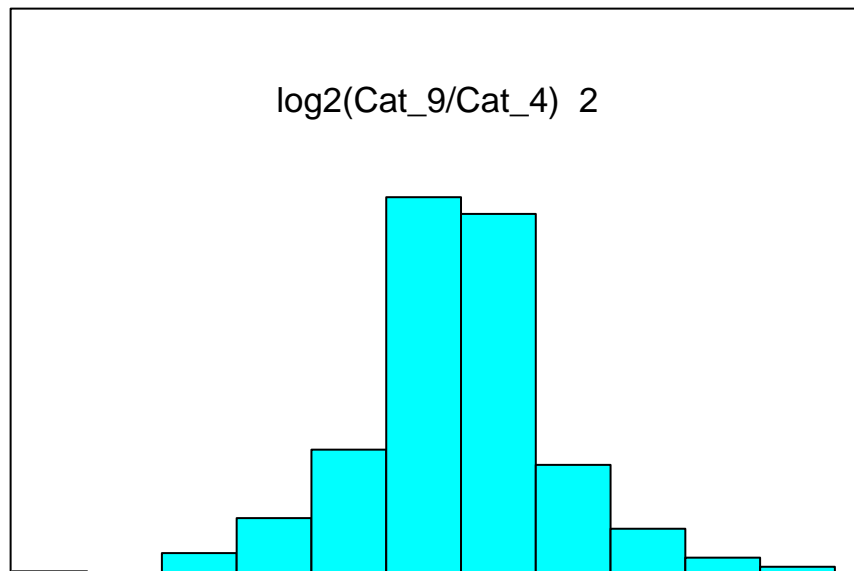

Supplement: Figure 6—source data 1. — Individual data from all figures involving small datasets displayed in individual tabs of this source file. This includes Figures 1B and 2A-F, Figure 3B, Figure 4, Figure 1—figure supplement 1 and Figure 2—figure supplement 1. [file elife-75798-fig6-data1.zip › Flores_Data/AF1_Cat_9.Cat_4-reproducibility_AFCat1.pdf]

Value-ordered fold change

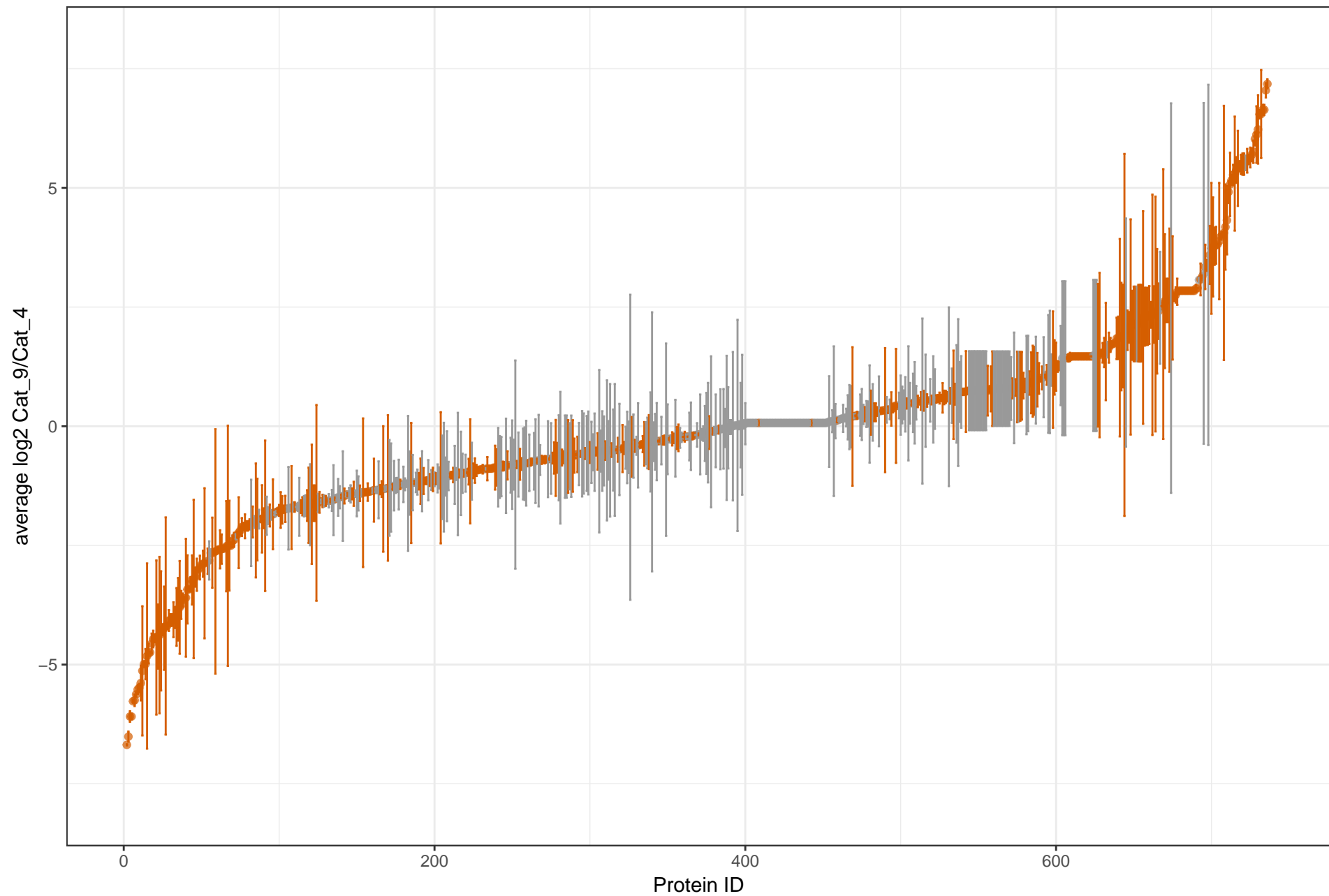

Supplement: Figure 6—source data 1. — Individual data from all figures involving small datasets displayed in individual tabs of this source file. This includes Figures 1B and 2A-F, Figure 3B, Figure 4, Figure 1—figure supplement 1 and Figure 2—figure supplement 1. [file elife-75798-fig6-data1.zip › Flores_Data/AF1_Cat_9.Cat_4-value-ordered-log-ratio_AFCat1.pdf]

**P-value vs Fold change**

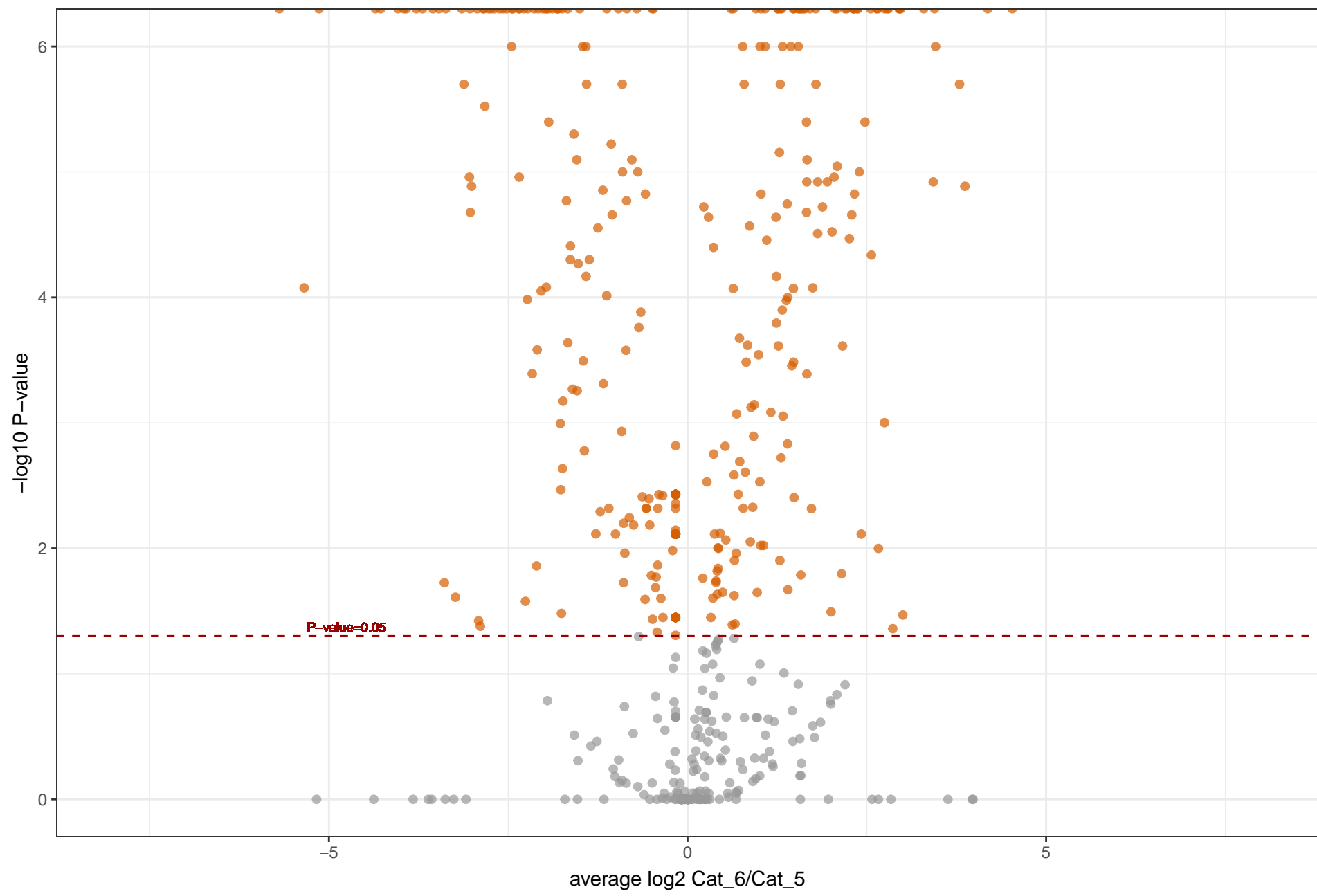

Supplement: Figure 6—source data 1. — Individual data from all figures involving small datasets displayed in individual tabs of this source file. This includes Figures 1B and 2A-F, Figure 3B, Figure 4, Figure 1—figure supplement 1 and Figure 2—figure supplement 1. [file elife-75798-fig6-data1.zip › Flores_Data/AF1_Cat_6.Cat_5-volcano_AFCat1.pdf]

Value-ordered fold change

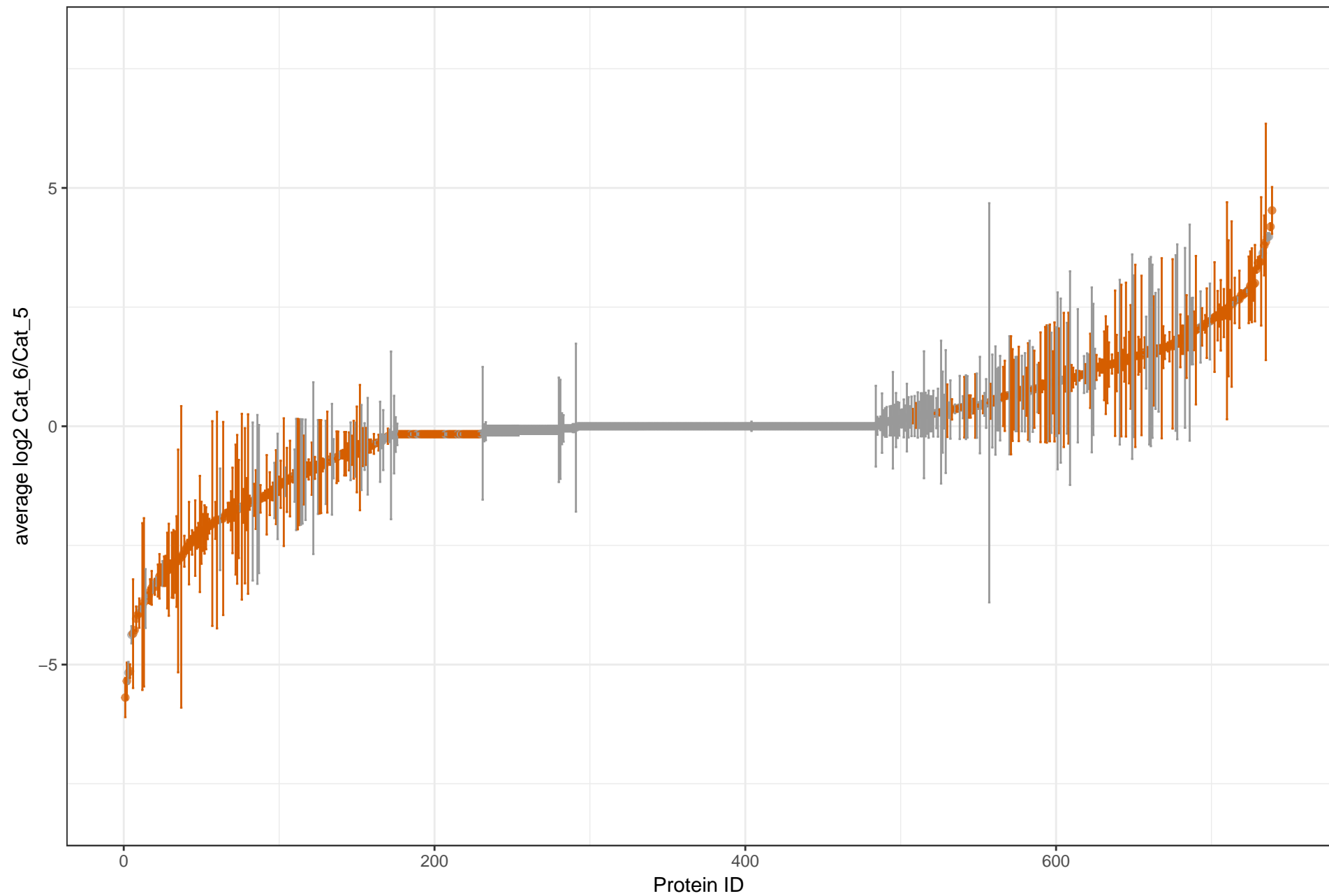

Supplement: Figure 6—source data 1. — Individual data from all figures involving small datasets displayed in individual tabs of this source file. This includes Figures 1B and 2A-F, Figure 3B, Figure 4, Figure 1—figure supplement 1 and Figure 2—figure supplement 1. [file elife-75798-fig6-data1.zip › Flores_Data/AF1_Cat_6.Cat_5-value-ordered-log-ratio_AFCat1.pdf]

P-value vs Fold change

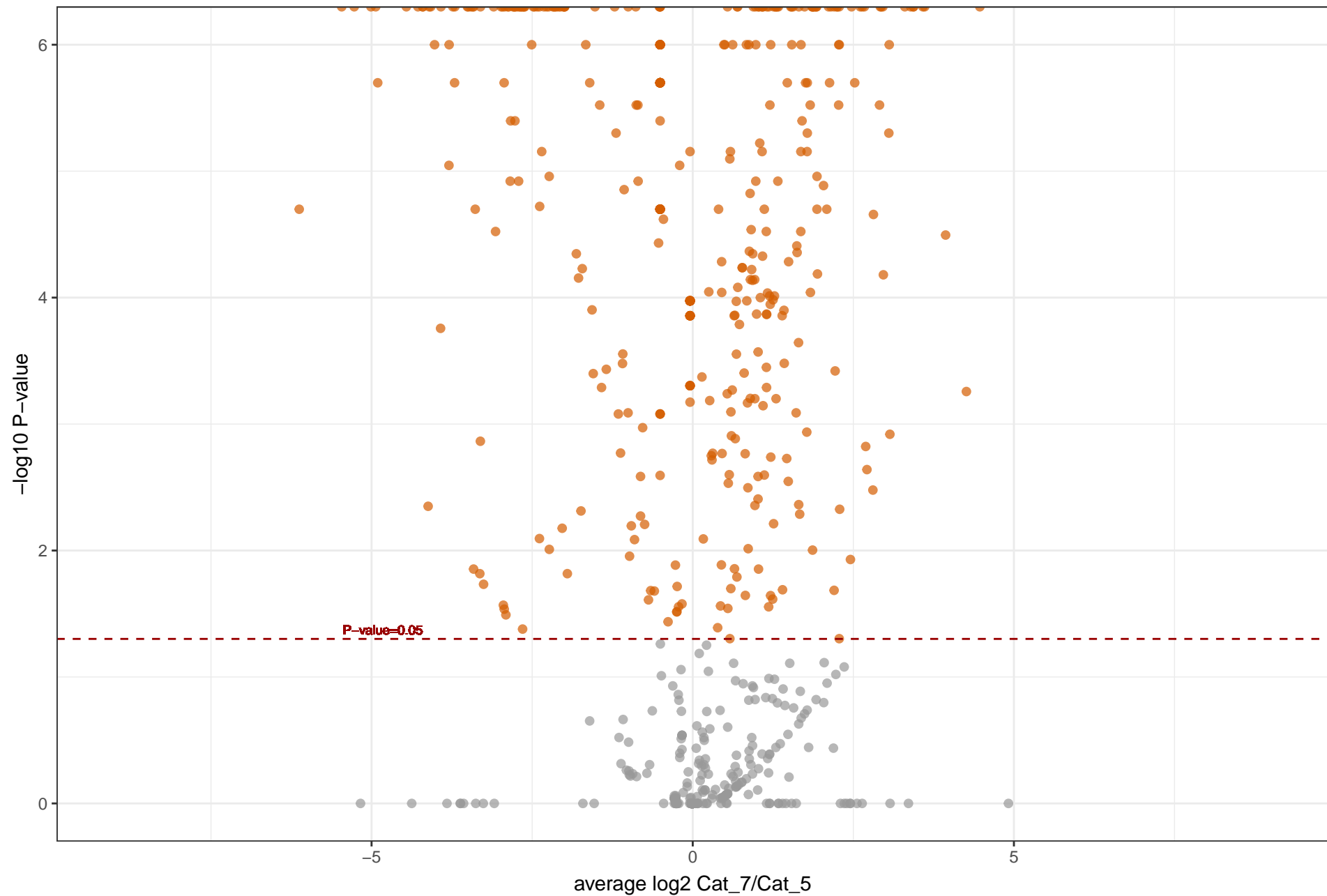

Supplement: Figure 6—source data 1. — Individual data from all figures involving small datasets displayed in individual tabs of this source file. This includes Figures 1B and 2A-F, Figure 3B, Figure 4, Figure 1—figure supplement 1 and Figure 2—figure supplement 1. [file elife-75798-fig6-data1.zip › Flores_Data/AF1_Cat_7.Cat_5-volcano_AFCat1.pdf]

MA plot

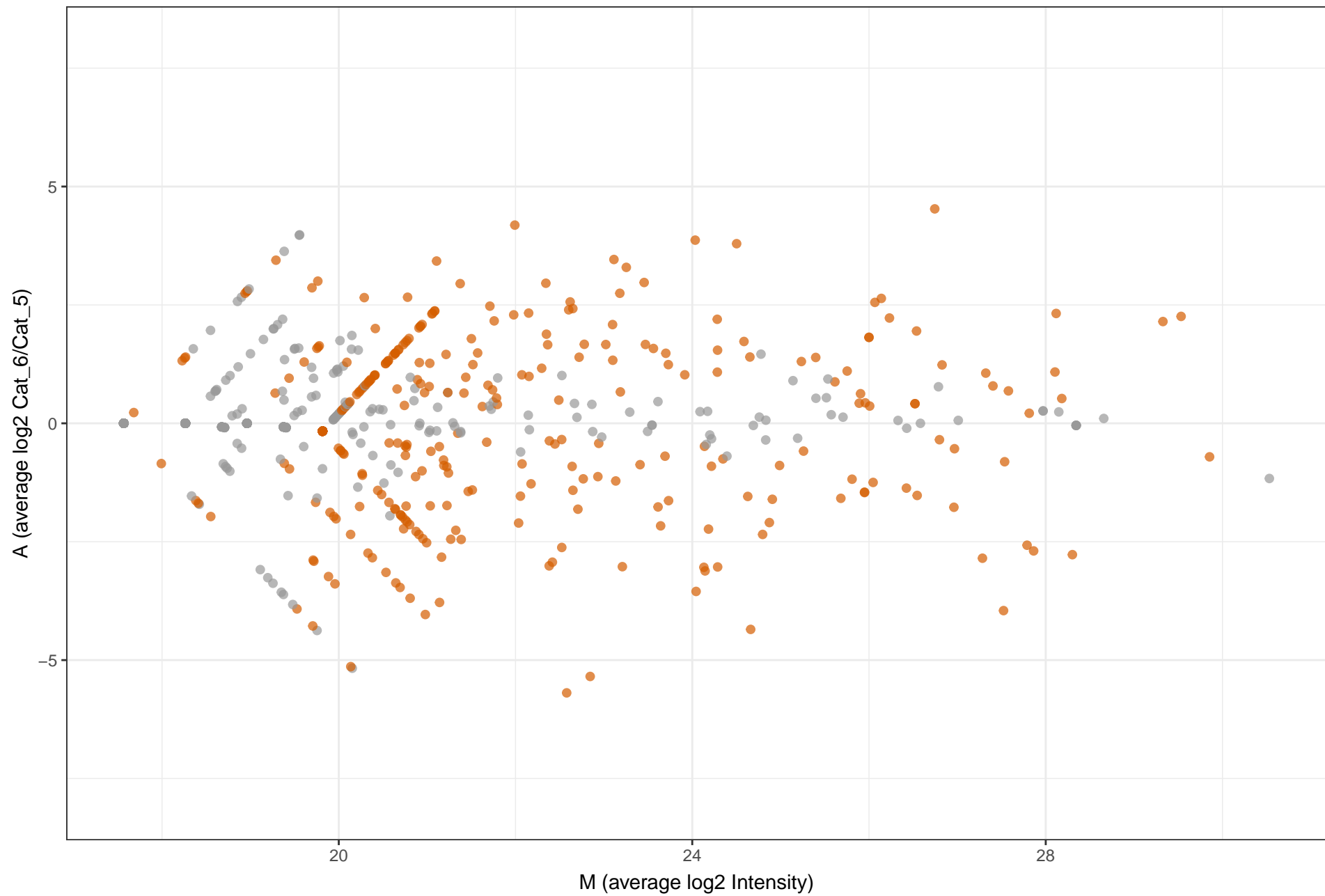

Supplement: Figure 6—source data 1. — Individual data from all figures involving small datasets displayed in individual tabs of this source file. This includes Figures 1B and 2A-F, Figure 3B, Figure 4, Figure 1—figure supplement 1 and Figure 2—figure supplement 1. [file elife-75798-fig6-data1.zip › Flores_Data/AF1_Cat_6.Cat_5-MA_AFCat1.pdf]

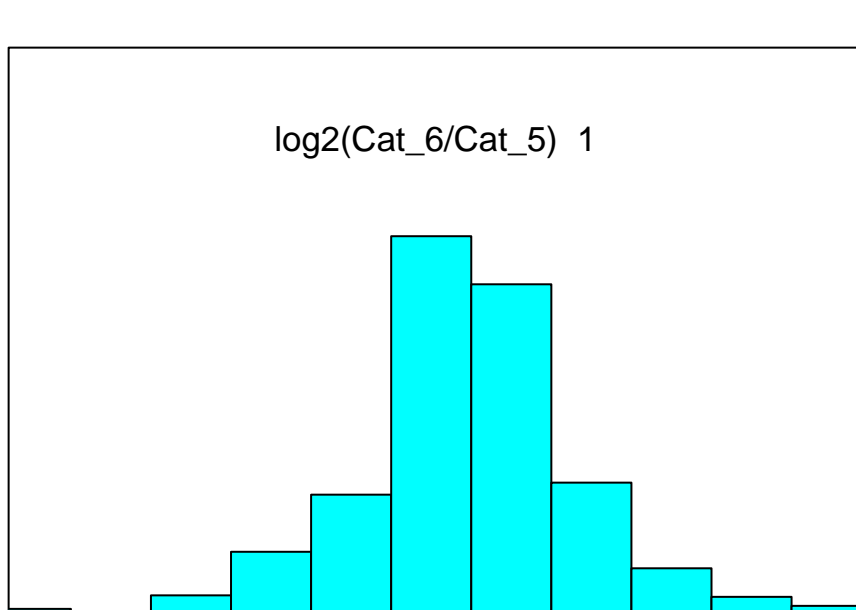

0.70

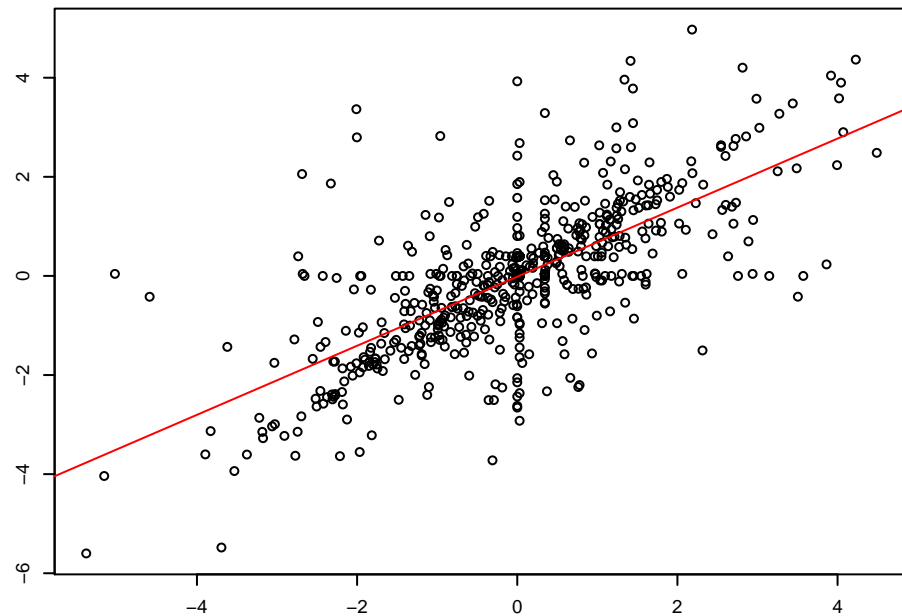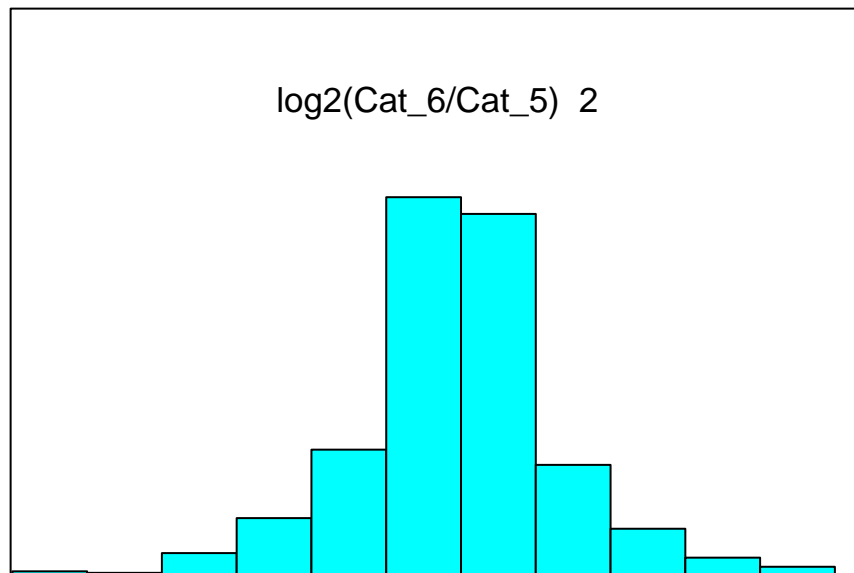

Supplement: Figure 6—source data 1. — Individual data from all figures involving small datasets displayed in individual tabs of this source file. This includes Figures 1B and 2A-F, Figure 3B, Figure 4, Figure 1—figure supplement 1 and Figure 2—figure supplement 1. [file elife-75798-fig6-data1.zip › Flores_Data/AF1_Cat_6.Cat_5-reproducibility_AFCat1.pdf]

MA plot

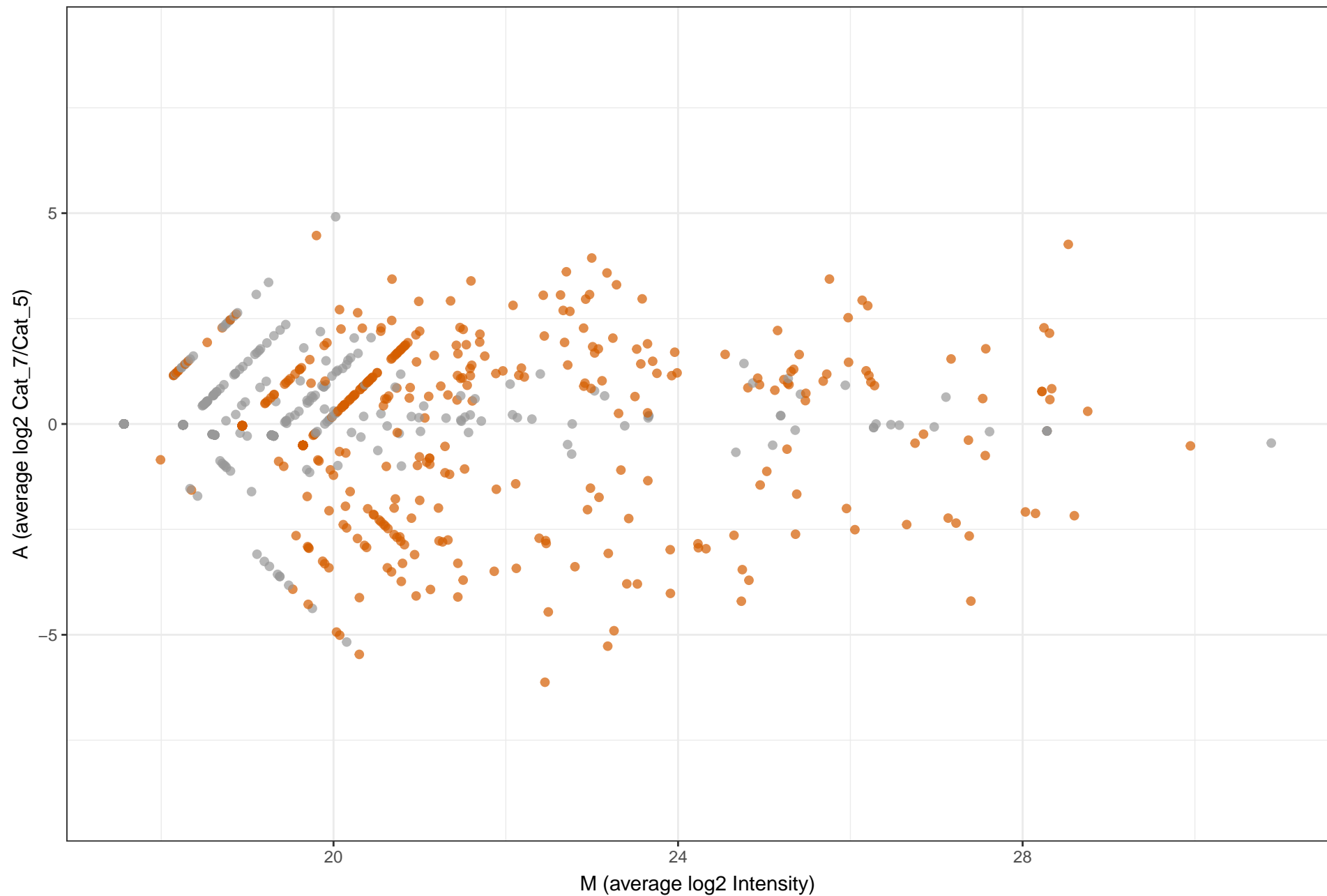

Supplement: Figure 6—source data 1. — Individual data from all figures involving small datasets displayed in individual tabs of this source file. This includes Figures 1B and 2A-F, Figure 3B, Figure 4, Figure 1—figure supplement 1 and Figure 2—figure supplement 1. [file elife-75798-fig6-data1.zip › Flores_Data/AF1_Cat_7.Cat_5-MA_AFCat1.pdf]

Value-ordered fold change

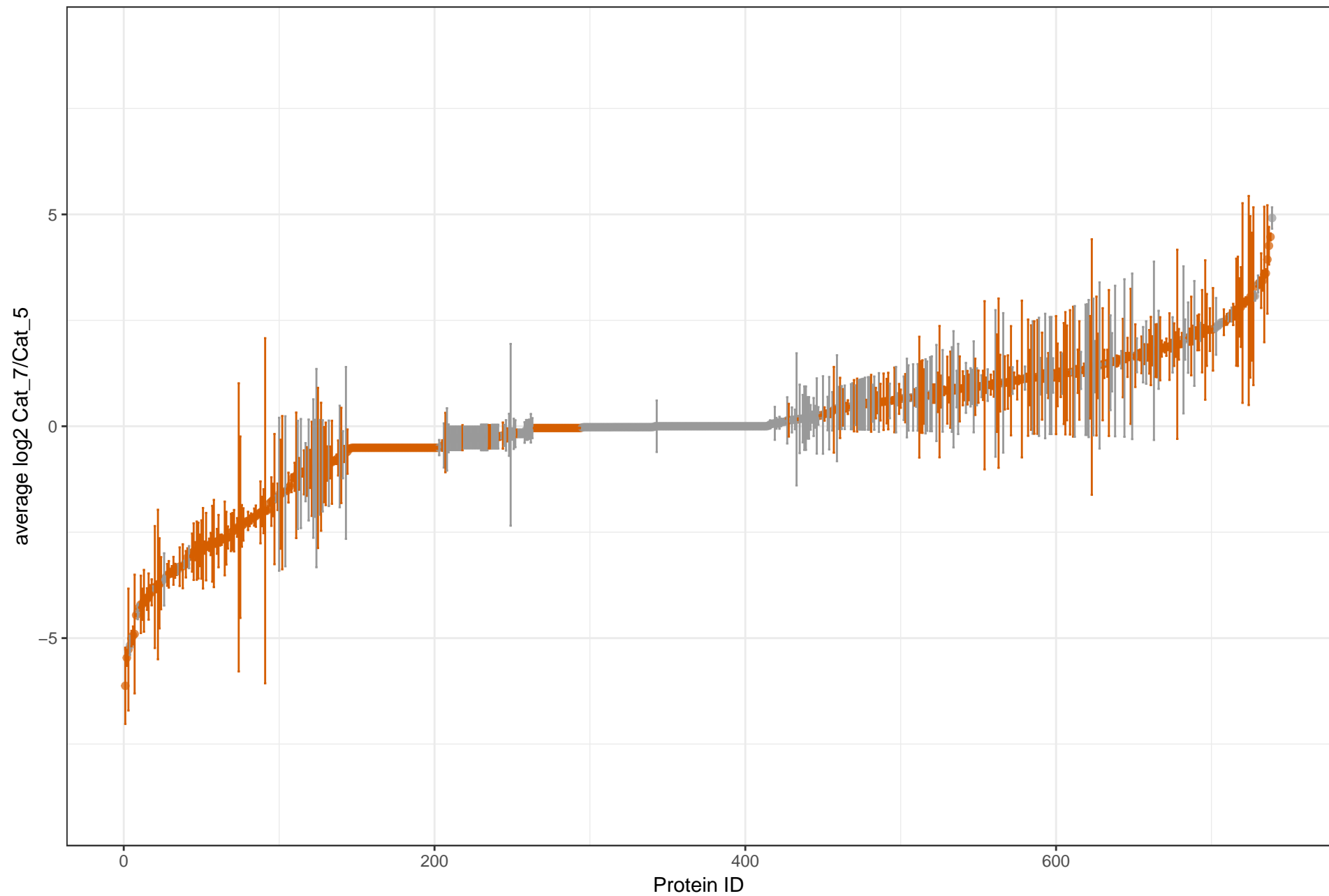

Supplement: Figure 6—source data 1. — Individual data from all figures involving small datasets displayed in individual tabs of this source file. This includes Figures 1B and 2A-F, Figure 3B, Figure 4, Figure 1—figure supplement 1 and Figure 2—figure supplement 1. [file elife-75798-fig6-data1.zip › Flores_Data/AF1_Cat_7.Cat_5-value-ordered-log-ratio_AFCat1.pdf]

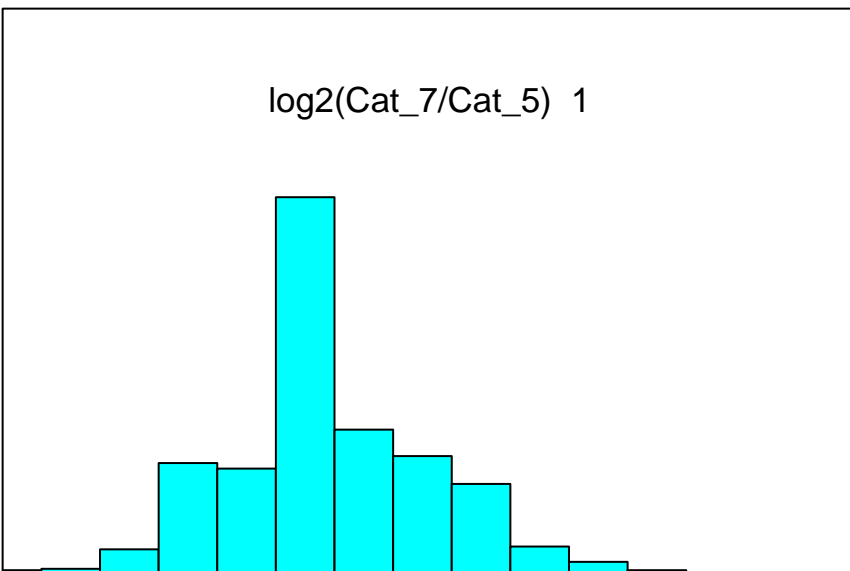

0.79

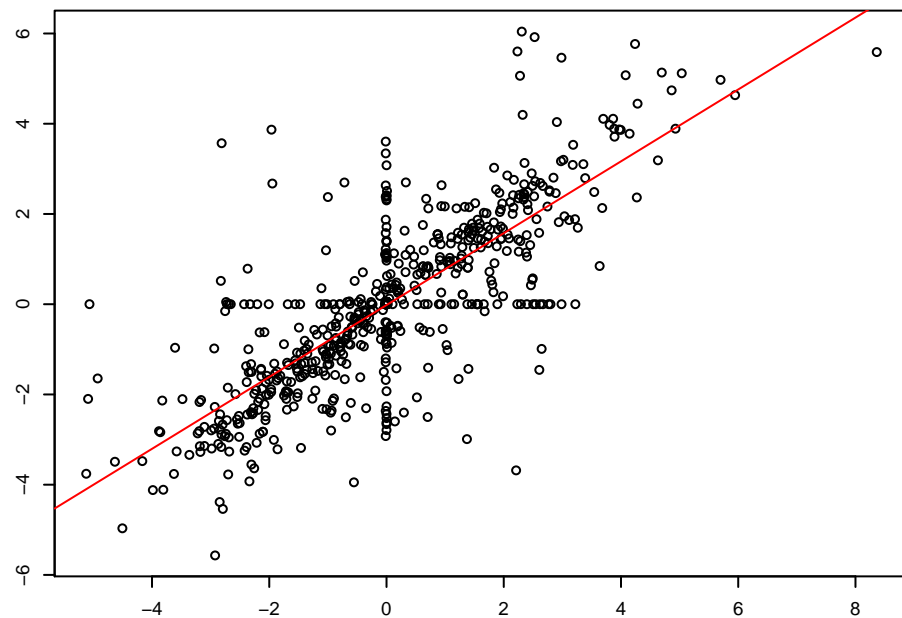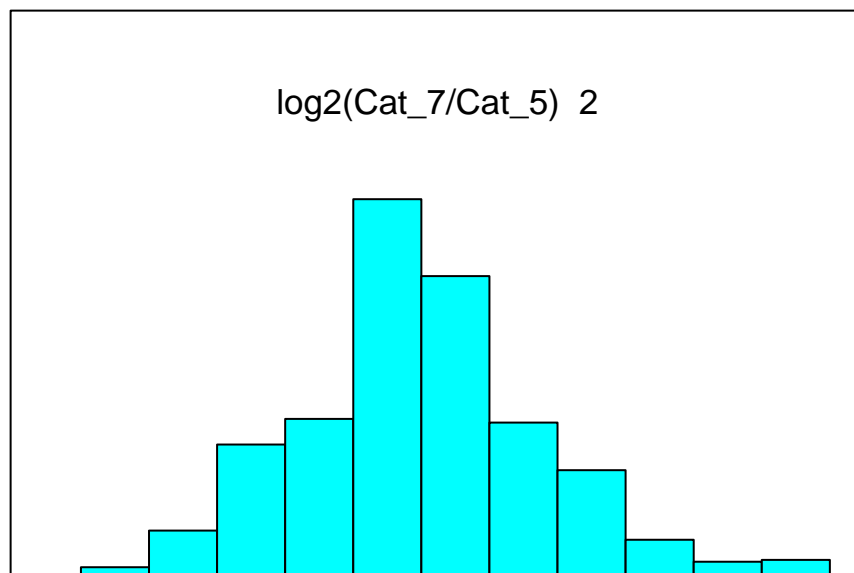

Supplement: Figure 6—source data 1. — Individual data from all figures involving small datasets displayed in individual tabs of this source file. This includes Figures 1B and 2A-F, Figure 3B, Figure 4, Figure 1—figure supplement 1 and Figure 2—figure supplement 1. [file elife-75798-fig6-data1.zip › Flores_Data/AF1_Cat_7.Cat_5-reproducibility_AFCat1.pdf]

P-value vs Fold change

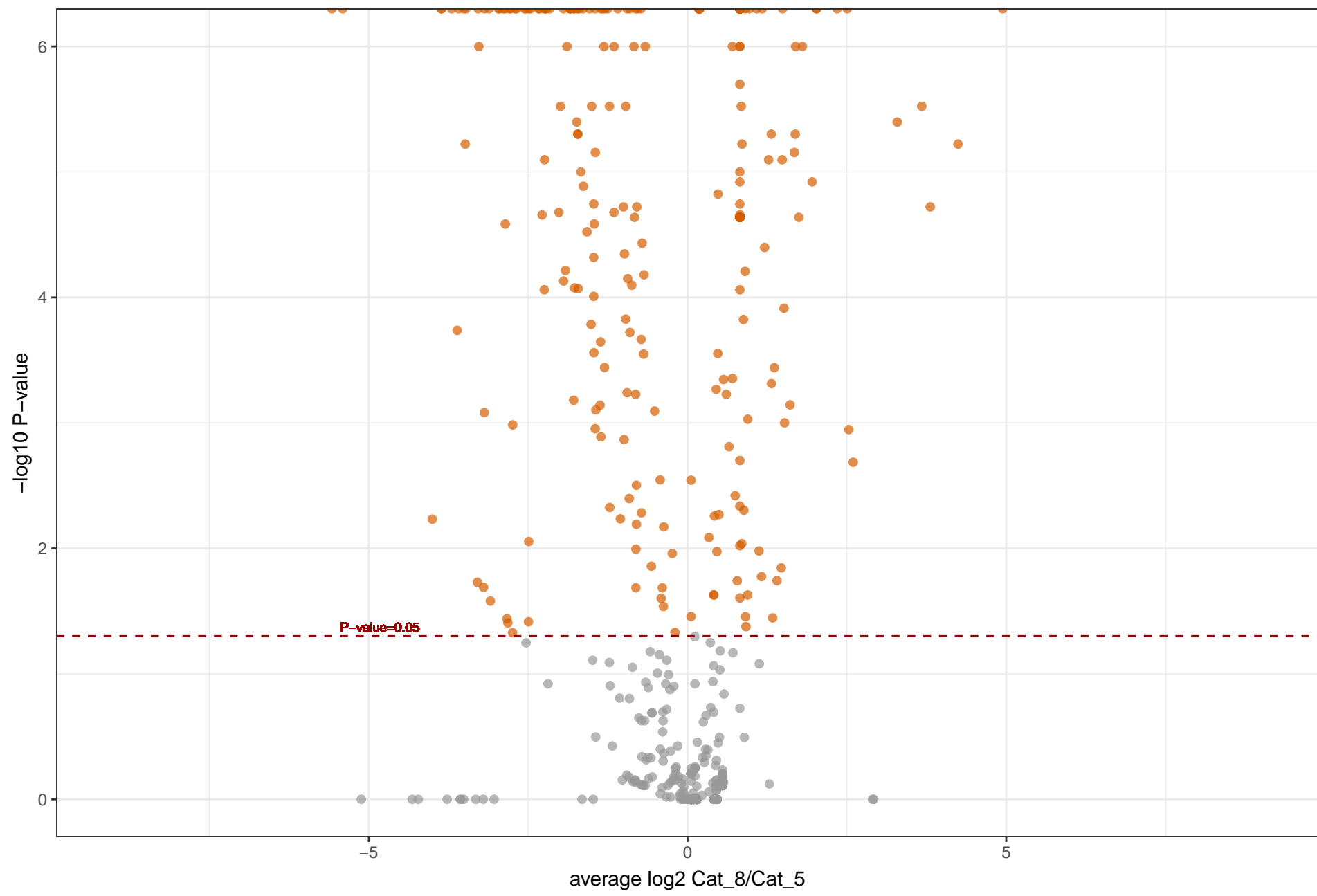

Supplement: Figure 6—source data 1. — Individual data from all figures involving small datasets displayed in individual tabs of this source file. This includes Figures 1B and 2A-F, Figure 3B, Figure 4, Figure 1—figure supplement 1 and Figure 2—figure supplement 1. [file elife-75798-fig6-data1.zip › Flores_Data/AF1_Cat_8.Cat_5-volcano_AFCat1.pdf]

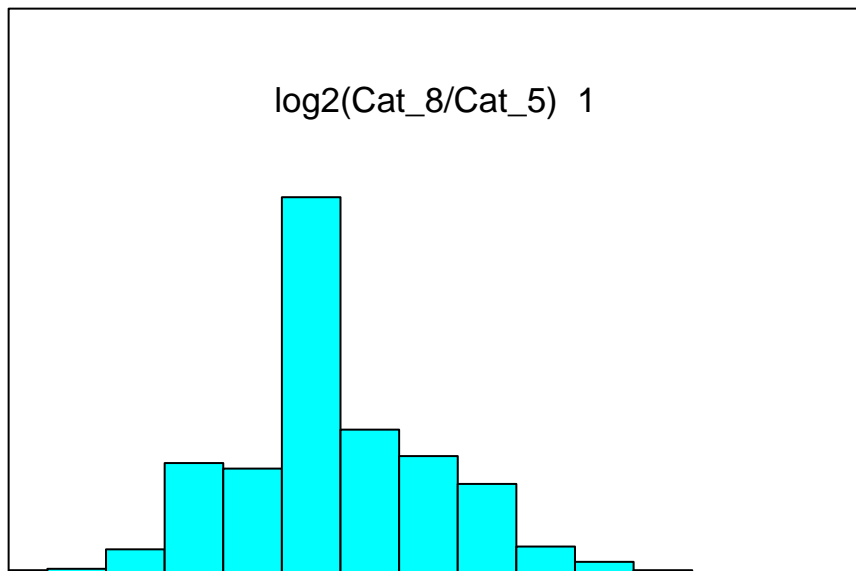

0.79

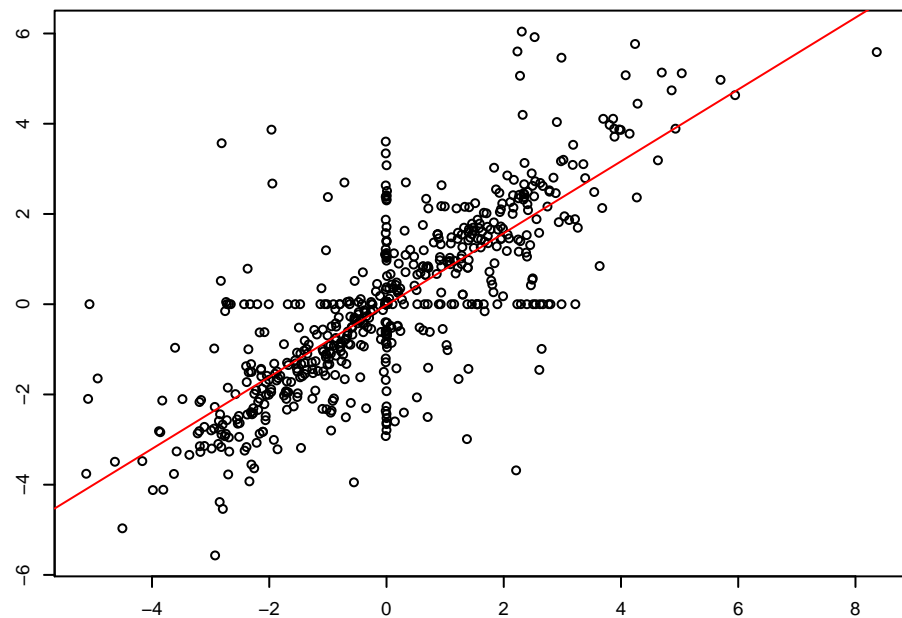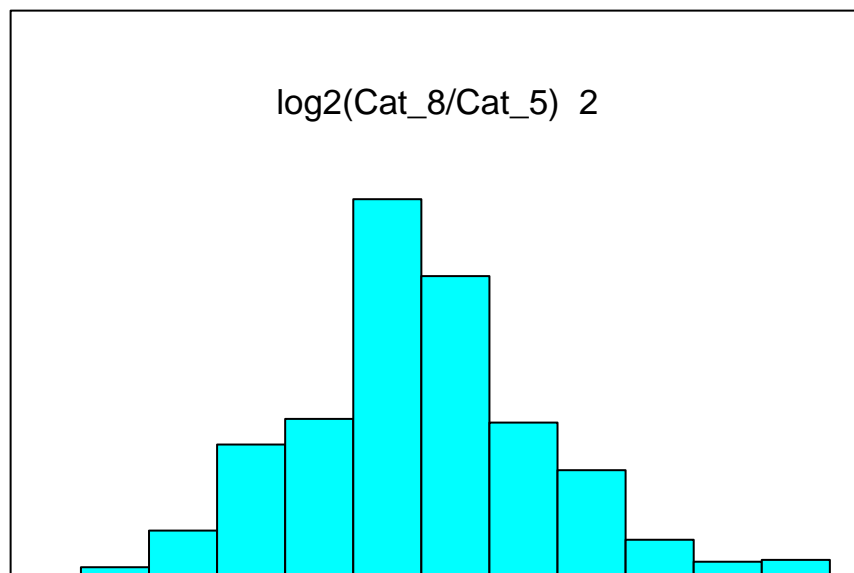

Supplement: Figure 6—source data 1. — Individual data from all figures involving small datasets displayed in individual tabs of this source file. This includes Figures 1B and 2A-F, Figure 3B, Figure 4, Figure 1—figure supplement 1 and Figure 2—figure supplement 1. [file elife-75798-fig6-data1.zip › Flores_Data/AF1_Cat_8.Cat_5-reproducibility_AFCat1.pdf]

Value-ordered fold change

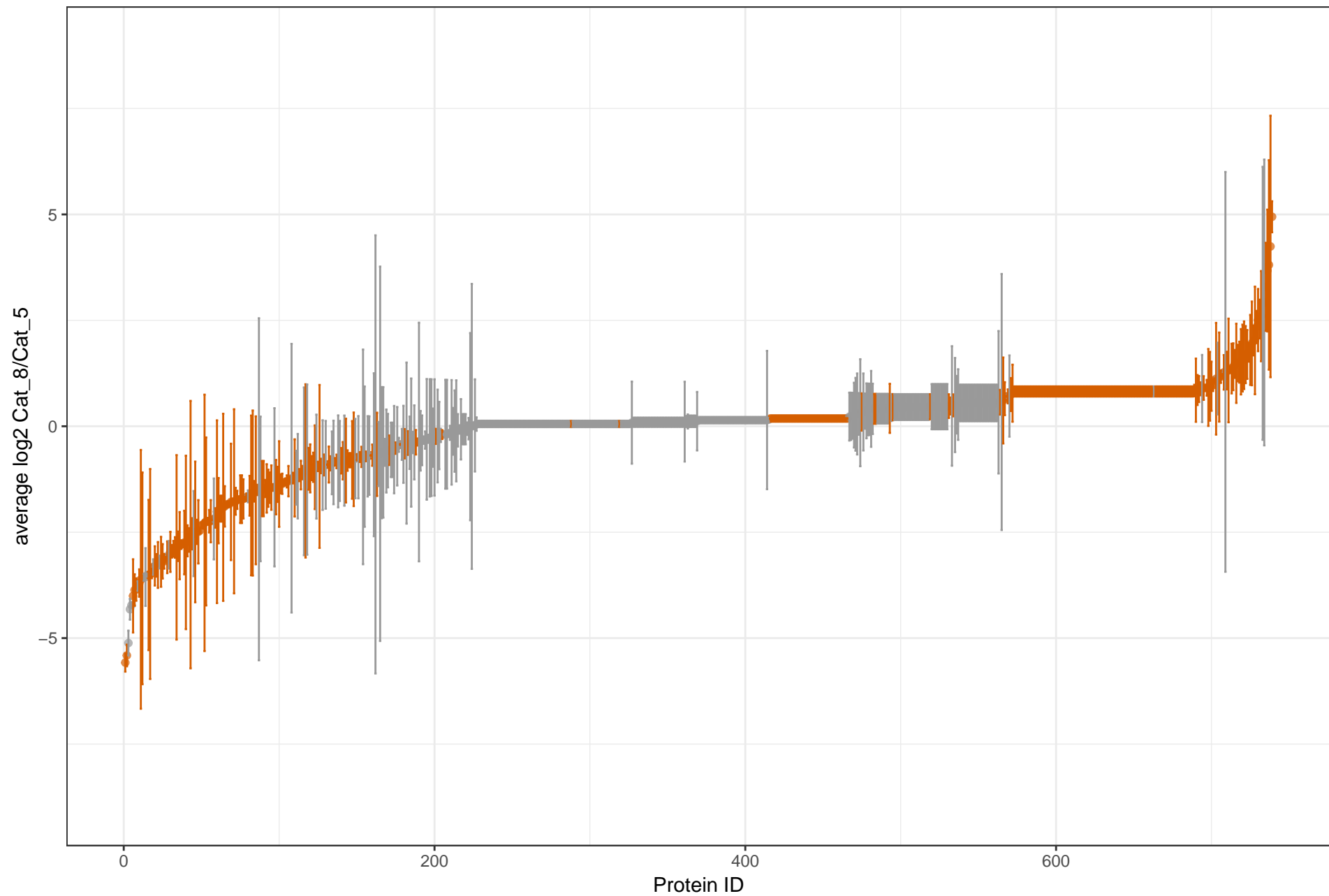

Supplement: Figure 6—source data 1. — Individual data from all figures involving small datasets displayed in individual tabs of this source file. This includes Figures 1B and 2A-F, Figure 3B, Figure 4, Figure 1—figure supplement 1 and Figure 2—figure supplement 1. [file elife-75798-fig6-data1.zip › Flores_Data/AF1_Cat_8.Cat_5-value-ordered-log-ratio_AFCat1.pdf]

MA plot

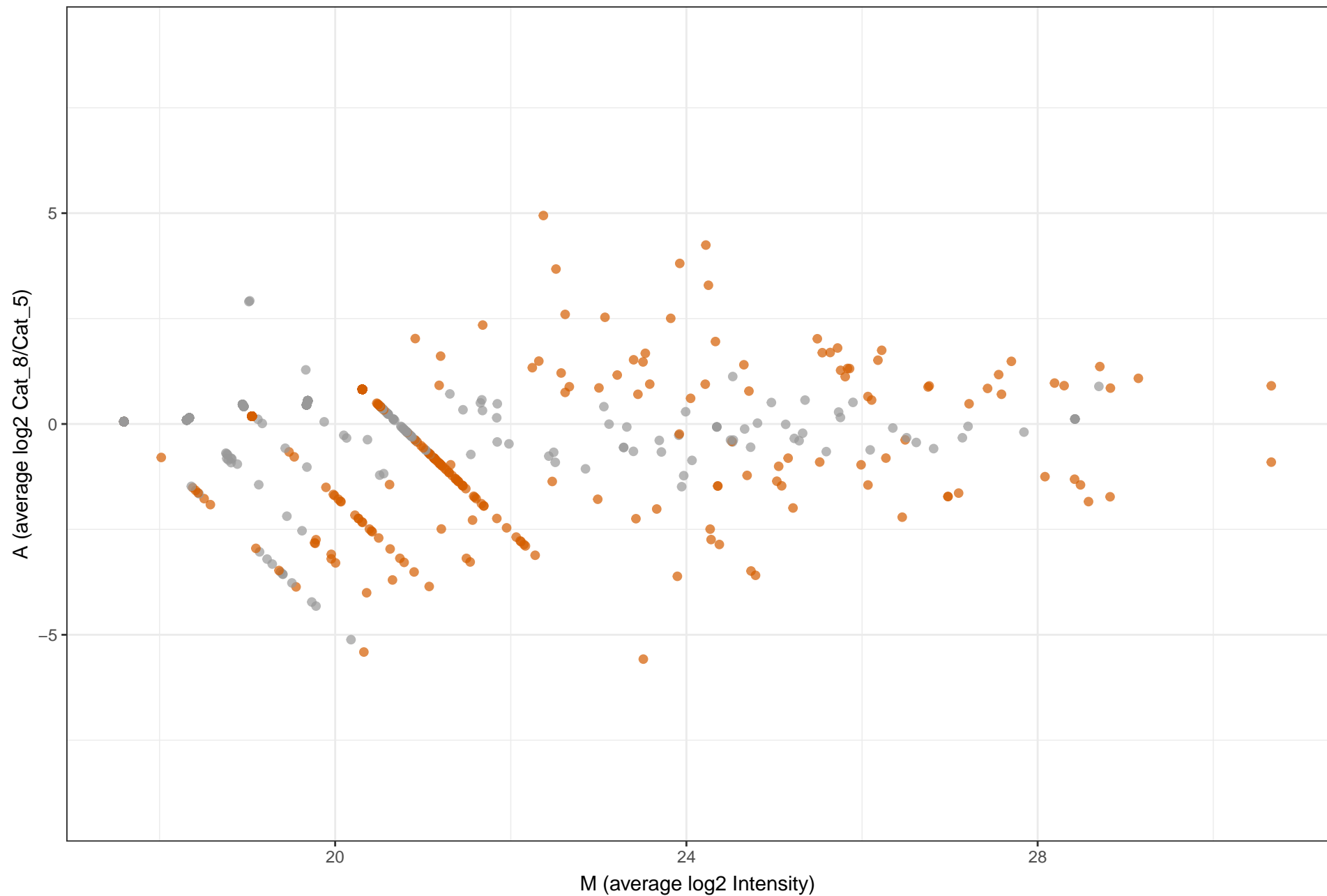

Supplement: Figure 6—source data 1. — Individual data from all figures involving small datasets displayed in individual tabs of this source file. This includes Figures 1B and 2A-F, Figure 3B, Figure 4, Figure 1—figure supplement 1 and Figure 2—figure supplement 1. [file elife-75798-fig6-data1.zip › Flores_Data/AF1_Cat_8.Cat_5-MA_AFCat1.pdf]

P-value vs Fold change

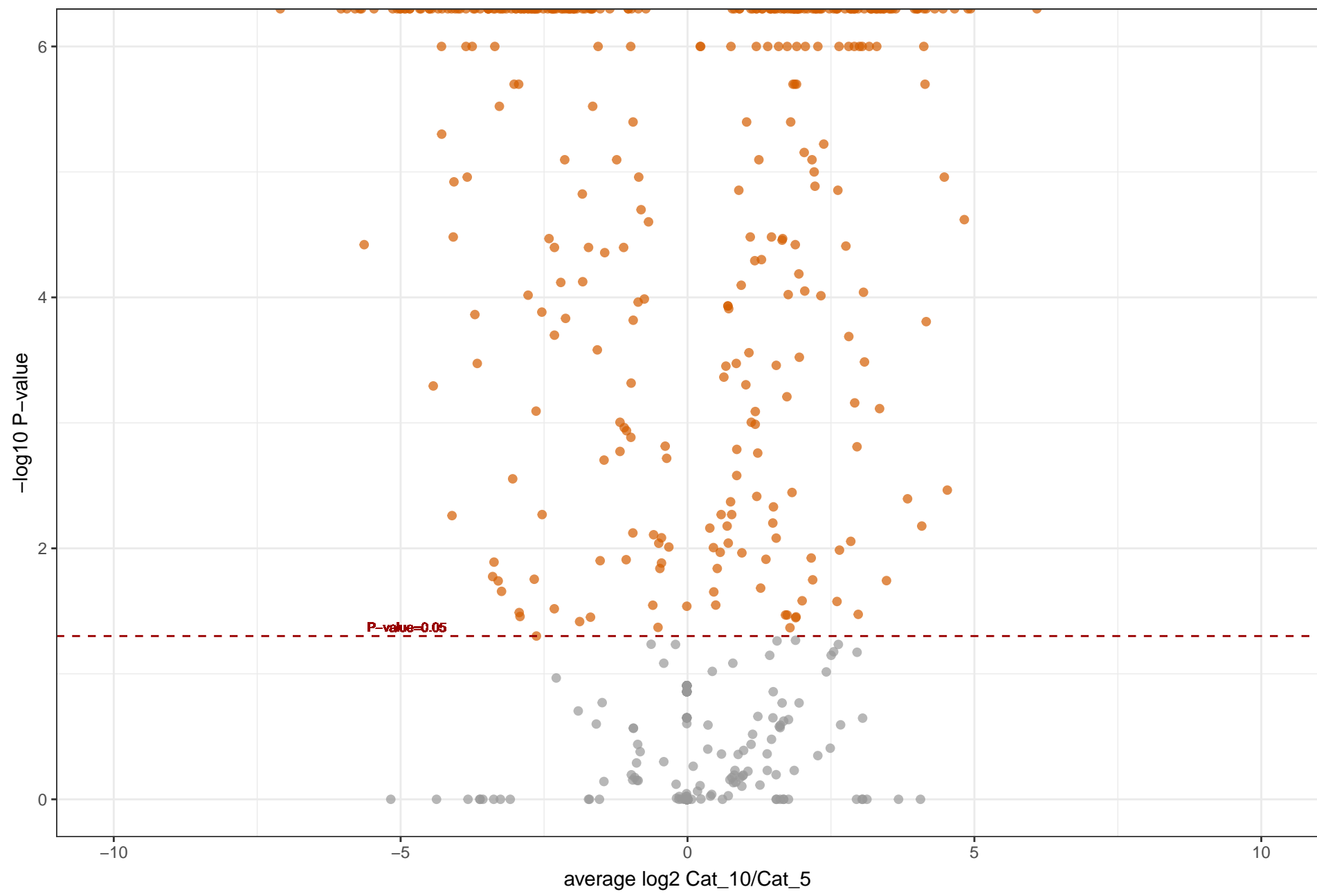

Supplement: Figure 6—source data 1. — Individual data from all figures involving small datasets displayed in individual tabs of this source file. This includes Figures 1B and 2A-F, Figure 3B, Figure 4, Figure 1—figure supplement 1 and Figure 2—figure supplement 1. [file elife-75798-fig6-data1.zip › Flores_Data/AF1_Cat_10.Cat_5-volcano_AFCat1.pdf]

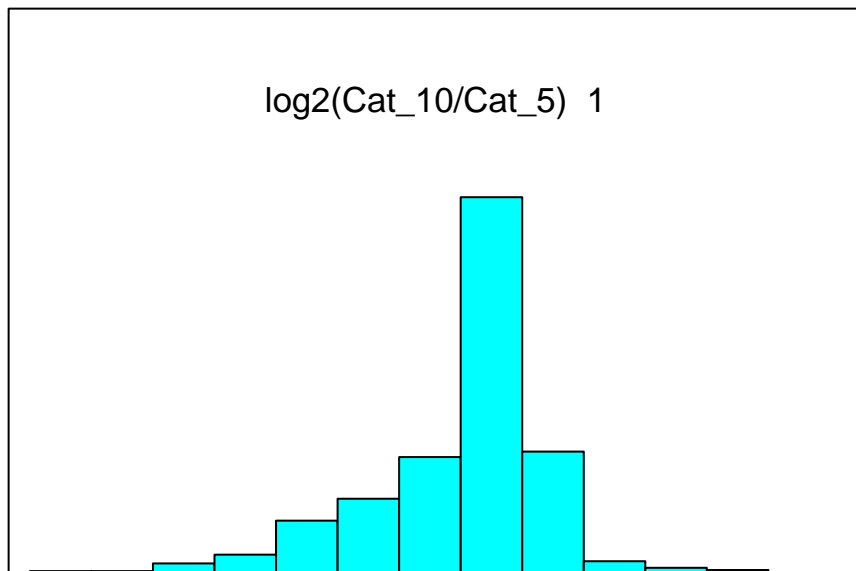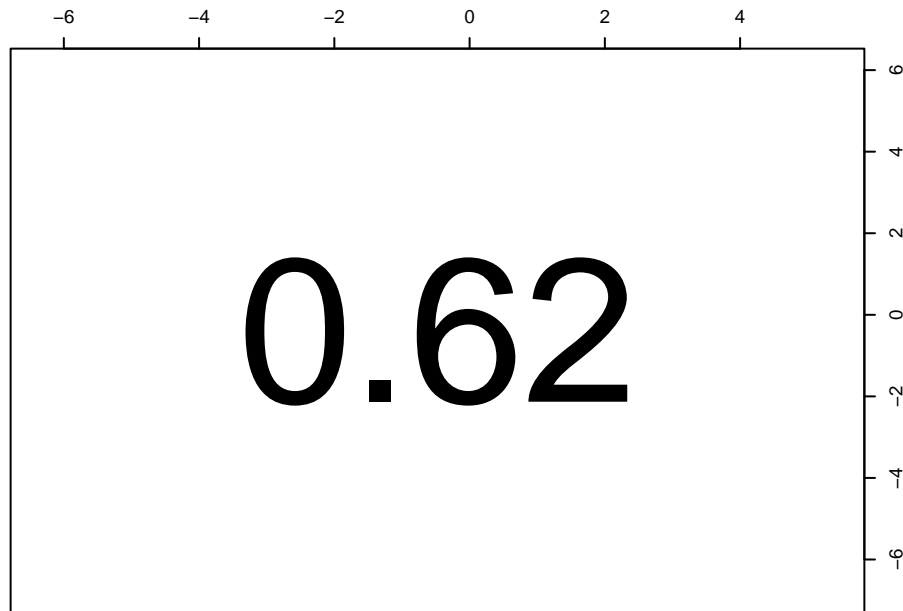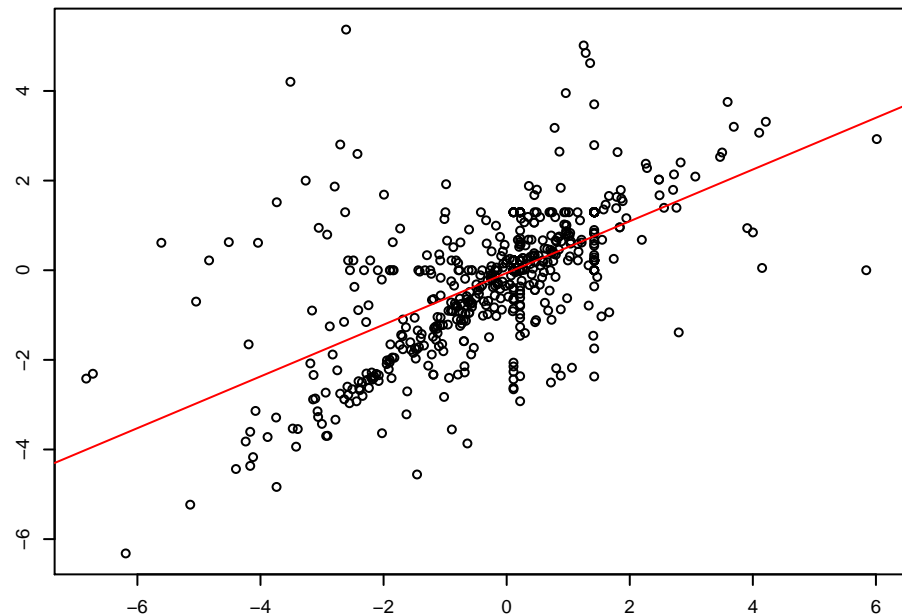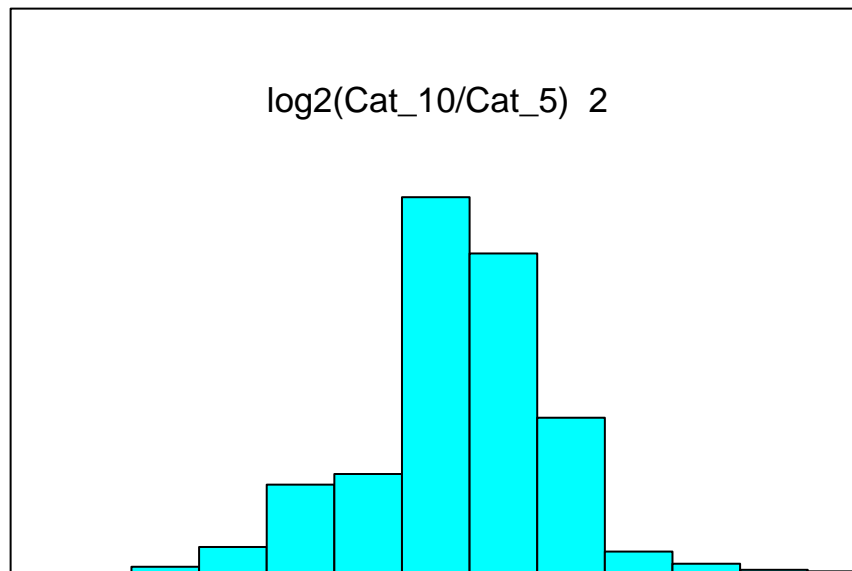

Supplement: Figure 6—source data 1. — Individual data from all figures involving small datasets displayed in individual tabs of this source file. This includes Figures 1B and 2A-F, Figure 3B, Figure 4, Figure 1—figure supplement 1 and Figure 2—figure supplement 1. [file elife-75798-fig6-data1.zip › Flores_Data/AF1_Cat_10.Cat_5-reproducibility_AFCat1.pdf]

Value-ordered fold change

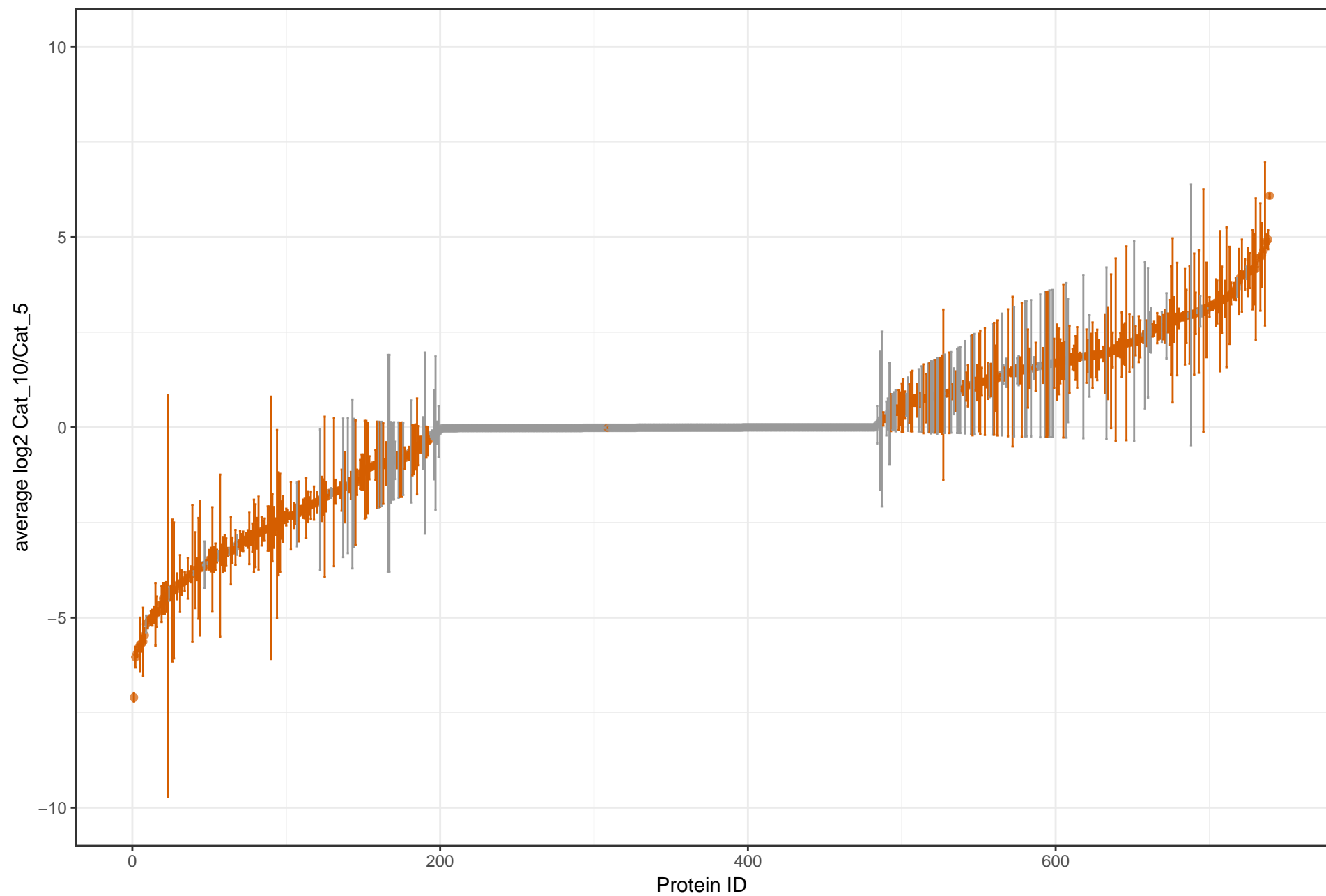

Supplement: Figure 6—source data 1. — Individual data from all figures involving small datasets displayed in individual tabs of this source file. This includes Figures 1B and 2A-F, Figure 3B, Figure 4, Figure 1—figure supplement 1 and Figure 2—figure supplement 1. [file elife-75798-fig6-data1.zip › Flores_Data/AF1_Cat_10.Cat_5-value-ordered-log-ratio_AFCat1.pdf]

MA plot

A (average log2 Cat\_10/Cat\_5)

10  
5  
0  
-5  
-10

20

24

28

M (average log2 Intensity)

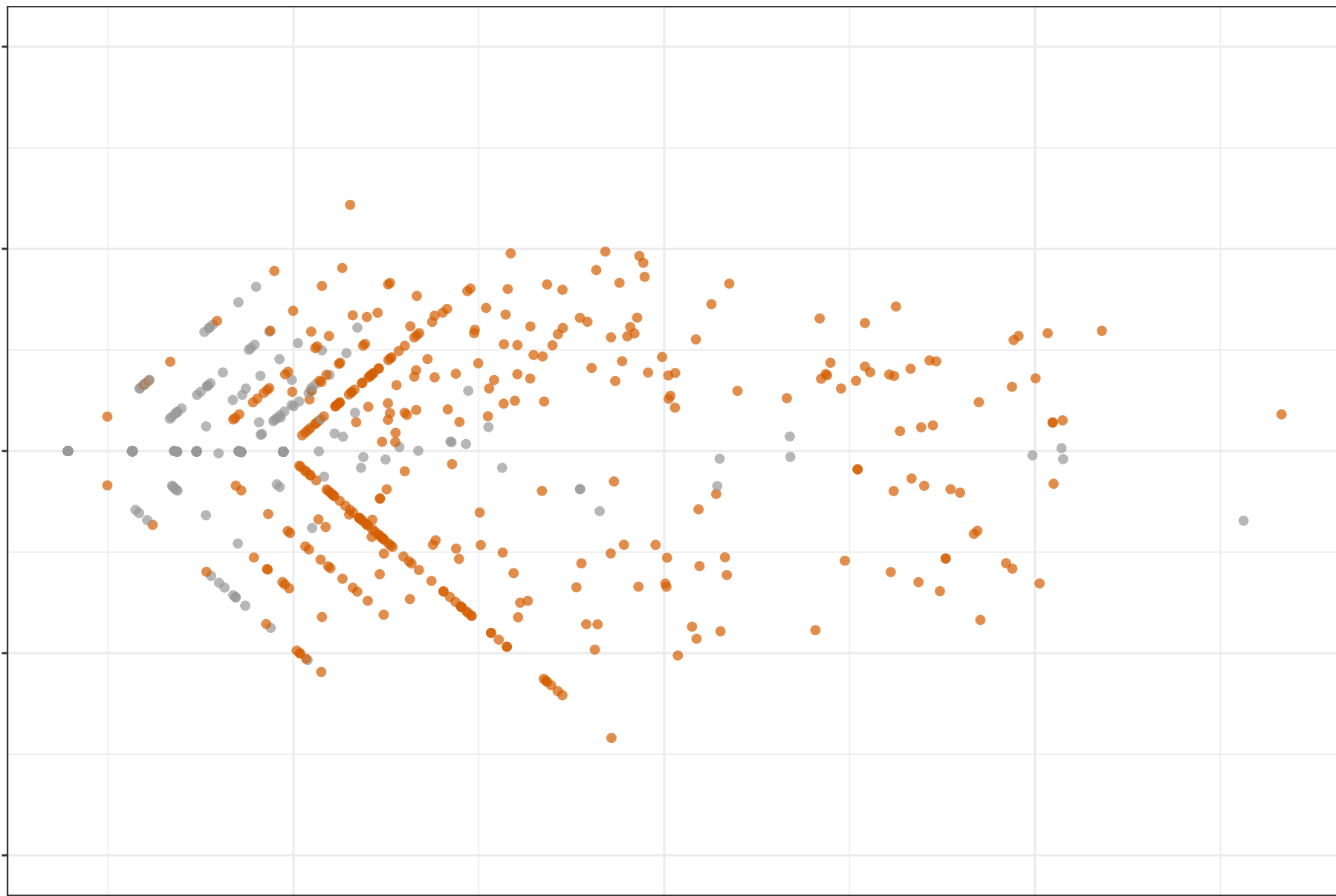

Supplement: Figure 6—source data 1. — Individual data from all figures involving small datasets displayed in individual tabs of this source file. This includes Figures 1B and 2A-F, Figure 3B, Figure 4, Figure 1—figure supplement 1 and Figure 2—figure supplement 1. [file elife-75798-fig6-data1.zip › Flores_Data/AF1_Cat_10.Cat_5-MA_AFCat1.pdf]
